# Social and genetic diversity in first farmers of central Europe

**DOI:** 10.1038/s41562-024-02034-z

**Published:** 2024-11-29

**Authors:** Pere Gelabert, Penny Bickle, Daniela Hofmann, Maria Teschler-Nicola, Alexandra Anders, Xin Huang, Michelle Hämmerle, Iñigo Olalde, Romain Fournier, Harald Ringbauer, Ali Akbari, Olivia Cheronet, Iosif Lazaridis, Nasreen Broomandkhoshbacht, Daniel M. Fernandes, Katharina Buttinger, Kim Callan, Francesca Candilio, Guillermo Bravo Morante, Elizabeth Curtis, Matthew Ferry, Denise Keating, Suzanne Freilich, Aisling Kearns, Éadaoin Harney, Ann Marie Lawson, Kirsten Mandl, Megan Michel, Victoria Oberreiter, Brina Zagorc, Jonas Oppenheimer, Susanna Sawyer, Constanze Schattke, Kadir Toykan Özdoğan, Lijun Qiu, J. Noah Workman, Fatma Zalzala, Swapan Mallick, Matthew Mah, Adam Micco, Franz Pieler, Juraj Pavuk, Alena Šefčáková, Catalin Lazar, Andrej Starović, Marija Djuric, Maja Krznarić Škrivanko, Mario Šlaus, Željka Bedić, Friederike Novotny, László D. Szabó, Orsolya Cserpák-Laczi, Tamara Hága, László Szolnoki, Zsigmond Hajdú, Pavel Mirea, Emese Gyöngyvér Nagy, Zsuzsanna M. Virág, Attila M. Horváth, László András Horváth, Katalin T. Biró, László Domboróczki, Tamás Szeniczey, János Jakucs, Márta Szelekovszky, Zoltán Farkas, Sándor József Sztáncsuj, Krisztián Tóth, Piroska Csengeri, Ildikó Pap, Róbert Patay, Anđelka Putica, Branislav Vasov, Bálint Havasi, Katalin Sebők, Pál Raczky, Gabriella Lovász, Zdeněk Tvrdý, Nadin Rohland, Mario Novak, Matej Ruttkay, Maria Krošláková, Jozef Bátora, Tibor Paluch, Dušan Borić, János Dani, Martin Kuhlwilm, Pier Francesco Palamara, Tamás Hajdu, Ron Pinhasi, David Reich

**Affiliations:** 1 Department of Evolutionary Anthropology, University of Vienna, Vienna, Austria.; 2 Human Evolution and Archaeological Sciences, University of Vienna, Vienna, Austria.; 3 Department of Archaeology, University of York, York, UK.; 4 AHKR, University of Bergen, Bergen, Norway.; 5 Department of Anthropology, Natural History Museum Vienna, Vienna, Austria.; 6 Institute of Archaeological Sciences, Eötvös Loránd University, Budapest, Hungary.; 7 BIOMICs Research Group, University of the Basque Country UPV/EHU, Vitoria-Gasteiz, Spain.; 8 Department of Human Evolutionary Biology, Harvard University, Cambridge, MA, USA.; 9 Ikerbasque—Basque Foundation of Science, Bilbao, Spain.; 10 Department of Statistics, University of Oxford, Oxford, UK.; 11 Department of Archaeogenetics, Max Planck Institute for Evolutionary Anthropology, Leipzig, Germany.; 12 Department of Genetics, Harvard Medical School, Boston, MA, USA.; 13 Howard Hughes Medical Institute, Boston, MA, USA.; 14 CIAS, Department of Life Sciences, University of Coimbra, Coimbra, Portugal.; 15 Museo delle Civiltà, Italian Ministry for Culture, Rome, Italy.; 16 Collection of Prehistory and Historical Archaeology, State Collections of Lower Austria, Asparn an der Zaya, Austria.; 17 Institute of Archaeology, Slovak Academy of Sciences, Nitra, Slovakia.; 18 Department of Anthropology, Slovak National Museum—Natural History Museum, Bratislava, Slovak Republic.; 19 Research Institute of the University of Bucharest, University of Bucharest, Bucharest, Romania.; 20 National Museum of Serbia, Belgrade, Serbia.; 21 Faculty of Medicine, Center of Bone Biology, University of Belgrade, Belgrade, Serbia.; 22 Vinkovci Municipal Museum, Vinkovci, Croatia.; 23 Anthropological Center, Croatian Academy of Sciences and Arts, Zagreb, Croatia.; 24 Centre for Applied Bioanthropology, Institute for Anthropological Research, Zagreb, Croatia.; 25 Déri Museum, Debrecen, Hungary.; 26 Teleorman County Museum, Alexandria, Romania.; 27 Department for Prehistory and Migration Period, Budapest History Museum, Budapest, Hungary.; 28 Hungarian National Museum, Budapest, Hungary.; 29 Eger, Hungary.; 30 Department of Biological Anthropology, Institute of Biology, Faculty of Science, Eötvös Loránd University, Budapest, Hungary.; 31 HUN-REN Research Centre for the Humanities, Institute of Archaeology, Budapest, Hungary.; 32 Ex Voto Régészeti Kft, Budapest, Hungary.; 33 Salisbury Kft, Budapest, Hungary.; 34 Székely National Museum, Sfântu Gheorghe, Romania.; 35 Dornyay Béla Museum, Salgótarján, Hungary.; 36 Herman Ottó Museum, Miskolc, Hungary.; 37 Department of Biological Anthropology, Faculty of Science and Informatics, University of Szeged, Szeged, Hungary.; 38 Ferenczy Museum Center, Szentendre, Hungary.; 39 Town Museum of Sombor, Sombor, Serbia.; 40 Museum Unit of Public Library ‘Branko Radičević’, Odžaci, Serbia.; 41 Göcseji Museum, Zalaegerszeg, Hungary.; 42 Municipal Museum of Subotica, Subotica, Serbia.; 43 Anthropos Institute, Moravian Museum, Brno, Czechia.; 44 Broad Institute of MIT and Harvard, Cambridge, MA, USA.; 45 Department of Antiquities and Museum, Ras al Khaimah, United Arab Emirates.; 46 Department of Environmental Biology, Sapienza University of Rome, Rome, Italy.; 47 Department of Anthropology, New York University, New York, NY, USA.; 48 Department of Archaeology, Faculty of Arts and Social Sciences, University of Szeged, Szeged, Hungary.; 49 Centre for Human Genetics, University of Oxford, Oxford, UK.

## Abstract

The Linearbandkeramik (LBK) Neolithic communities were the first to spread farming across large parts of Europe. We report genome-wide data for 250 individuals: 178 individuals from whole-cemetery surveys of the Alföld Linearbankeramik Culture (ALPC) eastern LBK site of Polgár-Ferenci-hát, the western LBK site of Nitra Horné Krškany, and the western LBK settlement and massacre site of Schletz, as well as 48 LBK from 16 other sites and 24 earlier Körös and Starčevo from 17 more sites. Here we show a systematically higher percentage of western hunter-gatherer (WHG) ancestry in eastern than western LBK sites, showing these two distinct LBK groups had different genetic trajectories. We find evidence for patrilocality, with more structure across sites on the male than female lines and a higher rate of within-site relatives for males. At Schletz we find almost no relatives, showing that the massacred individuals were from a large population, not a small community.

## Introduction

The archaeological roots of the Linear Pottery culture (Linearbandkeramik, LBK) ca. 5500-5000 BCE are conventionally traced to the Starčevo culture of central Transdanubia ^1-3^, as well as the Körös culture of the Great Hungarian Plain (Alföld) ^4^. The LBK is often divided into two subgroups: the 'eastern LBK' Alföld Linearbankeramik Culture (ALPC) on the Great Hungarian Plain and the much more geographically expansive 'western LBK'. The western LBK has been reconstructed to have spread in two waves, first from Transdanubia, at ca. 5500 BCE, to Slovakia, Austria, Moravia, Bohemia, and central and eastern Germany. Several centuries later, a second wave reached the Paris basin and adjacent areas of France as far west as Normandy and as far east as Poland, Ukraine, Moldova, and Romania ^1,2^.

LBK material culture appears strikingly uniform, given its geographic extent, with the typical LBK settlement pattern consisting of clusters of sites along the alluvial plains of rivers. Nevertheless, archaeological studies^5^ have documented subtle but significant differences in subsistence, settlement patterns, health, and lifeways among LBK communities^6^. The LBK culture is no longer recognized after around 5000-4900 BCE. Studies of the temporal distributions of radiocarbon dates suggest a demographic collapse in that century^7^, potentially linked with violence exemplified at the Late LBK massacres sites of Vrable in Slovakia^8^, Talheimin Southern Germany, and Asparn-Schletz ^9,10^ in Lower Austria.

Studies of variation in strontium (Sr) isotope ratios across individuals have provided insight into mobility patterns in the LBK, notably at Nitra Horné Krškany, Schwetzingen, and Vedrovice. These analyses revealed higher variability in Sr ratios in females than males^5^, showing that women originated from outside the communities where they were buried more often than men, implying different patterns of mobility between the two sexes and providing evidence of patrilocal practices. Further evidence for patrilocality came from a study showing that males buried with polished stone adzes, likely indicative of high social status, had less strontium variation than males without them, suggesting that the former tended to be born and live in their natal communities ^11^. In the archaeological context of settlement patterns, these results suggest that LBK society may have been organised into patrilocal kin-like groups ^12^, with land inherited through the male line. Most LBK sites are located on loess soils, and subsequently, movements of individuals within loess regions are not easily detectable based on strontium isotope ratios. In contrast, paleogenomic methods have the potential to reveal differences between male and female behaviours regardless of local geology. A caveat is that cross-cultural studies of where people live after marriage have shown that women tending to be buried away from their natal homes does not necessarily mean patrilocality; the observed patterns could also reflect more complex kinship systems including ones where couples tended to reside in either their paternal or maternal line family homes^13^.

Analyses of whole genome data from 157 LBK individuals published before this study showed that they inherited their predominant ancestry from Early European farmers (EEF) who then mixed with local European Mesolithic populations, resulting in admixed groups with typically 5% Western Hunter-Gatherer (WHG) ancestry ^14-19^, with a possible differential contribution of Starčevo to LBK and Körös to the ALPC ^20^. However, some LBK individuals have a much higher percentage of WHG ancestry (e.g. an individual at the LBK site of Brunn, Austria) ^18^, suggesting a more complex admixture process ^16^. The only published cemetery-scale studies of LBK substructure focused on the western LBK sites of Derenburg-Meerenstieg II and Stuttgart-Mühlhausen in Germany, both with homogenous ancestry ^16,21^. As the LBK also practised settlement burials, this leaves open the question as to whether cemeteries only represent a selected portion of the population.

A centerpiece of this study is a large sample-size analysis of intra- and inter-site variation in the LBK at three locations with different archaeological characteristics: 1) the ALPC settlement site of Polgár-Ferenci-hát (5500-5100 BCE) in eastern Hungary in which individuals were buried between houses rather than in a cemetery, 2) the cemetery of Nitra, western Slovakia, dated to the LBK expansion phase, 5200–5000 BCE, and 3) the enclosed settlement and massacre LBK site of Asparn-Schletz in Lower Austria dated to the final phase of the LBK at around 5000 BCE. We co-analyzed the newly generated genomic data for individuals from these sites together with new genomic data from 31 other archaeological sites and previously published data to address the following: 1) the extent of genetic differentiation between the LBK and ALPC; 2) kinship patterns of LBK communities and the extent of their correlation to variations in burial location, strontium isotopic values and grave goods; 3) correlations between kinship and differences in diet and mobility (which have previously been hypothesised to be related to LBK social structure); and 4) the genetic structure of the individuals of the settlement and massacred at Asparn-Schletz.

**Figure 1:**
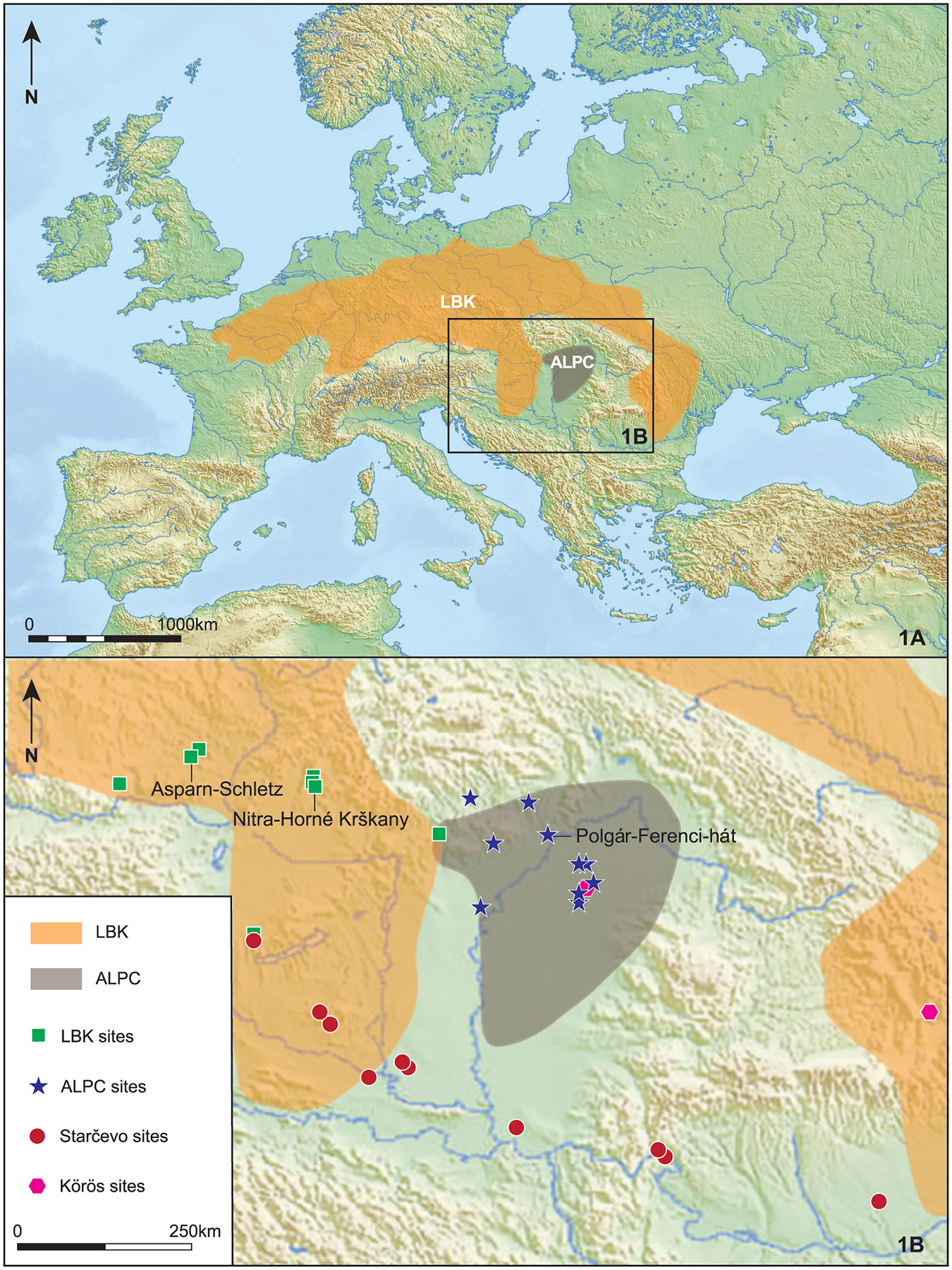
The LBK and ALPC extension: A) Map of the extent of the LBK and ALPC cultures in Central Europe. Generated with Illustrator. The extent of the LBK and ALPC cultures was obtained from Gronenborn and Horejs 2023 ^62^. B) Location of the studied sites, the symbols depict the cultural attribution.

**Figure 2:**
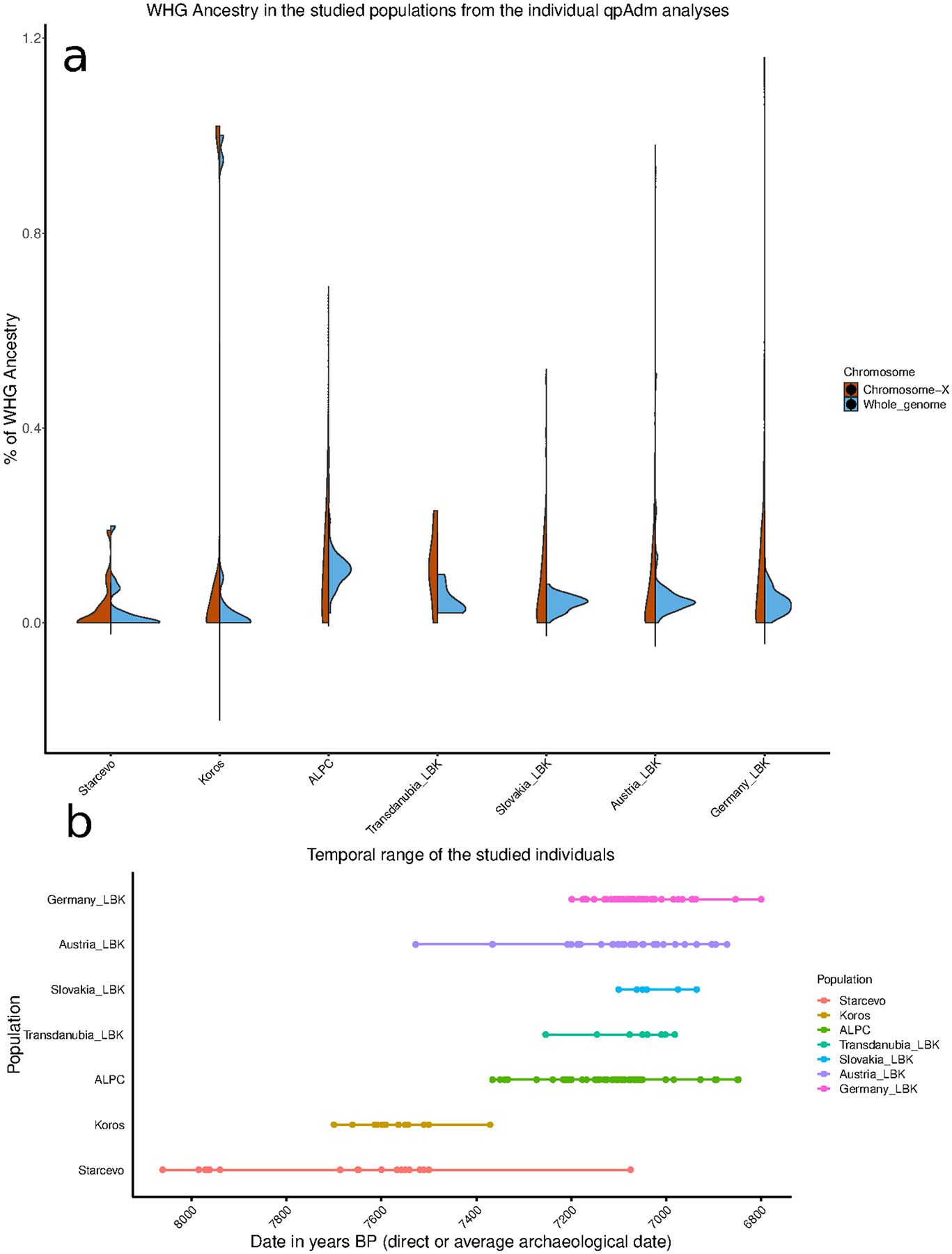
The genomic ancestry diversity in the LBK/ALPC **A)** Histograms of point estimates of ancestry proportions of LBK and ALPC individuals for both the autosomes and X-chromosome (generated with ggplot2 ^63^). **B)** Range of dates and culture span of the individuals included in the study

We generated genome-wide data passing standard metrics for authentication for 250 newly-reported individuals of the Starčevo, Körös, and LBK/ALPC cultures from Austria, Slovakia, Croatia, Romania, Serbia, and Hungary, using target enrichment for 1.24 million single nucleotide polymorphisms (SNPs), and reported improved quality data from an additional 7 individuals, generating a total of 282 new sequencing libraries (Figure 1, 2B, Supplementary Tables 1-2). The new data include 18 Starčevo, 6 Körös (pre-LBK), 80 Hungarian ALPC (henceforth "Hungary_ALPC"), 2 Transdanubian LBK ("Transdanubia_LBK"), 87 Austrian LBK ("Austria_LBK") and 57 Slovakian LBK ("Slovakia_LBK") from a total of 31 archaeological sites (Figure 1, 2B). Individuals with fewer than 30,000 SNPs covering the autosomal targets were not included in ancestry analyses, but their data are reported. In addition, we did not use data from 1st-degree relatives of higher coverage individuals in the data set for ancestry analyses. We co-analyzed these individuals with published data for 171 Starčevo, Körös, ALPC, and LBK individuals ^15,16,18-23^. We also generated 19 new radiocarbon dates and built Bayesian date models for four sites of the ALPC and LBK (Supplementary Section 2, Supplementary Figures 18-21).

### Elevated WHG ancestry in the eastern LBK

**Extended Figure 1:**
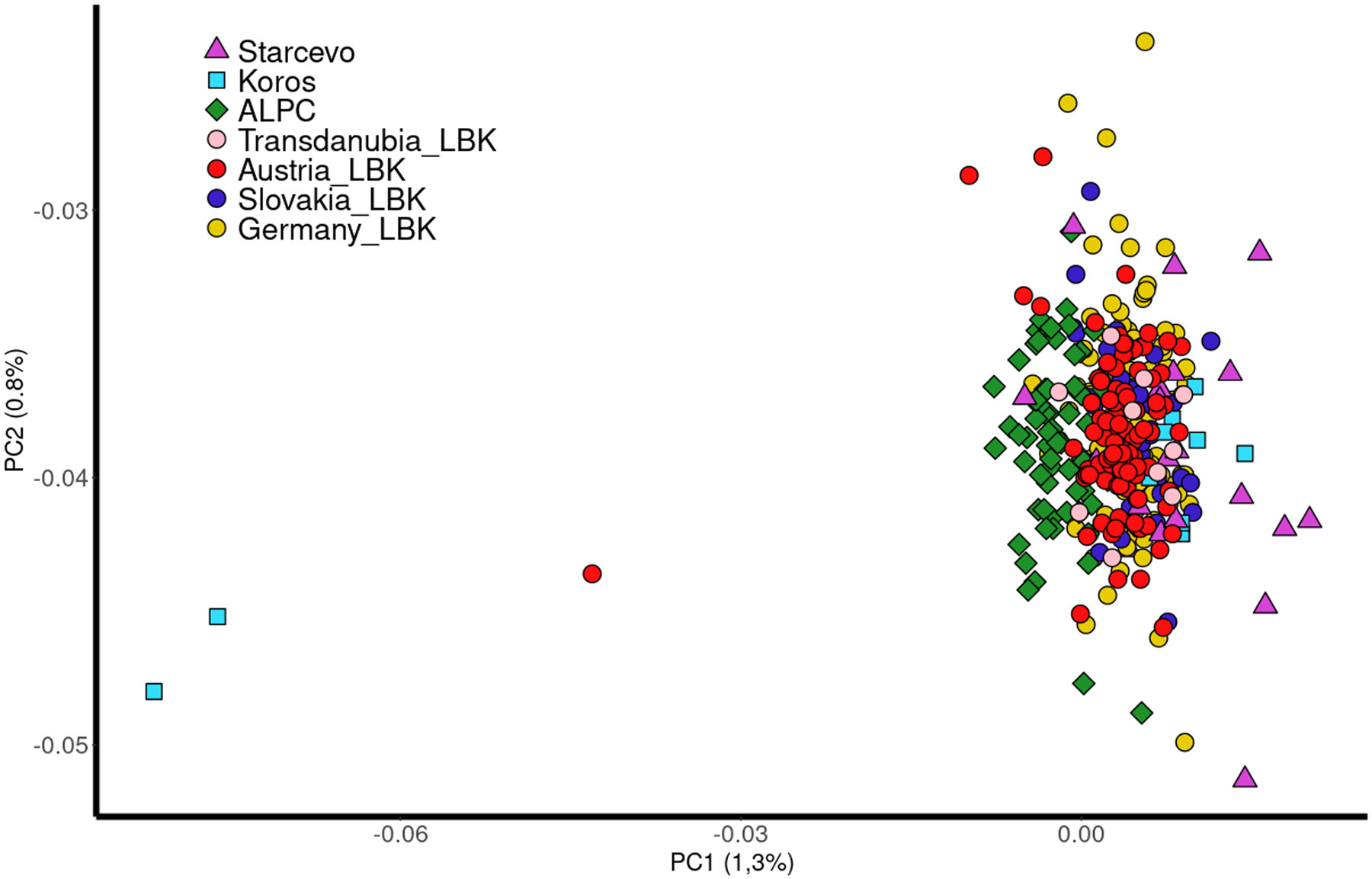
Principal Component Analysis (PCA): PCA performed with 879 modern Eurasian individuals in which the ancient individuals were projected. The modern individuals have been removed from the image. The PCA shows the clustering of the LBK and the position of individuals along the X axis, indicating differential WHG affinities and showing that WHG (represented by two Körös culture outliers with entirely WHG ancestry) are more closely related to ALPC. Three individuals: I6914 (Austria_LBK) and I1507, I497 (Köros) are outliers.

We used *smartpca* ^24^ to perform a Principal Components Analysis (PCA) (Extended Figure 1, Supplementary Figure 22) on genome-wide data from present-day European populations genotyped on the Affymetrix Human Origins SNP array and then projected the ancient individuals. The PCA shows that the individuals from the ALPC sites are located closer to the WHG-like individuals in the PCA. The Körös and Starčevo individuals cluster with the western LBK, suggesting that the analysed ALPC individuals might be the result of a mixture between an early Neolithic population and additional WHG.

**Extended Figure 2:**
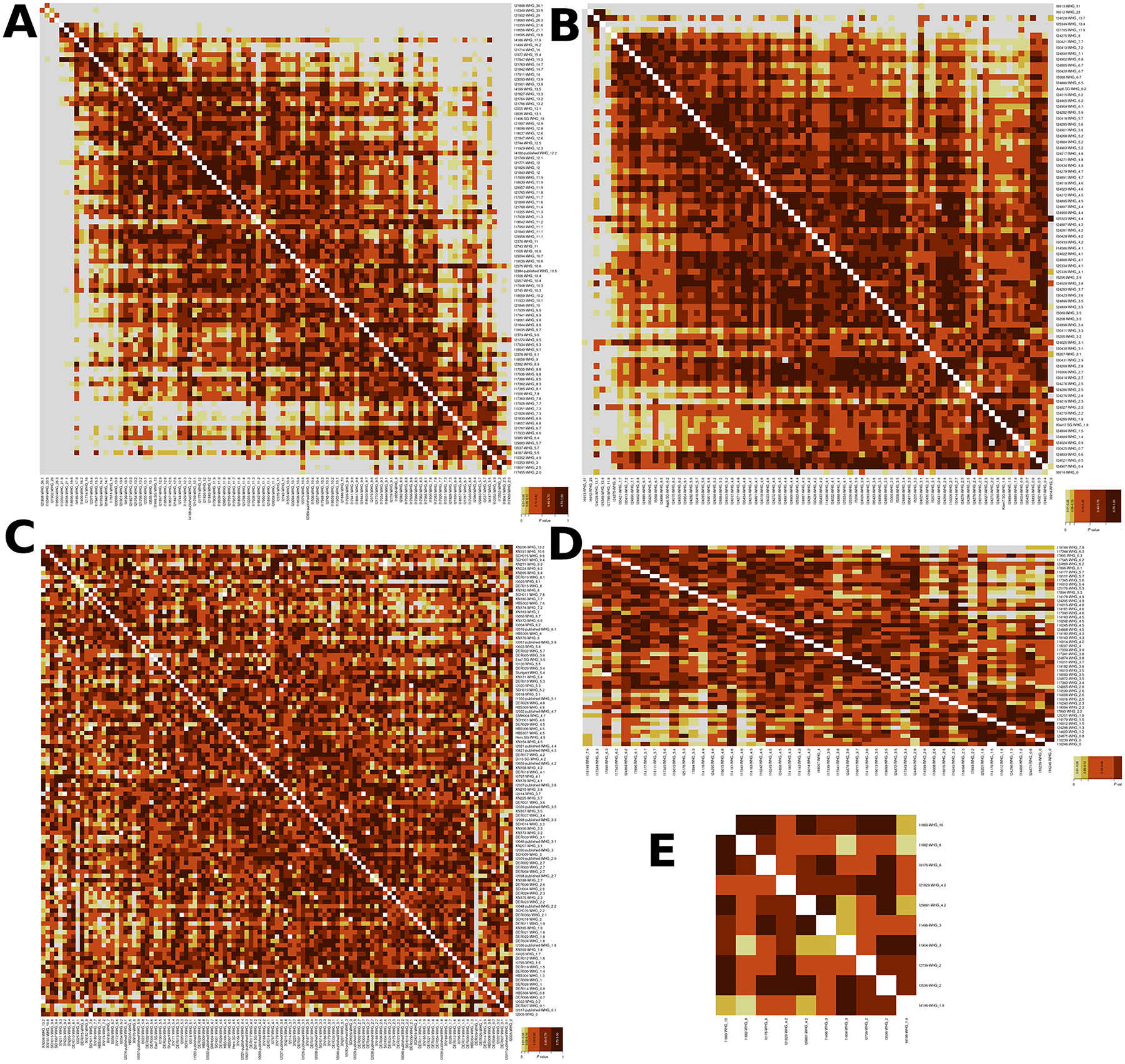
qpWave plots: qpWave plots to test for individual differentiation, with each population represented in one plot. Grey colour means results were highly significant (little genetically related). The number after the name of each individual relates is the point estimate of WHG ancestry from qpAdm. A) ALPC individuals. Individuals I21898, I10349, I21902, I18660, I10350, I18656, I18695, I4186, I1499, I21714, and I2377 are labelled in our analysis as ALPC outliers with high WHG ancestry. Individuals: I21828, I21830, I10351, I10352, I10353, I18657, I21767, 17933, I1500, I2380, I3537, I17455, I18636, I29883, I18641 and I4187 are labelled in our analysis as ALPC outliers with low WHG ancestry. B) Austria LBK Individuals: Individuals I27785, I25349, I6913, I6912 and I24028 are labelled in our analysis as outliers with high WHG ancestry. C) Germany LBK Individuals, D) Slovakia LBK Individuals: Individual I18144 is labelled in our analysis as an outlier with high WHG ancestry. E) Transdanubia_Hungary LBK Individuals: individuals I1882 and I1883 are labelled in our analysis as outliers with high WHG ancestry. We used qpWave from admixtools to perform the plots, each square represents the two-sided p-value of every single test.

We grouped individuals based on archaeological culture and geography (proxied by present-day country): Austria_LBK, Slovakia_LBK, Transdanubia_LBK, Hungary_ALPC, and Germany_LBK. We estimated ancestry proportions with *qpAdm*, using as proxies for the sources a pool of Balkan early farmers with little or no WHG admixture (Balkan_N) and a pool of western European hunter-gatherers (WHGA) ^25^. As Right reference outgroups, we used pools of Turkey_N, ancient Africans, and Mesolithic European hunter-gatherers more divergent in time or space (WHGB) (Supplementary Section 5 ). We used *qpWave* to identify significant outliers from the main cultural and geographical groups at *p-value*<0.05, adding the tags HGEXC (“hunter-gatherer excess”) and EEFEXC (“Early European Farmer excess") (Supplementary Table 1, Extended Figure 2). Eastern LBK Hungary_ALPC individuals have, on average, 11±0.3% WHG ancestry (*P*=0.77 for fit) (Figure 2A). In contrast, Slovakia_LBK and Austria_LBK individuals have an average of 4.5±0.4% WHG (*P*=0.01 and 0.09 for fit). Ten western LBK individuals from Transdanubia (Transdanubia_LBK), have an estimated 3% WHGA ancestry, although the qpAdm model is not a statistical fit (*p*<0.001) so this measurement should be viewed with caution (Supplementary Table 3 and Supplementary Section 5).

### No evidence for sex biased population mixture

We used DATES ^26^ to estimate the age of admixture in WHG and Early European Farmers. Consistent with previous findings, but now with higher resolution^22^, we infer that the mixture occurred on average ~400 years before the sampled Austrian_LBK, Slovakia_LBK, and Germany_LBK lived (range of 95% CI: 6,010-5,460 BCE) and 530 years before the ALPC individuals (range of 95% CI: 5,875-5,796 BCE), assuming an average date of 5,300 years of ALPC and 5,100 for the LBK (Supplementary Table 4). This suggests a scenario in which the dawn of the archaeologically defined LBK culture was marked by the completion of a period of mixture, reflecting a social incorporation of WHG communities, which plausibly could have been part of the process by which the LBK distinguished itself from preceding cultures.

Some degree of mixture with WHG continued into the LBK period itself, as documented by individuals at the early LBK site of Brunn (Austria) with evidence of admixture in the last couple of generations before they lived ^18^ (Supplementary Table 1). We found further evidence for this using the RFMix ^27^ method, where we inferred the locations and size of segments of WHG ancestry in each LBK individual after filling in missing genotypes and phasing the data using the imputation engine GLIMPSE ^28^ (Supplementary Figure 23, Supplementary Table 5, Supplementary Section 5). We correlated the summed length of inferred WHG segments from RFMix greater than 0.2 cM to *qpAdm* estimates of WHG ancestry and observed a high Pearson correlation coefficient of 0.85, suggesting that these inferred segments often reflect true WHG admixture (Supplementary Figure 24), although there are inevitably errors in this inference and we do not have a well-calibrated understanding of their rate or genomic distribution. We identified long putative WHG segments in some ALPC individuals (up to 55 cM, individual I21902 from Polgár-Ferenci-hát, 5371-5216 cal BCE), which if true suggest mixture in the last few generations in their history, similar to the pattern at Brunn. At the ALPC site of Polgár-Ferenci-hát, with its elevated rate of WHG ancestry, we also detected significant within-community variation in WHG ancestry. In one genetic group, henceforward referred to as “Family B” (Supplementary Figure 28), three individuals from this cluster (I21898, I21902, I18660), father, son and daughter, respectively, had significantly elevated WHG: (36%, 26% and 29%, respectively). The daughter, who we estimate to have been 27-28 years old at the time of her death, was buried with many grave goods which were otherwise uncommon at this settlement (Supplementary Figure 29). These individuals are related to a 3-4th-degree to two others (I21827 and I18695), father and daughter. The daughter of this second group had significantly elevated WHG ancestry (20%)(Supplementary Figure 28, Supplementary Table 1), while the father's ancestry was typical for the majority of individuals from this site (13% WHG). In the first group, the mother is unsampled, but we assessed her WHG ancestry to be ~9% lower than the father's (which explains the offspring’s intermediate WHG proportions). In contrast and by a similar calculation, in the second group, we estimate that the unsampled mother had ~7% higher WHG ancestry than the father. Thus, WHG admixture patterns appear to vary by family.

We tested directly for sex bias in WHG admixture patterns by comparing qpAdm estimates of ancestry on the X-chromosome, with 2/3 female ancestry, and the autosomes, without sex bias. The estimates are statistically indistinguishable in all tests (Supplementary Table 3) (Figure 2A), providing no evidence for either primarily male WHG contribution to early farmers ^29^, or hunter-gatherer Mesolithic women preferring farmers due to perceived higher status ^30^. A caveat is that these null results may reflect limited precision in X chromosome qpAdm estimates.

### Differential mating and social strategies in the LBK/ALPC

**Extended Figure 3:**
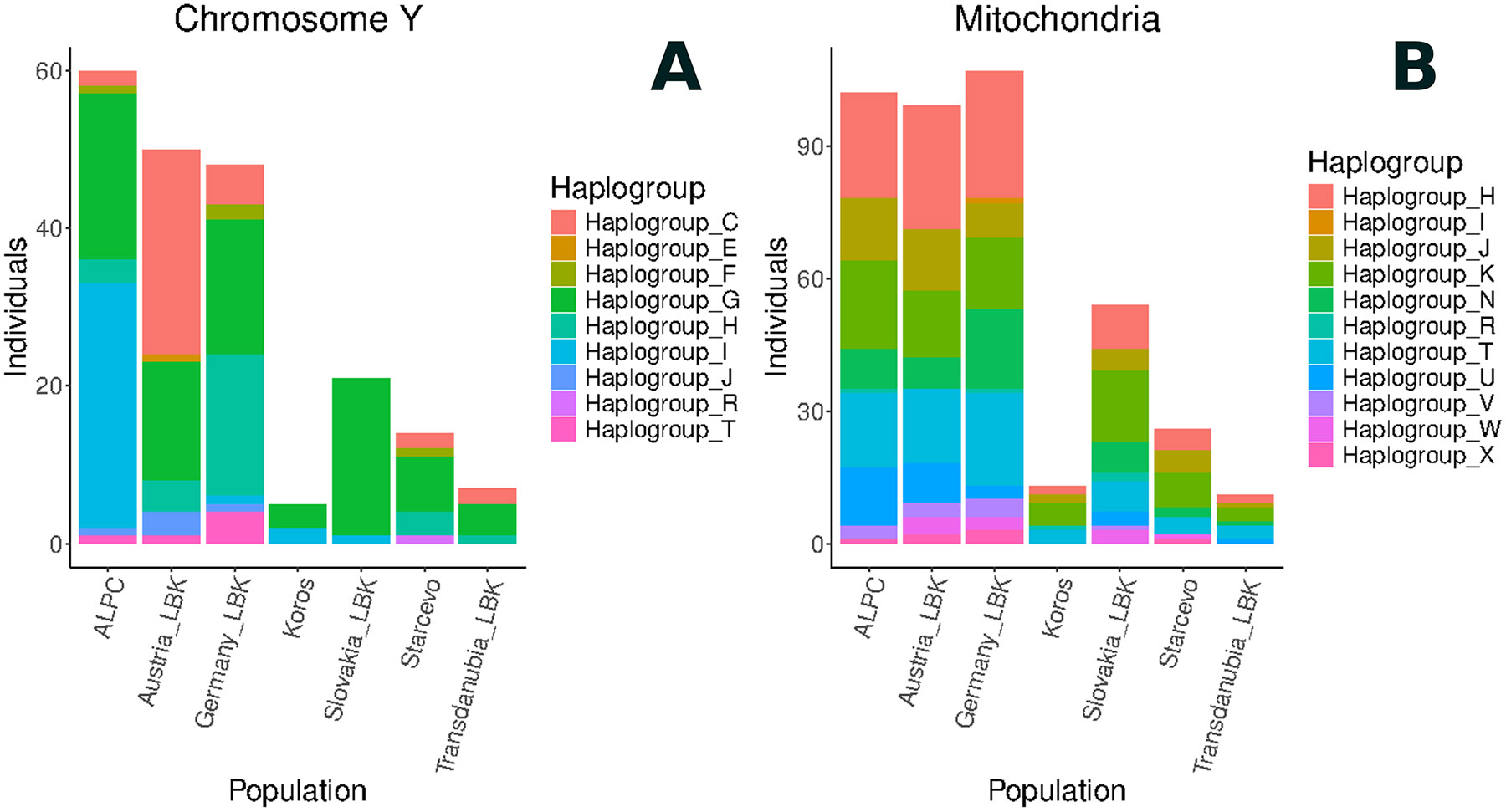
Parental haplogroups: Distribution of the Y chromosome and mtDNA haplogroups per population. The Y-axis represents the number of individuals.

The large sample size of LBK individuals analysed in this study allows us to perform a continental-scale comparison of patterns of variation on the Y chromosome reflecting the history of the entire male line, and mitochondrial DNA, reflecting the history of the entire female line. In the Y-chromosome analysis, we detect previously unappreciated geographic variation across the LBK (Extended Figure 3) (a χ^2^_205,42_=183 test for heterogeneity is highly significant at *P*<10^−19^), with haplogroup G dominant in the Slovakian, German, and Hungarian LBK; haplogroup C in the Austrian_LBK; and the majority of the Hungary_ALPC individuals with haplogroup I (36%), associated with Mesolithic populations such as those of the Iron Gates regions of Serbia and Romania ^20,31^. We present the list of mutations supporting each assignment in Supplementary Table 6. In contrast, we do not detect significant structure in mitochondrial DNA haplogroup frequencies, with no haplotype with a frequency greater than 30%, and no evidence for haplotype frequency differences across the regional groups (Extended Figure 3, Supplementary Table 1), (χ^2^_420,54_ = 58.8, *P* = 0.30). These results provide evidence of limited gene flow among LBK communities on the male line, and one possible reason for this is a much higher rate of movement of females between communities. Previous studies already suggested homogeneity in Y-chromosomes in the 6th Millennium BCE^32^. Here, we provide evidence that these differences are regionally variable, which could be explained by the limited movements of males. However, we do not have sufficient sampling to make any general claim about patrilocality practices in the ALPC ^13^.

**Figure 3:**
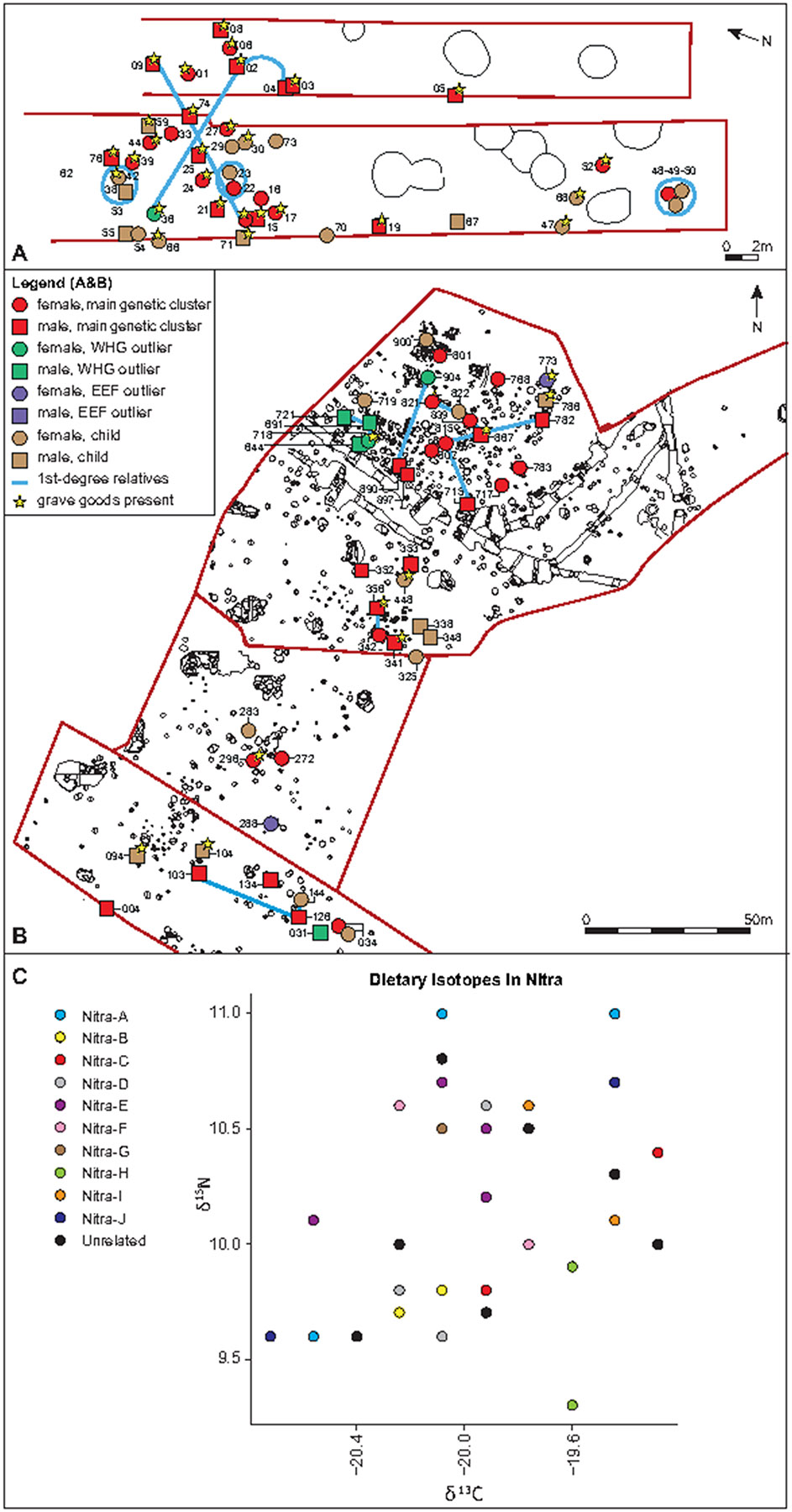
Kinship patterns in LBK sites: Burial layouts for A) Nitra Horné Krškany (top) and B) Polgár-Ferenci-hát (bottom). Each symbol represents one individual: squares males, circles females. Red denotes the main genetic cluster, green WHG outliers, and violet EEF outliers. Light brown are children. Blue lines or circles are 1^st^-degree relatives and the yellow pottery symbol grave goods in burials. Only individuals with ancestry information are plotted C) Dietary isotopes at Nitra Horné Krškany coded by families. Families at Nitra Horné Krškany do not cluster in dietary-specific groups. All plots are restricted to individuals with qpAdm estimates.

By studying the distributions of close relatives in the two burial locations where we detect many relationships (Figure 3A-B), we find genetic evidence for patrilocality in Polgár-Fernci-hát but not Nitra Horné Krškany. At Nitra Horné Krškany, we detect ten families, including a pedigree spanning four generations, and at Polgár-Ferenci-hát, we detect four families, including one with 12 individuals. Combining the two cemeteries, we find that relatives up to the 3^rd^ degree (Supplementary Table 7, Supplementary Section 6) tend to be buried together more often than random pairs of individuals. At Nitra Horné Krškany, we did not detect significant differences in the number of relatives between males and females χ^**2**^_47,1_=0.14, *P*=0.70. In contrast, we detect strong evidence of patrilocality at Polgár-Ferenci-hát, with more relatives for males (21 of 22) than for females (14 of 23): χ^**2**^_45,1_=7.78, *P=*0.005 (Table 1). We also identified that all the individuals from Rákóczifalva–Bagi-földek Site-8/A are from a single family group (Supplementary Section 6, Supplementary Table 7).

### No kinship-associated differences in mobility and diet

**Extended Figure 4:**
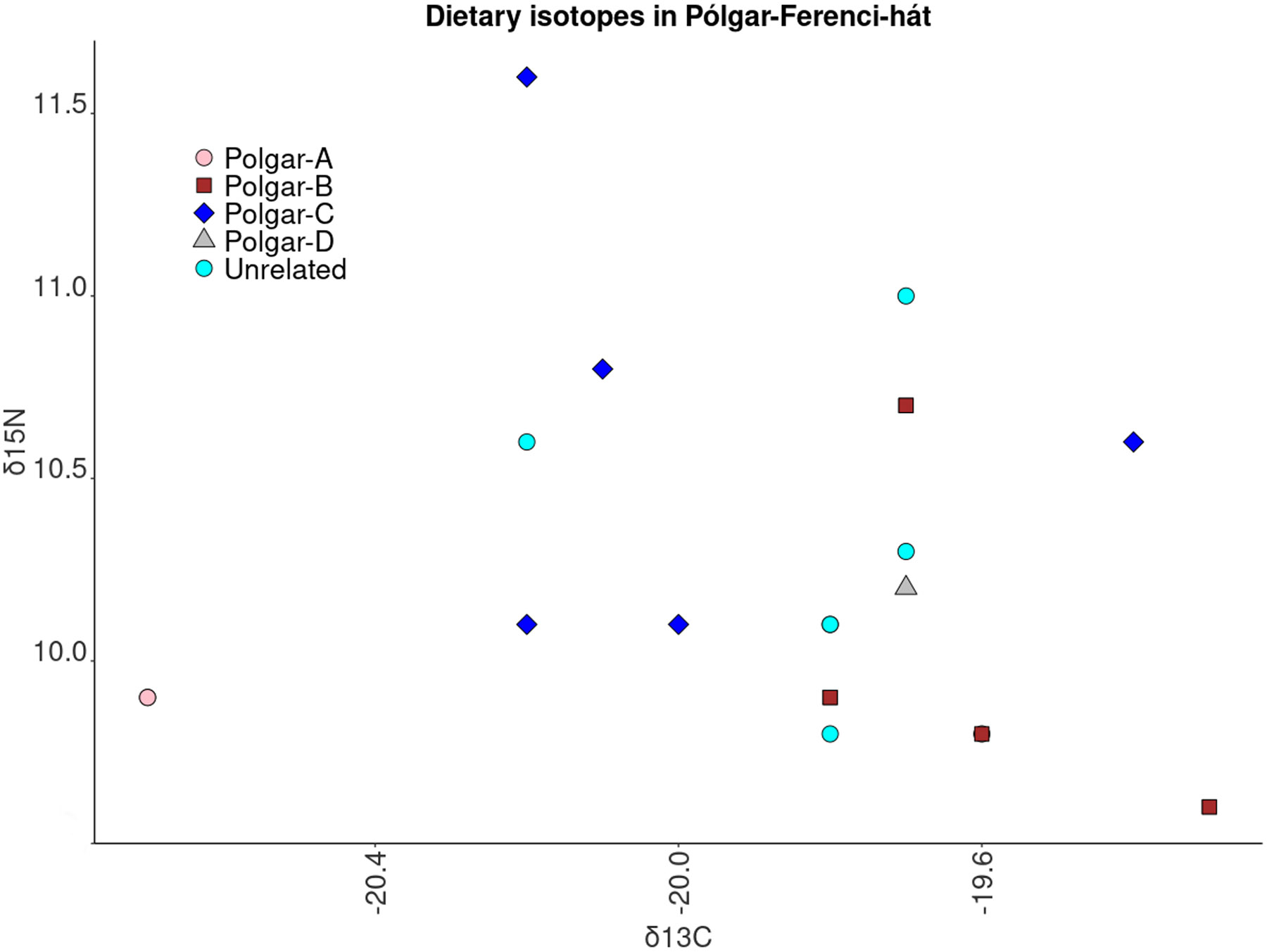
Isotopic data: Isotope data from Pólgar-Ferenci-hát. Here we plot the ratio δ^13^C/δ^15^N. Each dot represents one individual and the colour denotes the family.

We analysed the findings of genetic relatedness together with dietary (carbon, δ^13^C, and nitrogen, δ^15^N) and strontium isotope data (^87^Sr/^86^Sr) ^33^ (Supplementary Section 3, Figure 3C, Extended Figure 4, Supplementary Table 8). We did not perform similar analyses for Asparn-Schletz as we had dietary isotopic data for too few individuals and too few detected genetic relatives.

We detect significant within-family variation in the measurements of isotope sensitive to mobility both at Nitra Horné Krškany (Levene’s test for variances n=12, *P*=0.01) (Supplementary Table 14) and at Polgár-Ferenci-hát (Levene statistic for the difference in variance = 16.74, *P=*0.001) (Supplementary Table 17). This shows that people at both sites and even the same families varied in the places where they resided over their lifetimes.

We next tested for significant differences across families in their dietary patterns but found no strong signals. The only notable correlation we detect is at Nitra Horné Krškany, where we found a marginally significant signal of variation across families for δ^13^C carbon isotopes (Kruskal-Wallis=17.20, N=26, *P*=0.04) (Supplementary Table 16), providing some evidence that families sourced food from different landscape contexts, either through variation in direct consumption or through variation in consumption of animals eating these plants^27^. However, because we carried out multiple hypothesis tests, the observation of one marginally significant signal of correlation like this should not be interpreted as strong evidence.

We do not detect significant variation in strontium isotope ratios across families at Nitra Horné Krškany (Mann-Whitney U test, n=21, *P*=0.16) (Supplementary Table 15), nor do we detect a correlation between family structure and the presence of grave goods (Supplementary Section 2.1, Supplementary Table 8) (δ13C, Kruskal-Wallis=4.99, *P*=0.17; δ15N, Kruskal-Wallis = 1.45, *P*=0.69) (Supplementary Table 16). At Polgár-Ferenci-hát, we also do not detect variation in isotopic ratios across families: δ13C, Kruskal-Wallis = 4.99, *P*=0.17; δ15N, Kruskal-Wallis = 1.45, *P*=0.69 (Extended Figure 4, Supplementary Table 20). This suggests that diet, mobility and funerary rites were mostly independent of biological kinship ties.

### Variation across the LBK in community size and mate choice

We carried out ancient DNA analysis of all excavated skeletons from Asparn-Schletz, corresponding to 70 individuals from the ditch system associated with a massacre, three from a water well with older dates than the massacred and 20 individuals from settlement burials. A total of 92 of the 93 individuals yielded enough genomic data for genetic analyses (Supplementary Tables 1 and 2). Of the 69 individuals with genome-wide data from the base of the ditch system, including 48 genetic males and 21 genetic females, we detected only a single pair of 1st/2nd-degree relatives and possibly a pair of individuals between ditch and settlement contexts. Only 4 of the 69 analysed individuals from the Asparn-Schletz ditch system are related up to the 3^rd^ degree, contrasting with much higher rates at Nitra Horné Krškany and Polgár-Ferenci-hát (Table 1). We identified a single first-degree relationship between an older male adult (I24892) and a non-adult (I24280) from within the massacre context, providing further evidence that this was not an event that affected only a small community that might have been expected to include more families and hence more close relatives.

**Figure 4:**
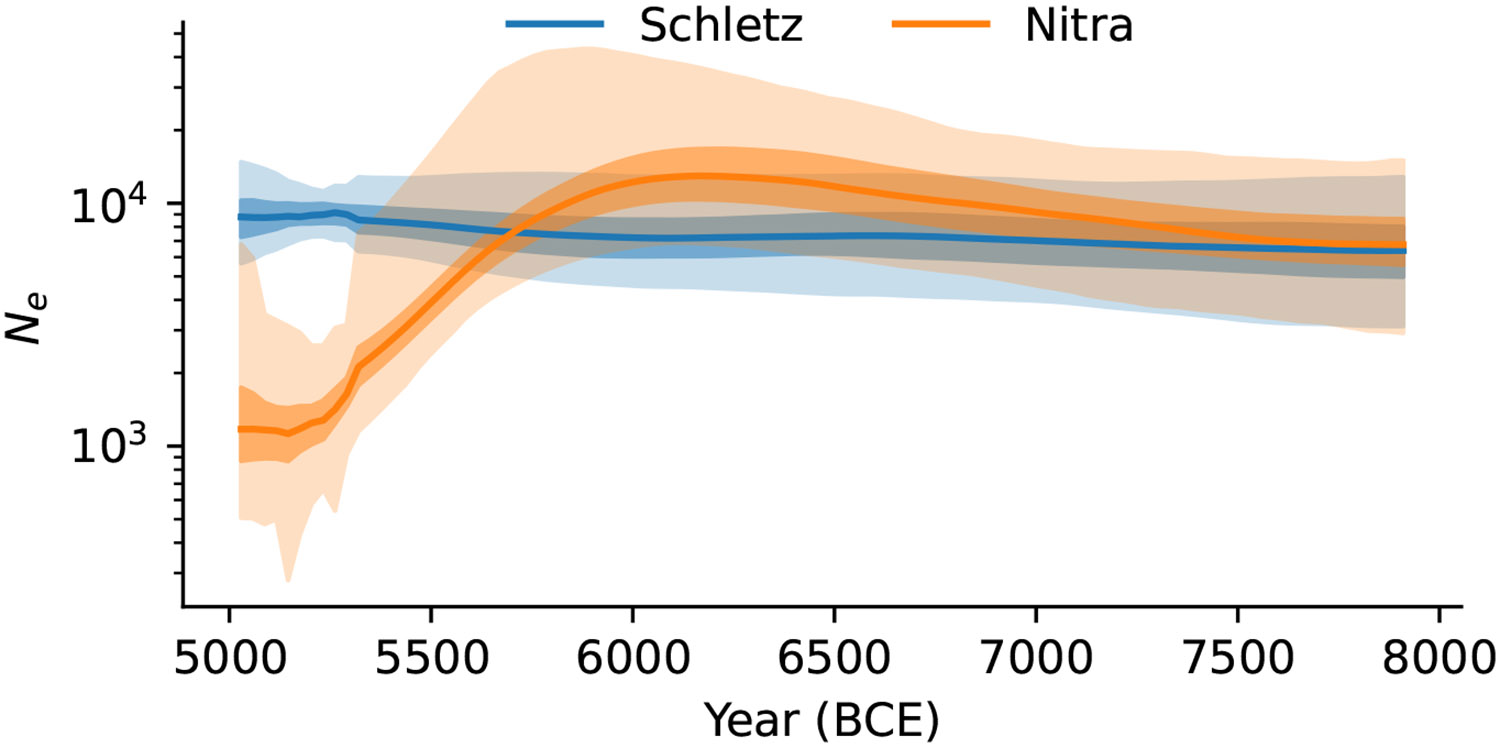
Asparn-Schletz population size: Inferred population size trajectory of Asparn-Schletz and Nitra based on HapNe-LD. The recent contraction in Nitra Horné Krškany likely reflects undetected families in the sample, while the Asparn-Schletz individuals have no evidence of being more closely related to each other than they are to the more widely sampled LBK. Error bars represent one (dark) and two (light) standard deviations.

We used HapNe-LD ^34^ to infer the effective population size trajectory of unrelated individuals from the Asparn-Schletz massacre in the hundreds of years before they lived (n=54). We find no evidence for a contraction in the gene pool in this period, which could be explained if the people massacred at Schletz were drawn from many and not a single community. In contrast, at Nitra Horné Krškany (n=18), we observe the signatures expected for a small community of people isolated from their neighbours (Figure 4, Supplementary Section 7).

**Figure 5:**
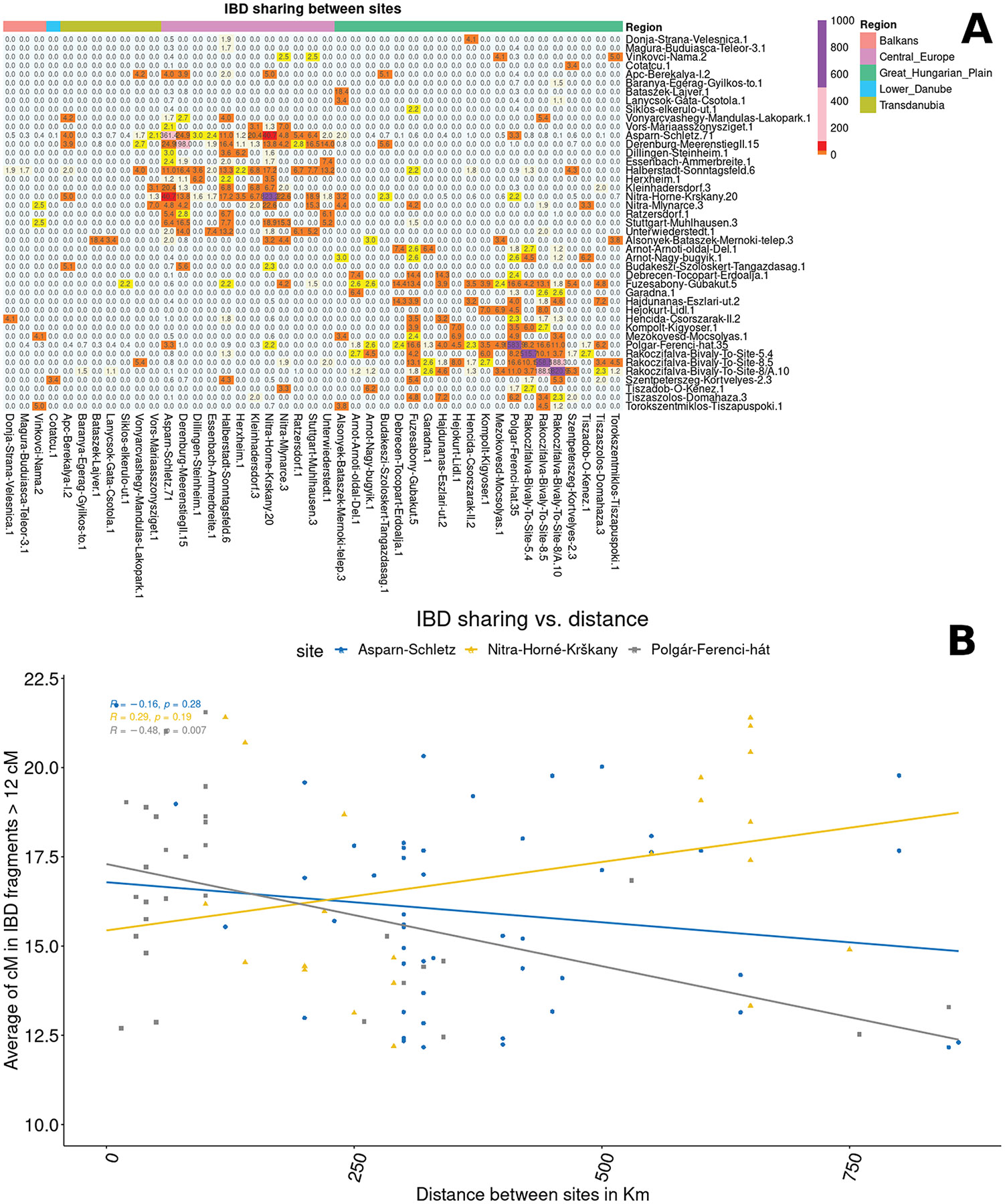
The LBK/ALPC networks: I: A) A heatmap showing the intensity of IBD, presenting the average total length of IBD segments > 12cM shared between all possible pairs by area or period. The numbers after the site names show the number of individuals per site included in these analyses. B) Regression of summed IBD >12cM shared between individuals of each pair of sites (averaged over all pairs), and geographic distance. Polgár-Ferenci-hat has more connections with closer sites supporting a localised ALPC community, while Asparn-Schletz and Nitra Horné Krškany do not show a clear association with distance, as would be expected if the western LBK expansion was so rapid that nearby groups were hardly more closely related than groups far apart.

Further evidence for the Asparn-Schletz individuals being drawn from a much larger population than those at the other sites comes from IBD sharing patterns between the studied individuals (Supplementary Table 9), inferred based on analysis of the imputed and phased dataset. We observe far less average sharing of IBD segments >12 cM among individuals at Asparn-Schletz (26 cM) than at Nitra Horné Krškany (174 cM) or Polgár-Ferenci-hát (158 cM). The reduction is significant (*P*=0.001), even after excluding 1st, 2nd, and 3rd-degree relative pairs (*P*=0.005), which suggests that the signal is driven by distant relatives in sites, not just close relatives (Figure 5).

**Extended Figure 5:**
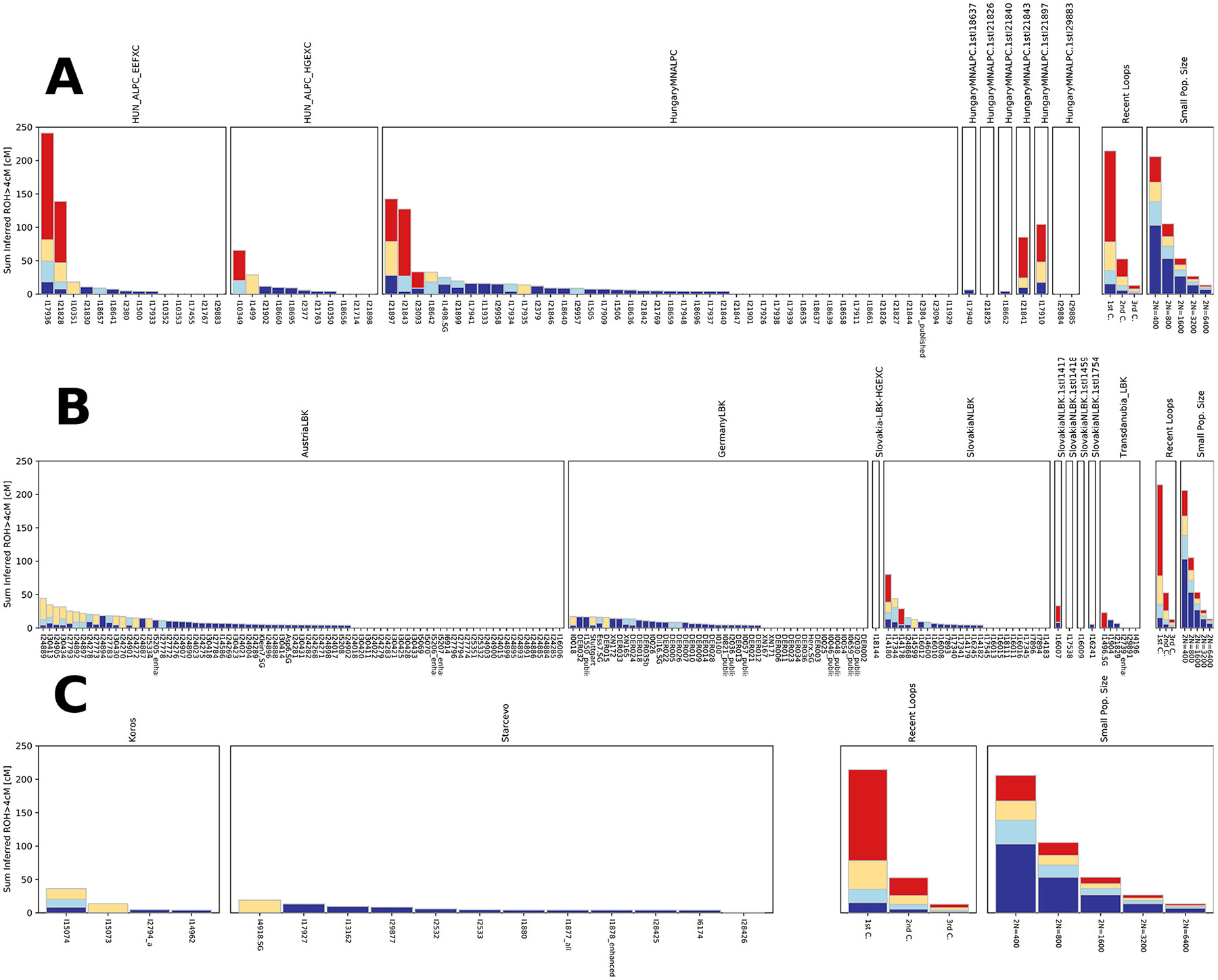
Rund of Homozigosity: ROH distribution in the dataset. A)LBK individuals, B) ALPC individuals, C) Koros and Starcevo individuals. Individuals with more than 400,000 SNPs and the assessed ROH. Individuals in the ALPC group show a higher rate of close-kin unions (as reflected in the presence of ROH segments >20cM) than the rest of the dataset.

Eight individuals from Polgár-Férenci-hát and four from Nitra Horné Krškany have elevated rates of Runs of Homozygosity (ROH), which reflects individuals reproducing within their own genetic lineages^35^. In contrast, the rest of the individuals at these sites, and all from Asparn-Schletz, have no segments with ROH >4 cM ^35^ (Extended Figure 5, Supplementary Table 10).

The IBD analysis gives evidence of two qualitatively distinct regional networks of people linked by distant familial relationships: one for the Great Hungarian Plain, where the across-site rate of sharing averages 45.56 cM, and one for Central-Western Europe, where the across-site rate of sharing averages 9.19 cM, but with a far lower 0.19 cM of sharing across regions (Supplementary Table 10, Figure 5A). This is in accord with archaeological studies that imply that Nitra Horné Krškany and Polgár-Ferenci-hát are associated with different LBK expansions and periods ^22,36,37^. We further observe that the rate of IBD sharing decreases significantly with distance from Polgár-Ferenci-hat (*p=*0.011), which could be explained if there was a localised network of people within the ALPC. In contrast, there is weak or no detectable association of IBD sharing with geographic distance in the western LBK, as would be expected if the western LBK expansion was so rapid that nearby groups were hardly more closely related than groups far apart (Figure 5B). Finally, the observation that Hungary_ALPC individuals have, on average, 16.64 cM in ROH (without 1st-degree relatives) and are the LBK group with the largest fractions of their genome in ROH, suggests that they may have had more restricted mating practices than the more widespread western LBK.

### high-frequency long-range haplotype screens for selection

We scanned the imputed diploid genotype data for the LBK and ALPC individuals for signals of selection by searching for haplotypes that had evidence of being very recent in origin based on their large scale and yet too common to have risen to such high frequency in the absence of selection. Because of the poor haplotype phasing expected for ancient genomes, we carried out these scans not only with the phased but also the unphased versions of the iHS and nSL scores, as implemented in Selscan 2.0 ^38^. We also used BetaScan^39^ to test for loci affected by long-term balancing selection.

**Figure 6:**
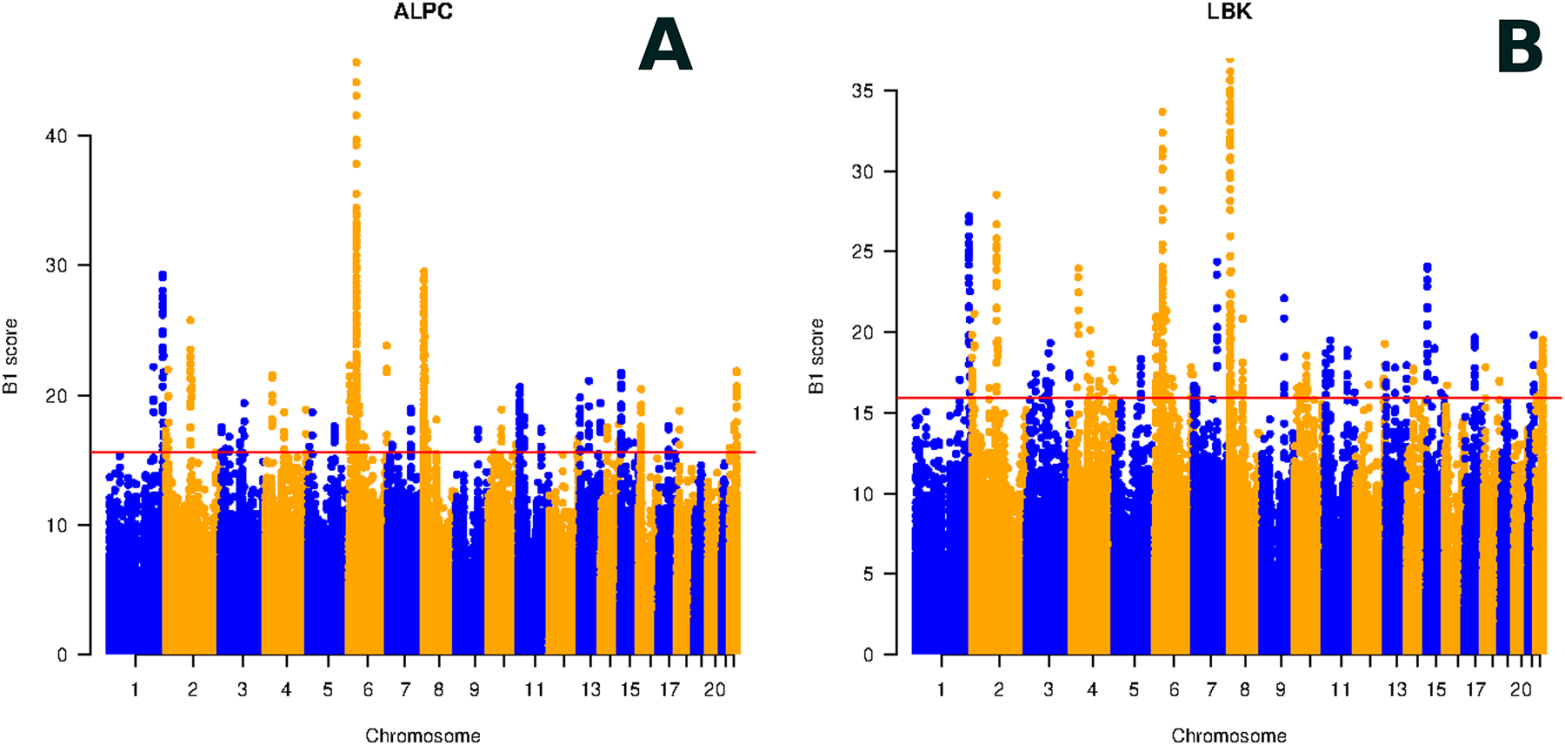
Selection in Neolithic genomes: (A) B1 scores in the ALPC. (B) B1 scores in the LBK. B1 shows regions with balancing selection, the highest signal on chromosome 6 at HLA.

We detected evidence of long-term balancing selection in the HLA region on chromosome 6, with elevated B1 scores (Figure 6), consistent with previous evidence of balancing selection at this locus in Neolithic Europeans ^40^. A second notable finding is 26 genes with evidence of balancing selection in the ALPC and LBK (Supplementary Table 11). Many were also reported as significant outliers based on analysis of patterns of variation in modern Europeans ^41^.

**Extended Figure 6:**
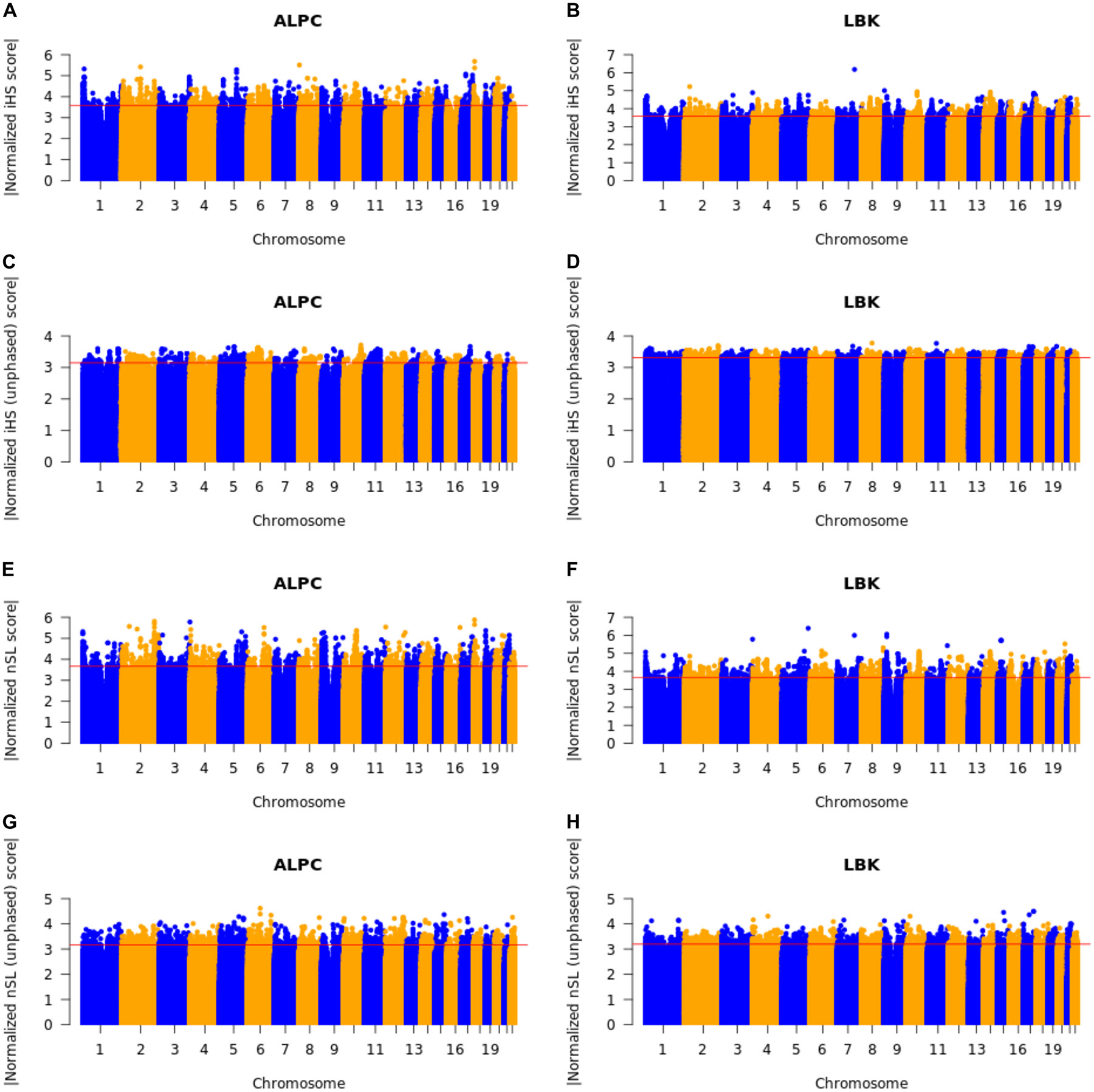
Natural selection in Neolithics: Tests for positive selection in the ALPC and LBK population, made with qqman ^64^. The red lines indicate the top 0.05% cutoff. (A) Normalized iHS scores in ALPC. (B) Normalized iHS scores in LBK. (C) Normalized unphased iHS scores in ALPC. (D) Normalized unphased iHS scores in LBK. (E) Normalized nSL scores in ALPC. (F) Normalized nSL scores in LBK. (G) Normalized unphased nSL scores in ALPC. (H) Normalized unphased nSL scores in LBK.

**Extended Figure 7:**
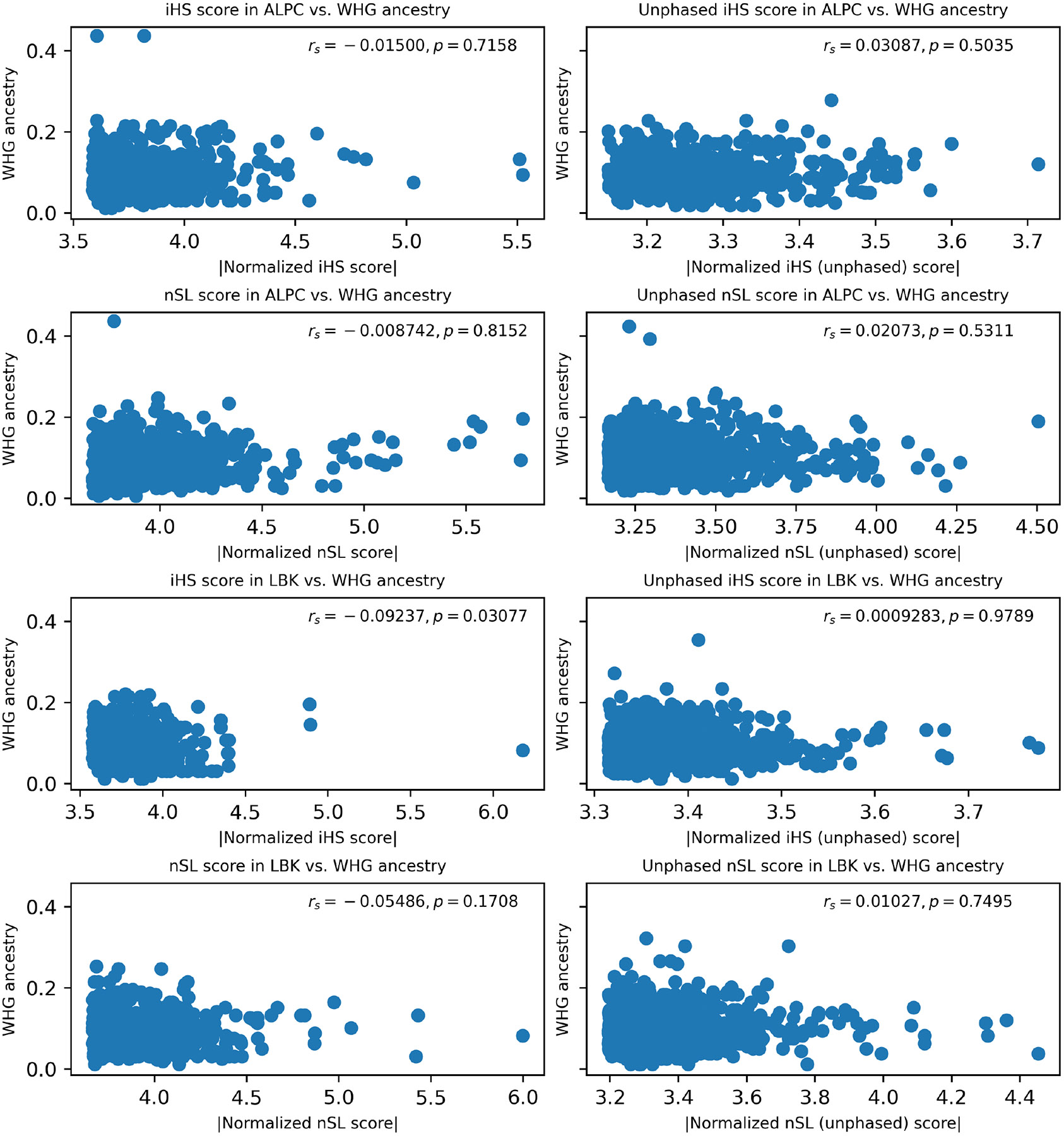
Correspondence between the ancestry in ALPC and LBK segments with the selection scan values. Each dot represents a region of 0.2 cM of the genome, in the Y-axis we display the average WHG ancestry of the region, and in the X-axis the average selection scores from candidate SNPs within the region (Supplementary Table S11). We show the two-sided Spearman correlation coefficients and p-value.

We identified 3 and 37 genes with evidence of positive selection in the ALPC and LBK, respectively (Supplementary Tables 11-12, Supplementary Section 8, Extended Figure 6), including notable examples associated with pigmentation. The *PRKCH* gene encodes the PKCη protein in melanocytes which is involved in the protein kinase C-dependent pathway regulating melanogenesis ^42^. The *PTPRN2* gene had a higher level of expression in lightly pigmented melanocytes than in darkly pigmented melanocytes, similar to *SLC45A2* which contains one of the strongest known signals of pigmentation selection in Europe ^43^. When we correlate the WHG local ancestry components with our selection signals, there is nominal evidence that non-WHG ancestry is more enriched at sites under selection (Supplementary Section 8, Extended Figure 7), although we have not ruled out the possibility that this could potentially be an artifact of greater sensitivity to selection signals at non-WHG regions.

## Discussion

Our study reveals differences in kinship structure, admixture, demography, and ancestry across the LBK. We report an average of around 11% WHG ancestry in the ALPC, a proportion that has reached as high as 35% in some individuals. This contrasts with the much lower average among the studied individuals from Austria (an average of 4,5% with a range of up to 14%) and Slovakia (an average of 4% with a range of up to 8%). This suggests that the admixture between farmers and hunters of the Great Hungarian Plain was more extensive than among the more westerly LBK communities. This admixture shows no sex-biased trend despite the high fraction of Y-chromosome haplogroups associated with WHG.

Correlation between isotopic and genetic shows no statistical relationships between diet and mobility patterns between families in Nitra Horné Krškany and Polgár-Ferenci-hát, but we find evidence for high variation in mobility within families, at least at Nitra Horné Krškany. We observe no evidence of a correlation between genetic patterns and archaeological markers of social status. We can, therefore, make no claims regarding population substructure driven by social status in the LBK.

We find that at both Nitra Horné Krškany and Polgár-Ferenci-hát, relatives were buried closer to each other than non-relatives. Polgár-Ferenci-hát males had significantly more relatives than females in the cemetery population we sampled. This pattern, combined with the evidence of limited regional diversity in the Y-chromosome and long IBD tracts, is consistent with limited mobility within the Great Hungarian Plain and patrilocal practices. We observed much less IBD across western LBK sites and approximately contemporary ALPC sites than within either community, suggesting they were part of different mating networks. We do observe IBD between sites of the Great Hungarian Plain.

The proportion of relatives in Asparn-Schletz is lower than at any other LBK site analysed. We only identified relationships between males and children and only one with an adult male. This raises doubts regarding the idea that the individuals recovered at the ditch represent a local community and instead suggests that people massacred at this key were likely drawn from a widespread population ^44^. When comparing Asparn-Schletz and Nitra Horné Krškany, we find evidence that Asparn-Schletz but not Nitra Horné Krškany represents a large genetic community. One possibility is that Asparn-Schletz was a central site that drew a population from a larger area in times of stress, such as outbreaks of violence ^9^. Another explanation could be that communities in the broader LBK expansion area were formed with few biologically related individuals, as at Derenburg-Meerenstieg II and Stuttgart-Mühlhausen, Germany. In any case, our results suggest that frequent mobility between sites was a factor in many LBK communities ^45^. A lack of related individuals has also been found in the Eneolithic massacre of Potocani, Croatia ^46^.

Our results illuminate how combining whole-burial assemblage ancient DNA, sampled and processed with responsible protocols^47^, with isotopic and archaeological data, can reveal the structure of past societies as well as evidence for local variations in mobility and diet, shedding light on unappreciated aspects of past behaviour.

## Methods

### Ancient DNA Data Generation

The 319 individuals screened in this study were sampled with permission from the authorities responsible for each of them and in engagement with local archaeologist stakeholders, in a way consistent with recommendations for ethical analysis of ancient DNA^47^. Permits for ancient DNA analysis of the skeletal remains was issued to the authors of this work. The permission specified sampling of the skeletal material for ancient DNA analysis, and generation of radiocarbon dates and associated isotopic information. We handled remains with respect, seeking to minimize damage to them for example by prioritizing analysis of disarticulated ossicles or petrous bones wherever possible, and using other minimally-invasive sampling techniques such as drilling from the cranial base or soaking teeth in extraction buffer ^47^. Additionally, we employed a standardized in-solution capture method, which maximizes DNA recovery while minimizing the required input material.

DNA was extracted from powder using an automated protocol with silica-coated magnetic beads and binding buffer ^48^. DNA extracts were converted to double-stranded libraries using a partial UDG treatment ^49^. Amplified libraries were enriched using two rounds of consecutive hybridisation capture enrichment 1240k strategy ^50,51^). Captured libraries were sequenced on an Illumina NextSeq500 instrument with 2 × 76 cycles (2 × 7 cycles for the indices) or an Illumina HiSeq X10 with 2 × 101 cycles (2 × 7 for the index). We trimmed adapters, merged paired-end sequences, and aligned to the human genome (hg19) and mitochondrial genome (RSRS) using BWA 0.6.1 ^52^. The computational pipelines are available on GitHub (https://github.com/DReichLab/ADNA-Tools, https://github.com/DReichLab/adna-workflow).

We evaluated ancient DNA authenticity using several criteria: a rate of cytosine deamination at the terminal nucleotide above 3%; a ratio of Y to combined X + Y chromosome sequences below 0.03 or above 0.35 ^53^(intermediate values are indicative of the presence of DNA from at least two individuals of different sex); for male individuals with sufficient coverage, an X chromosome contamination estimate whose lower bound of the 95% confidence interval is <1.1% (all but one below 0.5%); and an upper-bound rate for the 95% confidence interval for the rate to the consensus mitochondrial sequence that exceeds 95%, as computed using contamMix-1.0.10 ^54^. We added tags to samples that gave evidence of contamination by any of these criteria and discarded samples with at least two signals of contamination.

### Genetic sex, mitochondrial and Y chromosome haplogroup determination

To determine genetic sex, we searched for evidence of a Y chromosome by computing the ratio of Y-chromosomal 1240k positions with available data divided by the number of X-chromosomal and Y-chromosomal 1240k positions with available data. Individuals with a ratio of more than 0.35 were considered genetic males, and individuals with less than 0.03 were considered genetic females. To check for sex chromosome aneuploidies, we computed the mean coverage on X-chromosomal and Y-chromosomal 1240k positions. We normalised these values by autosomal coverage on 1240k positions for each individual. We did not find any evidence of sex chromosome aneuploidies. To determine mitochondrial haplogroups (Supplementary Table 1), we constructed a consensus sequence using RSRS sequence with samtools and bcftools ^55^, restricting to sequences with a mapping quality of >30 and a base quality of >30. We called haplogroups with Haplogrep2.1.1 ^56^. We determined Y chromosome haplogroups (Supplementary Table 1) based on the nomenclature of the International Society of Genetic Genealogy (http://www.isogg.org) version 14.76 (25 April 2019), restricting to sequences with a mapping quality of 30 or more and a base quality of 30 or more. For determining chromosome Y, we analysed not only targeted SNPs but also off-target SNPs, and determined allelic status by majority rule as discussed in detail in Supplementary Table 3, following the methodology described in ^57^. For the statistical tests, we used all the available individuals from the relevant periods as well as all the produced individuals with enough available positions. We met the assumptions of the statistical tests used. We have not assumed normality in the statistical tests.

### Biological kinship estimation and family reconstruction

We followed the same approach described by ^58^. We focused on 1st, 2nd, and 3rd-degree relatives for family reconstruction but also noted individuals detected as relatives up to the 4^th^ degree. The complete list is reported in Supplementary Table 7.

### Principal component analysis and f-statistics analyses

We used Western Eurasian populations genotyped on the Affymetrix Human Origins SNP array to perform Principal Components Analysis with *smartPCA* ^24^. In this PCA, we projected all the samples we report in this paper as well as other relevant ancient DNA data (Supplementary Table 1). We used same dataset to perform f-statistics-based analyses using admixtools 7.0.2 ^24^. (Supplementary Section 5)

We performed *qpAdm* analyses following the same strategy as in Patterson et al. 2022 ^25^. Individuals labelled as Ancient_Africa, WHGB, and Turkey_N were used as right outgroup populations and WHGA and Balkan_N as left sources. qpWave was performed using the same strategy.

### ROH

We called ROH with the methodology described in Ringbauer et al.^35^ optimised for the study of ancient individuals, restricting to individuals with more than 400,000 SNPs. We plotted the results with the python scripts at (https://github.com/hringbauer/hapROH).

### Imputation and IBD

We imputed and phased with GLIMPSE 2^59^ following the methodology in ^60^. We called IBD using ancIBD^60^. We filtered for IBD >12 cM and plotted the connections using Rstudio 4.3.2. Further details are provided in Supplementary Section 5.

### Local ancestry maps

We used diploid imputed genotypes to perform analyses. We ran RFMix v2.03-r0^27^using Balkan_N and WHGA as reference populations. We plotted results with Python 3.7.6 and Rstudio 4.3.2.

### Selection

The selection analysis is detailed in Supplementary Section 5

We can provide the full code used in this project upon request.

## Extended Data

## Supplementary Material

SI

Table - SI

## Figures and Tables

**Table 1:** **Patterns of relationships at three LBK sites with substantial new data** (*p<0.05)

	Polgár-Ferenci-hát (n=45)	Nitra Horné Krškany (n=47)	Asparn-Schletz (n=92)
Ratio related/unrelated	0.83	0.65	0.15
Ratio of males/females related	0.94/0.69*	0.66/0.65	0.1/0.05
Average no. of relatives	1.1	0.60	0.1

## Data Availability

All sequencing data are freely available at the European Nucleotide Archive (ENA) with the accession number PRJEB64177. All the data used to compare the data produced in this study is available in the Allen Ancient DNA Resource (AADR) ^61^
