# Supplementary material for: Social and genetic diversity in first farmers of central Europe
Source: Nat Hum Behav. Author manuscript; Available in PMC 2025 Jan 29. (PMC11774665; doi:10.1038/s41562-024-02034-z)
Supplement: SI [file NIHMS2044401-supplement-SI.docx]

**Table of Contents**

Section 1: Site descriptions

Section 2: Radiocarbon dates and modelling

Section 3: Integration of genetic and isotopic data

Section 4: Supplementary methods for ancient DNA analysis

Section 5: Classification of individuals with genetics methods

Section 6: Kinship

Section 7: Population size inferences

Section 8: Selection scans in the diploid data

Section 9: Supplementary references

**Section 1: Site descriptions**

**1.1 Topole, Bač (Serbia)**

**Author:** Anđelka Putica

In 1977, systematic excavations were carried out at the sites of Šećerana and Topole next to Bač (Northern Serbia, Vojvodina Province) led by Čedomir Trajković (Town Museum of Sombor). Southeast of the town, in the area of the future sugar factory (Šećerana), four pits with material from the Eneolithic period were excavated (Baden/Kostolac ceramic fragments). The excavations were extended to the surrounding areas, between the railway and the Danube–Tisa–Danube channel and the area south of the Bač-Bačka Palanka road bounded by the river Mostonga (Topole). Here, in addition to some Eneolithic finds, Early Neolithic settlement objects, such as pits, the floor of a building structure, pottery vessels, clay statuettes, stone tools, and three contracted skeleton burials, were revealed. On the basis of the findings, the Topole site has been dated to the late phase of Starčevo culture [^1–3^](https://paperpile.com/c/2vFjDD/ICR0G+c0fUs+tmhsi). In Porbe I, Burial 1 (female, 20–25 years old) and Burial 2 (male, 40–50 years old) were uncovered beneath the floor of a structure of an irregular rectangular shape. The well-preserved skeletons were in contracted position 50 cm apart, at the same level, lying on their right sides, symmetrically back to back, with their heads pointing in opposite directions. At the skull of Burial 1, there was a fragment of a ceramic vessel. Next to Burial 2, there were fragments of Starčevo ceramics, a shell, and a chipped stone tool [^1,2^](https://paperpile.com/c/2vFjDD/c0fUs+ICR0G). These burials were conserved *in situ* in their original position along with the soil around them and transported to the Town Museum of Sombor. The calibrated AMS date for Burial 1 is 6216-5917 calBCE (7170±50 BP, OxA-8693) (95% confidence interval), while we ignore the direct date for Burial 2 (8085±55 BP, OxA-8504) [^1,4^](https://paperpile.com/c/2vFjDD/2o0BP+ICR0G). As the archaeological context suggests, simultaneous burials and the burial customs are characteristic of the Early Neolithic period, the Mesolithic date of the Burial 2 samples which also is genetically consistent with being a first-degree relative of Burial 1 might be explained by possible contamination of the Burial 2 sample during the conservation process ^11^ In, 2021 a new date of BACT 2 was produced: 6068-5938 calBCE (7147±28 BP, BRAMS-2411) [^5^](https://paperpile.com/c/2vFjDD/Qa8ym), fully conclusive with the one of BACT1.

- **I7867**: BACT_1, 6216-5917 calBCE (7170±50 BP, OxA-8693)
- **I7868**: BACT_2, 6068-5938 calBCE (7147±28 BP, BRAMS-2411)[^5^](https://paperpile.com/c/2vFjDD/Qa8ym)

**1.2 Donja Branjevina, Deronje (Serbia)**

**Author:** Branislav Vasov

Donja Branjevina is located in northwestern Serbia, next to the village of Deronje (Municipality of Odžaci) on an old alluvial terrace of the Danube, between the former Mostonga River (today the Danube–Tisa–Danube channel) and the Danube attributed to Starcevo culture. The locality itself is located on a curve formed by the alluvial terrace, which is 4-6 m higher than the western part of the terrain, and thus a suitable place for settlement. The site was discovered in 1965, during the construction of the 2nd flood protection line, which was needed to protect against the floods caused by heavy rains and the overflowing of the Danube that year. Immediately after the flood, as well as the following year, probe excavations were conducted here under the leadership of local teacher and amateur archaeologist Sergej Karmanski. Afterwards, archaeological research continued from 1986 to 1996, which started again in 2020 and continues until today. These campaigns were led by several archaeologists, such as Predrag Medović, Bogdan Brukner, Vladimir Leković, and Vojislav Trbuhović, with the participation of Karmanski. In addition to the Neolithic, Late Bronze Age and Medieval horizons have also been confirmed at this site. Settlement objects from the Neolithic period, such as pit houses and trash pits, were documented. In these objects and the layers above them, the most common finds were fragments of ceramic vessels with typical early Neolithic paint or ornaments. In addition, numerous bone and stone artefacts were found, such as spoons, hooks, awls, axes, adzes, hammers, etc. There are also innumerable finds of ceramic altars, ritual vessels, and zoomorphic and anthropomorphic figurines. The most notable find is a female figurine with pronounced steatopygia, better known as the “Red-Hair Goddess” [^4,6–8^](https://paperpile.com/c/2vFjDD/2o0BP+FUx29+vIGz1+xt5YP). The following individual from the site havs been studied:

- **I7712**: grave 3, DON4, 6000-5300 BCE

In probe I/66, in the subsoil between pit 6 and pit 7 (layer 1), a child burial was found. The nearly complete skeleton was in the contracted position, lying on its left side, N/S oriented, facing east, without grave goods [^6,9^](https://paperpile.com/c/2vFjDD/Ux0zn+FUx29). The individual is of the Starcevo culture.

**1.3 Pančevo, Starčevo-Grad(Serbia)**

**Author:** Andrej Starović, Marija Djuric

Prehistoric site “Grad” in the village of Starčevo (south Banat, Serbia) was detected and initially archaeologically tested in the late 1920es [^10,11^](https://paperpile.com/c/2vFjDD/YcOpc+iFdNO). In 1931 and 1932, a joint American-Serbian team headed by V.J. Fewkes conducted large-scale excavations there and discovered indicative remains of an Early/Middle Neolithic permanent settlement. Located on the left aboriginal Danube’s riverbank (Supplementary Figure 1), the site preserved traces of semi-subterranean house constructions, so-called pit-dwellings. A series of such densely constructed houses (more than 15, counting Grbić’s 1928 season, Fewkes 1931–32, and Ehrich and Garašanin 1969-1970 campaigns), and dozens of badly preserved scattered graves had been found during the fieldwork [^12^](https://paperpile.com/c/2vFjDD/RN571). Still today, the name of the site “Starčevo” is an eponym for the entire Early/Middle Neolithic culture in SE Europe. The spatial distribution of named samples among the Neolithic settlement structures at Starčevo is the following (Supplementary Figure 2).


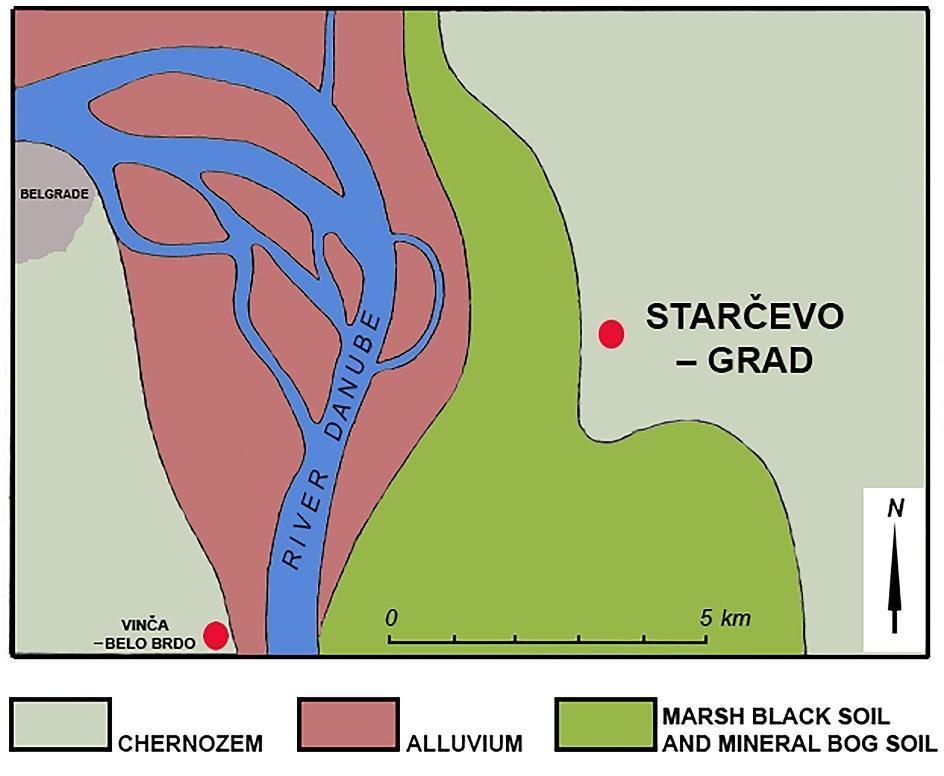


**Supplementary Figure 1:** Location of the prehistoric site of Starčevo–Grad, near the left Danube’s riverbank, near Belgrade, Serbia. Image with permission of publication.


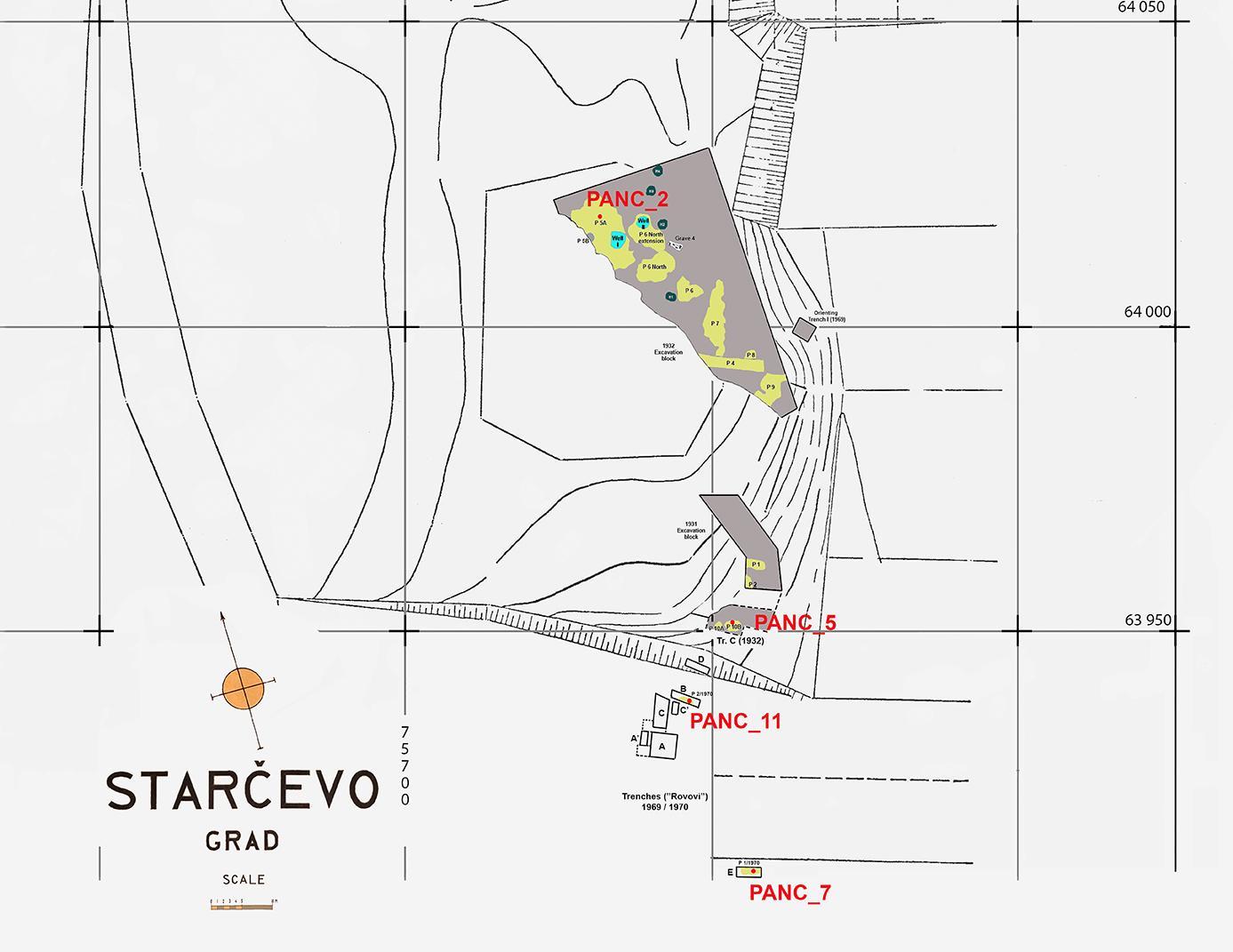


**Supplementary Figure 2:** Part of the excavated area of the site Grad at Starčevo, showing the exact position of the sampled bones; yellow features represent horizontal outlines of 12 different pit-dwellings; sampled groups of human bones, used in this study, are marked with red circles (drawn and digitally processed by A. Starović . Image with permission of publication).

- **I8116**: PANC_2 , 6000-5300 BCE

*Context:* Starčevo-Grad, Sector I-II, Pit 5A, burnt level, bag 578, September 2, 1932.

Pit 5A presents the largest and stratigraphically the most complex pit-dwelling found at Starčevo, having (at least) two superimposed living horizons, as well as a rare architectural structure – freshwater well. The “burnt level” is the uppermost layer, where the bone remains of two different persons had been discovered. Indirectly, the layer itself was dated from the sample taken from cervid antler (OxA-8560): 5490–5360 cal BC [^4,13^](https://paperpile.com/c/2vFjDD/2o0BP+eKOGO).

This unit contains two sets of human bones, both consisting of cranial fragments of adult persons. The first set comprises frontal bone with glabella and left supraorbital foramen with a fragment of the right supraorbital margin. The coronary suture is not fused, which implies a younger adult person. Right zygomatic bone with preserved nasal bone and right maxilla is present, as well. The maxilla is damaged at the level of the first incisor. The second incisor and the canine were lost post-mortem, but both permanent premolars are present. The first and the second molars are absent, and lost post-mortem (Supplementary Figure 3).

**
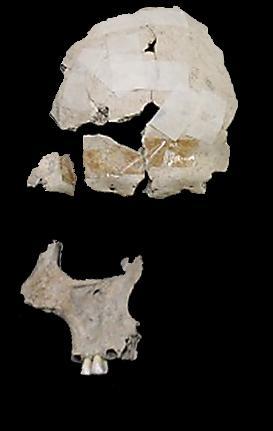

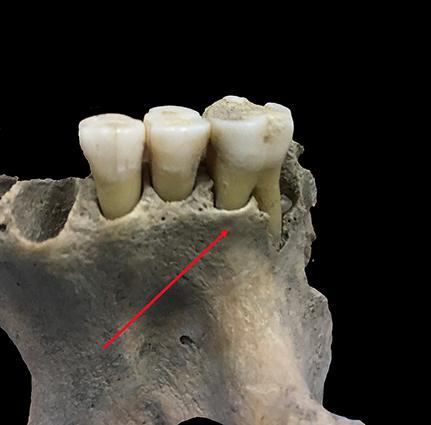
**

**Supplementary Figure 3**: Human bone remains from a sampled individual, named “Panc_02”; M1 taken from the upper jaw was marked by a red arrow (photo by M. Djuric, digitally processed by A. Starović with permission of publication).

The second group of bones from the same unit contains several fragments of parietal bone with preserved sagittal, lambdoid, and squamosal suture. Obviously, this skull of an adult (PANC_1) does not fit morphologically to previously described bone remains, so MNI from this unit is 2 (Supplementary Figure 4)*.*


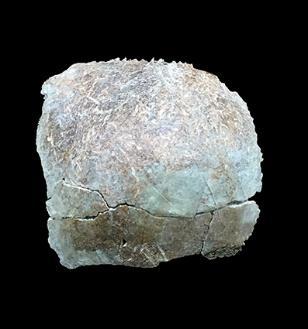


**Supplementary Figure 4:** Human skull remains from another individual from the same unit, signed as “Panc_01”. Image with permission of publication.

- **I8105**: PANC_5, 6000-5300 BCE

*Context:* Starčevo-Grad, Sounding C, Pit 10B, bag 578, human bones found among animal bones, September 15, 1932.

Pit 10 (A and B) had been found in the up to back then southernmost excavated part of the site, in the scope of the last explored “Sounding C”. Numerous animal bones had been found during the excavations in the context of the pit-dwelling floor, but after the subsequent analysis, two fragments of human bones have been recognised among the animal ones, both deriving from Pit 10B (eastern room of the dwelling) (Supplementary Figure 5). The first bone is a fragment of parietal bone with part of a lambdoid suture. The fragment implies an *adult* (*probably younger*) person because the sutures were not fused. The second is the fragment of the body of the mandible belonging to a *young adult*, its right side at the level of the second premolar, first and second molar. The first premolar was lost post-mortem, and the third molar was taken as a sample for palaeogenomic study. The other two molars are present, tied to bone fragments. The first molar shows progressive abrasion up to exposed dentine on the top of tooth cusps. Alveolar ridge resorption, in height of 3–4mm, was detected on the first molar, as well. *Foramen mentale* is visible on the body of the mandible fragment.


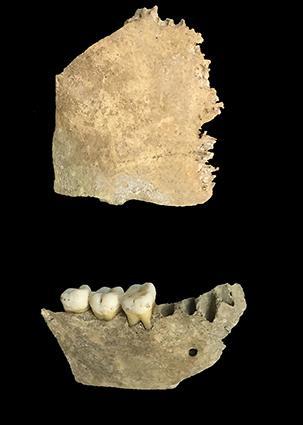


**Supplementary Figure 5**: Occipital and mandible fragments of human skull from Pit 10 at Starčevo, Serbia (photo by O. Cheronet, digitally processed by A. Starović with permission of publication).

Two-room spatial structure of the Pit 10 (A and B) was absolute dated, using the sample taken from a worked ovicaprid metapodial bone (OxA-8561): 5930–5720 cal BC [^4,13^](https://paperpile.com/c/2vFjDD/2o0BP+eKOGO). As a living structure (pit-house), this is the oldest ever found and excavated at Starčevo. Two samples had been taken from the above-described mandible fragment: the right lower tooth M3, and a small piece of the mandible body (Supplementary Figure 6).


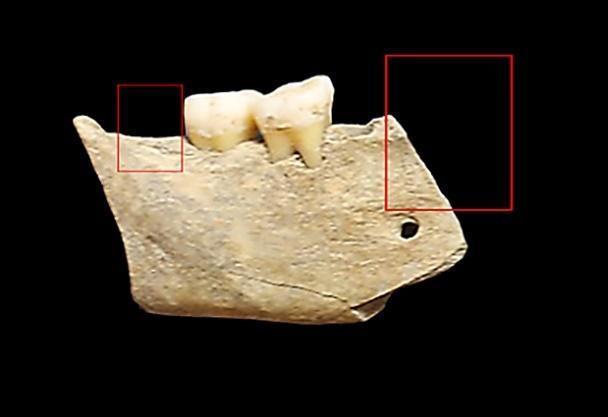


**Supplementary Figure 6**: Mandible fragment from Pit 10B at Starčevo, after taking the samples; red rectangles are showing their exact position (photo by M. Djuric, digitally processed by A. Starović with permission of publication).

- **I8117**: PANC_7, 6000-5300 BCE5765-5631 calBCE (6820±35 BP, PSUAMS-15077)

*Context:* Starčevo-Grad, Trench E – east, Pit 1/1970, “below the 2^nd^ level of white nuggets” bag 214A, October 1, 1970.

The pit had been discovered in the scope of the southernmost excavated trench of the site. In the internal stratigraphy of the pit itself, under a thin layer consisting of small nuggets of white clay, several fragments of badly destroyed human skulls were detected. That particular layer in the pit was absolute dated indirectly, using two animal bones (GrN-9035 and GrN-9037): 5810–5640 cal BC and 5710–5550 cal BC [^13,14^](https://paperpile.com/c/2vFjDD/eKOGO+uLoPl).

The skull is present as fragmented (cranial vault and base): fragmented frontal bone, the left parietal, the right parietal with a fragment of the right temporal bone; then, a fragment of the left part of the occipital bone with the left temporal is preserved. All cranial sutures are lost, implying that this is most probably *an older adult (maturus)*:

- **I8109:** PANC_11, 6000-5300 BCE

*Context*: Starčevo-Grad, Trench B, Pit 2/1970, bag 99, September 28, 1970

This stratigraphically complex pit (similar to Pit 5A from 1932) had been explored in two fieldwork seasons (1969 and 1970). Its lower horizon was discovered below a thin layer of yellow clay, and here two scattered human bones were found (Supplementary Figure 7):


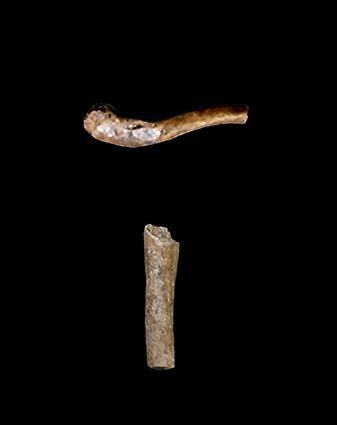


**Supplementary Figure 7**: Two child’s bones found in the lower horizon of the Pit 2/1970, “below yellow clay” (photo by M. Djuric, digitally processed by A. Starović with permission of publication).

The unit lower layer of Pit 2/1970 could be absolute dated, according to one sample of animal bones (GrN-7155): 5780–5630 cal BC. The first one is the right (lateral) half of the right clavicle belonging to a young child.

The second one presents a proximal fragment of the diaphysis of the left femur of a very young child (age assessed to a range between 2 and 4 years – based on comparison with casted models from Prof. Djuric bone reference collection). The child’s clavicle was sampled for the purpose of a palaeogenomic study (Supplementary Figure 8).


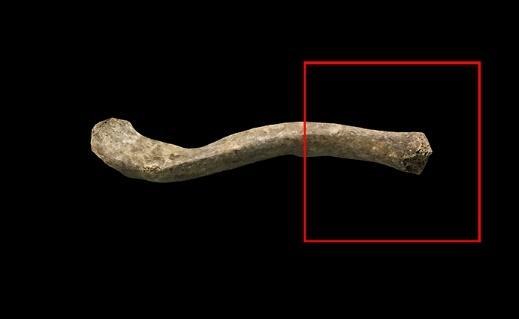


**Supplementary Figure 8**: Right clavicle of a child from the Pit 2/1970, red rectangle showing the sampled bone part (photo by O. Cheronet, digitally processed by A. Starović with permission of publication).

**1.4 Siklós-elkerülő út (Csukma-dűlő) (Hungary)**

**Author**: János Jakucs

The site ’Csukma-dűlő’ is located in the north-western part of the town of Siklós, near the locality of Máriagyűd, in Baranya County (Southern Hungary). The site was discovered during the construction of the road bypassing Siklós from the north in 1999 (archaeologist: István Ecsedy). Remains of a large-scale settlement of the early Neolithic Starčevo culture were observed during the excavation. A vast number of ceramic fragments, burnt daub, animal bones, and stone tools were uncovered from the early Neolithic layers and pit complexes. One of the partially uncovered early Neolithic pit complexes contained the poorly preserved skeleton of a 35-45-year-old female. On the basis of the pottery style, the discovered assemblage is plausibly derived from the latest phase of the Starčevo culture.

- **I29876**: HUNG 447/HUNG 483, 5700-5500 BCE

**1.5 Vörs-Máriaasszony sziget (Hungary)**

**Authors**: Nándor Kalicz, Katalin T. Biró, Zsuzsanna M. Virág

On the territory of the village Vörs, lying at the eastern margin of the former Kis-Balaton („Little Balaton”) marshes, on the western border of Somogy county facing the neighbouring Zala county, were sites and finds from various periods from almost all periods of prehistory since the Early Neolithic. Finds include materials from the Early Neolithic (Starčevo culture), from the Early Copper Age Lengyel III. culture, from the Middle Copper Age Balaton-Lasinja culture, from the Late Copper Age Kostolac culture, from the Early Bronze Age Kisapostag culture, from the Late Celtic and (Early-) Roman period, and from the (Early Medieval-) Árpád-dynasty period. Here we analyse one individual attributed to the Starčevo culture[^15^](https://paperpile.com/c/2vFjDD/CAbFW), with a consistent new radiocarbon date.

- **I17927**:HUNG517, Grave 1, 5631-5482 calBCE (6645±40 BP, PSUAMS-15078)

**1.6 Vinkovci – NaMa (Croatia)**

**Authors:** Mario Šlaus, Željka Bedić, Maja Krznarić Škrivanko

Tell Tržnica is the oldest prehistoric settlement located on the elevated left bank of Bosut in Vinkovci, first mentioned in 1902 by Josip Brunšmid [^16^](https://paperpile.com/c/2vFjDD/seRAI). The first finds of the Starčevo culture in Vinkovci were discovered in 1951 during the levelling of the southern part of the market (Tržnica is market in Croatian) [^17^](https://paperpile.com/c/2vFjDD/2hvH2). The first trial excavation on the tell Tržnica was carried out in 1962 by S. Dimitrijevic, and it confirmed the existence of a settlement of the final, spiral B phase of the Starčevo culture[^18^](https://paperpile.com/c/2vFjDD/6d4M9) . The first rescue archaeological excavation on the tell Tržnica was carried out as early as 1973. With the intensive urbanization of Vinkovci in the second half of the 1970s, large rescue excavations were carried out in the central part of the tell Tržnica. In the Eastern part of 2632 m2 rescue excavation in Duga street 26 (Na-Ma) archaeologists documented edge of Starčevo culture settlement with pit dwellings, waste pits and 11 graves buried in previously abandoned pit dwellings[^19^](https://paperpile.com/c/2vFjDD/1e7Ap).Of 11 graves discovered, 3 graves are presented in the paper. Graves 7 and 11 were laid in the same large pit dwelling in the quadrant LIV, in which there was 5 graves (7, 8, 9, 10 and 11). Grave 15 was buried in a large elongated object in quadrant LXXVI, as were the graves 16 and 17, which were severely damaged by later burials [^20^](https://paperpile.com/c/2vFjDD/faaYc). The head of archaeological research conducted in 1976 and 1977 was Ivana Iskra-Janošić of Vinkovci Municipal Museum. Iskra-Janošić mentions 11 prehistoric graves found in both supine and flexed positions, with rare finds laid directly on the soil and, in the majority of cases, situated at the edges of the dwellings[^21^](https://paperpile.com/c/2vFjDD/eSM5g). The geographical location of the Vinkovci-tell Tržnica site expands from the Bosut river at the location of the modern hotel Slavonija (known today as Duga ulica 1) along the promenade of D. Švagelja and Duga ulica, westwards to the location of the former Na-Ma department store (today Duga ulica 26), which represents the northwestern end of the site and settlement. The Vinkovci NaMa micro-toponym is located on the north-western side of the Vinkovci-tell Tržnica site [^22^](https://paperpile.com/c/2vFjDD/S9Cfa). Four burials associated with the Starčevo culture contained skeletons in flexed positions lying either on their left or right side. Only one burial contained grave goods in the form of a ceramic vessel. In this project, we included graves 11, 15, and 7 [^23^](https://paperpile.com/c/2vFjDD/usoJp).

- **I28425:** grave 11, 5650-5450 BCE

The skeleton was buried in a crouched position on the left side, located below grave 7 in a large pit dwelling, in quadrant LIV at a depth of 3.20 m. The skeleton was damaged, lying on his back with legs flexed and placed to the left, hands laid side by side and bent at the elbows, fists laid in front of the face, north-south orientation.

The skeleton’s dimensions were: skeleton length 0.62 (0.96) m; femur 0.38 m; lower leg length 0.31 m. No grave goods were found. This morphological female aged 30 to 35 years exhibits only dento-alveolar pathologies: five caries lesions, two abscesses, and six teeth lost antemortem.

- **I28427:** grave 15, 5650-5450 BCE

The skeleton was buried in a crouched position on the right side in a large object in the quadrant LXXVI, at a depth of 3.60 m. The skeleton is laid on the right side, with the head facing south, hands bent at the elbows, fists laid in front of the face, east-west orientation with the head to the west facing south. The skeleton’s dimensions were: skeleton length 1.20 m, femur 0.46 m. Fragments of Starčevo culture pottery were found under the legs.

This morphological male skeleton aged 30 to 35 years has several pathological changes. Ectocranial porosity is present on both the parietal and the occipital bone. Linear enamel hypoplasia is recorded on the maxillary and mandibular canines. Degenerative osteoarthritis is present on one thoracic and one lumbar vertebra and Schmorl's node on one thoracic vertebra. On the lateral condyle of the distal right femur, a possible osteochondritis dissecans measuring 14×10 mm is present while on the same location on the left femur, a protrusion measuring 8×5 mm is observed.

- **I28426**, grave 7, 5625-5482 calBCE (6630±35 BP, PSUAMS-15082)

The skeleton was buried in a crouched position on the left side in the western part of a large pit dwelling in the LIV quadrant at a depth of 2.70-3.00 m. The skeleton was damaged, lying on the back with hands bent at the elbows, legs flexed and placed to the left, north-south orientation with head to the south, face to the west. The skeleton’s dimensions were: length 0.86 m; upper arm 0.28 m; forearm 0.27m; lower leg 0.33 m. No grave goods were found. The skeleton belongs to a male aged 17 to 19 years at the time of death. Pathological changes include cribra orbitalia in the left orbit, ectocranial porosity on both parietal and the occipital bone, and benign cortical defect on the latissimus dorsi muscle attachment of the right humerus.

**1.7 Magura Buduiasca, Teleor 3 (Romania)**

**Authors:** Catalin Lazar, Pavel Mirea

The site of Magura-Buduiasca (TELEOR 003) is located on the lower eastern terrace of Teleorman River, 8 km from Alexandria town and 45 km north of the Danube River. This flat settlement includes several habitation horizons belonging to the Early and Middle Neolithic period (Starčevo-Criş, Dudeşti, and Vădastra cultures), spanning 6100 to 5200 BCE.

The Early Neolithic site of Magura occurs in an area of loess soils that overlie marl (calcareous sediment that includes silt-sized quartz) on the edge of high ground overlooking the floodplain of the Teleorman River. The habitation consists of pits, hearts, and pit huts with different dimensions and depths. All these features are cut into the marl [^24^](https://paperpile.com/c/2vFjDD/8PM5L). The features contain rich material culture: potsherds, figurines, flint and stone tools, grinding stones, wood items, bone ornaments and tools, shells, and animal bones [^25^](https://paperpile.com/c/2vFjDD/LRIST). One of the significant features of this settlement is the presence of a considerable number of scattered human bones [^26^](https://paperpile.com/c/2vFjDD/lVqIM).

Pit C57 (aka Cpl. 57) was identified in the S48 survey. It had an irregular shape, with more fill levels that contained pottery sherds, flints, other small finds, animal bones, and human bones. All human bones are scattered in the pit fill, without anatomical connection or other signs of funerary treatment. Generally, the anatomical elements represented here are fragments of skulls, fibula and femur diaphysis, vertebrae, phalanx, and teeth. According to the C14 data, the C57 pit was used between 6066-5741 calBCE. Two samples from the C57 feature were analysed in this study. The analysed samples included in this study come from a pit (C57) and the Starčevo-Criş habitation levels (Supplementary Figure 9).

- **I6174:** ROM-02-2017, 6074-5927 calBCE (7155±35 BP, PSUAMS-3903)

A left mandibular central incisor (LI1) was identified in Unit 2870, at -1.40/-1.50 m. It belongs to an adult.

- **I6699:** ROM-05-2017, 5292-5000 calBCE (6180±45 BP, PSUAMS-3983)

A right mandibular central incisor (RI1) was identified in Unit 2871, at -1.50/-1.60 m. It belongs to an adult. This incisor, along with the other one identified in Unit 2870, probably belongs to the same person.

- **I6181:** ROM-07-B1, 6000-5300 BCE

An isolated human mandible fragment was identified in Unit 2874 at -1.70/-1.80 m. It belongs to an adult inferred to be morphologically female.


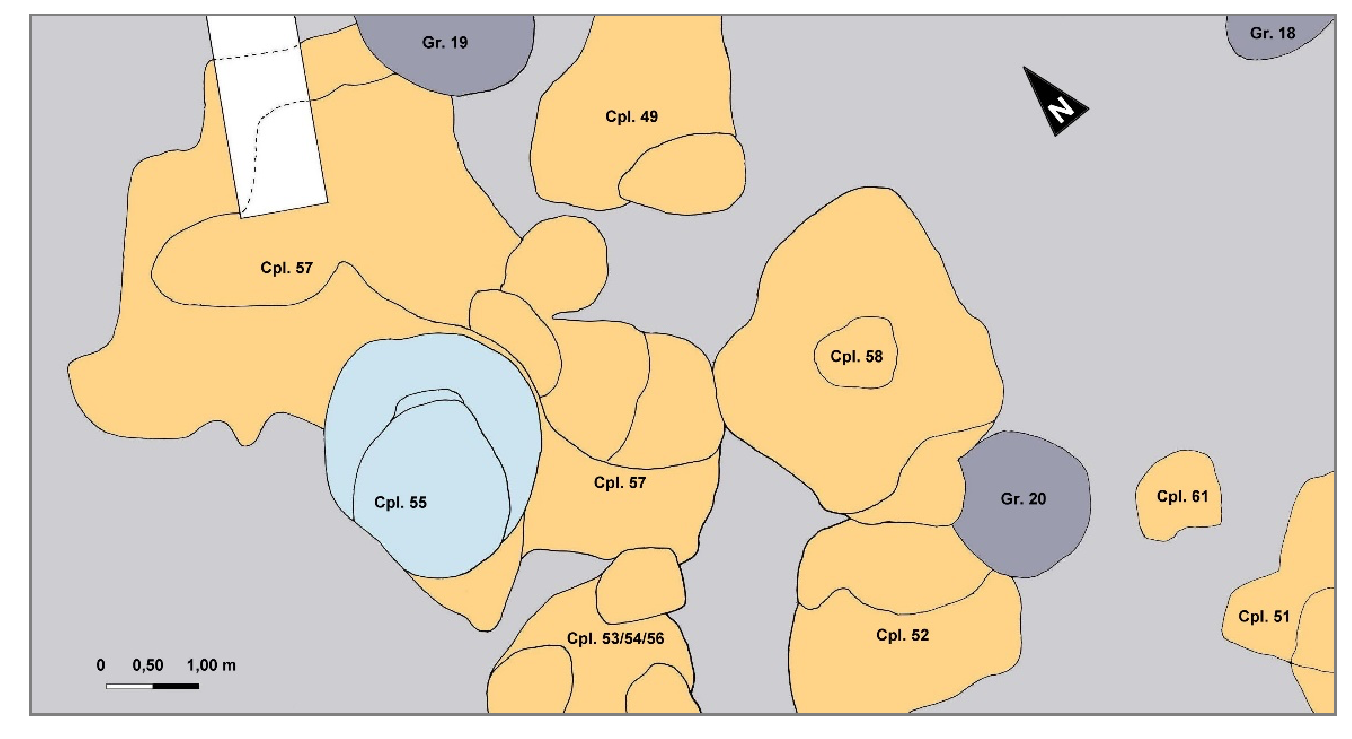


**Supplementary Figure 9:** Map of the S48 survey and location of pit C57 (Cpl.55) and Cpl.57. Image from Catalin Lazar with rights of publication.

**1.8 Donja Strana-Velesnica (Serbia)**

**Author:** Dusan Boric

Velesnica is situated on the right bank of the Danube in the Ključ region of the Danube Gorges area, around 50 km from Lepenski Vir as the crow flies or around 100 km downstream along the Danube. The area of 660 m^2^ was excavated in 1980–1982 [^27^](https://paperpile.com/c/2vFjDD/7UIXt). There is a very thin, lowermost occupation layer that can be assigned to the Mesolithic, with several animal bones and bone tools [^28^](https://paperpile.com/c/2vFjDD/4As6H) found beneath the Early Neolithic levels. This layer remains undated at present and was found beneath the layer with Early/Middle Neolithic Starčevo ceramics, burnt house daub, and other artefacts. The Early Neolithic phase at Velesnica is characterised by Starčevo culture ceramics, and the thickness of the occupation layer varies from 1.5–1 m deep along 80 m of the river bank. Irregular stone constructions and a circular hearth with burnt soil, as well as a stone foundation, were found.

The burials at Velesnica can be dated to the Early/ Middle Neolithic period on the basis of their stratigraphic context, association with Neolithic material culture, and crouched body position of the deceased [^27,29^](https://paperpile.com/c/2vFjDD/7UIXt+raVpa). They have also been directly AMS-dated [^30^](https://paperpile.com/c/2vFjDD/qVbt3). The burials were concentrated in two adjacent excavation areas marked as Block A and Trench 8 [^29^](https://paperpile.com/c/2vFjDD/raVpa). Burial 1 was a very contracted child skeleton aged between three and seven years old, which lay on its lateral left side and was oriented north-south. The burial was found directly underneath a large stone “altar”/mortar [^27^](https://paperpile.com/c/2vFjDD/7UIXt), the pressure from which appears to have crushed the child’s skull, which was very fragmentary [^31^](https://paperpile.com/c/2vFjDD/KQMPD). A ceramic bowl accompanied the individual in this burial and can probably be considered a grave offering. Burial 2A-G at Velesnica contained the remains of five primary burials, some of which were placed on top of the other [^27^](https://paperpile.com/c/2vFjDD/7UIXt). There were also the remains of two disarticulated individuals. This multiple-burial was located in Trench 7, around 15 m north of Trench 8, and the inhumations were found within a pit with a diameter of around 1.2 m infilled by Early Neolithic Starčevo ceramics, red burnt soil, mollusc shells, and animal bones. The difference in depth between the bottommost burial (at 34.84 masl) and the topmost burial (35.30 masl) was only 0.46 m, and the excavator notes that the burial cut was made from only 0.1 m above the head of the most recent burial, at around 35.4 masl [^27^](https://paperpile.com/c/2vFjDD/7UIXt)). The most recent inhumation, Burial 2A(+2E), was of an adult woman in a very crouched position, perhaps on her right hip, oriented east-northeast and located along the north edge of the burial pit. A second adult female, Burial 2B(+2F) (Supplementary Figure 10), was placed in the centre of the burial pit and oriented east-west. She lay on her lateral left side, and her upper limbs were flexed at the elbow. This individual was also characterised by a series of dental pathologies, including caries on two teeth, which is infrequent in the preceding forager population of the Danube Gorges [^31^](https://paperpile.com/c/2vFjDD/KQMPD). On the southern edge of the pit lay Burial 2C, of a child between seven and eleven years of age. The child was laid to rest on its lateral right side with the upper limbs flexed at the elbow and was oriented west-east. Burials 2B and 2C were found at approximately the same level within the burial pit, faced each other, and were arranged in symmetrical positions. In the central part of the burial pit and directly beneath Burial 2B, Burial 2D was found of an older adult female. This individual was laid to rest on her lateral left side and was oriented northeast-southwest. Thus, superposed Burials 2B and 2D were both laid to rest on their lateral left sides but with diametrically opposite orientations so that the skull of Burial 2B lay over the lower limbs of Burial 2C [^29,32^](https://paperpile.com/c/2vFjDD/raVpa+WPs2D). At the bottom of the burial pit was Burial 2G, of a child lying with the torso on its back, with the thigh bones flexed on the torso and with the legs flexed on the thigh bones, splayed outwards and crossed at the ankles. The final burial from Velesnica, Burial 3, was found in block A, and was of a young woman laid to rest in a crouched position on her right side and oriented south-north [^33^](https://paperpile.com/c/2vFjDD/kq6eP). Bonsall et al., 2015 reported seven AMS measurements that date seven individuals from Velesnica. All available skeletal remains from the site have now been dated apart from burial 3. The obtained date for Burial 1, after correction for the aquatic reservoir effect, falls in the first 150 years of the sixth millennium BCE (95% confidence). On the other hand, measurements obtained for six primary burials in multiple Burial 2, after correction for the aquatic reservoir effect and modeling within the Bayesian statistical framework, suggest that all these burials were placed here, most likely between 6065–5995 calBCE (95 % probability)[^32^](https://paperpile.com/c/2vFjDD/WPs2D). The exception is a Middle Mesolithic date obtained on disarticulated remains of a neonate found with primary Burial 2G at the bottom of this multiple burial place.

- **I5770**: VELE_2B, Tr. 7/7, spit XVIII-XIX, 6221-6019 calBCE (7235±44 BP, OxA-19192; corrected from 7385±39 BP)

Velesnica Burial 2B (Lower left PM4, deformed and worn). *Context*: Tr. 7/7, spit XVIII-XIX. The remains of this individual were directly AMS-dated by OxA-19192: 7385±39 BP (uncorrected for the freshwater reservoir effect); 7235±44 BP (corrected for the freshwater reservoir effect); 6215–6020 calBCE (calibrated range at 95% confidence); δ13C=–19.3 δ15N =10.7, C/N=3.2

- **I13162**:VELE_2C, grave 842 + 843, 6081-5912 calBCE (7145±45 BP, OxA-19209; corrected from 7245±39 BP)

Velesnica Burial 2C (right petrous). The remains of this individual were directly AMS-dated by OxA-19209: 7245±39 BP (uncorrected for the freshwater reservoir effect); 7145±45 BP (corrected for the freshwater reservoir effect); 6081–5912 calBCE (calibrated range at 95% confidence); δ^13^C=–19.2 δ^15^N =9.9, C/N=3.2

**
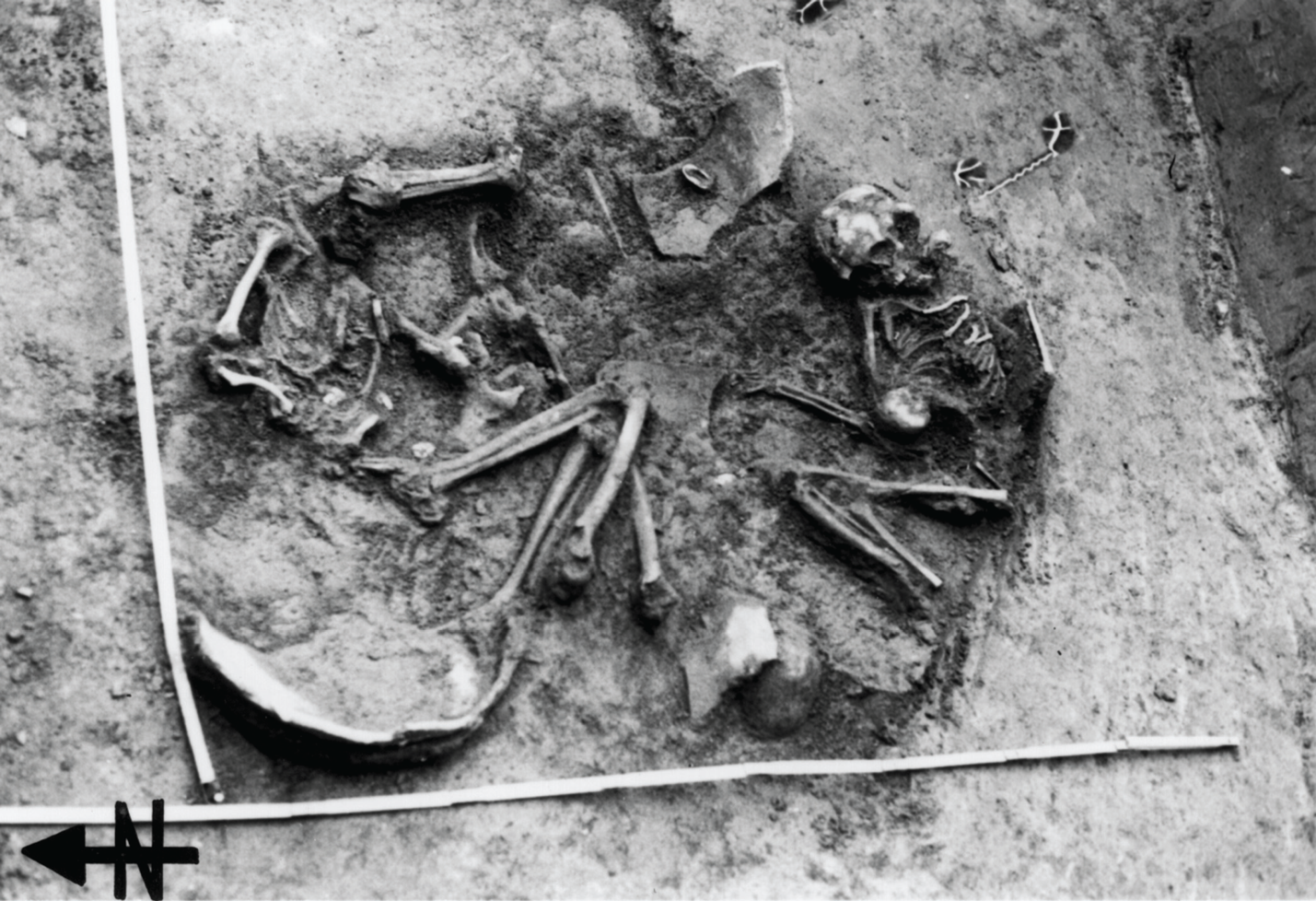
**

**Supplementary Figure 10:** Individuals 2B(+2F) and 2C, Velesnica, found buried in flexed positions on their lateral sides symmetrically facing one another. Picture from Dusan Boric with rights of publication.

**1.9 Egerág-Gyilkos-tó (HT 156. site), (Hungary)**

**Author:** János Jakucs

This site is located northwest of the village of Egerág, in Baranya County. The site was discovered during an archaeological excavation before the construction of the Croatian-Hungarian gas interconnection pipeline in 2010 (Archaeologist: Jácint Ligner). Although the excavated material was first attributed to the Transdanubian LBK and reported as such[^34^](https://paperpile.com/c/2vFjDD/nXbvH), during the re-examination of the archaeological material (by János Jakucs in 2024) it was proven that it belongs to the early Neolithic Starčevo culture, which was also confirmed by the radiocarbon dating of the human remains. Based on its absolute date, the burial from Egerág can be associated with the earliest horizon of early Neolithic communities in Transdanubia. Grave 2/4: This skeleton was discovered in Pit 2. The partially preserved skeleton of the 50-59-year-old man, oriented SE-NW, was lying on its right side in a semi-crouched position: the head was strongly bent back, the torso was slightly twisted, and the legs were slightly pulled up. The burial did not contain any grave goods or findings relating to clothing. The position of the body suggests that it may not have been a traditional burial but rather a corpse that was thrown into the pit.

- **I29877:** HUNG 484, grave 2, 6069-5909 calBCE (7120±40 BP, PSUAMS-15083)

**1.10 Szentpéterszeg–Körtvélyes II (Hungary)**

**Authors:** Tamara Hága

The site is located between Berettyóújfalu and Szentpéterszeg, north of the Berettyó River, east of the Herpály tell, and on the southern high bank of a watercourse. After a rescue excavation, several features from different periods were found, the vast majority of which dated to the Neolithic. In the south-eastern part of the site, there were Early Neolithic (Körös culture) and Middle Neolithic (Alföld Linear Pottery culture, Esztár group) settlement features and burials, while in the north-western part of the site, on a hill, Late Neolithic (Herpály culture) features were excavated.

The burials and human remains related to the Körös culture were not found in separate burial graves but in settlement features. One human skeleton was usually found in each feature. The exception was the largest pit (Feature 50, SNR: 81) dating to the Early Neolithic, which was originally used for clay mining and which contained sixteen contracted burials and the remains of eight human skulls. The amorphous pit, measuring 21 × 17 cm, was 180 cm deep from the surface without humus. It contained very few finds (pottery, a steatopygous idol, animal bone, wattle and daub, snail, shell, chipped and polished stones, grinding stone) compared to its size and to other Early Neolithic features. All but one of the skeletons and skulls were located at or near the bottom of the pit, on a thin layer of fill. The filling of the graves was identical to that of the pit, with no evidence of digging in. Only the upper part of the pit was intersected by a ditch system from the Árpád Period. An intact horn of an auroch was also found near the burials in the northern half of the pit, on the same level as the burials. The majority of the human remains were female: nine of the sixteen skeletons were female, three were male, and four of them were children. Five of the eight skulls belonged to women, one to a man, one to an adult of indeterminate sex, and one to a child. The orientation of the graves and the degree of their contraction varied [^35^](https://paperpile.com/c/2vFjDD/NYKUP).

- **I14962**: Obj. 88, Strat. 144, 5665-5525 calBCE (6680±35 BP, PSUAMS-9998)

Feature 88 SNR 144 (I14962, 2136): Burial of an adult female (aged 25-30) at the bottom of the pit, in an oval digging in (L: 147 cm, W: 76 cm, D: 12 cm), excavated in 2015. The deceased was lying on her right side in a slightly contracted position, with her hands in front of her face. Bones are in medium preservation. The skeleton was oriented ENE to WSW. There was no grave good in the grave.

- **I14965**: Obj. 82, Strat. 132, 5800-5300 BCE

Feature 82 SNR 132 (I14965, 2137): The burial of an adult female (aged 25-35) at the bottom of the pit excavated in 2015. The deceased was lying on her right side in a medium contracted position, with her skull tilted back. The bones of the upper body were slightly disturbed. Bones are in medium preservation. The skeleton was oriented SE to NW. There was no grave good in the grave, the three fragments of vessels near the deceased can be interpreted as finds of the pit.

- **I15073**: Obj. 141, Strat. 259, 5835-5484 calCE [union of two inconsistent dates: 5835-5667 calBCE (6865±35 BP, PSUAMS-10195), 5655-5484 calBCE (6665±35 BP, PSUAMS-9999)]

Feature 141 SNR 259 (I15073, 2138): Burial of an adult female (aged 20-25) at the bottom of the pit, excavated in 2015. The deceased was lying on her left side in a strongly contracted position, with her hands in front of her face. Bones are in medium preservation. The skeleton was oriented SE to NW. Next to the right femur was an animal bone. The skull of another individual was found 30 cm southwest of the skeleton.

- **I15074**:Obj. 320, Strat. 623, 5800-5300 BCE

Feature 320 SNR 623 (I15074, 2139): The burial of an adult female (aged 25-30) at the bottom of the pit, excavated in 2015. The deceased was lying on her left side in a slightly contracted position. The skull was tilted back. The left arm was in an extended position beside the body, and the hand was partially under the right knee. The right arm was behind the deceased’s back and the hand was under the pelvis. Bones are in medium preservation. The skeleton was oriented WSW to ENE. There was no grave good in the grave.

- **I18614**:Obj. 309, Strat. 606, 5800-5300 BCE

Feature 309 SNR 606 (I18614, 2135): The burial of an adult female (aged 30-50) 20-30 cm above the bottom of the pit, excavated in 2015. The deceased was lying on her left side in a medium contracted position. The skeleton is partially incomplete. Bones are in bad preservation. The skeleton was oriented NW to SE. There was no grave good in the grave.

**1.11 Turia (hu: Torja) – Apor-kúria kertje (Romania)**

**Authors:** Sándor József Sztáncsuj

The site of Turia–Apor-kúria kertje is located in South-East Transylvania, in the piedmont area on the western edge of the Black River (Feketeügy/Râul Negru) basin. The site was discovered and researched by Zoltán Székely in 1984–1986. Archaeological excavations have revealed traces of habitation from several periods, from the Neolithic to the Middle Ages. The earliest settlement remains from the site belong to the Neolithic Starčevo-Criș Culture, consisting of a 40-50 cm thick habitation layer. Excavations brought to light the remains of three semi-subterranean houses, each of them with an area of approximately 3×3 m, with walls built on a wooden structure covered with clay. The rich archaeological material discovered in the habitation layer and inside the houses, consisting of pottery, stone and bone tools, and various clay artefacts, date the settlement to the late phase of the Starčevo-Criș Culture. Four inhumation graves have also been unearthed from the area of the early Neolithic settlement. The individuals were lying in a contracted position, oriented north to south. The particularly poor funerary inventory consisted of a few fragments of vessels deposited next to the deceased. One of the graves, with no funerary inventory, was disturbed by a pit from a later period.

- **I21906_d**: Grave 2, 5623-5483 calBCE (6625±30 BP, PSUAMS-14999)

Burial of a (possible) adult, discovered in 1984, in trench number II, at a depth of 37 cm. The body was lying on the right side, in a contracted position, oriented north to south, with the head on the north side. The skeleton was incomplete, with only the upper part being preserved, the rest being destroyed by a pit from the Dacian period. No burial goods.

**1.12 Hencida, Csörsz-árok II./Gyűrű-szeg 2. (Hungary)**

**Author:** László D. Szabó

Rescue excavation during the construction of the M4 motorway between Berettyóújfalu and Nagykereki. Several graves were found from different archaeological periods. Two are attributed to Early-Middle Neolithic culture based on body position and materials.

- **I17455**: HUNG515, Feature 9, 5350-5150 BCE

The individual burial of a juvenile (14-15 years old) (Supplementary Figure 11) was found in the sub-humus layer in the probe nr.111. The deceased boy was lying in the fetal position (strongly contracted legs, drawn up tightly; arms bent at the elbows, pulled in front of the face) on his left side. No grave goods were observed. The body was positioned in the E-W direction. The grave pit was rounded, rectangular, and shallow.

- **I18642**: HUNG516, feature 5, 5312-5211 calBCE (6275±25 BP, PSUAMS-14126)

This is a child burial (9-11 years old) found in the fill of a shallow Early Neolithic (Koros) waste pit (Feature 5/Str 5). The deceased was lying in the fetal position (strongly contracted legs, drawn up tightly; arms bent at the elbows, pulled in front of the face) on the left side. The body (especially the part behind the skull) was surrounded by pottery and bone fragments. A bovine mandible was found 30 cm behind the body. These are interpreted to be finds from the pit and not intentional grave goods.


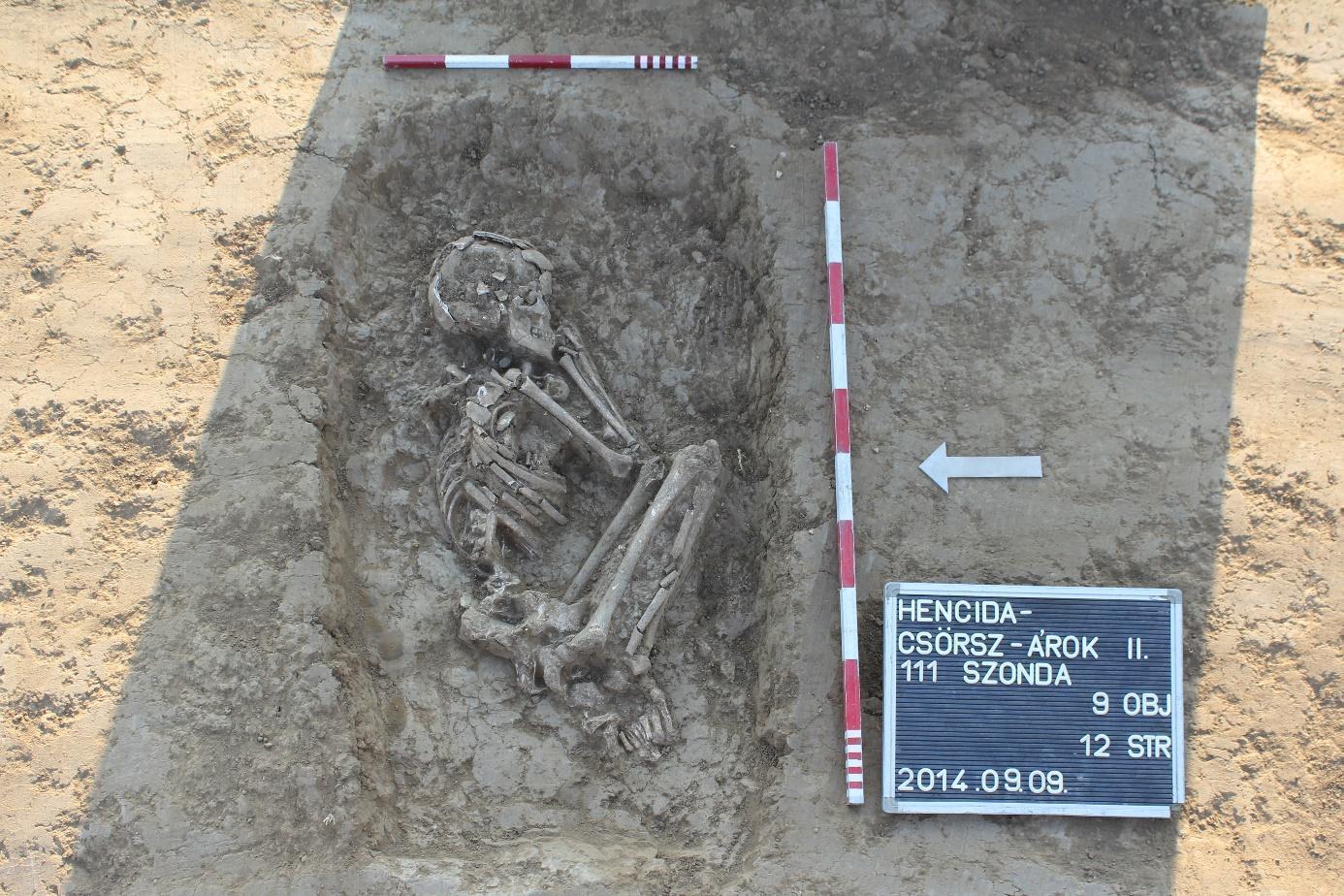


**Supplementary Figure 11:** Grave from Hencida, image from László D. Szabó with permission of publication.

**1.13 Arnót –Arnóti-oldal Dél (Hungary)**

**Author:** Krisztián Tóth

Arnóti-oldal Dél site is situated in Borsod-Abaúj-Zemplén county, Northeastern Hungary, 2 kilometers from the eastern part of Miskolc city and 900 meters to the West from the village of Arnót. The Sajó river widens here and becomes more river branches. According to the maps of the military surveys of the 18th and 19th centuries, the area between the river branches was marshland. Geographically this area is the border between the Great Hungarian Lowland and the North Hungarian Mountains. In this swampy territory, small hills were suitable for human activity such as the small hill where the site is located closer to the Eastern river branch of the river, which is called Kis-Sajó (Small Sajó river).

Large-scale rescue excavations were carried out connected to the building of the M30 motorway around Miskolc in 2014-2015. This bypassing part of the motorway is around Miskolc city leading through the swampy area mentioned above. Therefore, they had to build an embankment for the road, but this would have prevented the flood of the Sajó River, so to the East of the road, they had built a 4,5 kilometers long, 100 meters wide, and 10 meters deep new riverbed. As a result of this investment, the West part of the site had to be excavated on 3580 m^2^ in 2014.

During the excavation, features were unearthed from the Neolithic Period (ALPC), Middle Bronze Age, Late Iron Age (La Téne), Roman Period (Germans), and Middle Ages. From the Neolithic Period, most settlement features had been unearthed. One of the most interesting ones was among them a well (stratigraphic number 76), which was made using a carved tree trunk. Only the lower carved pieces of the tree trunk, up to a height of 10 cm, have been preserved due to the constant groundwater. The well was at the West edge of the small hill, only a few meters from the postholes of the houses and long pits.

5 Neolithic burials were also found at the site, one of them was the number S3 individual buried 23 meters to the North from the well. The grave was separated by a greyish-brown, slightly oval-shaped patch from the light brown loam, with an obsidian splint on its surface. The grave pit was 163 cm long and 130 cm wide. The adultus-maturus (35-45 years old) male was in a sleeping position oriented to the Southeast facing to the South. North from the skeleton, just behind it was a conical, straight-walled bowl On it lay a fragment of a smaller vessel. At the pulled-up knees South from the body, there was an 8 cm long obsidian blade. There is a carbon 14 date from the wood of the well S76 (Supplementary Figure 12), which is cal BC 5211 – cal BC 4964. This part of the settlement may have existed in this interval[^36^](https://paperpile.com/c/2vFjDD/tQuB2).

- **I29958**: HUNG 954, grave s3, 5200-4900 BCE

**
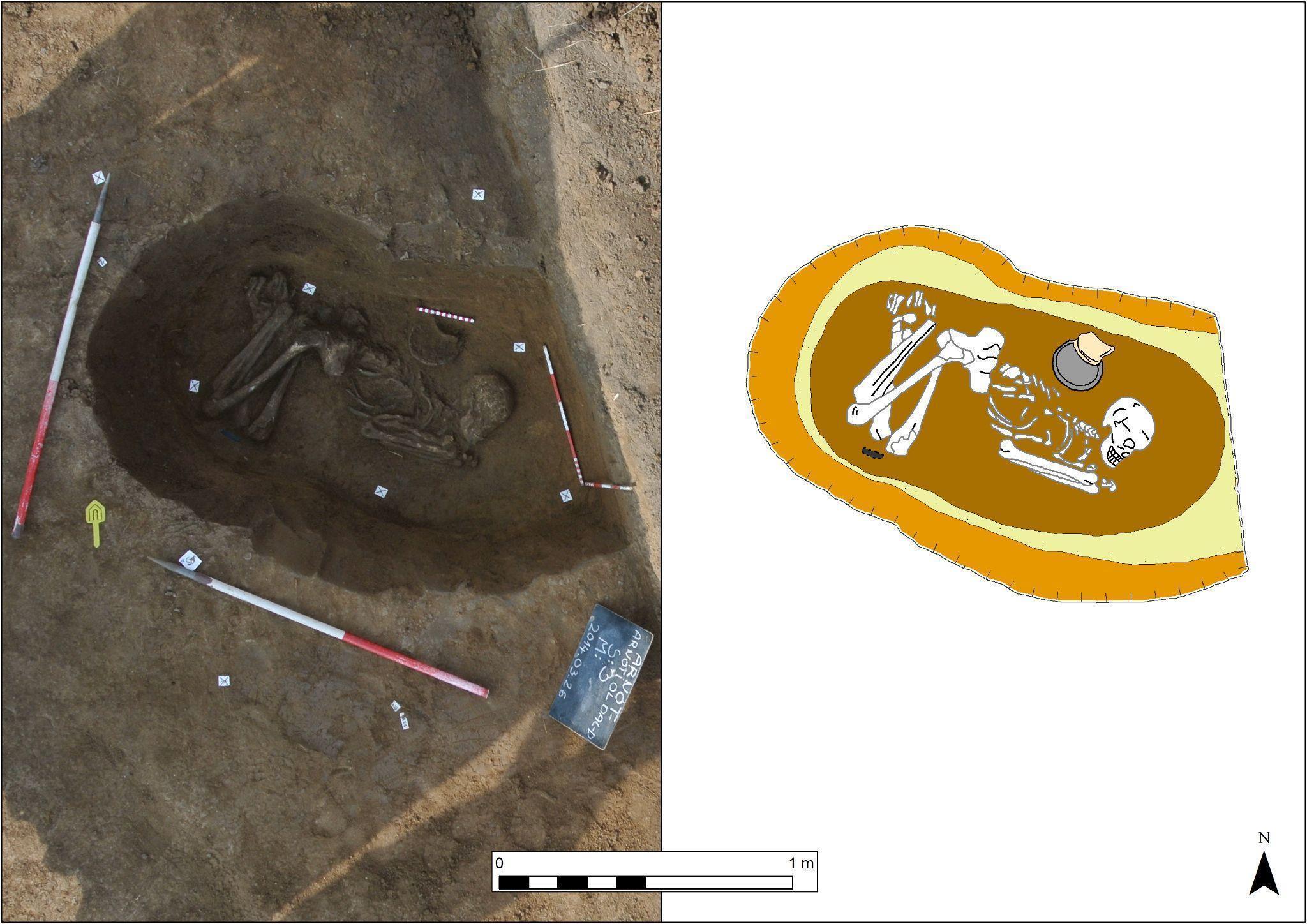
**

**Supplementary Figure 12:** Individual from Arnót – Arnóti-oldal Dél. Figure from Krisztián Tóth with rights of publication.

**1.14 Arnót –Nagy-bugyik (Hungary)**

**Author:** Krisztián Tóth

Nagy-bugyik site is situated in Borsod-Abaúj-Zemplén county, Northeastern Hungary, 3 kilometers from the Northeastern part of Miskolc city and 900 meters to the West of the North part of Arnót village. The Sajó river widens here and becomes more river branches. Geographically, this is the border between the Great Hungarian Lowland and the North Hungarian Mountains. At the edge of this swampy territory, river terraces were suitable for human activity, such as the 1,2 kilometers long one. The site is 900 meters to the North of Arnóti-oldal Dél site at the northern part of the terrace between two riverbeds of the Sajó River.

Large-scale rescue excavations were carried out in the connected building of the M30 motorway around Miskolc in 2014-2015. This bypassing part of the motorway is around Miskolc city leading through the swampy area mentioned above. Therefore, they had to build an embankment for the road, but this would have prevented the flood of the Sajó river, so to the East of the road, they had built a 4,5 kilometres long, 100 meters wide, and 10 meters deep new riverbed. This increased Arnót’s risk of flood. As a result of this investment, the West part of the site had to be excavated on the track of the embankment on 1810 m2 in 2014 and 2015. The section was 5 meters wide and 100 meters long.

During the excavation, features were unearthed from the Neolithic Period (ALPC), Copper Age, Roman Period (Germans), and Early Migration Period. From the oldest period, some more shape pits, a well (S9) and four burials had been discovered. In the case of the well, the traces of the former tree trunk were clear, but due to the low groundwater, no organic matter remained in it. The buried individuals, in every case, lay in sleeping positions, three of them were oriented to the Southeast. The grave (S17) of a 13-15-year-old child contained a grinding stone at the foot. One meter to the Northwest from this grave was the fourth burial (S18) oriented to the North differently from the others. During removing the topsoil, the poor conservated bones had been discovered in the yellowish loam without any sign of digging in. The 15-20-year-old individual lay on its right also differently from the others. The skeleton was 80 centimeters long. There is no carbon 14 date from this site from this period, but the features may be dated to the same period as in Arnót-oldal Dél site. [^37^](https://paperpile.com/c/2vFjDD/d8R8d)

- **I29957**: HUNG 953, grave s18, 5200-4900 BCE

**1.15 Tiszaszőlős-Domaháza (Hungary)**

**Author:** László Domboróczki

With the 2003 discovery of the Körös Culture settlement at Tiszaszőlős–Domaháza-puszta it became evident that the northern spread of the Körös Culture may not have halted in the middle of the Great Hungarian Plain but reached the Upper Tisza Region. The Tiszaszőlős–Domaháza-puszta site is located 40 km to the north of Szolnok, along the Tisza River. It was first occupied by Körös people (two pits and a house identified) and later settled during the ALPC period (Domboróczki 2010).

The small Körös culture settlement, extending no wider than 40 by 20 m, was established on the south bank section ca. 400 metes from the Tisza itself. It was found in 2003 as a result of a deliberate and carefully planned systematic search that began with a surface collection. Working in 1 metre-square units, an area of 50 by 200 m was surveyed. Then an excavation surface of 370 m2 was opened where the concentration of Körös-type finds was densest. In the exploratory trenches, the work was carried out in 1×1 m units in a manner similar to the surface collection, and during the course of removing 10–20 cm artificial layers, all the finds were counted according to different find types. Soil samples were taken from the excavated features, and almost all of the fill was wet-sieved. Besides the Bronze Age and medieval features, two pits (Pit 6 and 15) and a walking surface (Walking Level 1 that seems to be the remains of a house) were discovered in the excavation area that contained early neolithic Körös-culture material.

According to the find-density maps from the different levels of the excavation, Walking Level 1 was very regular in shape, forming an oblong of 12×5.5 m, with its long axis oriented NW–SE. It consisted of a thick layer of ceramic and mussel shell fragments, and the burnt remains of a hearth were found in the centre of the southern half. This walking surface can be interpreted as the site of a house, even though there were no traces of postholes observable on the level of the loessy subsoil, heavily disturbed by rodent activity.

The largest feature containing Körös finds was Pit 6. It measured 9×7.5 m and, similarly to the site of the house, was oriented with the long axis NW–SE. It yielded a tremendous amount of ceramic material and, similarly to the house, also contained large quantities of mussel shell, which here, however, constituted continuous layers within the fill. Pit 6 was almost 2 m deep and was uncovered in 15 artificial layers, each 10–20 cm in depth. Find material from the Körös culture was present in all layers. While in the lower layers, only Körös finds were recovered, in the upper layers Szatmár Group (ALPC) finds were also recovered. The superposition of the Körös and Szatmár is found in Pit 6, as well as hints at successive events. It seems that the pit was initially dug out and filled in by the people of the Körös culture. When the people of the Szatmár Group arrived at the site, the walking level containing their finds covered the area of the former Körös pit. Over time, however, the high organic content of the Körös pit subsided to such an extent that the finds were compressed, causing the later Szatmár walking level to become the uppermost level of the original Körös pit.

Evidence of the Szatmár walking level was also found north of Pit 6. This walking level (Walking Level 2) might have stretched as far as the area of Pit 15. Pit 15, unfortunately, was not completely excavated, so its exact shape is unknown. The 5×4 m part that was excavated had a semi-cylindrical form with vertical walls. It contained substantial numbers of Körös ceramic finds but very few mussel shell fragments. Moreover, it also yielded some Szatmár- as well as Szakálhát-type finds.

Out of the six human individuals found at the site, only one skeleton can be assigned securely to the Körös culture. This skeleton (Grave 2–3), was separated into two or three parts, probably by later disturbance. It was found in the lower layers (10–13) of Pit 6. Here the intrusion must have happened soon after the funeral since the conjoined body parts were seemingly moved away together, more or less keeping their anatomical order. Two other sets of human remains (Graves 4 and 5; only parts of bodies: a skull and a jaw) collected from this same pit also belonged to the Körös culture, as was proved by more recent 14 C testing. The skull fragment, thanks to different DNA analyses could be viewed as the remains of a Mesolithic person [^38,39^](https://paperpile.com/c/2vFjDD/BE1Yv+Yzi14). Some long bones, a skull fragment and phalanges (Grave 7) were found in the area of Walking Surface 1 (House 1), but in a very dispersed way. They may have belonged to a formal burial as well. One bone fragment from these was dated to the time of the ALPC. The most complete skeletons of the site are the ones found in graves 1 and 6 that were cut in Pit 6. One is in the eastern, and the other is in the northern rims of Pit 6. These belonged to regular burials, laid on their right sides in a contracted position, facing east. The human remains of the site were analysed by Zsuzsanna K. Zoffmann.

The animal bones from the site were examined by István Vörös, who discovered that the percentage of domesticated animals at the site was 44%, while the percentage of wild animals amounted to 34%. The remaining 22% of the animal bones came from fish, birds, and reptiles. The several thousand mussel shell fragments were analysed by Levente Fűköh, according to whom the leading type was the painter’s mussel (Unio pictorum) at 36.9% of the total, followed by the swollen river mussel (Unio tumidus) at 30.7%, and the thick-shelled river mussel (Unio Crassus) at 26.6%.

The analysis of more than one thousand carbonised seeds brought extraordinary results. According to Ferenc Gyulai, among the 38 different species, 70% were from cultivated plants, cereals, or legumes [^40^](https://paperpile.com/c/2vFjDD/XRWKE).

The stone material was meticulously analysed by Małgorzata Kaczanowska and Janusz K. Kozłowski [^41^](https://paperpile.com/c/2vFjDD/uIekL). They point out that obsidian dominated among the raw materials; microliths were most often used for tools, and blades were the most frequent form of flake [^42^](https://paperpile.com/c/2vFjDD/eiTJ1).

- **I21828**: grave 1, HUNG344, 5207-4786 calBCE (6040±60 BP, deb-10901)

40-46-year-old male in contracted position on his left side. The radiocarbon dating shows that it belongs to the late ALPC period (Supplementary Figure 13).


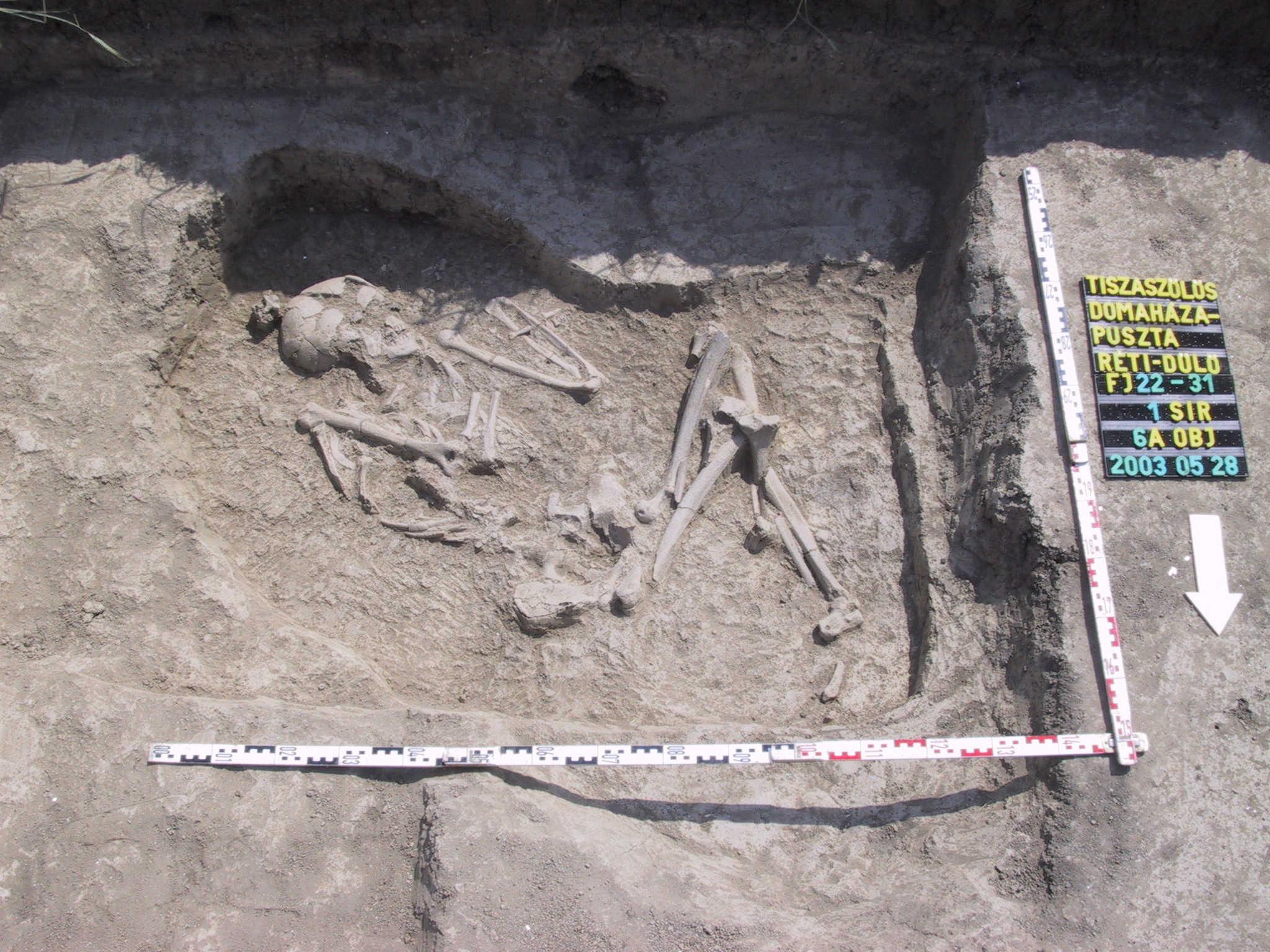


**Supplementary Figure 13:** Grave 1 Tiszaszőlős-Domaháza. Image with permission of publication.

- **I21830**: grave 6, 5211-4788 calBCE (6060±80 BP, deb-11084)

34-40-year-old male in contracted position on his left side and dated to the late ALPC period (Supplementary Figure 14).

**
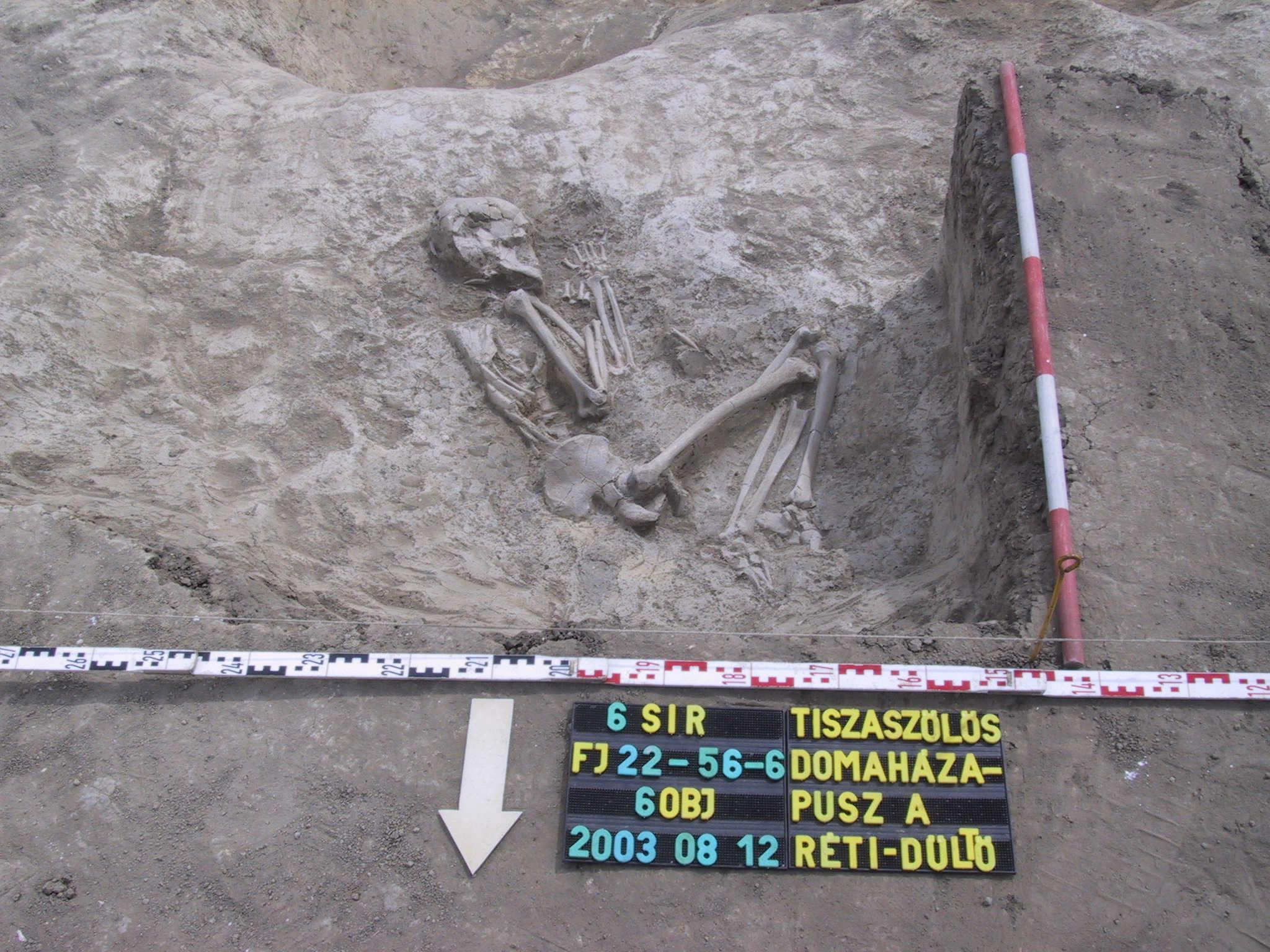
**

**Supplementary Figure 14:** grave 6 of Tiszaszőlős-Domaháza. Image with permission of publication.

**1.16 Füzesabony-Gubakút**

**Author:** László Domboróczki

The Füzesabony-Gubakút site is situated at the northernmost edge of the Great Hungarian Plain, approximately 3 km south-west of the modern village of Füzesabony and about 10 km to the south of the Mátra-Bükk foothills. The area of the ALPC settlement is separated into two parts by a broad but shallow valley that was cut by a one-time riverbed into late Pleistocene sediment. The site was rescue-excavated between 1995 and 1996 before motorway construction.

The most significant result of this excavation was the discovery of an idiosyncratic settlement layout that has been documented since that time at other ALPC sites [^43^](https://paperpile.com/c/2vFjDD/g2IKR). The evidence of triple-partitioned houses, 12–16 m long by 5–6 m wide, and the regular settlement structure here contradicted earlier views of small, irregular ALPC settlements made up principally of pit dwellings. In Füzesabony-Gubakút, the houses were arranged in rows on either side of a stream. Between the houses there were pits, and at the corners of the houses, there were human burials. A total of 13 graves were found on the settlement, all left-crouched and orientated with the head to the southeast or east. All of them were analysed by Zsuzsanna K. Zoffmann from an anthropological, and later by others from osteological and pathological perspectives in the Lifeways Project [^44^](https://paperpile.com/c/2vFjDD/YuzSY). Seven graves were furnished with grave goods that were, except for one pot, entirely Spondylus ornamentation.

The topography of Füzesabony-Gubakút and the many radiocarbon dates obtained have presented an excellent opportunity to examine the chronology of the site and its internal development. Informal inspection of the radiocarbon results (based on the animal bone from the pits) has suggested 12 settlement phases, each lasting 30–50 years, between 5560–5000 calBCE [^45,46^](https://paperpile.com/c/2vFjDD/kyKzi+d19B6). This was supported by seriation based on 74,580 sherds derived from 18 of the 28 Neolithic pits found at the site. House-pit-grave ensembles could be identified as household areas and assigned absolute time limits. Using surface collection data gained in 2007, the whole settlement area could be modelled. The working hypothesis was that the settlement began with the appearance of a pioneer family at the site around 5560 calBCE and, through a continuous history as well as dynamic growth, reached its peak with 12–14 contemporaneous houses/families between 5325–5220 calBCE.

Around Füzesabony, an interesting network of ALPC settlements has been recorded, which consisted of regularly spaced larger settlements similar to Gubakút, surrounded by smaller settlements in their close vicinity, echoing the model of larger, central/mother settlements, with smaller, satellite/daughter sites around them proposed elsewhere for the ALPC distribution. The large settlements were aligned along ancient riverbeds and located at regular intervals of some 2 km from one another. By calculating house numbers and possible population levels and estimating the size of herds and the scale of meat consumption, it was concluded that circles of 1 km diameter probably met the requirements for the pasturage necessary for grazing 15 cattle and 30 sheep per household in a settlement of 14 families. This large land requirement suggests that the extent of the pasturage would have been the greatest determining factor in the sustainability of a settlement during this phase of the Neolithic. Building on this model, it was suggested that inter-settlement relationships may have also influenced the demographic development of the region and its settlement pattern, and the individual central settlements — such as Füzesabony-Gubakút — represented different lineages or groups. Modelling the process of settlement development at different scales (by analysing different patterns and using terms such as local development, in and outward migrations, demographic pressure, etc.) it was concluded that Neolithization may have proceeded along similar principles for hundreds of years in the region.

- **I10351**: grave 5, 5370–5200 calBCE (6295±40, VERA-4242)

A 37-46-year-old male in contracted position on the left side wearing large spondylus beads around the neck. The anthropological material consisted of a fragmentary and incompletely preserved skull and skeletal bones.

- **I10352**: grave 3, 5211-4788 calBCE (6060±80 BP, deb-11084)

A 31-40-year-old female in contracted position on the left side wearing small spondylus beads on the head. The very fragmented and incomplete anthropological find was barely suitable for a more detailed analysis.

- **I10353**: grave 2, 5470-4955 calBCE (6250±90 BP, deb-11092)

A 14-16 year-old child in contracted position on the left side. The find consists of the fragments of the skull and the skeletal bones. The age at death was determined by the teeth and the ossification of the epiphysis joints of the long bones.

- **I10355**: grave 1, 5359-5210 calBCE (6295±35 BP, VERA-4237)

A 20-22-year-old female, in contracted position on the left side. The find consists of the fragments of the skull and the skeletal bones, which were unsuitable for a detailed analysis.

- **I10349**: grave 10, 5292-5046 calBCE (6200±30 BP, PSUAMS-10194)

52-61-year-old male in contracted position on the left side the only relatively well-preserved find in the series from the site.

- **I10350**: grave 4, 5316-5129 calBCE (6269±27 BP) [R_Combine: (6275±35 BP, PSUAMS-13111), (6260±40 BP, VERA-3055)]

25-34-year-old female in contracted position on the left side wearing small spondylus beads on the head and around the neck. The anthropological material was very fragmented and incomplete, so no detailed analysis could be made.

**1.17 Polgár-Ferenci-hát (Hungary)**

**Author:** Alexandra Anders

The ALPC site of Polgár-Ferenci-hát is located east of the Tisza River, to the north of the great Hungarian Plain, and was first studied in 2004 in a rescue excavation. The site shows two different stages of occupation. The first phase is ALPC I (Szatmár), with grave 718 being a remarkable specimen with a particular grave differentiated from the rest [^47^](https://paperpile.com/c/2vFjDD/1ByVo). The second occupation corresponds to phases II-IV of the ALPC. A total of 113 graves have been excavated, with radiocarbon dates spanning from 5300 to 5070 calBCE. The graves are distributed in small clusters. Previous dietary Carbon/Nitrogen isotope analyses [^44^](https://paperpile.com/c/2vFjDD/YuzSY) revealed no differences between males and females and no correlation to grave goods.

The Neolithic settlement of Polgár-Ferenci-hát lines in the Polgár Island micro-region within the Upper Tisza region. This site offers a unique possibility for tracing the cultural changes in the later sixth millennium BCE, as well as the local dynamics of the internal transformation of the Alföld Linear Pottery (ALPC) of the Middle Neolithic.

As previously mentioned, there are two occupational phases in Polgár-Ferenci-hát: The first can be dated to the earliest phase of the ALPC (The ALPC-I: 5467 and 5344 calBCE). During this period, the settlement had a rather dispersed layout, with six burials and some smaller and larger pits lying quite far from each other. The second period (ALPC II-IV: 5293–5068 calBCE), this phase corresponds with a period of more intensive usage of the site.

The accumulation of several layers in this central part of the settlement is indicative of the type of stratigraphic events that led to the emergence of tell sites in the southern section of the Great Hungarian Plain, near Szakálhát and Esztár type settlements. Outside the enclosure, traces of a dispersed horizontal settlement were found. With an overall horizontal extent of 9-12 ha and a vertical accumulation of strata in its centre, the settlement of Polgár-Ferenci-hát represents a dualistic site-formation process.

Characteristic sherd types found within the closed artifactual assemblages related to the circular ditch system recovered at Polgár-Ferenci-hát are indicative of a synthesis between the Tiszadob-Bükk and Esztár-Szakálhát-Vinča ceramic styles. Previously, late stylistic groups of Alföld LBK pottery were often classified into different chronological phases. Nevertheless, distinctly synchronous occurrences recorded at this site raise the need to reconsider the relative chronology of these styles seriously. The ALPC II–IV occupation at Ferenci-hát can be dated between 5293–5068 cal BC.

In addition to settlement features, 116 graves were excavated. These graves represent the largest number of ALPC burials in the Tisza River region. The most outstanding burial of Phase I was Feature 718. A woman of 22–28 years of age was laid to rest here on her left side, in a crouched position. She wore a string of Spondylus shell beads around her neck. She was buried with six regular and five miniature vessels and an anthropo-zoomorphic figurine.

The graves of ALPC II–IV Phase were clustered in groups of 3–10 smaller and greater concentrations of burials within the excavated area. The grave pits were oval or trapezoid. Sometimes the deceased was not placed in an ordinary grave pit but in a storage pit. Thanks to the water-logged deeper strata, traces of a coffin or a wooden bier could also be observed for the first time in the history of the Middle Neolithic in Hungary.

The most common orientation was SE to NW, with only minor deviations. With only three exceptions, the deceased was laid on their left sides in a more-or-less crouched position. The majority of the deceased were placed in the grave alone. However, two double burials were observed as well. In four cases, the deceased were buried without their heads. Two of these people were buried in proper graves, while the other two were placed in storage pits. All four of them were men of mature adult age. According to studies by Zsuzsanna Zoffmann (physical anthropologist), no cut marks were present on the remaining bones, that is, the deceased was not decapitated, but their skulls were removed sometime after burial. Two scattered-ash burials are of special significance. Based on previous research, evidence of cremation is known only from the Late Neolithic in Hungary.

In almost 10% of the graves, traces of ochre were found under the skull and the bones of the leg. Eighty-two of the 113 excavated graves contained no grave goods. A total of 16 burials contained vessels. They usually occurred as single finds, although some of the deceased were buried with two or even three vessels. A special group of burials with vessels is represented by three graves in which vessels full of ochre were placed into the grave pit. Another group of important grave goods is represented by stone tools, although only six graves contained obsidian blades or ground stone chisels, which occurred in the burials of women, men, and children alike. Two additional burials deserve special attention. Both of them contained a large obsidian core (exceeding 10 cm in length) placed near the head of the deceased. There were also signs of secondary, post-burial placement of grave goods (Nachgaben). In these cases, pottery sherds were discovered at the end of the grave pit, but well above the skeleton. A total of 24 neonate and child burials were excavated at the site. Pots and jewellery had been placed in the graves of 12 children. The differential distribution of Spondylus objects is perhaps the most pronounced difference between adult and child burials. A total of 38 such ornaments came to light from seven burials and comprised large beads, necklaces and bracelets strung of smaller beads, and arm rings. Of these, only one bead was recovered from the burial of an adult male; the other six graves contained the burials of newborn infants and children aged less than 6-7 years.

Overall, the burial rite observed at the site of Polgár–Ferenci-hát was similar to the general mortuary behaviour of the ALPC groups, and largely similar care was taken of the deceased within the broader distribution area of the LBK culture.

- **I21826**: grave 821, 5350-5000 BCE
- **I21827**: Grave 890, 5500-4750 BCE
- **I21840**: Grave 342, 5500-4750 BCE
- **I21842**: Grave 134, 5500-4750 BCE
- **I21843**: Grave 126, 5400-5000 BCE
- **I21844**: Grave 4,5500-4750 BCE
- **I21847**: Grave 341, 5213-5008 calBCE (6165±35 BP, VERA-4333)
- **I17909**: Grave 786, 5500-4750 BCE
- **I17911**: Grave 719, 5474-5312 calBCE (6400±30 BP, PSUAMS-14099)
- **I18656**: Grave 691, 5500-4750 BCE
- **I18658**: Grave 353, 5500-4800 BCE
- **I18659**: Grave 352, 5500-4750 BCE
- **I18661**: Grave 768, 5500-4750 BCE
- **I18696**: Grave 897, 5290-5006 calBCE (6185±40 BP, VERA-4338)
- **I18657**:Grave 773, 5310-5071 calBCE (6250±35 BP, VERA-3058)
- **I17950:** Grave 338, 5350-4950 BCE
- **I21898:** Grave 721, 5467-5219 calBCE (6355±30 BP, PSUAMS-10203)
- **I18660:** Grave 718, 5474-5315 calBCE (6405±30 BP, PSUAMS-10201)
- **I18695:** Grave 904, 5500-4750 BCE
- **I21902:** Grave 644, 5371-5216 calBCE (6330±30 BP, PSUAMS-10204)
- **I21899:** Grave 448, 5500-4750 BCE
- **I21901:** Grave 34, 5400-5000 BCE
- **I21763**: Grave 34, 5500-4750 BCE
- **I21765**: Grave 144, 5500-4750 BCE
- **I21766:** Grave 283, 5500-4750 BCE
- **I21767:** Grave 288, 5500-4750 BCE
- **I21768:** Grave 348, 5500-4750 BCE
- **I21769:** Grave 783, 5500-4750 BCE
- **I21771:** Grave 822, 5500-4750 BCE
- **I21846:** Grave 900, 5500-4750 BCE
- **I21714:** Grave 31, 5210-5008 calBCE (6155±30 BP, PSUAMS-14101)
- **I23093:** 468, grave 296, 5500-4750 BCE
- **I23094:** Grave 34, 5299-5060 calBCE (6220±25 BP, PSUAMS-14110)
- **I21897**: Grave 782, 5296-5047 calBCE (6205±30 BP, PSUAMS-14720)
- **I21770:** Grave 801, 5045-4842 calBCE (6050±35 BP, PSUAMS-10207)
- **I21841**: Grave 103, 5316-5069 calBCE (6260±40 BP, VERA-4331)
- **I17910**: Grave 815, 5500-4750 BCE
- **I18662**: Grave 356, 5500-4750 BCE
- **I21824**: Grave 807, 5500-4750 BCE
- **I21825**: Grave 867, 5306-5061 calBCE (6235±35 BP, VERA-3056)
- **I21772**: Grave 871, 5500-4750 BCE
- **I21773**: Grave 881, 5500-4750 BCE

**1.18 Rákóczifalva–Bagi-földek sites 5, 8 and 8a (Hungary)**

**Author**: Katalin Sebők.

This chain of sites is situated on a somewhat bluff-like, flood-free bank of a one-time bed of the river Tisza southwest of Rákóczifalva in Central Hungary. Today, the river runs west of the sites in a bed where it was confined by water regulation in the 19th century. As part of creating a reservoir, extensive preventive excavations were carried out in the path of a new embankment, cutting through the top of the one-time flood-free bank and, thus, the highest points in the area, between 2005 and 2007. All three sites were excavated by two teams from the Eötvös Loránd University, Budapest, with Katalin Kovács, Katalin Sebők, Gábor Szabó, and Gábor Váczi.

Not surprisingly, the plateaus and high slopes of the bank’s sandhill row were used in multiple historical periods, including the Middle and Late Neolithic, the Early Copper Age, the Late Bronze Age, as well as the Early Roman Imperial (Sarmatians), Migration (Gepids and Avars), Hungarian Conquest, Late Mediaeval, and Modern Periods.

The sampled sites line up next to each other. Site 8 lies at the westernmost end of the string of hills next to the current riverbed, and probably in the immediate vicinity of the river at the time of the Neolithic and Copper Age occupations. East of Site 7, separated by only a slight depression, lies Site 8A, stretching on the highest point of the area. The eastern slope of site 8A concludes in a rather extended but less densely occupied site, no. 5, stretched on a less prominent but still high plateau consisting of several lesser elevations.

The three sites are, in fact, part of the same inhabitations in more than one period is manifest in that the features associated with any horizon do not necessarily cease at the (arbitrarily determined) border but continue into the “next” site. Thus, Neolithic and Szakálhát features were scattered on all three sites, while the traces of settlements in the Early Copper Age were found in sites 8 and 8a.

The cultural features determined as Neolithic and Szakálhát probably belong to the same settlement. This record has not been evaluated and processed yet; therefore, the following numbers cannot be regarded as final. On the three sites (5/8A/8), altogether, 225 features (73/104/48), comprising about 30 (3/22/5) inhumation burials, belonged to this era. There is no available radiocarbon data for these features.

All burials seemed to follow the same funerary rite: the dead were laid in a narrow oval grave pit or inside a larger pit complex in a crouched position on either side. Persisting grave goods were scarce and included mainly knapped flint (and, occasionally, obsidian) tools and, rarely – probably for women– necklaces with a large cylindrical limestone or spondylus bead as a centrepiece. Several burials did not contain any grave goods.

All the individuals from this site are attributed to Szakálhát. All the individuals were buried in rounded, individual grave pits, either dug for them or turned into one. No individuals were buried with the material.

**Rákóczifalva-Bivaly-Tó-5**

- **I17941**: 3646 (HUNG570; Site 5, 272, 41825, 373), 4991-4803 calBCE (6010±25 BP, PSUAMS-14125)
- **I17933**: 3638 (HUNG571; Site 5, 340, 41826, 450), 5050-4750 BCE
- **I17938**: 3643 (HUNG569; Site 5, 404, 41830, 504), 5302-5047 calBCE (6215±35 BP, PSUAMS-15079)
- **I17939**: 3644 (HUNG568; Site 5 351, 41828, 461), 5050-4750 BCE

**Rákóczifalva-Bivaly-Tó-8**

- **I17934**: 3639 (HUNG564; Site 8, 41, 41855, 93), 5250-4950 BCE
- **I18635**: 3646 (HUNG570; Site 5, 272, 41825, 373), 5306-5056 calBCE (6230±35 BP, PSUAMS-15081)
- **I18636**: HUNG563 (grave 249), 5300-5000 BCE
- **I18637**: HUNG565 (grave 136), 5300-5000 BCE

**Rákóczifalva–Bagi-földek Site 8/A**

- **I29883**: HUNG501 (2014.9.115, 690), 5209-5005 calBCE (6150±30 BP, PSUAMS-11430)
- **I29884**: HUNG504 (2014.9.73, 579), 5250-4950 BCE
- **I29885**: HUNG505 (2014.9.234, 718), 5250-4950 BCE
- **I17926**: HUNG507 (grave 754 (2014.9.222), 5250-4950 BCE
- **I18640**: 3652 (HUNG510, 622, 2014.9.85, 747),5250-4950 BCE
- **I18641**: 3653 (HUNG502, 561, 2014.9.68, 670), 5250-4950 BCE
- **I17936:** 3641 (HUNG506, 790, 2014.9.153, 923), 5250-4950 BCE
- **I17937**: 3642 (HUNG508, 849, 2014.9.169, 988), 5250-4950 BCE
- **I17935:** grave 803 (HUNG500, 2014.9.156), 5250-4950 BCE
- **I18639:** 777 (HUNG503), 5250-4950 BCE
- **I17940**: 3645 (HUNG566, 87, 41872, 138), 5250-4950 BCE

**1.19 Furta–Töviskés (Hungary)**

**Author:** László Szolnoki

The site located on the outskirts of Furta, in Hajdú-Bihar county, was excavated by László Szolnoki (Déri Museum) in the autumn of 2010 in the course of a rescue excavation. On the site, 25 features were excavated from the Alföld Linear Pottery culture, one of which was a burial.

- **I17362**: grave 102, SNR146, 5329-5211 calBCE (6295±30 BP, PSUAMS-14995)

Inhumation burial of a female in a shallow burial pit, in the humus. The deceased was lying on her left side in a strongly contracted position. The arms and legs were strongly bent. Bones are in good preservation. The skeleton was oriented from E to W. On both wrists of the deceased, there was a bracelet made of beads.

**1.20 Berettyóújfalu-Nagy-Bócs-dűlő 2**

**Author**: János Dani

The site of Nagy Bócs-dűlő was excavated during 4 campaigns (2004, 2005, 2008, 2011). The preventive excavations were associated with the construction of a regional waste treatment plant. A total area of more than 9.2 ha was excavated. This multi-period site (with 10 periods from the Early Neolithic until the Middle Ages) – is located in the SW part of the town Berettyóújfalu (part Berettyószentmárton) on the bank of the earlier riverbed (meander) of the Berettyó river. We sampled individual HUNG279 that was determined to be Neolithic according to the body position; radiocarbon dating confirmed a Middle Neolithic attribution.

- **I17947:** HUNG279, 5359-5212 calBCE (6300±35 BP, PSUAMS-15080)

**1.21 Debrecen Tócópart Erdőalja (Hungary)**

**Authors:** Emese Gyöngyvér Nagy, Zsigmond Hajdú

The site is situated in the eastern part of Hungary, in the Great Hungarian Plain (Hajdú-Bihar County), near Debrecen. In 2008–2009, based on a contract with the Cultural Heritage Service, the Déri Museum carried out preventive excavations on a section of Route 4 bypassing Debrecen. The NE–SW oriented site also yielded remains from 11 other periods from the Middle Neolithic (ALPC and Esztár group). During the excavation in 2008, four ALPC graves were unearthed. Three had no grave goods at all, while the fourth contained, in addition to vessels and limestone beads, traces of ochre as well. In 2009, in the area north of the railway, we excavated six ALPC inhumation burials, six postholes that probably belong to this period, a well, and 25 pits. Four of the pits could only be partly excavated due to their large size. In the southern part of the site, beyond the railway, excavation was hindered by the fact that the Middle Neolithic (Esztár group), Late Iron Age (Celtic), Sarmatian, Árpád Period and Late Medieval settlements spatially overlapped: there were many superpositions even within a single period. The Esztár group was represented by a well, two inhumation burials, and 23 huge clay extraction pits.

According to the physical anthropological study, the Neolithic skeletal material is rather heterogeneous and is in a bad state of preservation, so it is unsuitable for generalizations and group identification.

- **I17365:** HUNG305, Feature 353, 5350-5000 BCE.

Burial of a male lying on his left side, in a strongly contracted position, excavated in 2008. The skeleton was incomplete and oriented southeast to northwest 146˚ with hyper-flexed legs. The grave was disturbed by a Sarmatian circular ditch. A pottery grave good was situated beside the legs. Other grave goods include traces of ochre under the skull and four large limestone beads.

- **I17948:** HUNG305, Feature 930, 5299-5052 calBCE (6215±30 BP, PSUAMS-14998)

Burial of an adult male lying on his left side, in a strongly contracted position, excavated in 2009. The skeleton was incomplete and oriented southeast to northwest 108˚ with hyper-flexed legs without grave goods.

**1.22 Ebes-Zsong-völgy (Hungary)**

**Author:** János Dani

Rescue excavation with limited information. Based on the anthropological examination, the deceased was a 43-60 year old (mature) woman. The burial represents the local, late phase of the ALPC (Esztár group).

- **I17363**: HUNG274, Feature 1412, 5305-5064 calBCE (6235±30 BP, PSUAMS-14996)

**1.23 Debrecen-Tesco (Mikepércsi street Sports field) (Hungary)**

**Author**: Márta Szelekovszky

Excavation of a single burial, the grave represents the late phase of the ALPC (Esztár group).

- **I17366**: HUNG289, feature 286, 5321-5127 calBCE (6275±30 BP, PSUAMS-14997)

This burial is of a female estimated to be 40-60 years old. The bone material is in quite fragmented and incomplete condition. Her arms were probably bent at the elbows, hands in front of her face. In front of the cranium was a fragmentary red-coloured, hemispherical vessel with knobs under the rim. The body was positioned in the ESE-WNW 102° direction (Supplementary Figure 15).


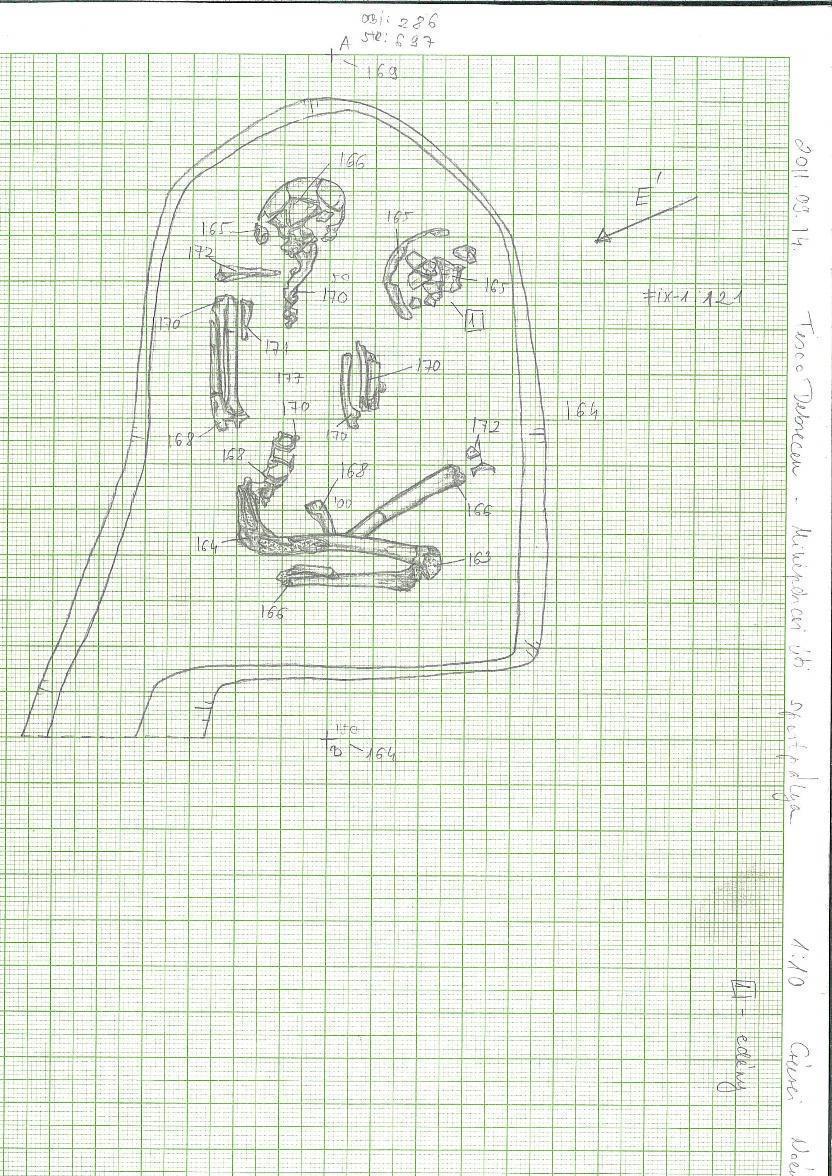


**Supplementary Figure 15:** the burial represents the local, late phase of the ALPC (Esztár group). Image from Márta Szelekovszky with permission of publication.

**1.24 Vonyarcvashegy-Mandulás lakópart III. ütem site**

**Author:** Havasi Bálint:

During rescue excavations, 48 settlement features (post holes, houses, pits) and one Neolithic grave were found. The settlement objects came from the Neolithic and Roman periods. In the trench of the Neolithic settlement, a child lying on his left side, without grave goods, oriented east-west (feature 71) was excavated. The width of the grave was 100 cm, and its length was 155 cm. Our new radiocarbon date confirms the LBK assignment.

- **I29891**: HUNG 680, 33/A, 5208-4907 calBCE (6100±30 BP, PSUAMS-15000)

**1.25 Apc-Berekalja (Hungary)**

**Author:** László Domboróczki

The site is situated north of Hatvan, along road no. 21. It is primarily a late LBK site, but the earliest phase is also present here. The uniqueness of the site lies in the fact that it sits at the easternmost periphery and is still one of the largest settlements of the LBK in the territory of Hungary. Several houses and pits were excavated here, and some graves were too (Supplementary Figure 16).

Although – based on a surface find collection – the site was registered as early as 2002, intensive research and partial exposure of the archaeological site only began later: in 2008-2009, thanks to lane widening on road 21. This excavation was led by József Danyi with the assistance of the present author. The earthworks affected the archaeological site on both sides of Road 21. Along the west side of the road, an opening of about 550x30 m in size became necessary, while on the east side of the road, a strip of 550x4 m was examined.

In the excavation area, the density of archaeological features was not uniformly thick. On the northern 140 m long section of the excavation area, the settlement features were only sporadic, then there was a 120 m long, almost artefact-free zone. Here the humus layer thickness increased significantly beyond the average 90-100 cm. As it turned out, this back swamp area might also have been the bed of a river, which probably functioned as an active channel during prehistoric times since prehistoric settlement features did not stretch over this zone. To the south of this assumed river bed, a long, very densely covered area 290 m in length stood out. It was there, beside the dominant Neolithic settlement features (houses and pits), that late Copper Age Baden, early Bronze Age Makó, and late Bronze Age Kyjatice pits also appeared, and where some additional independent graves of the late Neolithic Lengyel, late Copper Age Baden, middle Bronze Age Füzesabony and Migration Period Sarmatian cultures were also found. It must be mentioned that in 2014, the road building was to continue to the south, and from the southern end of the 2009 section, another 170 m long site section was identified during the excavation conducted by Mónika Gutay. Based on the c. 720 m long north-south section uncovered so far, one can estimate that the LBK site was at least 20 to 30 ha in size. This would mean that we have only excavated 6-10% of the total settlement area. Although the process of their scientific evaluation is far from over, the remains of 36 post-framed houses were identified here. The excavation of the house areas was carried out with utmost care, and entire surfaces were orthophotographed. The features of the excavation, from the observation of the patches, through their partial digging up, right up until their final unearthing, were very well documented. Perhaps uniquely in the case of the LBK period, even surfaces of find densities suggestive of the one-time walking levels were observed and documented. At Apc-Bereklja, based on the existing house plans, at least five settlement rows can be postulated. As for the length of the settlement rows at Apc, estimations cannot be made for the time being, as we do not know exactly where the western and eastern fringes of the settlement were [^48^](https://paperpile.com/c/2vFjDD/3lZ73)

On the evidence of pottery, it turned out that before the Music-note and Želiezovce phases, even the early Bicske-Bíňa phase of the LBK was present at the site. Several features of the settlement attested to the presence of those early LBK find materials that were represented by artistic ornaments, buttons, finger-printed edges, carved and polished decorations and typical vessel forms that are most reminiscent of material from the Starčevo culture, yet find their closest parallels in the published finds at Bicske-Galagonyás, Budapest-Aranyhegyi út, Bíňa and Brunn-Wolfholz. While the early Bicske-Bíňa types of the LBK were only sporadically observed in closed-find associations, the later music-note (Notenkopf) motif pottery and the latest Želiezovce-type ceramics were found nearly everywhere at the site. The clusters of C14 data confirm the main ceramic phases of the LBK settlement that existed in the period between 5470-4950 calBCE. Of the currently available 30 age data of the LBK settlement, which have an average ± 20 to 40-year deviance, six fall into the interval between 6424-6388 calBCE. These are very early dates and can indeed be considered as being among the earliest LBK dates in the entire territory it covers.

A total of 14 human skeletons and several dispersed bones were examined and described by Zsuzsanna K. Zoffmann in 2013 using classical methods. From the period of the LBK, three burials have been examined: two adult (a male and a female) skeletons and a child skeleton. The two adults buried in the LBK graves fit well typologically with the population of other sites.

The chipped and polished stone tools were examined and processed by Małgorzata Kaczanowska, Janusz K. Kozłowski, and Michał Wasilewski. The Neolithic LBK features at Apc provided 408 chipped stone artefacts. As excavations confirmed the multi-phase evolution of the site from the pre-Notenkopf to the Želiezovce phases in Apc, the local evolution of the LBK chipped stone industry could have been studied. In comparison with other LBK sites in Northwestern Hungary and Southwestern Slovakia, the series of lithic artefacts from Apc seems large. However, in proportion to the excavated area and the fact that all the phases of the LBK are represented, the number of artefacts per phase and habitation unit is small. The general inventory structure indicates that a specific, small-scale, local lithic production was conducted on the site. The role of blades was minor, while flakes were more important.


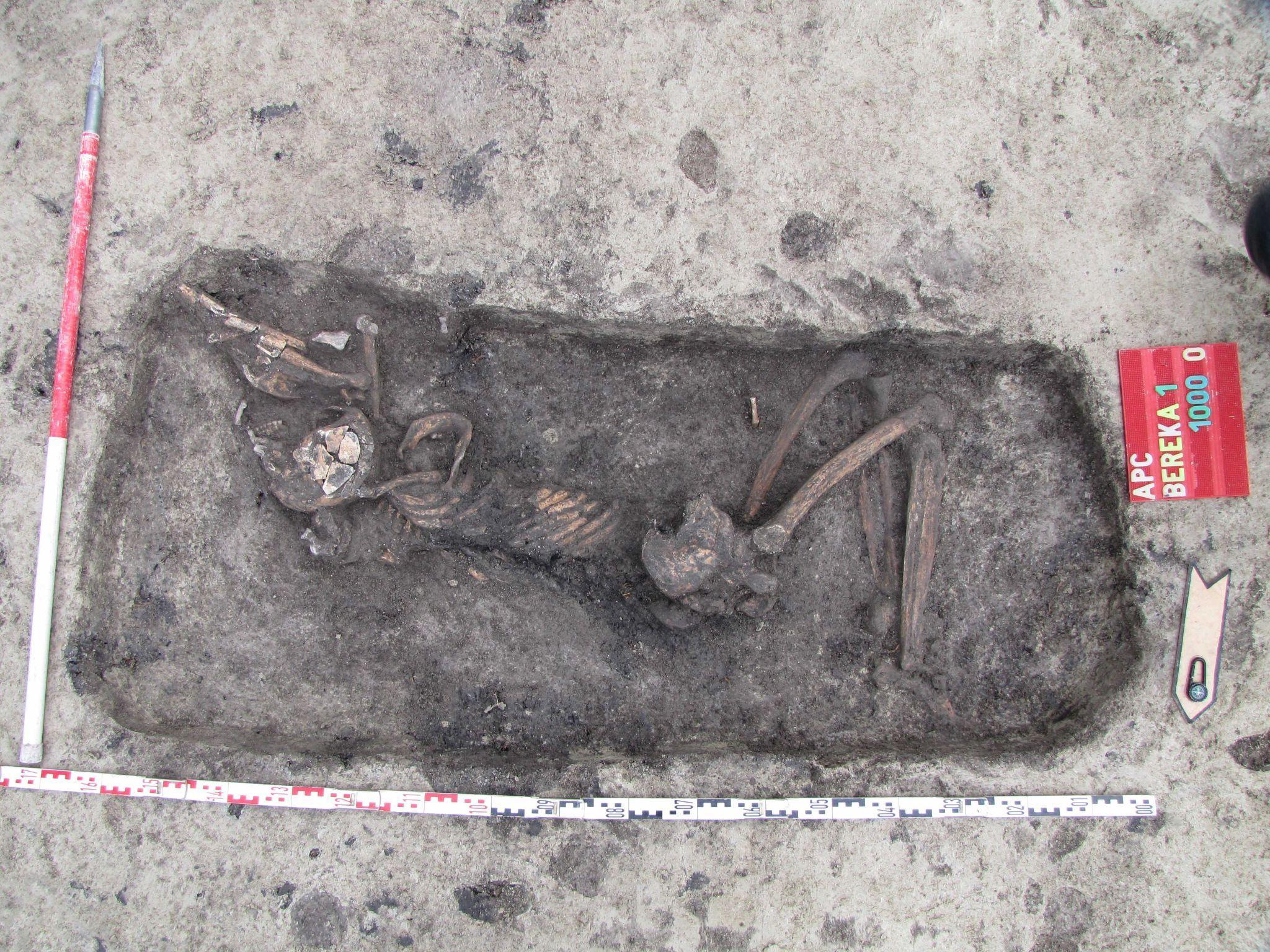


**Supplementary Figure 16:** Grave 12 from Apc-Berekalja site. Image with permission of publication.

The animal bones were identified and processed by László Daróczi-Szabó. From the total of 4,169 animal remains found at the site, 3,317 were successfully dated, coming from five different ages. The largest part of the animal remains a total of 2,254 fragments, came from the Neolithic period, from which 2,232 could be identified at least on a level of biological order. 95.6% (2,131 fragments) of these originate from domestic mammals: cattle (1,578 fragments, 70.6% of the identified remains), sheep and/or goat (385, 17.3%), domestic pig (163, 7.3%), and dog (5, 0.2%).

A total of 69 soil samples from the LBK features (813.5 l volume) have been collected during the course of the excavation and wet sieved at the State Office for Cultural Heritage Preservation Hesse (LfDH) (Wiesbaden, Germany). The archaeobotanical investigations are carried out by Péter Pomázi and Angela Kreuz. All in all, 17,962 charred and 1,661 mineralised plant remains from 46 plant taxa have been identified and archived in Wiesbaden under project number HU7 APC [^49^](https://paperpile.com/c/2vFjDD/MfY1v)

- **I21829**: HUNG359, grave 12, 5463-5216 calBCE (6341±33 BP, MAMS-14823)

53-59-year-old male in contracted position on the left side. The degree of contraction was not as extreme as with others from this time, as the legs were not lifted up above the hip.

**1.26 Kleinhadersdorf (Austria)**

**Author:** Maria Teschler-Nicola

The site is in KG Kleinhadersdorf, cadastral section Machleiten, not far from the municipality of Poysdorf in the north-east of Lower Austria, 70 km north of Vienna and approx. 15 km south of the border with the Czech Republic. Geomorphologically, it is a flat, wavy basin landscape with very fertile loess soils. Several Linear Pottery (LBK) sites are known here on both sides of the Poybach, often only documented by surface finds. These formed a small settlement cluster and thus correspond to the settlement pattern known from the LBK. The relationship of these settlements to the cemetery located somewhat higher and away from the settlements has hardly been researched and is unclear. The necropolis of Kleinhadersdorf, although only partially archaeologically explored, is considered the largest Early Neolithic/Linear Pottery burial site in Austria to date and probably originally comprised well over a hundred graves; however, many of the finds and features close to the surface were destroyed due to the hillside location and the agricultural use of the fields as vineyards.

Numerous people, collectors and local historians, as well as experts, and institutions, were involved in researching the Kleinhadersdorf burial ground. The first reports of finds by local historian Viktor Kudernatsch date back to 1911, 1924, and 1926. The human skeletal remains he mentioned from four inhumation graves were destroyed by fieldwork and have not been preserved. In 1931, the prehistorian custodian Josef Bayer, was informed that further graves had been found in this area during the trenching. He then carried out the first systematic excavation (7 graves were recovered). During a second expedition in the same year, a further six graves were finally surveyed [^50^](https://paperpile.com/c/2vFjDD/C68e8). Bayer compiled brief descriptions, location sketches, and high-quality photographs [^51^](https://paperpile.com/c/2vFjDD/GpA1g), from which reliable information about the burial rite, in particular the position of the corpses and grave goods, can be derived today. After Bayer's unexpectedly early death, Viktor Lebzelter completed this excavation in August 1931, albeit with less precise documentation than his forerunner. The results of the analysis of the human skeletal remains were presented in 1936 [^52^](https://paperpile.com/c/2vFjDD/gkfax). This study was carried out in line with the research concepts and focal points of the time, using a racial typological approach that is obsolete today. Mention is also made of the gracility of the skeletal remains and pathological changes, but not all former diagnoses could be verified in the new edition [^53^](https://paperpile.com/c/2vFjDD/xTdTJ).

It was only after a vineyard had been destroyed by frost in the winter of 1986/87 and was to be replanted, knowing that the graves already uncovered in the 1930s were very shallow and that further burials were in danger of being destroyed by renewed trenching, that the Department of Archaeological Monuments of the Federal Monuments Office, under the direction of J.-W. Neugebauer, carried out a test investigation on plot 1384/3 in August 1987. Apart from a few burials, the features only extended 10-20 cm into the subsoil! This excavation was continued in four further annual campaigns until 1991 on plots 1385, 1386, 1387, 1384/1-3, 1383, 1389/90 and 1384/2 of KG Kleinhadersdorf.

***Burial custom:***

The graves are predominantly orientated SE-NW and,next most frequently, NW-SE, W-E, E-W, and NE-SW, whereby the deceased were consistently buried lying on their left side of the body in the (highly variable) crouched position usual for the LBK; in only 5 burials were the deceased laid down on their right side. These include, for example, the grave of a woman who died in adulthood (grave Verf. 55), which also differs from other burials in two other parameters – their absolute age (OxA22936: 6228 BP; [^54^](https://paperpile.com/c/2vFjDD/PyMOU):149), which can thus be considered the oldest grave of the Kleinhadersdorf cemetery, and the Strontium isotope ratio of the teeth, which identifies them as non-local (0.712749; [^44^](https://paperpile.com/c/2vFjDD/YuzSY):175). In Kleinhadersorf, in addition to the inhumation graves, there is also evidence of cremation graves (without grave goods). It was a challenge to determine with certainty whether these were cremation graves or "corpse shadows" or the remains of human or animal bodies, as the bones could no longer be located for an osteological assessment. Their number is small, with 4 cremation graves and 59 inhumation graves. Another special feature of this cemetery are the so-called "empty graves". According to Lenneis's assessment, they correspond to the dimensions of the regular burial pits in terms of shape, i.e. outline, size, and depth; several plana were created during the excavation, but no or only a few fragments of human bones were discovered. They are distributed almost evenly over the area of the cemetery and probably date to the first (LBK I / II) and the late phase of occupation (LBK III). Their proportion seems unusually high in Kleinhadersdorf: in addition to the inhumation graves (66 %) and cremation graves (5 %), 29 % of them were empty graves, which – with one exception – is significantly above the average of many known LBK cemeteries. A multi-stage burial ritual with a temporary burial and subsequent exhumation is under discussion as an explanation for this complex phenomenon, which differs from genuine "cenotaphs" or symbolic graves due to traces of clear disturbance in the findings [^55,56^](https://paperpile.com/c/2vFjDD/sqfxB+0vLI0).

As far as grave goods are concerned, the entire range of known LBK grave goods categories can be found, i.e. vessels, pottery fragments, grinding stones, adzes, flints, bone awls, graphite, boar tusks, and tapping stones, as well as jewellery. The grave goods were mainly deposited in the head and upper body area, whereby there appears to be an age and gender-specific correlation. Men's graves are characterised by a more varied range of grave goods (axes, arrowheads, boar tusks, meat offerings, coloured stones, rubbing plates, flint implements and pottery) than women's graves. The equipment of women's graves is less varied (vessels and pottery fragments) and less numerous, but there are clearly more jewellery objects.

A further mortuary custom typical of LBK cemeteries concerns the scattering of red ochre, which was found in 9 of 41 burials (42%) in Kleinhadersdorf, namely three women, three men and three children. A (relatively chronological) assignment to the older occupation phases of the cemetery (the transitional phase LBK I/II and LBK II a) is being discussed; however, this ritual could also have been practised up to the third occupation phase (LBK II b/c). Since not all individuals were radiocarbon dated, but the phase assignment was made because of the pottery, this assumption is subject to a certain degree of uncertainty [^51^](https://paperpile.com/c/2vFjDD/GpA1g).

***Dating:***

In addition to the relative chronological assignments, which were determined based on the pottery finds, radiocarbon dating was also carried out on 18 graves. Human skeletal remains were sampled and analysed either in the VERA lab or in the Oxford AMS laboratory [^54^](https://paperpile.com/c/2vFjDD/PyMOU). These data revealed a period of use of the cemetery between 5220-4980 cal BC [^54^](https://paperpile.com/c/2vFjDD/PyMOU). Interestingly, the so-called "agreement coefficient" (correlation of archaeological and ^14^C dating) showed a very low value, which means that the relative chronology cannot (for the time being) be matched with the ^14^C data; however, this initial finding is based on a small sample, so that a further discussion is pointless until more data is provided.

***Stable isotope analyses:***

The fact that Kleinhadersdorf was included in the LBK Liveway project [^44^](https://paperpile.com/c/2vFjDD/YuzSY) has provided additional insights from the stable isotope analyses beyond the osteological findings. For the carbon and nitrogen analysis, 39 individuals (16 females, 10 males, unsexed adults, six juveniles, and two infants) were analysed. Apart from the children, who show a breastfeeding signal, all isotope values are very homogeneous in a cluster; there are no significant differences regarding age at death and sex. Only the unusually high $\delta$^13^C value of an adult woman is striking (-18.9 ‰; which represents the highest value of all data determined in the Liveway project), which was plausibly explained by a diet rich in C4 plants[^44^](https://paperpile.com/c/2vFjDD/YuzSY).

For the strontium isotope analysis, the molars of 34 individuals (12 males, 10 females, 5 undetermined adults, and 7 non-adults) were sampled. The mean ^87^Sr/^86^Sr ratio is 0.70992 and thus corresponds to the value typical for loess soils [^57^](https://paperpile.com/c/2vFjDD/04KOS). There were no significant sex differences, neither in the Sr isotope ratio nor in the Sr concentration. In general, the results obtained for the stable isotopes are very homogeneous; if there were any social hierarchies, these are not reflected in the stable isotope data.

- **I16006:** Kleinhadersdorf Grab 65, 5209-4947 calBCE (6120±30 BP, PSUAMS-14994)

This unfurnished burial 65 was uncovered by Christine Neugebauer-Maresch in 1990 in the NW area of the cemetery The Kleinhadersdorf skeleton collection (N=62) is kept at the Anthropological Department of the NHM. Corresponding preparatory work (cleaning and preparation) was carried out here and subjected to systematic bioanthropological documentation and analysis for the first time as part of a diploma thesis at the University of Vienna [^53,58^](https://paperpile.com/c/2vFjDD/xTdTJ+IX1cL). Burial Verf. 65 contained the remains of an adult woman (approx. 35(-45) years old) who had been laid down in the grave in a left-sided crouched position (upper body in supine position). The grave is orientated SE-NW, facing SW.

The skeletal remains are largely complete but poorly preserved; epiphyses, parts of the pelvis, and trunk are missing, the cranium is severely damaged and incomplete, and 15 isolated teeth of the lower jaw were recovered.

Several particular features were observed on the skeletal remains: Porotic hyperostosis is diagnosable at the occipital bone; strong abrasion characterises the frontal teeth; strong muscle insertions are located at the *tuberositas deltoidea* (both sides) and *tuberositas glutaea.* These structures are often observed in Neolithic populations and are associated with heavy physical load.

As part of the LBK lifeways project, "*wide-ranging studies of diet, lifetime mobility, health, and physical condition*" [^44^](https://paperpile.com/c/2vFjDD/YuzSY) were carried out based on around 3000 burials. The analyses of the stable isotopes for burial 65 revealed the following: ^87^Sr/^86^Sr ratio 0.708994; $\delta$d^13^C: -20.0, $\delta$^15^N: 9.3.

**1.27 Ratzerdorf (Austria)**

**Author:** Maria Teschler-Nicola

Ratzersdorf at the Traisen Valley is a small village located near the Lower Austrian capital St. Pölten (ca. 60 km west of Vienna). From 1998 on, agriculturally used areas between the northeastern outskirts of Ratzersdorf and the Kremser Schnellstrasse S 33 were converted into land for building for the development of commercial enterprises. As archaeological sites are already known and prospected by aerial photography since 1987/88 [^59^](https://paperpile.com/c/2vFjDD/WaBEm)preventive rescue excavations were carried out in 1998 and 1999 by the Department for Archaeological Monuments of the Federal Monuments Office [^60,61^](https://paperpile.com/c/2vFjDD/VRD2i+Vp0I2).

In July, at the request of the municipal authorities of the provincial capital of St. Pölten, a 14,000 m^2^ area was archaeologically investigated before it was rezoned as industrial territory. During the archaeological investigations in 1998 (led by J.-W. Neugebauer and Christoph Blesl), not only was an extensive settlement from the Early Bronze Age, level Gemeinlebarn ll, discovered, but also twelve east-west oriented burial pits from the late phase of the LBK (Notenkopfkeramik) directly adjacent to the B1 to the east. (One skeleton was complete, from another burial only isolated teeth, dress components and grave goods were preserved; further, only grave goods and/or dress components were recovered from five graves. Additionally, five soil discolourations could be identified as burials due to their orientation, location, and dimensions; they were empty, however). The human skeletal remains are in general, poorly preserved – a fact assumed to be linked to the environment and current intensive agricultural activities [^60^](https://paperpile.com/c/2vFjDD/VRD2i)

In April 1999, the rescue excavations in the projected Ratzersdorf industrial area, which had covered the north-western half of the plot 1629 the previous year, were extended further. Traces of the known Early Bronze Age settlement and eight further graves from the late phase of the LBK (Notenkopfkeramik) cemetery discovered in 1998 along the B1 (federal road no. 1) were documented on an area measuring 120 x 80 meters [^60,61^](https://paperpile.com/c/2vFjDD/Vp0I2+VRD2i). Despite the unfavourable soil composition in this area of the site, some of the burials were reasonably well preserved. Among them was the richest grave, a female burial, which contained six cylindrical spondylus beads from a necklace and a spondylus valve [^62^](https://paperpile.com/c/2vFjDD/9zUnv). The burial site of the late phase of the LBK from Ratzersdorf comprised until then 20 graves, but it was assumed that the necropolis would extend further southeast beyond the B1.

The results of both excavation campaigns in 1998 and 1999 prompted further archaeological investigations in this area and led to the neighbouring properties being placed under protection as well. At the request of the property administration of the municipality of St. Pölten, the archaeological survey of the plot of land 1171 to the southeast of the B1 federal road began in February 2000. By mid-December, 16,000 m^2^ of the 23,000 m^2^ field had been mechanically cleared of surface humus, and more than 750 prehistoric objects of different time levels had been identified, documented, and excavated. Between old water branches of the former Traisen that had silted up, house foundations, storage pits, inhumation burials, and artefacts from various periods (late phase of the LBK; late Neolithic (Baden culture/Ossarn I); early and middle Bronze Age) were found on a slightly raised gravel bank. The late phase of the LBK was represented by long-house floor plans, storage pits, and objects belonging to the classical and late Notenkopfkeramik. During this excavation, one inhumation burial in a crouched position – grave Verf. 1401 – was recovered within the settlement of this period [^63^](https://paperpile.com/c/2vFjDD/2My6k). This individual has been sampled by RP for aDNA analysis in 2015 and included in the present study.

The archaeological investigations were continued in 2001 on plot 1171 and on the neighbouring plots 1170 and 1172. This brought to light further artefacts, such as large clay extraction points from the Linear Pottery settlement and part of a Linear Pottery longhouse

[^64,65^](https://paperpile.com/c/2vFjDD/jpnKv+DOkgA). A Linear Pottery grave (grave Verf. 2010), orientated approximately NNW-SSO, was discovered in the southern part of the investigation area, but only a few human bone fragments and pottery sherds were preserved [^66^](https://paperpile.com/c/2vFjDD/WFVLG).

Some of the finds are kept in the depot of the MAMUZ and the Anthropological Department of the NHM. A bioanthropological analysis of the human remains recovered from the late LBK necropolis and the settlement at Ratzersdorf is so far lacking.

- **I14586:** Ratzersdorf Verf. 1401, 298/2000-1401, 5301-5054 calBCE (6220±30 BP, PSUAMS-15002)

Cranium partially preserved, 19 teeth retained in the sockets, eight teeth isolated, three teeth lost intra vitam; postcranial remains partially preserved; partially eroded bone surface. Based on the obliteration of the sutures and abrasion of the molars, this individual was most likely deceased between 35-45 years (adult-matur). Female, based on cranial and pelvic features and the morphology/robusticity of the long bones.

The following alterations were observed: Porotic inflammatory changes on the palatum, granuloma at the right M3 with lingual breakthrough and adjoining inflammatory changes; spondylarthrotic changes at the *cervical vertebrae* 1 and 2, at some *thoracic* and *lumbar vertebrae*, degenerative changes at the hip joints, enthesopathic changes at *crista tuberculi majoris* of both humeri. Possible peri-mortal fractures observed at the cranial and some postcranial remains are under discussion.

**1.28 Asparn-Schletz (Austria)**

**Author:** Maria Teschler-Nicola

The Early Neolithic settlement of Schletz (Supplementary Figure 17), located in northeastern Lower Austria, represents the most important Linear Pottery site in Austria. Already known for many years through surface finds, it was not until the early 1980s that greater attention was paid to it after oval-shaped soil discolourations became visible during aerial surveys by the Austrian army; these could finally be interpreted as a backfilled ditch system of a settlement [^67,68^](https://paperpile.com/c/2vFjDD/SeQ8o+0NNSk). The site was systematically investigated between 1983 and 2005 in annual archaeological excavation campaigns of the (then) Lower Austrian State Museum under the direction of Helmut Windl. In the process, about 20% of the more than 254,000 m^2^ settlement was exposed, including a complex ditch system with three ditches (to optimise the excavation area, magnetic prospecting was carried out between 1992-1995 by the Central Institute for Meteorology and Geodynamics, see aerial photograph redrawing and entry of archaeomagnetics in Windl et al., 1996 ). These ditches differ in their shape. In addition to an approximately oval construction with two sole ditches (Sohlgräben) running parallel in sections (Ditch I = inner ditch; Ditch II = outer ditch), a third sole ditch (Ditch III) could be traced, enclosing an approximately trapezoidal area north of the oval [^69–72^](https://paperpile.com/c/2vFjDD/pSUy0+e63pe+4uwhk+j2SU3). They reached a maximum width of up to 4 m and a depth of up to 2 m.

Currently, based on the analysis of the pottery, we assume that the settlement and all ditches date to the younger to youngest Linear Pottery, i.e. according to regional chronology to phases IIb - III [^73^](https://paperpile.com/c/2vFjDD/Aafks). According to this, the settlement is Late Linear Pottery (LBK), which was also confirmed by radiocarbon dating, which yielded an age between ca. 5210 and 4950 BCE [^74,75^](https://paperpile.com/c/2vFjDD/VII5s+Rur7r). Within the settlement, numerous settlement objects (postholes of several post-type buildings, cupola ovens, several storage pits, and one object that was interpreted as a well due to its internal construction and depth) and graves either near buildings or in already backfilled ditch sections could be detected. The archaeological finds spectrum includes coarse pottery (bottles, bowls, and bombs), stone and bone implements (including adzes, mace-head fragments, and awls), and abundant animal bone material primarily from the settlement pits and ditches (cattle, sheep, goats, pigs, and dogs; game was less important [^70^](https://paperpile.com/c/2vFjDD/e63pe)).

The site has become the focus of much attention, primarily because of the human skeletal remains recovered from the bottom of ditch II. These remains were found in atypical poses with consistently incomplete representation (hand and foot bones and distal portions of the lower arm and leg are missing). An initial bioanthropological/forensic analysis of the skeletal individuals recovered through 1991 (N=67) revealed an unexpected age-at-death distribution that implied a deficit of young females. In addition, the bony remains were characterised by the presence of a variety of perimortem injuries, interpreted as the effect of close combat weapons (e.g., stone axes and clubs) or distance weapons (such as throwing weapons, slingshot weapons, or bow weapons). It was thus concluded that the individuals died during a violent attack on the settlement dated to circa 5000 BCE, i.e., the final phase of the LBK. Those killed appear not to have undergone any mortuary ritual but were instead left unburied and were thus exposed to circumstances that resulted in a variety of taphonomic alterations, e.g., carnivore activity in the form of biting [^76^](https://paperpile.com/c/2vFjDD/CLoxh). Based on these findings and the fact that artefacts diagnostic of subsequent cultural developments seem to be absent, it was assumed that the site was abandoned.

As archaeological excavations continued through 2005, additional human skeletal remains of unburied bodies were recovered from the outer trench (ditch II) of the settlement, so this sample currently includes 130 individuals (including the 67 already known). Among them, 58 children and adolescents, corresponding to a proportion of about 43% (Infans I (individuals within 0-6 years): 29 children; Infans II (individuals within 6-12 years): 17 children; adolescents: 12 individuals). A total of 74 individuals were inferred to have reached adulthood, corresponding to a proportion of 57% (32 under 40 years of age, 42 over 40 years of age), with a clear preponderance of males (44 males and 24 females). The proportion of young (up to 40 years old) women is lower than that of older (over 40 years old) women and seems to confirm the finding of a "deficit of young females" derived from the smaller sample [^76^](https://paperpile.com/c/2vFjDD/CLoxh). During the continuation of the bioanthropological and paleopathological investigations of the human skeletal remains, the finds recovered at the settlement area of Schletz, which have been excluded from analysis and remained unpublished, are due to be formally published in a study by Pieler and Teschler-Nicola (2023, in press), of which the present section is a summary. In addition to the victims of the violent conflict, two further clusters of human remains were discovered. Among them are 17 regular, “typical” inhumations in grave pits located around the houses or in a sector of Trench II (which had already been filled at the time of the massacre or in abandoned settlement/dwelling pits). When comparing the age and sex profile of the 130 massacre victims and the individuals exhumed at "regular" burials, a significant difference is evident. Male individuals dominate the group of massacre victims located in Ditch II, while females and children predominate in the "typical" settlement burials. This predominance of children and women in settlement inhumations is well known and has also been observed at other Linear Pottery sites.


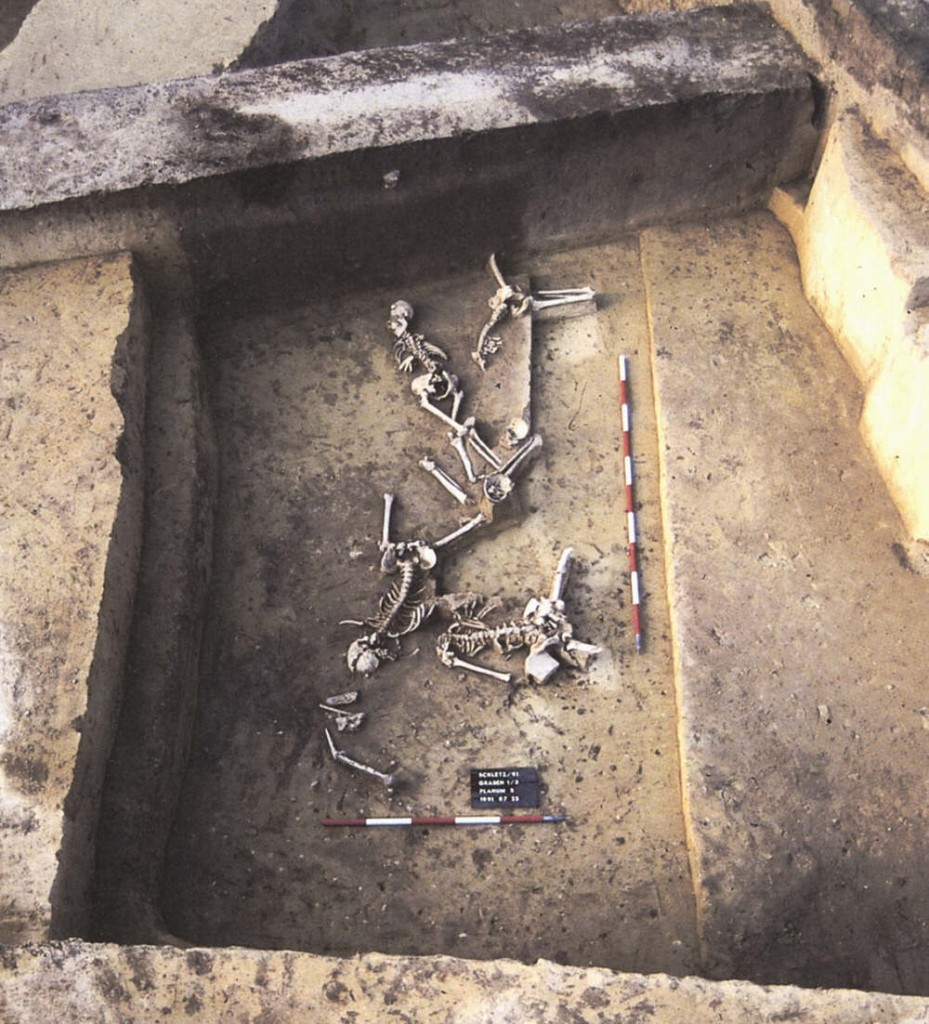


**Supplementary Figure 17:** Skeletal remains of five males in the vicinity of a bridgework *in situ* at the base of the trench of the Asparn-Schletz settlement. massacre. These individuals are related to the violent event. Image from MAMUZ with rights for publication.

Another cluster of human skeletal finds in the settlement area is formed by isolated human skeletal elements or small bone fragments, often mixed with those of animals; they were found throughout the area, most frequently in settlement pits, but also in ditches I and II and in the LBK-era well.

To uncover possible diachronic trends in the treatment of the deceased, a few samples were also taken from the inhumations for radiocarbon dating. Preliminary results indicate that some of the “typical” burials are chronologically older (ca. 5,300 BC) than the victims of the massacre recovered from Ditches I-II (ca. 5,000 BC). However, a few dates overlap, and it is not possible yet to establish a clear dating sequence, an undertaking that would need a much larger number of radiocarbon-dated individuals. One internment yielded a slightly younger 14C date, which may indicate at least occasional use of the site after the violent assault.

Different appearances of human remains from Schletz, with a juxtaposition of apparently carelessly deposited, unburied dead and buried in the rite of the time, as well as of scattered individual bones, testify to different practices that existed within the LBK community. The complex situation of the features, especially the ditches, makes it possible here, as at hardly any other site, to investigate the temporal and spatial sequence and relations of the individual phenomena and to shed light on the possible supraregional connections of conflicts because of assumed socio-economic (climatic changes, scarcity of resources, population increase) or socio-cultural changes towards the end of the Linear Pottery period.

We concluded with a note on terminology for the burials analysed from the Asparn-Schletz sites. As mentioned above, the site was systematically investigated over a more than twenty-year period from 1983 to 2005 in annual archaeological excavation campaigns that were consistently carried out in July/August with the help of annually changing student groups and different excavation directors under the responsibility of the (then) Lower Austrian State Museum and the direction of Helmut Windl. Based on these factors and the varying levels of training of the participants, the protocol records and photographic documentation also vary in their form; in addition, we have also to mention that the complexity of this site with many finds of unburied individuals at the base of a ditch (thus, deviating from "normal" burials that were normally characterised and identifiable by “grave numbers”) and the multitude of isolated, fragmentary bones which were often not immediately identified as "human remains" (thus, became first included in the animal bone assemblages) were only recognised as such in the course of the current anthropological investigations. This complexity is expressed in the different naming of the human skeletal finds, which were handed over in different portions and at different times for anthropological studies: ind + no. (e.g., ind3) = individual number, allocated during the first anthropological investigations in the 1990s; year + no. (e.g., 2000/1 = year of excavation and individual number used for further findings); FN = finding number either for an inhumation burial or for isolated skeletal elements; names (e.g., "Berbel", “Herbert” ) were allocated by the members of the excavation team; we abstained here from this practice and used the FN.

- **I30418**: ind. 4, 1991, FN 3340, 5300-5000 BCE
- **I24884**: 93/17, 1993, FN 4520, 5205-4847 calBCE (6075±35 BP, VERA-2012)
- **I24885**: 93/18-2, 1993, FN 4202, 5215-5008 calBCE (6175±35 BP, VERA-2007)
- **I24891**: 93/7-1, 1993, FN 4223/4224/4381/433, 5203-4784 calBCE (6025±55 BP, ETH-14373)
- **I24898**: 97/2, 1997, FN 5838, 5300-5000 BCE
- **I24899**: 97/4, 1997, FN 5839, 5215-5016 calBCE (6175±30 BP, VERA-2737)
- **I27771**: 93/10, 1993, FN 4269, 5250-4800 BCE
- **I27772**: 93/11, 1993, FN 4264/25310, 5308-5058 calBCE (6235±40 BP, VERA-2020)
- **I27773**: 93/15, 1993, FN 4472, 5296-5047 calBCE (6205±30 BP, VERA-2738)
- **I27774**: 93/16, 1993, FN 4476, 5300-5000 BCE
- **I27776**: 93/17, 1993, FN 4520, 5207-4945 calBCE (6113±23 BP) [R_combine: (6130±35 BP, VERA-2010); (6100±30 BP, VERA-2011)]
- **I27778**: 93/19-2, 1993, FN 4503, 5300-5000 BCE
- **I27780**: 96/4, 1996, FN 5185, 5300-5000 BCE
- **I25323:** 96/6, 1996, FN 5081, 5300-5000 BCE
- **I27783**: 98/2, 1998, FN 6567, 5300-5000 BCE
- **I27784**: 98/4, 1998, FN 6449, 5300-5000 BCE
- **I27787_d**: 2001, FN 10640 (“Sa”), 5000 BCE
- **I27788_d**: 2001, FN 11057 (“Pa”), 5000 BCE
- **I25334**: 2003, FN 12566, 5300-5000 BCE
- **I25336**: 2003, FN 12626, 5300-5000 BCE
- **I27793**: FN 12671, 5300-5000 BCE
- **I27794**: ind 46, 1985, FN 209, 5300-5000 BCE
- **I27796**: ind. 52, 1988, FN 763, 5300-5000 BCE
- **I27805**: 2005, FN 14560, 5300-5000 BCE
- **I24268**: 2001, FN 11051 (“Ph”), 5300-5000 BCE
- **I24270**: 2003, FN 12549, 5300-5000 BCE
- **I24271**: 2003, FN 12670, 5300-5000 BCE
- **I24275**: 1985, FN 265, 5300-5000 BCE
- **I24281**: ind. 41, 1987, FN 670, 5209-4951 calBCE (6125±35 BP, VERA-2014)
- **I24282**: ind. 44, 1987, FN 646, 5626-5525 calBCE (6646±21 BP) [R_combine: (6657±26 BP, MAMS-46038), (6627±35 BP, MAMS-48728)]
- **I24283**: ind. 50, 1987, FN 601, 5300-5000 BCE
- **I24285:** ind. 25,1989, FN 1449, 5300-5000 BCE
- **I30413**: ind. 6, 1983, FN 75a/77, 5300-5000 BCE
- **I24289**: ind. 51, 1990, FN 2865, 5300-5000 BCE
- **I24886**: 93/13, 1993, FN 4471, 5300-5000 BCE
- **I24887**: 93/14, 1993, FN 4473, 5300-5000 BCE
- **I24888**: 93/19-1, 1993, FN 4503, 5311-5072 calBCE (6254±31 BP, MAMS-42229)
- **I24889**: 93/20, 1993, FN 4529, 5313-5074 calBCE (6258±31 BP, MAMS-42232)
- **I24890**: 93/25, 1993, FN 4444, 5300-5000 BCE
- **I24892**: 93/4, 1993, FN 4464/4518, 5197-4844 calBCE (6055±35 BP, VERA-2009)
- **I24276**: 1985, FN 278, 5300-5000 BCE
- **I30433** (I24277): ind. 56, 1983, FN 88a/110, 5300-5000 BCE
- **I24279**: ind. 63, 1985, FN 286, 5300-5000 BCE
- **I30431** (I24280): 1984, FN 162, 5300-5000 BCE
- **I24893**: 93/5, 1993, FN 4333, 5211-4995 calBCE (6145±35 BP, VERA-2008)
- **I24894**: 93/6, 1993, FN 4456, 5300-5000 BCE
- **I24895**: 93/7-2, 1993, FN 4223, 5300-5000 BCE
- **I24896**: 93/9, 1993, FN 4451, 5300-5000 BCE
- **I24897**: 95/1, 1995, FN4694, 5300-5000 BCE
- **I24900**: 97/3, 1997, FN 5613, 5300-5000 BCE
- **I24901**: 97/7, 1997, FN 5959, 5300-5000 BCE
- **I24902**: 98/1, 1998, FN 6316, 5300-5000 BCE
- **I24903**: 99/1, 1999, FN 7899, 5300-5000 BCE
- **I24905**: 99/4, 1999, FN 8264,5300-5000 BCE
- **I30421**: 96/3, 1996, FN 5184, 5300-5000 BCE
- **I30423**: ind. 5, 1991, FN 3341, 5300-5000 BCE
- **I30425**: ind. 7a, 1990, FN 2490, 5300-5000 BCE
- **I30428**: 96/2, 1996, FN 5076, 5300-5000 BCE
- **I30430:** ind. 1, 1991, FN 3342/3141, 5300-5000 BCE
- **I27800:** ind. 7b, 1990, FN 2491,5300-5000 BCE
- **I27785**: 99/2, 1999, FN 8053, 5300-5000 BCE
- **I30434**: ind. 47, 1985, FN 281, 5300-5000 BCE
- **I30414**: 1983, FN 53, 5300-5000 BCE
- **I24907**: 2001, FN 10821,5300-5000 BCE
- **I25349**: 2000, FN 10351, 5206-4935 calBCE (6100±25 BP, PSUAMS-11286)
- **I24286_d**: ind. 28, 1989, FN 1465, 5300-5000 BCE
- **I24906:** (P8467), 2001, FN 10806 (“Je”), 5300-5000 BCE
- **I24269**: 2001, FN 11660 (“Ph”), 5300-5000 BCE
- **I24278**: ind. 57, 1985, FN 249, 5300-5000 BCE
- **I25347**: Graben 3, FN 3491, 5300-5000 BCE
- **I24272**: 2005, FN 14143, 5000 BCE
- **I24904**: 99/3, 1999, FN 8328, 5300-5000 BCE
- **I30411**: 2000, FN 10343,_settlement (“Be”),5300-5000 BCE
- **I24015**: 2000, FN 9872,_settlement burial 16-1 (“He-1”), 5300-5000 BCE
- **I24016**: 2000, FN 9366, settlement burial 15 (“Si”), 5033-4847 calBCE (6050±25 BP, MAMS-38865)
- **I24017**: 2000, FN 9230, settlement (“Trau-1”), 5300-5000 BCE
- **I24018**: 2000, FN 9230, settlement (“Trau-2”), 5301-5053 calBCE (6219±31 BP, MAMS-38863)
- **I24021**: 2000, FN 9872, settlement burial 16-3 (“He-3”), 5214-5040 calBCE (6174±25 BP, MAMS-38866)
- **I24022**: 2000, FN 9872, settlement burial (“He-2”), 5300-5000 BCE
- **I24023**: 2001, FN 11449, settlement burial 17 (“Ed”), 5300-5000 BCE
- **I24024**: 2001, FN 11676, settlement burial 18 (“Da”), 5300-5046 calBCE (6210±35 BP, VERA-2198)
- **I24025**: 1986, FN 347, 5302-5041 calBCE (6210±40 BP, VERA-2016)
- **I24026**: 2005, FN 14300, burial (“Grö”), 5186-5120 calBCE (6207±25 BP, MAMS-38867)
- **I24027**: 2004/2005, FN 13627, burial (“He”), 5300-5000 BCE
- **I24028**: 2001, FN 11803, burial (“Da”), 5213-5008 calBCE (6165±35 BP, VERA-2441)

**1.29 Nitra-Horné Krškany (Slovakia)**

**Authors:** Daniela Hofmann, Penny Bickle.

Located in the Nitra river valley in western Slovakia, the cemetery lies just where the foothills of the Carpathian mountains begin to stretch eastwards. Like many Linearbandkeramik (LBK) sites, it is located on loess soils, which are found intermittently as the higher ground becomes the Danubian plain to the south and west [^77^](https://paperpile.com/c/2vFjDD/oHgo8),. The site came to light in the course of rescue excavations in advance of the construction of a potato storage building in the south of the modern town of Nitra, in an area named Horné Krškany, about 250–300m from the river bank [^78^](https://paperpile.com/c/2vFjDD/eWTKc). In 1964 and 1965, Pavúk [^77^](https://paperpile.com/c/2vFjDD/oHgo8) excavated at the site, identifying 76 graves, two of which were empty, with a few graves destroyed by the initial building works [^79^](https://paperpile.com/c/2vFjDD/sU6FL). The excavations opened up two parallel trenches, covering an area of 50m by 15m, which seems to have included most of the graves that had been preserved, as further test pitting to the north-east and southwest did not uncover more graves [^77^](https://paperpile.com/c/2vFjDD/oHgo8). Today 74 individuals are known, with a further 8 cremations, all of which appear to date to the LBK based on both ceramic styles [^77^](https://paperpile.com/c/2vFjDD/oHgo8) and the radiocarbon dating of 12 graves [^80^](https://paperpile.com/c/2vFjDD/wvb3n),[^77^](https://paperpile.com/c/2vFjDD/oHgo8). Assessment of the ceramics accompanying the burials suggested that the cemetery was used over two to three centuries. In terms of Pavúk’s chronology, this is from the LBK phase II to the Želiezovce phase, or from the second expansion of the LBK, usually dated to about 5300 calBCE, to its end around 5000 calBCE. A Bayesian model of the 12 radiocarbon dates from the cemetery falls roughly in line with the suggestion of Pavúk and estimates that the cemetery started in *5370–5220 calBCE (95.4% probable)* or *5320–5230 calBCE (68.2% probable)*. The end of activity at the Nitra cemetery is estimated to have occurred in *5210–4980 calBCE (95.4% probable)* or *5210–5090 calBCE (68.2% probable)*. The duration of burial at the site is therefore estimated to have lasted between *20–360 years (95.4% probable)* or *30–220 years (68.2% probable)* [*^80^*](https://paperpile.com/c/2vFjDD/wvb3n). Thus, it seems possible that the cemetery received burials for anything from a generation to several centuries.

The burials themselves cluster in the northeastern part of the trenches, but beyond this, and in contrast to other cemeteries (e.g. Vedrovice, Cz, and Aiterhofen, Germany), other groupings or sub-divisions are not obvious. Pavúk, [^77^](https://paperpile.com/c/2vFjDD/oHgo8) suggested that the graves may be arranged in lines rather than groupings. Some 22 burials appear associated through nine sets of intercutting grave pits (in pairs or clusters of three burials), which is a feature far rarer at other cemeteries (but also suggested for Elsloo [^81^](https://paperpile.com/c/2vFjDD/gqyPo). Burials are largely found in single inhumations, except for a triple burial (individuals 48, 49, and 50) of an adult female with two children, the latter of whom had received blows to the head, which most likely caused their deaths [^44^](https://paperpile.com/c/2vFjDD/YuzSY). Most graves were oriented along a southeast-to-northwest axis, with the head to the southeast, though burials could fall between east–west, and south–north [^77^](https://paperpile.com/c/2vFjDD/oHgo8). As another counter-point to other LBK cemeteries, no burials are found in the antipodal orientations. Where body position can be determined, the deceased was placed in a crouched position mostly on their left-hand sides [^77^](https://paperpile.com/c/2vFjDD/oHgo8), though a few were found with the upper part of their body on the front or back. Only two burials (43 and 71) were found on their right-hand sides. Overall, there is more similarity in grave orientation and body position than at other LBK cemeteries.

The grave goods accompanying the burials are typical for LBK cemeteries and comprise pottery, polished stone tools, chipped stone implements, imported *Spondylus* shell either as beads or as “belt buckles'', worked bone, ochre colouring and, in one instance, pieces of graphite (burial 5) [^77^](https://paperpile.com/c/2vFjDD/oHgo8). About a third of burials were accompanied by no grave goods at all, which is comparable with other LBK cemeteries ([^44^](https://paperpile.com/c/2vFjDD/YuzSY), 142). Unusually, one burial is accompanied by seven perforated human and dog or fox teeth (burial 19) ([^77^](https://paperpile.com/c/2vFjDD/oHgo8), 11). Pottery decorated in a style that mixed local patterns with those more closely associated with the Alföld Linear Pottery group (located in north-eastern Hungry) was found in grave 17, suggesting wider connections ([^77^](https://paperpile.com/c/2vFjDD/oHgo8), 84). This particular connection is also suggested by a pot in Grave 2 ([^77^](https://paperpile.com/c/2vFjDD/oHgo8), 84). Overall, older men, and to a lesser extent older women, appeared to be accompanied by the most numerous and diverse grave good assemblages, which led Pavúk [^77^](https://paperpile.com/c/2vFjDD/oHgo8), 72) to suggest that Nitra was a gerontocratic society, with status increasing for some as they aged. This trend for older individuals to have the most grave goods also holds true when using up-to-date methodologies for estimating age and sex from skeletal data^2^.

The osteological collection from Nitra has been subject to several different studies. Linda Fibiger, for the LBK lifeways project, carried out an extensive assessment. The 75 individuals studied by Fibiger consisted of 27 adult females, 18 adult males, 4 unsexed adults, six adolescents, 16 juveniles, and four infants [^44^](https://paperpile.com/c/2vFjDD/YuzSY). This is probably not representative of a living population and under-represents the likely rate of infant mortality. Dočkalová and Čižmář [^82,83^](https://paperpile.com/c/2vFjDD/Kn7Un+XJQSB) have demonstrated that in this region, higher rates of non-adults were buried in settlements, suggesting some deliberate selection for burial in cemeteries based on age. Among the adult burials, females have slightly higher representation in the young adult category (18-25 years at death), likely representing death in pregnancy or childbirth; otherwise, males and females are found in roughly equal proportions as they aged, with the highest numbers of individuals falling into the mid-adult category [^44^](https://paperpile.com/c/2vFjDD/YuzSY). Evidence for metabolic and infectious conditions, as well as generalised stress markers like enamel hypoplasias, were equal between the sexes, suggesting that periods of stress likely affected the whole population [^44^](https://paperpile.com/c/2vFjDD/YuzSY). Overall, at least a fifth of the population had signs of periosteal changes and infection. Alongside the two children noted above, a young adult male (72) and a young adult female (1) also showed traces of skull trauma [^44^](https://paperpile.com/c/2vFjDD/YuzSY).

Sex-based differences were suggested based on a number of lines of evidence. Higher rates of dental caries in women, coupled with higher δ15N values [^44^](https://paperpile.com/c/2vFjDD/YuzSY) in males, indicate a degree of dietary differences, but these were not detectable by dental microwear [^84^](https://paperpile.com/c/2vFjDD/z4nfC). In particular, males buried with polished stone axes had higher nitrogen values than the site mean, while all burials accompanied by imported *Spondylus* shell (independently of the sex of the deceased) had higher δ13C values on average than the rest of the population [^44^](https://paperpile.com/c/2vFjDD/YuzSY). Stronger associations between the sex of the deceased and strontium isotope ratios were found. Women were found to have a much wider range of strontium isotope values, and all 6 of the individuals falling above the upper limit of the loess strontium range were women [^44^](https://paperpile.com/c/2vFjDD/YuzSY). A sexed division of labour has since also been identified from use-wear analysis of the stone tools accompanying male and female burials. Males were associated with tools that had been used in woodworking, animal butchery and/or interpersonal violence, and harvesting, and women with tools for hide working. Occlusal grooves on teeth also indicated that women engaged in sinew or plant fibre processing more often than males [^85^](https://paperpile.com/c/2vFjDD/9DFCw); [^44^](https://paperpile.com/c/2vFjDD/YuzSY).

- **17346**: 2946, grave 7, 5300-5000 BCE
- **I17539**: 2939, grave 40, 5300-5000 BCE
- **I18106**: 50, grave 8, 5300-5000 BCE
- **I11866**: 209, grave 56, 5300-5000 BCE
- **I11872**: 216, grave 26,5300-5000 BCE
- **I14177**: 1530, grave 67, 5300-5000 BCE
- **I14178**: 1514, grave 50, 5300-5000 BCE
- **I14179**: 1490, grave 30, 5300-5000 BCE
- **I14180**: 1525, grave 62, 5300-5000 BCE
- **I14181**: 1537, grave 74, 5300-5000 BCE
- **I14182**: 1518, grave 55, 5300-5000 BCE
- **I14183**: 73, grave 73 , 5300-5000 BCE
- **I14599**:1483 grave 22, 5300-5000 BCE
- **I14600**:1466 grave 4, 5300-5000 BCE
- **I16008**:1474 grave 14, 5300-5000 BCE
- **I16010**:1534, grave 71, 5300-5000 BCE
- **I16011:** 54, grave 54, 5300-5000 BCE
- **I16012**:1489, grave 29, 5300-5000 BCE
- **I16013**: 1508, grave 44 , 5300-5000 BCE
- **I16014**: 1503, grave 39, 5300-5000 BCE
- **I16015**: 1529, grave 66, 5300-5000 BCE
- **I16016**: 1522, grave 59, 5300-5000 BCE
- **I16239**: 1463, grave 1, 5300-5000 BCE
- **I16240**: 1511, grave 47, 5300-5000 BCE
- **I16242**: 1507, grave 42, 5300-5000 BCE
- **I16245**: 1479, grave 19, 5300-5040 calBCE (6196±36 BP, OxA-24576)
- **I17339**: 1484, grave 24, 5300-5000 BCE
- **I17340**: 1493, grave 33, 5300-5000 BCE
- **I17341**: 1531, grave 68, 5300-5000 BCE
- **I17343**: 1539, grave 76, 5300-5000 BCE
- **I17344**: 1464, grave 3, 5300-5000 BCE
- **I17345**: 1468, grave 5, 5310-5050 calBCE (6222±37 BP, OxA-24574)
- **I17545**: 1476, grave 16, 5300-5000 BCE
- **I18093**: 1469, grave 6, 5310-5050 calBCE (6226±36 BP, OxA-24575)
- **I18094**: 1478, grave 17, 5300-5000 BCE
- **I18097**: 1485, grave 25, 5310-5050 calBCE (6216±36 BP, OxA-24577)
- **I18111**: 8541, grave 21, 5211-5006 calBCE (6160±35 BP, PSUAMS-11096)
- **I25176**: 1516, grave 53, 5300-5000 BCE
- **I18143**: 5628, grave 8, 5300-5000 BCE
- **I18144**: 8539, grave 36,5310-5060 calBCE (6227±35 BP, OxA-24580)
- **I25201**: 1487, grave 27, 5310-5050 calBCE (6221±35 BP, OxA-23793)
- **I18091**: 1465, grave 2, 5330-5210 calBCE (6298±33 BP, OxA-24095)
- **I16009**: 1481, grave 23, 5300-5000 BCE
- **I16246**: 156078, grave 9, 5300-5000 BCE
- **I16007**: 1513, grave 49, 5300-5000 BCE
- **I16241**: 1475, grave 15, 5300-5000 BCE
- **I17538**: 1501, grave 38, 5300-5000 BCE
- **I11873**: 1512, grave 48, 5300-5000 BCE
- **I25175**: 1515, grave 52, 5300-5000 BCE
- **I18105**:1533, grave 70, 5380-5210 calBCE (6328±36 BP, OxA-24582)

**1.30: Jelšovce (Slovakia)**

**Authors**: Matej Ruttkay, Jozef Bátora

During extensive rescue research at the polycultural site in Jelšovce, in the district of Nitra in southwestern Slovakia, it was possible to examine a part of the Neolithic settlement, which was represented by the Želiezovce group. Among the uncovered houses, house 615 stood out from the others, in the foundation gutter of which were found the skeletons of two adult women aged 40-50 years old. These skeletons were marked as graves 615A and 615 B.

The burial of individuals directly in the foundation gutter has not yet been well documented in the Central European area from the early Neolithic period [^86^](https://paperpile.com/c/2vFjDD/06sbW). The find from Jelšovce is therefore of particular importance.

Because traces of injuries were found on the skulls of both women buried in the foundation gutter of house [^87^](https://paperpile.com/c/2vFjDD/XsLeI), a possibility is that both women died violent deaths as part of ritual ceremonies held in connection with the start or end of the construction of a new house [^88^](https://paperpile.com/c/2vFjDD/CrNq9).

- **I24295**: Jelšovce 615b, 5500-4500 BCE

Skeleton A was located in the lower narrowed part of the western half of the foundation gutter at a depth of 43 cm, oriented in the NE - SW direction. Due to the narrow space (the width of the trough was 23-30 cm), the skeleton was forced into the gutter. There were 3 vessels at a depth of 15 cm above the skull of the skeleton.

- **I24868**: Jelšovce 615a/87, 5210-4992 calBCE (6135±30 BP, PSUAMS-15001)

Skeleton B was located in the eastern part of the gutter at a depth of 30-36 cm. It was placed on the right side, oriented in the WSW - ENE direction. The view of the facial part of the skull was directed to the SSE. No grave goods were found by the skeleton.

**1.31: Nitra Mlynárce**

**Authors:** Matej Ruttkay, Jaroslava Ruttkayová, Alena Šefčáková

The burial site was discovered in 1951 during construction activities, by which time many graves had already been destroyed. The site is located in the Nitra-Mlynárce cadastre, 4 km NW of the city of Nitra (SW Slovakia), near the Nitra River.

Over time, graves were discovered in several locations. During 1951-53 at least 16 graves were examined on the right bank terrace of the Nitra River, five of them concentrated in an area of 8.5x3.5m (graves 1-5/51) about 300 m from the settlement, another group consisted of 10 graves (graves 1-10/52), and an additional grave (1/53) was found in 1953 [^89,90^](https://paperpile.com/c/2vFjDD/wSykL+YPsC8).

The orientation of the graves was inconsistent. The dead were most often placed in a crouched position on the left flank, with only grave 1 on the right flank. In one of the best-preserved graves, the deceased was laid on their back with their arms bent sharply at the elbows and hands resting on their shoulders. Their face was tilted towards the south, and their lower limbs were also bent in that direction [^89^](https://paperpile.com/c/2vFjDD/wSykL). Another grave was a double burial, where the deceased were interred in an antipodal position - that is, one behind the other in different orientations, with their feet facing each other [^91^](https://paperpile.com/c/2vFjDD/bqslK). Graves 1/52 and 2/52 were probably in superposition.

Red dye traces were discovered on the skeletons. The grave goods included pottery, an amphibolite axe and hoof wedges, shell and marble beads, bone needles, and artefacts from a chipped stone industry [^89,92^](https://paperpile.com/c/2vFjDD/wSykL+xNjPw). Evidence of an LBK settlement was discovered near [^89^](https://paperpile.com/c/2vFjDD/wSykL). Several archaeological excavations have been conducted on both banks of the Nitra River in Nitra-Mlynárce. In addition to Neolithic findings (LBK, Želiezovce culture), evidence of settlements and/or cemeteries from various periods - including Eneolithic (Ludanice group and Baden culture), Bronze Age, La Tène period, Roman period and Middle Ages – have been discovered here [^89,90,92–94^](https://paperpile.com/c/2vFjDD/YPsC8+wSykL+xNjPw+EZhPX+qrSmv).

- **I7892**: NTMY_2/52, 5250-4800 BCE

Female (obtained osteologically), young individual (juvenis/adultus I), a damaged gracile to medium robust postcranial skeleton, and fragmentary *cranium* with weak muscular relief.

- **I7893**: NTMY_3/52, 5205-4847 calBCE (6075±35 BP, PSUAMS-15084)

Female (obtained osteologically and genetically), ca 20-30 years (adultus I), a damaged gracile postcranial skeleton, and fragments of *cranium*.

- **I7894**: NTMY_4/52, 5250-4800 BCE

Male (obtained osteologically and genetically), ca 35-45 years (adultus), a damaged gracile to medium robust postcranial skeleton and fragments of *cranium*, sacralisation on the *os sacrum*.

- **I7895**: NTMY_5/52, 5250-4800 BCE

Male (obtained osteologically and genetically), ca 40-60 years (maturus), a damaged rather gracile postcranial skeleton, and fragments of *cranium* with medium muscular relief.

- **I7896**: NTMY_7/52, 5250-4800 BCE

Female (obtained genetically), ca 8-12 years (infans II), a very damaged skeleton.

**Section 2: Radiocarbon dates and modelling**

**Authors:** Pere Gelabert, Olivia Cheronet, Penny Bickle

Radiocarbon dating was carried out to confirm human remains were the date proposed by any accompanying material culture and their archaeological context when the material was available. 19 dates were produced in the course of this project (Supplementary Table 13), supplemented by dates already published. All dates are reported alongside the burials in Supplementary Information 1, with their quality information data where available. Where three or more dates were available from sites, these were subjected to Bayesian modelling to refine our chronological understanding. This was possible at six sites, Füzesabony-Gubakút, Polgár-Ferenci-hát, M3-31, Nitra Horné Krškany, and Asparn-Schletz, where the ditch burials, water well burials and those from the settlement context were modelled separately.

**Supplementary Table 13:** New Radiocarbon dates generated in this study

| Sample ID | Code | Method for Determining Date | Date mean in BP | SD | Full Date |
| --- | --- | --- | --- | --- | --- |
| **I7869** | MSTG_1 | Direct: IntCal20 | 6953 | 69 | 5205-4852 calBCE (6085±30 BP, PSUAMS-14993)* |
| **I8117** | PANC_7 | Direct: IntCal20 | 7648 | 33 | 5765-5631 calBCE (6820±35 BP, PSUAMS-15077) |
| **I14586** | 1186 | Direct: IntCal20 | 7113 | 70 | 5301-5054 calBCE (6220±30 BP, PSUAMS-15002) |
| **I16006** | 1322 | Direct: IntCal20 | 7019 | 76 | 5209-4947 calBCE (6120±30 BP, PSUAMS-14994) |
| **I17362** | 3063 | Direct: IntCal20 | 7214 | 37 | 5329-5211 calBCE (6295±30 BP, PSUAMS-14995) |
| **I17363** | 3064 | Direct: IntCal20 | 7144 | 75 | 5305-5064 calBCE (6235±30 BP, PSUAMS-14996) |
| **I17366** | 3067 | Direct: IntCal20 | 7205 | 43 | 5321-5127 calBCE (6275±30 BP, PSUAMS-14997) |
| **I17927** | 3629 | Direct: IntCal20 | 7519 | 40 | 5631-5482 calBCE (6645±40 BP, PSUAMS-15078) |
| **I17938** | 3643 | Direct: IntCal20 | 7106 | 70 | 5302-5047 calBCE (6215±35 BP, PSUAMS-15079) |
| **I17947** | 3061 | Direct: IntCal20 | 7217 | 43 | 5359-5212 calBCE (6300±35 BP, PSUAMS-15080) |
| **I17948** | 3062 | Direct: IntCal20 | 7105 | 67 | 5299-5052 calBCE (6215±30 BP, PSUAMS-14998) |
| **I18635** | 3647 | Direct: IntCal20 | 7130 | 76 | 5306-5056 calBCE (6230±35 BP, PSUAMS-15081) |
| **I21906_d** | P6552 | Direct: IntCal20 | 7510 | 39 | 5623-5483 calBCE (6625±30 BP, PSUAMS-14999) |
| **I28426** | P9678 | Direct: IntCal20 | 7511 | 40 | 5625-5482 calBCE (6630±35 BP, PSUAMS-15082) |
| **I29877** | HUNG484 | Direct: IntCal20 | 7940 | 44 | 6069-5909 calBCE (7120±40 BP, PSUAMS-15083) |
| **I29891** | HUNG680 | Direct: IntCal20 | 6982 | 76 | 5208-4907 calBCE (6100±30 BP, PSUAMS-15000) |
| **I7893** | NTMY_3, 52 | Direct: IntCal20 | 6936 | 72 | 5205-4847 calBCE (6075±35 BP, PSUAMS-15084) |
| **I24296** | P8498 | Direct: IntCal20 | 7071 | 59 | 5215-5008 calBCE (6175±35 BP, PSUAMS-15085) |
| **I24868** | P8416 | Direct: IntCal20 | 7041 | 71 | 5210-4992 calBCE (6135±30 BP, PSUAMS-15001) |

*Not included individual due to date.

**Dates modeling**

Chronological modelling was undertaken using the program OxCal v4.4 and the IntCal 20 [^95^](https://paperpile.com/c/2vFjDD/FR5Yq) calibration curve [^96^](https://paperpile.com/c/2vFjDD/S8Vzz), to produce simple phase models, assessing the probable start, end, and duration of the burials.

**Nitra Horné Krškany**

The modelling of this site suggests that burials began between 5347 and 5214 calBCE (95.4% probability), and ended between 5199 and 4979 cal BCE (95.4% probability), with a span of 20-341 years. Overall, the model had good agreement, with Amodel=91.61%, Aoverall=92.2%, but one date had poor agreement (OxA-24581). The model is reported in Supplementary Figure 18.


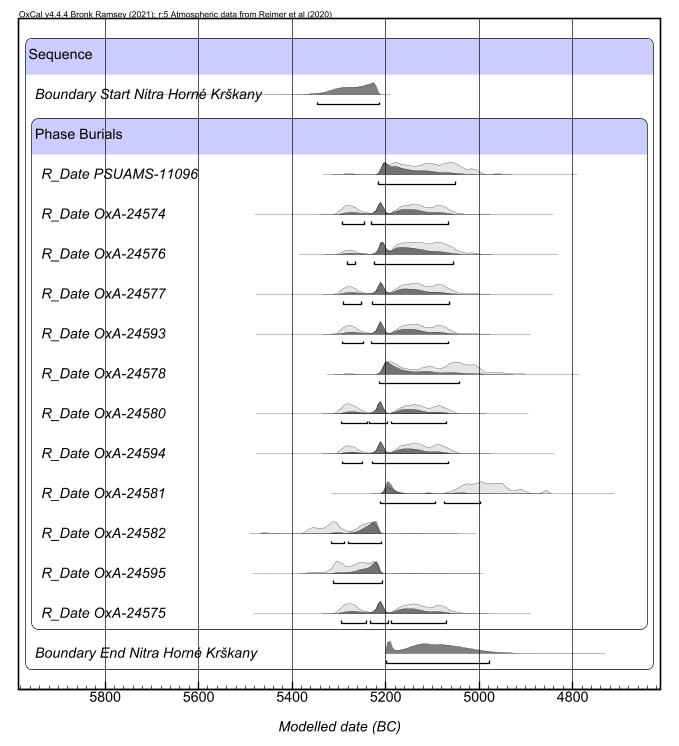


**Supplementary Figure 18:** date modelling of Nitra Horné Krškany.

**Füzesabony-Gubakút**

The modelling of this site suggests that burials began between *5485* and *5244 cal BC (95.4% probability)*, and ended between *5292* and *5076* *cal BC (95.4% probability*), with a span of 0-369 years. Overall, the model had good agreement, with Amodel=85.1%, Aoverall=87.3%, but two dates had poor agreement (deb-131042 and PSUAMS-10194). The model is reported in Supplementary Figure 19.


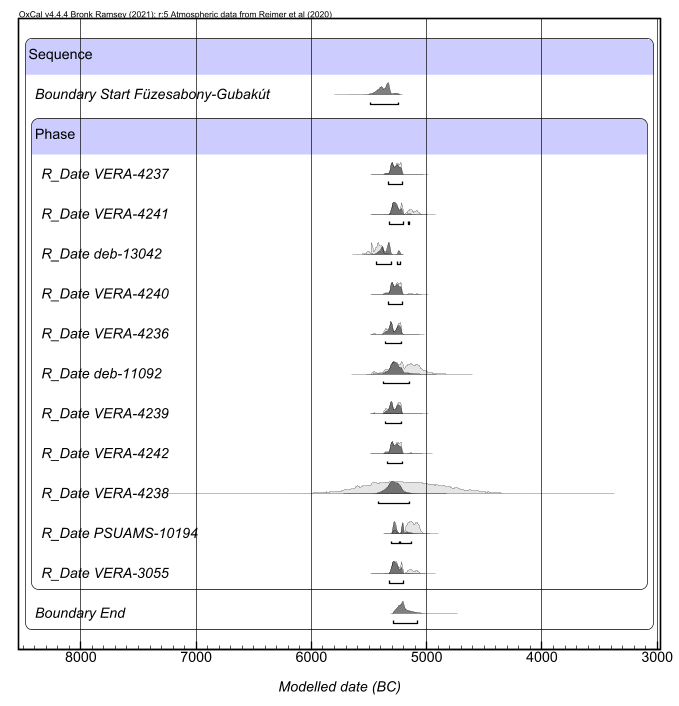


**Supplementary Figure 19:** Date modelling of Füzesabony-Gubakút.

**Polgár-Ferenci hát, M3-31**

The modelling of this site suggests that burials began between *5507* and *5321 cal BC (95.4% probability)*, and ended between *5193* and *4871* *cal BC (95.4% probability*), with a span of 151-588 years. Overall, the model had good agreement, with Amodel=81.1%, Aoverall=83%, but one date had poor agreement (PSUAMS-10207). The model is reported in Supplementary Figure 20.


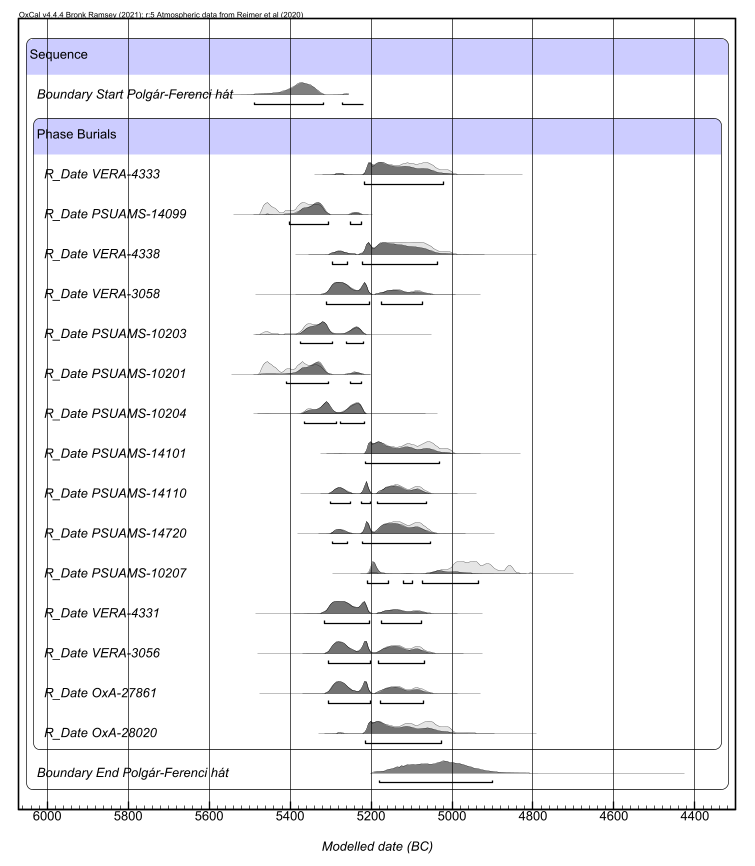


**Supplementary Figure 20:** Date modelling of Polgár-Ferenci-hát

**Asparn-Schletz**

For Asparn, we modelled the three different sets of burials, each assumed to have a different chronology unrelated to other burials at the site: settlement burials, three burials from a well, and the massacre. To handle this, we modelled them as overlapping phases. Overall, the model had good agreement, with Amodel=107.1%, Aoverall=97.2%. The modelling of this site suggests that settlement burials began between 5343 and 5084 cal BC (95.4% probability), and ended between 5161 and 5124 cal BC (95.4% probability), with a span of 9-460 years. For the well burials, the modelling suggests that burial began between 5610 and 5125 cal BC (95.4% probability), and ended between 5302 and 4848 cal BC (95.4% probability), with a span of 0-686 years. For the massacre, the modelling suggests that the individuals died between 522 and 5017 cal BC (95.4% probability), and ended between 5044 and 4861 cal BC (95.4% probability), with a span of 0-253 years. The model is reported in Supplementary Figure 21.


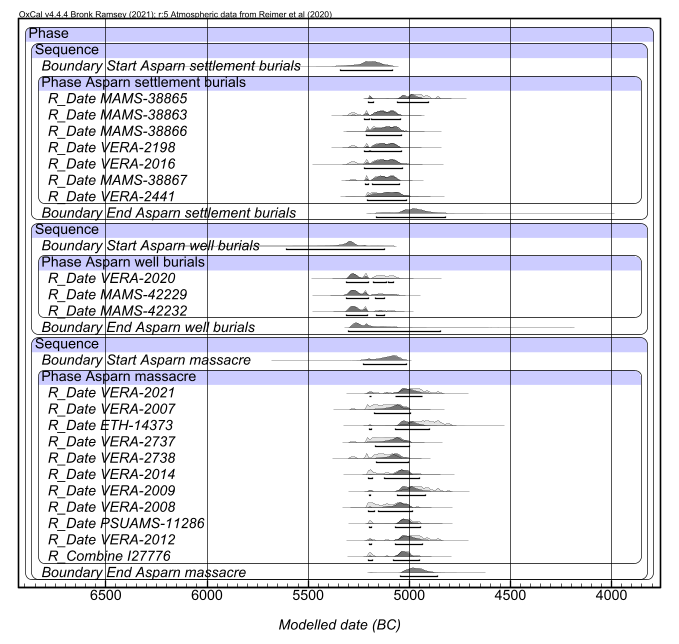


**Supplementary Figure 21:** Date modelling of Asparn-Schletz

**Section 3: Integration of genetic and isotopic data**

**Author:** Penny Bickle

Our analyses asked two questions, enabled by a comparison of the newly reported genetic data to the already collected isotopic data.

(1) Were there statistical differences in the means and variance of the isotope data when relatives (up to a third-degree) and non-relatives were compared? Due to the differences demonstrated between the sexes in previous studies, we also analysed related and unrelated individuals by biological sex.

(2) Did “family” groupings demonstrate different means and variances? As family groups are likely to have non-biological relatives, we interpret any of these differences as roughly indicating that kinship differences shaped diet and mobility.

Three main isotopes were analysed: carbon, nitrogen, and strontium. Total strontium concentration in the tooth was also studied. Carbon δ^13^C and nitrogen δ^15^N provide information about dietary protein consumption, with nitrogen isotopes varying with trophic level and hence meat protein consumption. Carbon isotopes are likely to primarily reflect variation in forest canopy cover in an LBK context (rather than consumption of C3 vs. C4 plants as at most later times) [^97,98^](https://paperpile.com/c/2vFjDD/cErZY+NPaGe). Although some freshwater fish consumption may have occurred in the LBK, it is unlikely to have significantly impacted stable isotope variation [^99^](https://paperpile.com/c/2vFjDD/WiCnU). Strontium isotopes, analysed in tooth enamel, are sensitive to lifetime mobility when the Sr ratio from the tooth differs from that of the local geology [^100^](https://paperpile.com/c/2vFjDD/eXSAF).

We used Levene’s test for assessing homogeneity in variance. This is particularly significant for analysing strontium isotope ratios where differences between groups are likely to speak to more or less mobility. As is frequent practice in the statistical analysis of isotopic data in archaeology, we assume that outliers result in non-normal distributions of the data. As non-parametric tests use few assumptions, we also report those tests approaching significance or those that are close to but slightly above *p*=0.05. Thus, we used the Mann-Whitney U test when comparing the results by biological sex and the Kruskal-Wallis between the family groupings. Infants showing a weaning signature by having elevated nitrogen levels were excluded from the analysis (1 for Nitra, and 3 for Polgár-Ferenci-hát).

**Nitra Horné Krškany**

A total of 45 individuals from Nitra Horné Krškany produced reliable results for aDNA and carbon, nitrogen, and strontium isotope ratios. Not all of these 45 individuals had both strontium and stable isotope values, mainly due to no teeth being preserved, but in some cases because of poor collagen preservation of the ribs which were sampled for the stable isotope analysis. This meant that some comparisons could not be carried out (Supplementary Tables 14-16).

The published isotopic results from Nitra Horné Krškany suggested a small but significantly elevated average nitrogen isotope ratio for male adults over the rest of the population , potentially suggesting a high protein or meat content in their diet. Adult females had significantly more variable strontium isotope ratios than men and accounted for all the outliers [^101^](https://paperpile.com/c/2vFjDD/vnhYW).

* Significant (*p*<0.05)

** Approaching significance (following Lifeways 0.05<*p*<0.1)

**Supplementary Table 14:** Levene’s test for significance difference in variance in Nitra Horné Krškany

| Groups compared | Isotope | N | Levene statistic | Sig. |
| --- | --- | --- | --- | --- |
| Related (all) and unrelated (all) | Sr ratio | 34 | 0.01 | 0.975 |
|  | Sr conc | 32 | 0.279 | 0.601 |
|  | δ^13^C | 35 | 2.328 | 0.137 |
|  | δ^15^N | 34 | 0.326 | 0.572 |
| Related males and females | Sr ratio | 20 | 3.121 | 0.093** |
|  | Sr conc | 20 | 1.516 | 0.233 |
|  | δ^13^C | 25 | 0.1.232 | 0.278 |
|  | δ^15^N | 25 | 0.172 | 0.682 |
| Unrelated males and females | Sr ratio | 12 | 1.466 | 0.249 |
|  | Sr conc | 10 | 4.707 | 0.055** |
|  | δ^13^C | - |  |  |
|  | δ^15^N | - |  |  |
| Related females and unrelated females | Sr ratio | 20 | 0.164 | 0.689 |
|  | Sr conc | 19 | 0.414 | 0.528 |
|  | δ^13^C | 23 | 3.188 | 0.087** |
|  | δ^15^N | 23 | 2.832 | 0.095** |
| Related males and unrelated males | Sr ratio | 12 | 0.002 | 0.966 |
|  | Sr conc | 11 | 13.942 | 0.003* |
|  | δ^13^C | - |  |  |
|  | δ^15^N | - |  |  |
| Related males and unrelated females | Sr ratio | 18 | 3.702 | 0.07** |
|  | Sr conc | 17 | 1.151 | 0.298 |
|  | δ^13^C | 18 | 0.179 | 0.677 |
|  | δ^15^N | 17 | 0.666 | 0.426 |
| Related females and unrelated males | Sr ratio | 14 | 1.208 | 0.290 |
|  | Sr conc | 13 | 0.544 | 0.474 |
|  | δ^13^C | - |  |  |
|  | δ^15^N | - |  |  |
| Family grouping | Sr ratio | 12 | 9.220 | <0.001* |
|  | Sr conc | 12 | 6.957 | 0.003* |
|  | δ^13^C | 16 | 1.374 | 0.277 |
|  | δ^15^N | 16 | 2.017 | 0.106 |

**Tests with significant results (***p<0.05***):**

1. Unrelated males and related males had significant variation in strontium concentration (*p*=0.003). This would be consistent with unrelated males having more variable origins in childhood.
2. Variance in family groupings is not equal across Sr isotope ratio (*p*<0.001) and Sr concentration (*p*=0.003). This suggests that “families” did not share the same mobility patterns.

**Supplementary Table 15:** Mann-Whitney U tests for difference in means in Nitra Horné Krškany

| Groups compared | Isotope | N | Mann-Whitney U | Sig. |
| --- | --- | --- | --- | --- |
| Related (all) and unrelated (all) | Sr ratio | 36 | 148.5 | 0.86 |
|  | Sr conc | 34 | 126.5 | 0.845 |
|  | δ^13^C | 37 | 134 | 0.987 |
|  | δ^15^N | 36 | 106 | 0.59 |
| Related males and female | Sr ratio | 22 | 40.5 | 0.203 |
|  | Sr conc | 22 | 66 | 0.722 |
|  | δ^13^C | 27 | 118.5 | 0.134 |
|  | δ^15^N | 27 | 112 | 0.251 |
| Unrelated males and females | Sr ratio | 14 | 10 | 0.188 |
|  | Sr conc | 12 | 11 | 0.727 |
|  | δ^13^C | 10 | 0 | 0.2 |
|  | δ^15^N | 9 | 8 | 0.222 |
| Related females and unrelated females | Sr ratio | 22 | 59.5 | 0.974 |
|  | Sr conc | 21 | 53 | 0.972 |
|  | δ^13^C | 25 | 59.5 | 0.487 |
|  | δ^15^N | 24 | 71 | 0.697 |
| Related males and unrelated males | Sr ratio | 14 | 28 | 0.304 |
|  | Sr conc | 13 | 19 | 0.593 |
|  | δ^13^C | 12 | 10 | 0.333 |
|  | δ^15^N | 12 | 1 | 0.333 |
| Related Males and unrelated females | Sr ratio | 20 | 34 | 0.247 |
|  | Sr conc | 19 | 44.5 | 0.968 |
|  | δ^13^C | 20 | 50.5 | 0.941 |
|  | δ^15^N | 19 | 65 | 0.091** |
| Related females and unrelated males | Sr ratio | 22 | 59.5 | 0.974 |
|  | Sr conc | 21 | 53 | 0.972 |
|  | δ^13^C | 25 | 59.5 | 0.487 |
|  | δ^15^N | 24 | 71 | 0.697 |

**Supplementary Table 16:** Kruskal-Wallis test to compare multiple groups in Nitra Horné Krškany

| Groups compared | Isotope | N | Kruskall-Wallis test statistic | Sig. |
| --- | --- | --- | --- | --- |
| Family groups | Sr ratio | 22 | 11.098 | 0.196 |
|  | Sr conc | 22 | 8.808 | 0.359 |
|  | δ^13^C | 26 | 17.208 | 0.046* |
|  | δ^15^N | 26 | 10.135 | 0.34 |

**Tests with significant results:**

- Family groupings had different average carbon values to each other although the significance (*p*=0.046) is not compelling after correcting for multiple hypothesis testing. This may suggest that they were sourcing their food from different locations in the landscape.

Overall, the most notable observation is that family groupings are not unified in diet and mobility, and are significantly different in variation from each other.

**Polgár-Ferenci-hát**

A total of 47 individuals from Pólgar-Ferenci-hát produced reliable results for carbon and nitrogen and 34 for strontium isotope analysis. Only 50% of the burials were analysed for isotopes by the LBK lifeways project. In total, only 23 burials had both isotope and aDNA data, 17 related (6 females, 11 males) and 6 unrelated (5 females, 1 male). Due to the low numbers of unrelated individuals, comparing the family groupings was the main focus of the analysis (Supplementary Tables 17-19).

In the published work, the main isotopic results from Pólgar-Ferenci-hát found no evidence of systematic dietary differences suggested between the sexes, though women’s nitrogen values increased as they aged. Adult females had significantly more variable strontium isotope ratios than men, though this was perhaps less pronounced than at other sites. It was hypothesised based on variation in strontium isotopes between the molars that sex-based dietary differences became more pronounced between the ages of 8-12 [^101^](https://paperpile.com/c/2vFjDD/vnhYW).

* Significant (*p*<0.05)

** Approaching significance (following Lifeways 0.05<*p*<0.1)

**Supplementary Table 17:** Levene’s test for significance difference in variance in Polgár-Ferenci-hát.

| Groups compared | Isotope | N | Levene statistic | Sig. |
| --- | --- | --- | --- | --- |
| Related (all) and unrelated (all) | Sr ratio | 15 | 0.33 | 0.574 |
|  | Sr conc | 15 | 2.035 | 0.174 |
|  | δ^13^C | 21 | 1.244 | 0.277 |
|  | d15N | 18 | 0.19 | 0.893 |
| Family grouping | Sr ratio | 12 | 16.747 | 0.001* |
|  | Sr conc | 12 | 16.207 | 0.002* |
|  | δ^13^C | 16 | 21.767 | <0.001* |
|  | δ^15^N | 16 | 3.667 | 0.047* |

**Tests with significant results:**

- Family groupings all have different variances to each other across all isotopes.

**Supplementary Table 18:** Mann-Whitney U tests for difference in means in Polgár-Ferenci-hát.

| Groups compared | Isotope | N | Mann-Whitney U | Sig. |
| --- | --- | --- | --- | --- |
| Related (all) and unrelated (all) | Sr ratio | 17 | 32.5 | 0.792 |
|  | Sr conc | 17 | 42 | 0.204 |
|  | δ^13^C | 23 | 40.5 | 0.460 |
|  | δ^15^N | 20 | 47 | 0.404 |

**Supplementary Table 19:** Kruskal-Wallis test to compare multiple groups in Polgár-Ferenci-hát.

| Groups compared | Isotope | N | Kruskall-Wallis test statistic | Sig. |
| --- | --- | --- | --- | --- |
| Family groups | Sr ratio | 12 | 3.007 | 0.391 |
|  | Sr conc | 12 | 1.157 | 0.763 |
|  | δ^13^C | 17 | 4.994 | 0.172 |
|  | δ^15^N | 15 | 1.451 | 0.694 |

**Conclusions for Pólgar-Ferenci-hát:**

- Family groupings are significantly different in variation from each other for mobility and diet, similar to the pattern at Nitra Horné Krškany. (Supplementary tables 18-20)

**Section 4: Supplementary Methods for Ancient DNA Analysis**

**Laboratory procedures**

We generated powder from the skeletal remains of all individuals that are listed in Supplementary Tables 1-2. Supplementary Table 2 shows the list and details of the individuals and generated libraries. The powder was produced from the cochlea [^102^](https://paperpile.com/c/2vFjDD/KFmap), ossicles [^103^](https://paperpile.com/c/2vFjDD/vVqKd), or teeth in clean rooms at the University College Dublin or the University of Vienna.

We extracted DNA in dedicated ancient DNA laboratories at Harvard Medical School or the University of Vienna, following published protocols [^104,105^](https://paperpile.com/c/2vFjDD/B0emc+y1U6A). Double-stranded libraries were prepared from the extracts, using either dual-barcoded double-stranded libraries [^106^](https://paperpile.com/c/2vFjDD/Rt7Fu) or dual-indexed single-stranded libraries [^107^](https://paperpile.com/c/2vFjDD/8OeA8), both treated with uracil-DNA glycosylase (UDG) to reduce the rate of ancient DNA damage [^108^](https://paperpile.com/c/2vFjDD/Svsim). Double-stranded libraries were treated in a modified partial UDG preparation (‘half’), leaving a reduced damage signal at both ends (5′ C-to-T, 3′ G-to-A). For some individuals with little success, we made more than one library per extract. The list of the libraries generated in this study is presented in Supplementary Table 2.

The newly produced libraries were captured with in-solution target hybridisation to enrich sequences that overlap the mitochondrial genome and about 1.24 million genome-wide SNPs [^109–112^](https://paperpile.com/c/2vFjDD/Bw5ma+FtgOj+MaYt2+rLX9g) (‘1240K’). Then, captured libraries were indexed with two seven-base-pair indexing barcodes to the adapters of each double-stranded library. The indexed and pooled libraries were sequenced in an Illumina NextSeq500 instrument with 2 × 76 cycles or an Illumina HiSeqX10 instrument with 2 × 101 cycles and reading the indices with 2 × 7 cycles (double-stranded libraries).

After sequencing, paired-end libraries were merged. Before alignment, we merged paired-end sequences, retaining reads that exhibited no more than one mismatch between the forward and reverse base if the base quality was ≥20, or 3 mismatches if the base quality was <20. A custom toolkit (available at https://github.com/DReichLab/ADNA-Tools) was used to merge and trim adapters and barcodes. The merged reads were aligned with BWA samse v.0.7.15-r114053 [^113^](https://paperpile.com/c/2vFjDD/Rxwsd) using typical aDNA parameters (-n 0.01, -o 2, and -l 16500) to the reconstructed human mtDNA consensus sequence (RSRS)[^114^](https://paperpile.com/c/2vFjDD/Ps4cf) and the human reference genome version hg19. We removed duplicates with Picard MarkDuplicates tool [^115^](https://paperpile.com/c/2vFjDD/TcdYb). After this, we trimmed two terminal bases from UDG-half libraries to reduce damage-induced errors.

To discard contaminated samples, we discarded libraries with less than 3% cytosine-to-thymine substitutions at the end of the sequenced fragments. and point estimates of mitochondrial DNA (mtDNA) contamination below 5% using contamMix v.1.0-1248, and point estimates of X chromosome contamination (in males) below 3%. We also used contamLD to confirm low contamination rates (less than about 6%). The results are presented in Supplementary Table 1. For SNP calling, we randomly sampled an overlapping read with a minimum mapping quality of ≥10 and a base quality of ≥20. Individuals with <30,000 covered SNPs were excluded from quantitative analyses. For first-degree relatives, we always excluded the individual with less coverage from the pair in all the population genetics analyses: qpAdm, PCA, f-statistics, and DATES.

**Bioinformatics**

Genetic data were merged with published datasets of Early Neolithic and Mesolithic individuals [^38,39,111,116–119^](https://paperpile.com/c/2vFjDD/BE1Yv+gXzgO+Yzi14+MaYt2+Gqr9A+d3Y7Y+EBkKz). We excluded from the analyses all individuals with a 1st degree relative, showing clear signs of human contamination, or with low coverage (less than 30,000 SNPs in the autosomes).

We used Principal Components Analysis (PCA) (Extended Figure1, Supplementary Figure 22) with the smartpca package of EIGENSOFT to graphically represent the individuals and the relationship between them and to position the individuals within the main axis of ancestry (WHG and Balkan_N). The PCA was performed with 879 modern individuals from the following populations that represent the western-Eurasian diversity:Abkhasian, Adygei, Albanian, Armenian, Balkar, Basque, BedouinA, BedouinB, Belarusian, Bulgarian, Chechen, Croatian, Cypriot, Czech, Druze, English, Estonian, Finnish, French, Georgian, Greek, Hungarian, Icelandic, Iranian, Italian_North, Italian_North.DG, Italian_South, Jordanian, Kumyk, Lebanese, Lezgin, Lithuanian, Maltese, Mordovian, North_Ossetian, Norwegian, Orcadian, Palestinian, Russian, Sardinian, Saudi, Scottish, Sicilian, Spanish, Spanish_North, Syrian, Turkish, Ukrainian. The genotypes cover the 597,573 of the Human Origins dataset accessible through

(<https://reichdata.hms.harvard.edu/pub/datasets/amh_repo/curated_releases/V54/V54.1.p1/SHARE/public.dir/v54.1.p1_HO_public.tar>) We used f-statistics to compare the relationship between groups of individuals using admixtools 7.0.2 [^120^](https://paperpile.com/c/2vFjDD/EvHav) .

We ran qpWave, grouping all the individuals from the same culture and location. This strategy was used to describe genetic outliers using the strategy described in Patterson et al., 2022[^121^](https://paperpile.com/c/2vFjDD/Y0ADE) . The Körös and Starčevo included in Patterson et al., 2022 as WHGA (I1507, I4971) or BalkanN (I0174, I1876, I1508, I2794) were used among the source populations, and therefore, we do not present qpWave results of Köros and Starcevo, we also do not present results of qpAdm for these individuals as they were used as sources. Individuals with a p-value > 0.05 were labelled as outliers. The results are presented in Extended Figure 2.

DATES 3600 [^122^](https://paperpile.com/c/2vFjDD/exOV) was used to date the admixture events detected in the qpAdm analyses using Balkan_N and WHGA as source populations, using default parameters and a generation time of 28 years.

We imputed our data following the same imputation threshold and strategy already extensively tested and validated and published in Ringbauer et al., 2024[^124^](https://paperpile.com/c/2vFjDD/oe44t) . We started by imputingthe calls of the individuals with more than 600,000 recovered SNPS of the 1240k dataset using GLIMPSE 2 [^123^](https://paperpile.com/c/2vFjDD/qzZxq), a software that has been demonstrated to perform correctly with low-coverage data. Following the same methodology described in Ringbauer et al., 2024[^124^](https://paperpile.com/c/2vFjDD/oe44t) we first generated genotype probabilities using bcftools mpileup (v1.10.2) [^125^](https://paperpile.com/c/2vFjDD/72Sef), which were the GLIMPSE input and VCF imputed files were obtained. We used VCF and TSV files generated from the 1000Genome Phase 3 dataset as references for the imputation. We imputed at 78,397,683 SNPs (bi-allelic SNP sites from the 1000 Genome Project dataset). We then analyzed a subset of 6,237,558 high-quality SNPs (based on average genotype posterior probabilities greater than 0.9). We used ancIBD to infer Identity By Descent (IBD) segments on the imputed dataset comprising 182 previously and newly reported Neolithic individuals used for the analysis. The methodology described in Ringbauer et al., 2024[^124^](https://paperpile.com/c/2vFjDD/oe44t) was used for IBD reconstruction from the diploid imputed data.

For the local ancestry analyses, we selected the 6,237,558 quality autosomal SNPs present in the imputation panel. We also selected all the ALPC individuals with an imputation quality score (IQS) > 0.8. A total of 79 passed this filter (Supplementary Table 5). The focus on ALPC aims to understand specific patterns of admixture of this admixed population. These 79 phased genomes were analysed with RFMix 2.03 [^126^](https://paperpile.com/c/2vFjDD/rmjda), setting 16 generations of admixture time and 2 EM interactions, -c 0,2 cM, -G 16, -t 500, and the rest with default parameters. Chromosomes were collapsed and plotted, accepting sites with >0.9 probability. We used the output bed files from RFMix to isolate the WHG ancestry with Plink 1.9 and subtract the WHG fragments from the pseudo-haploid calls. We used these data to perform f-statistics analyses with admixtools 7.0.2.

We calculated the average WHG ancestry of ALPC genomes in chunks of 0.2 cM and added this information to the selection scan results (Supplementary Table 11). We calculated the correlation between the ancestry predicted by qpAdm and RFMix using a regression test with R (Supplementary Figure 24).

**Grave Distances Calculation**

The distances between graves in the Nitra and Polgár-Ferenci-hát cemeteries were calculated using ImageJ and R. Pixel coordinates of each grave were recorded in ImageJ from Figure 3. These data were then used to compute the relative distances between each individual. These calculations were performed in R.

**Section 5: Classification of individuals with genetic methods**

**Authors**: Pere Gelabert

From the PCA (Extended Figure 1, Supplementary Figure 22) three outliers were obvious, two labelled as ALPC. Individual (I1877) is almost entirely of Early European Farmer (EEF) ancestry (95%), one Starčevo individual has substantial WHG ancestry (I1876) (18%) [^39^](https://paperpile.com/c/2vFjDD/Yzi14) and one labelled as Austria_LBK, with 51% WHG ancestry (I6914)[^116^](https://paperpile.com/c/2vFjDD/gXzgO). Nevertheless, we used qpWave to systematically determine the outliers (Extended Figure 2). Those that show consistent deviation from the general cluster are labelled EXC in Supplementary Table 1.

Previous ancient DNA studies of the Körös and Starčevo archaeological cultures [^39^](https://paperpile.com/c/2vFjDD/Yzi14) documented that members of these communities varied in their degree of admixture with local hunter-gatherers and, thus, were far from genetically homogeneous. Given small sample sizes, however, it was impossible to identify cultural features associated with those people more likely to have experienced this admixture. Pooling the individuals from each culture and excluding those with elevated WHG ancestry (excluding the two full WHG individuals I1507 and I4971), we found that the statistic *f_4_*(Turkey N, WHG; Körös, Starčevo) has a value of 0.0002 (Z=1.89) suggesting no differential presence of WHG between Körös and Starčevo. To further explore these relationships, we applied qpAdm to model the Körös genomes using Balkan_N and WHGA as possible sources after the removal of the Körös and Starčevo individuals from the Balkan_N set. Both populations can be modelled with Balkan_N genomes (*p*>0.01). Four Körös individuals have higher than average WHG values: I18642 has 11%, I17931 has 11%, I2373 has 9%, and I4971 is an unadmixed WHG. Additionally, we report two Starčevo individuals with higher-than-average WHG values: I4918 with 7% and I6699 with 19% (Supplementary Table 1).

We tested for differences in the WHG source populations for the different European farmer groups from a far western European context (Loschbour from Luxembourg) or a central European context (KO1: an individual with WHG ancestry found at a Koros site), using the statistic *f_4_*(Mbuti, X; Loschbour, KO1). This statistic is sensitive to differences in rates of allele sharing of a test population with the two important sources for WHG ancestry in Europe. We performed these analyses at a populational level, excluding the individuals with elevated WHG ancestry, labelled HGEXC in Supplementary Table 1. Neither result was statistically significantly asymmetric at the |Z|>3 significance level. We then used the statistic *f_4_*(Mbuti, X; Körös, Starčevo) to test the previously suggested hypothesis [^127^](https://paperpile.com/c/2vFjDD/tcRR5) that the ALPC derived ancestry from Körös while the LBK derived Starčevo ancestry (Supplementary Table 21). The results do not reveal any significant asymmetries in the sense that none of the comparisons yields a Z-score > |3|.

To obtain further insight into the WHG ancestry, we generated diploid genotype calls for the admixed ALPC individuals with imputation scores > 0.8 (79 individuals) using the software GLIMPSE 2 [^123^](https://paperpile.com/c/2vFjDD/qzZxq), and performed a local ancestry-segmentation analysis using RFMix 2.03 [^126^](https://paperpile.com/c/2vFjDD/rmjda), aiming to detect segments that are likely to derive from WHG (Supplementary Figure 23). We also explored if *f_4_* tests on the WHG segments would give more resolution regarding the origin of the WHG in the ALPC, but this was not so (Supplementary Table 20).

**Supplementary Table 20:** relevant *f_4_* results of the analysis.

| *f_4_* results in the combination *f_4_*(Mbuti,X;WHG,BalkanN) | | | | | | | |
| --- | --- | --- | --- | --- | --- | --- | --- |
| Mbuti | X | PopA | PopB | Estimate | SD | D-score | SNPs used |
| Mbuti | Starcevo | WHGA | BalkanN | 0.01 | 0.00 | 27.47 | 1061664 |
| Mbuti | Koros | WHGA | BalkanN | 0.01 | 0.00 | 51.42 | 1017813 |
| Mbuti | Germany_LBK | WHGA | BalkanN | 0.01 | 0.00 | 27.34 | 1064095 |
| Mbuti | Transdanubia_LBK | WHGA | BalkanN | 0.01 | 0.00 | 24.73 | 1063954 |
| Mbuti | Transdanubia_LBK-HGEXC | WHGA | BalkanN | 0.01 | 0.00 | 27.71 | 1052001 |
| Mbuti | Slovakia_LBK | WHGA | BalkanN | 0.00 | 0.00 | 12.42 | 374722 |
| Mbuti | Austria_LBK | WHGA | BalkanN | 0.01 | 0.00 | 27.31 | 1064094 |
| Mbuti | Austria_LBK-HGEXC | WHGA | BalkanN | 0.00 | 0.00 | 2.82 | 193285 |
| Mbuti | Hungary_ALPC | WHGA | BalkanN | 0.00 | 0.00 | 19.40 | 1063300 |
| Mbuti | Hungary_ALPC-HGEXC | WHGA | BalkanN | 0.01 | 0.00 | 14.12 | 220005 |
| Mbuti | Hungary_ALPC-EEFEXC | WHGA | BalkanN | 0.01 | 0.00 | 23.54 | 1045518 |
| *f_4_* results in the combination *f_4_*(Mbuti,X;Loschbour,KO1) | | | | | | | |
| Mbuti | X | PopA | PopB | Estimate | SD | D-score | SNPs used |
| Mbuti | Starcevo | Loschbour | Koros-HG | 0.00 | 0.00 | -1.49 | 324265 |
| Mbuti | Koros | Loschbour | Koros-HG | 0.00 | 0.00 | -1.80 | 321865 |
| Mbuti | Germany_LBK | Loschbour | Koros-HG | 0.00 | 0.00 | -1.55 | 324302 |
| Mbuti | Transdanubia_LBK | Loschbour | Koros-HG | 0.00 | 0.00 | -0.46 | 324299 |
| Mbuti | Transdanubia_LBK-HGEXC | Loschbour | Koros-HG | 0.00 | 0.00 | -1.78 | 324090 |
| Mbuti | Slovakia_LBK | Loschbour | Koros-HG | 0.00 | 0.00 | 0.80 | 132147 |
| Mbuti | Austria_LBK | Loschbour | Koros-HG | 0.00 | 0.00 | -1.35 | 324302 |
| Mbuti | Austria_LBK-HGEXC | Loschbour | Koros-HG | 0.00 | 0.00 | -2.34 | 69456 |
| Mbuti | Hungary_ALPC | Loschbour | Koros-HG | 0.00 | 0.00 | -2.19 | 324298 |
| Mbuti | Hungary_ALPC-HGEXC | Loschbour | Koros-HG | 0.00 | 0.00 | -0.36 | 76403 |
| Mbuti | Hungary_ALPC-EEFEXC | Loschbour | Koros-HG | 0.00 | 0.00 | -2.27 | 323908 |
| *f_4_* results in the combination *f_4_*(Mbuti,X;Koros,Starcevo) | | | | | | | |
| Mbuti | X | PopA | PopB | Estimate | SD | D-score | SNPs used |
| Mbuti | Germany_LBK | Koros | Starcevo | 0.00 | 0.00 | 1.72 | 1034957 |
| Mbuti | Transdanubia_LBK | Koros | Starcevo | 0.00 | 0.00 | 0.99 | 1034867 |
| Mbuti | Transdanubia_LBK-HGEXC | Koros | Starcevo | 0.00 | 0.00 | 1.19 | 1026807 |
| Mbuti | Slovakia_LBK | Koros | Starcevo | 0.00 | 0.00 | 1.11 | 372606 |
| Mbuti | Austria_LBK | Koros | Starcevo | 0.00 | 0.00 | 0.72 | 1034956 |
| Mbuti | Austria_LBK-HGEXC | Koros | Starcevo | 0.00 | 0.00 | 0.43 | 192506 |
| Mbuti | Hungary_ALPC | Koros | Starcevo | 0.00 | 0.00 | 1.36 | 1034451 |
| Mbuti | Hungary_ALPC-HGEXC | Koros | Starcevo | 0.00 | 0.00 | -0.32 | 218524 |
| Mbuti | Hungary_ALPC-EEFEXC | Koros | Starcevo | 0.00 | 0.00 | 2.17 | 1021343 |
| *f_4_* results in the combination *f_4_*(Mbuti,ALPC-LOCALWHG;POP1,POP2) with the data from the RFMix analyses | | | | | | | |
| Mbuti | X | PopA | PopB | Estimate | SD | D-score | SNPs used |
| Mbuti.DG | ALPC-WHG | Koros-HG | Starcevo-HG | 0.00 | 0.00 | -0.97 | 1655 |
| Mbuti.DG | ALPC-WHG | Koros | Starcevo | 0.00 | 0.00 | 0.31 | 766352 |
| Mbuti.DG | ALPC-WHG | Loschbour | KO1 | 0.00 | 0.00 | -0.84 | 218729 |

**
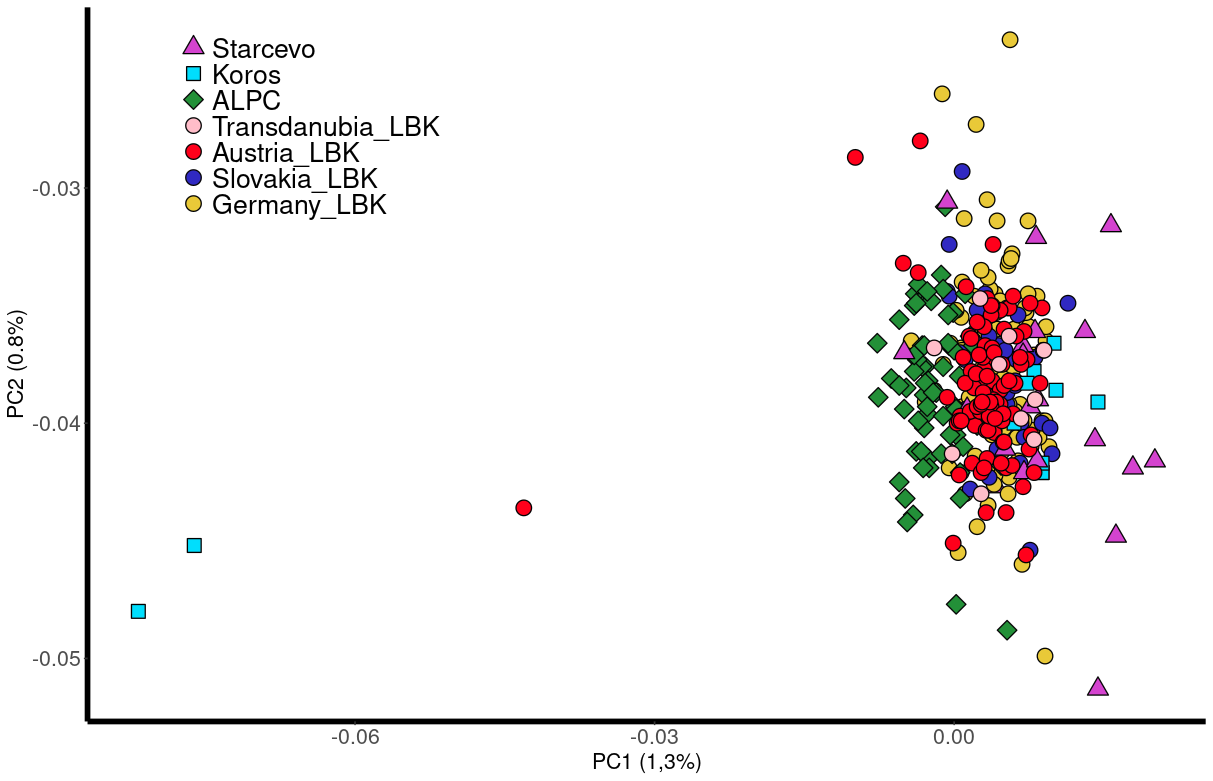
**

**Supplementary Figure 22:** Principal components analyses of the Neolithic dataset without the modern populations (Extended Figure 1) that were used to perform the PCA.


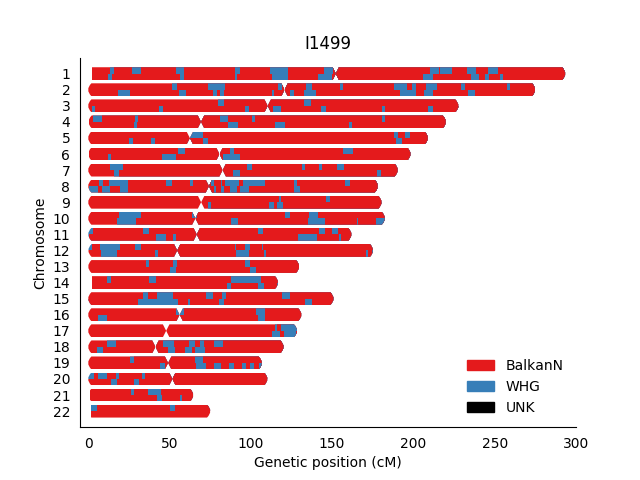

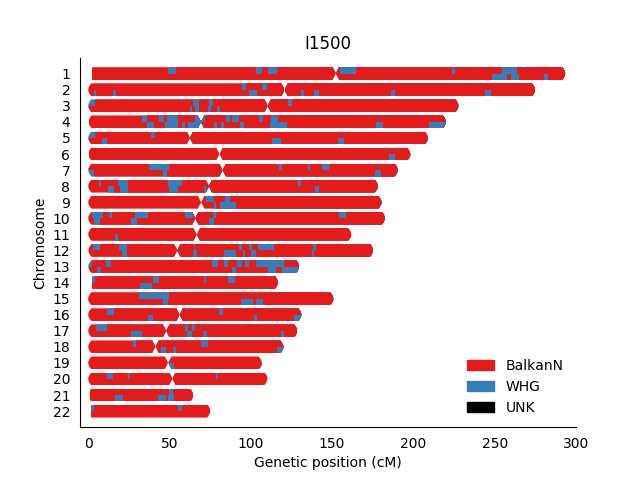

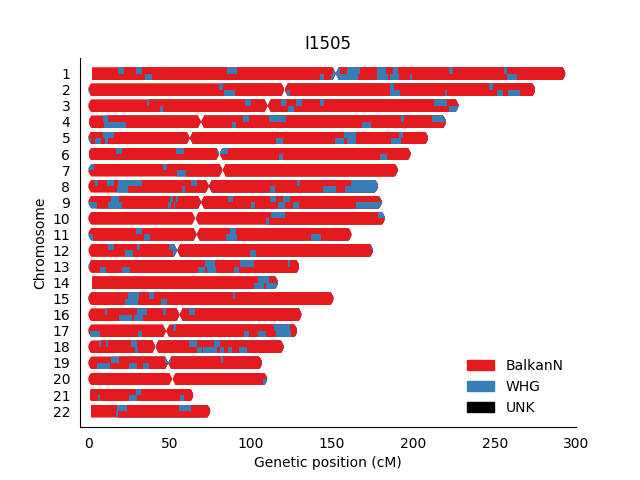

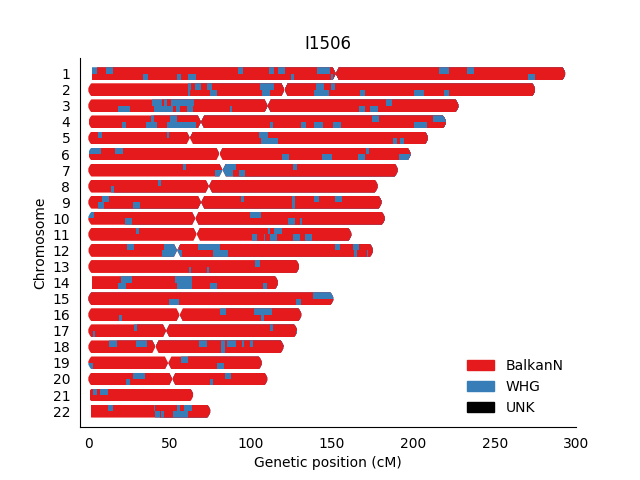

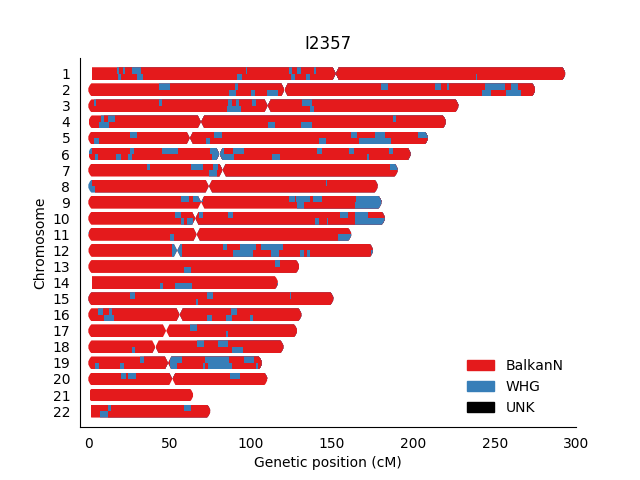

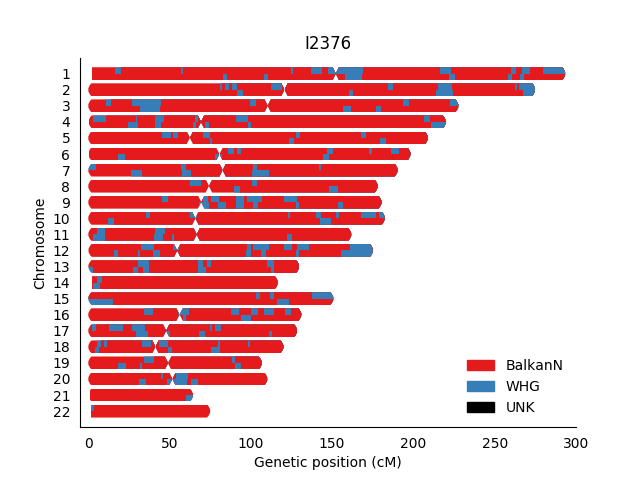

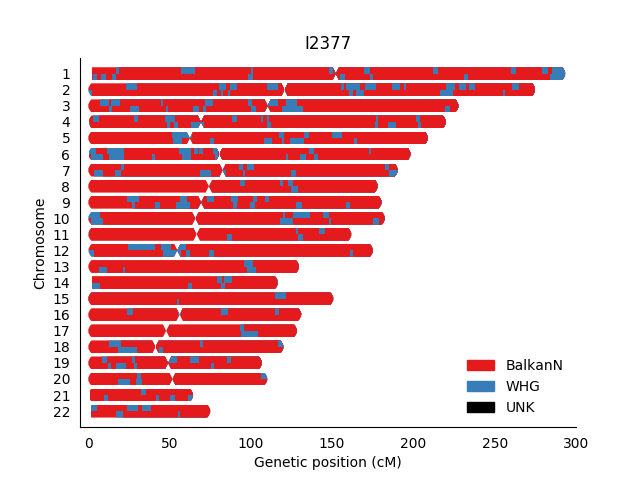

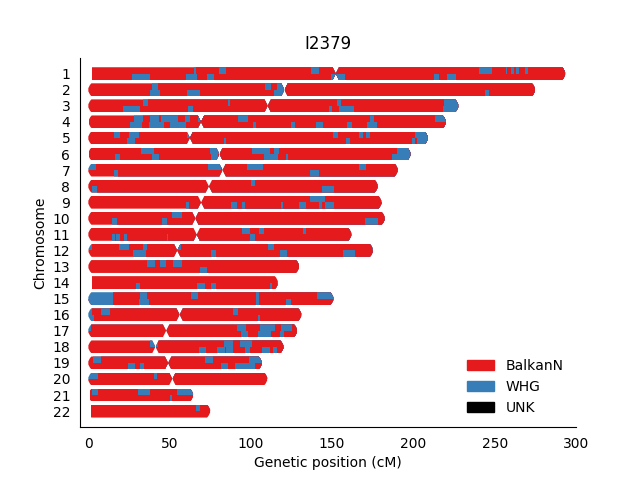

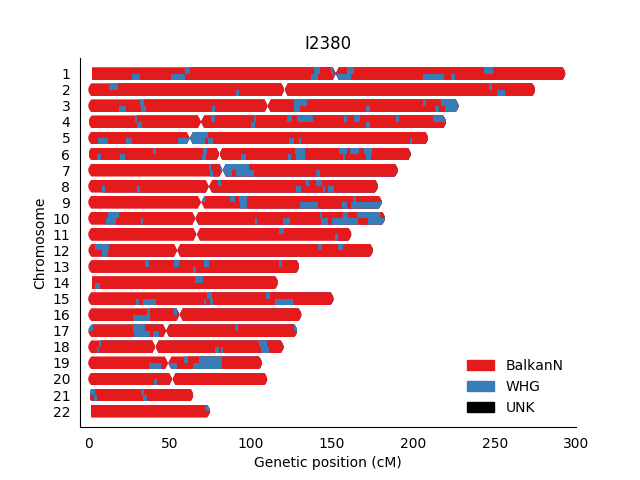

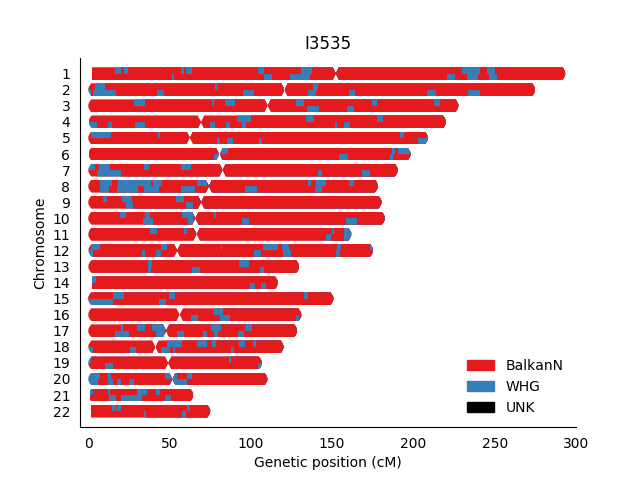

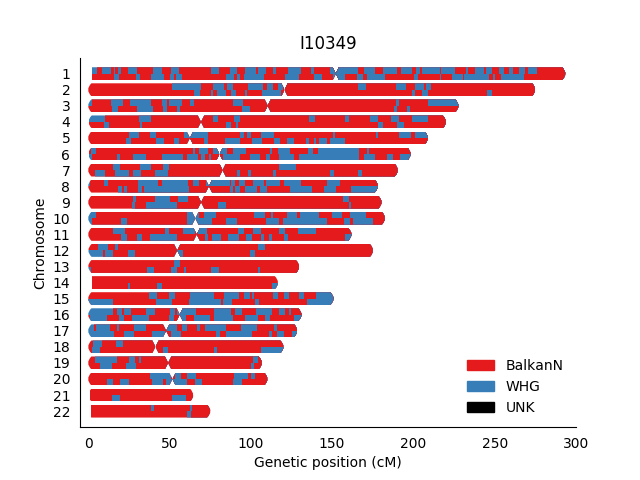

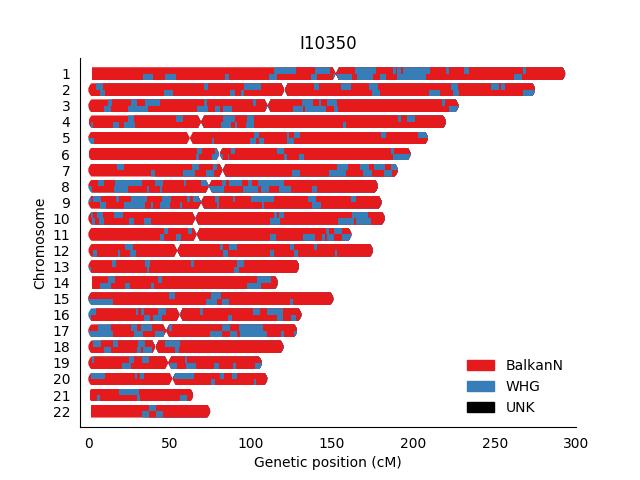

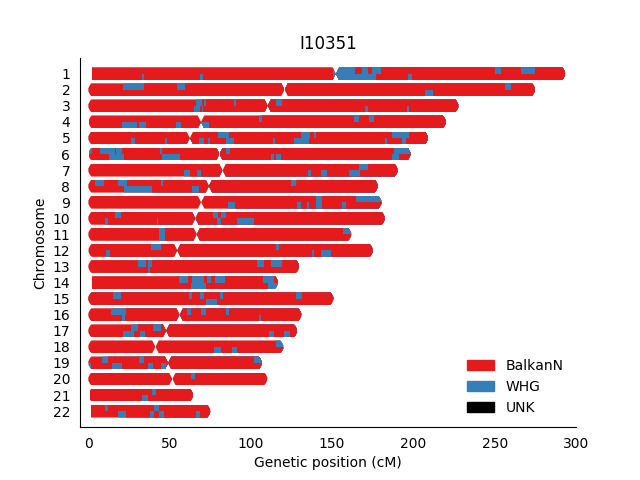

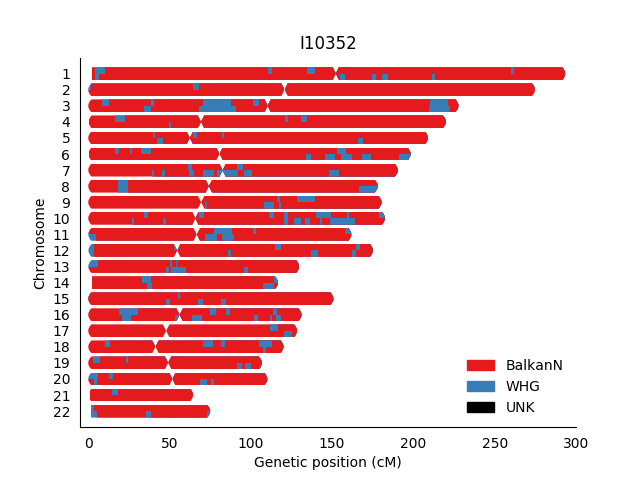

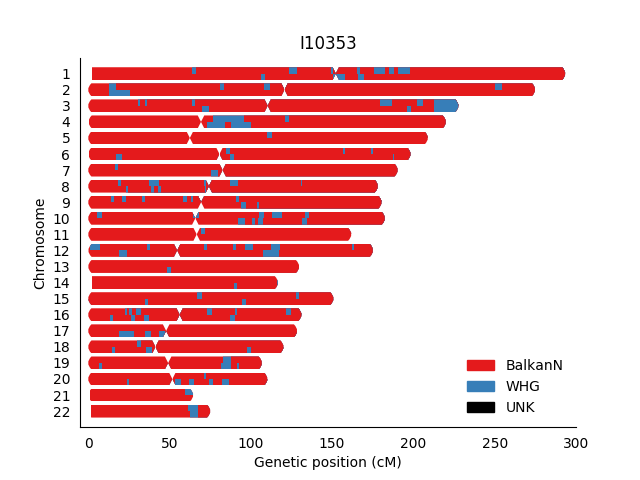

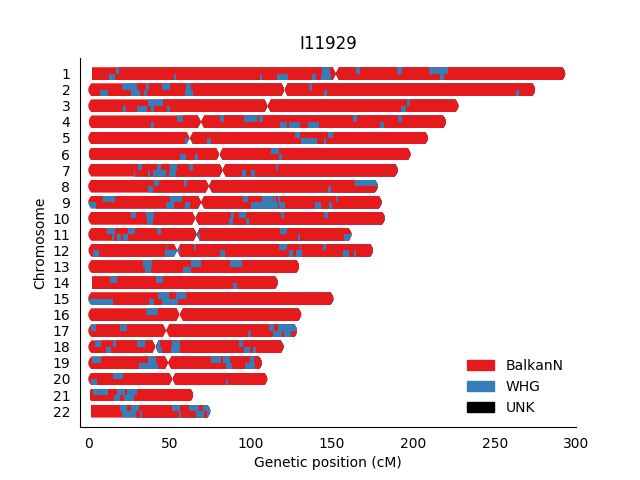

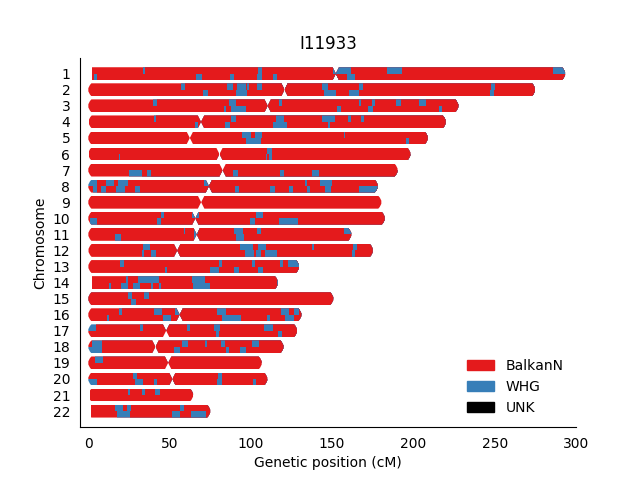

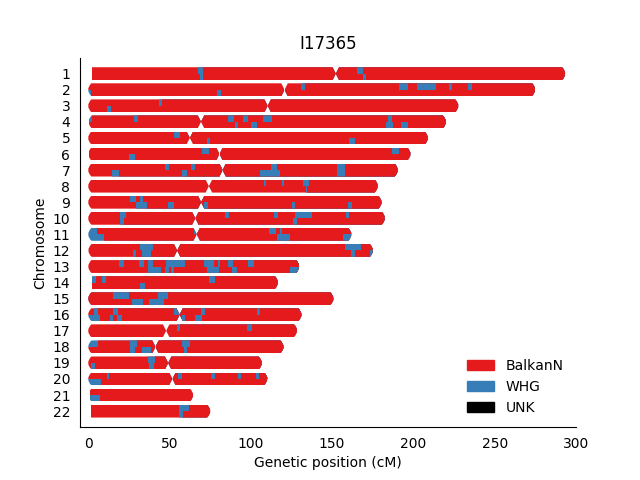

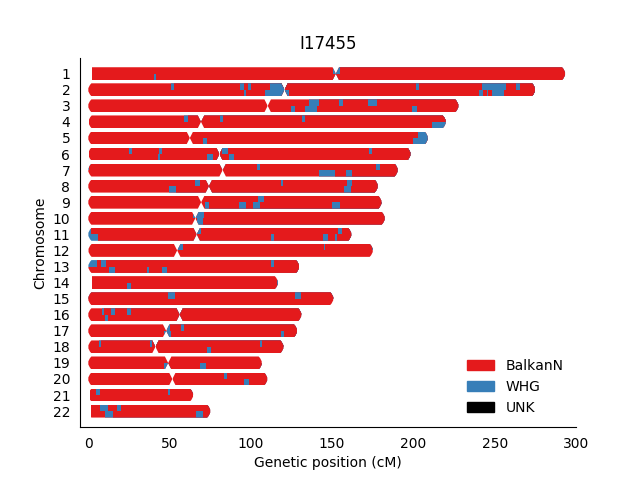

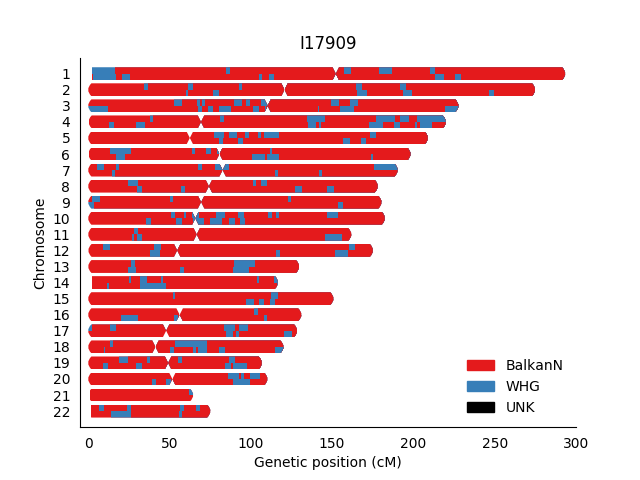

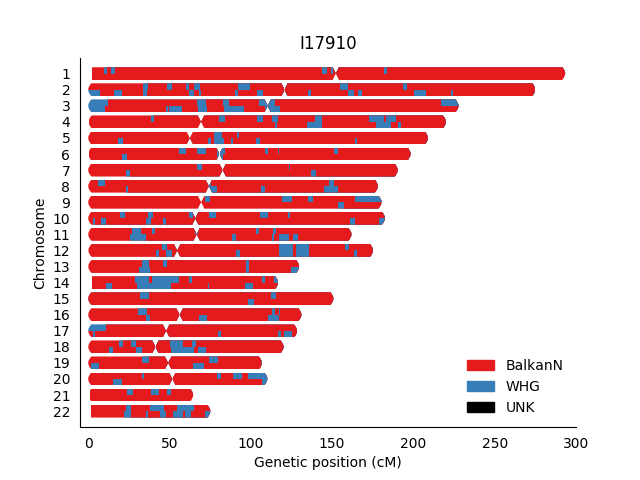

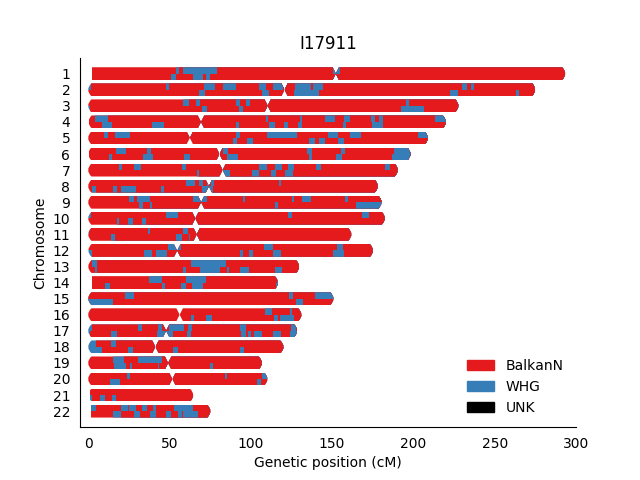

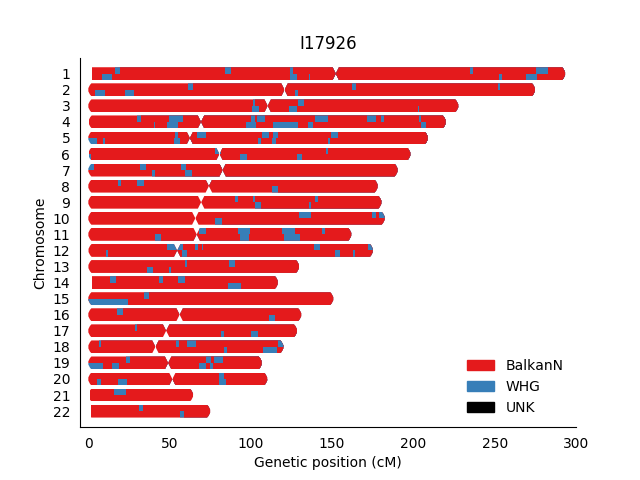

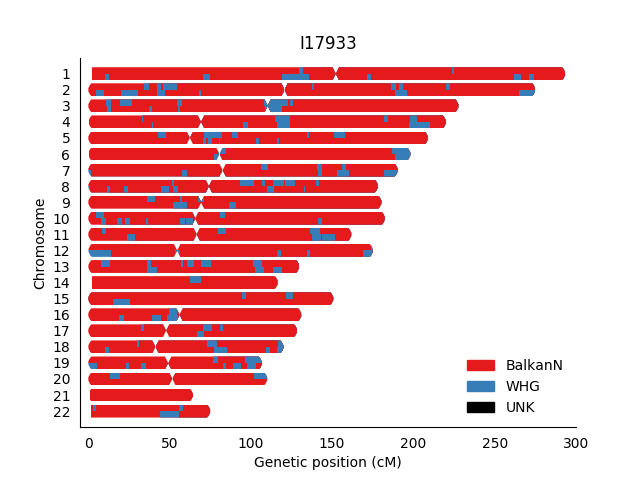

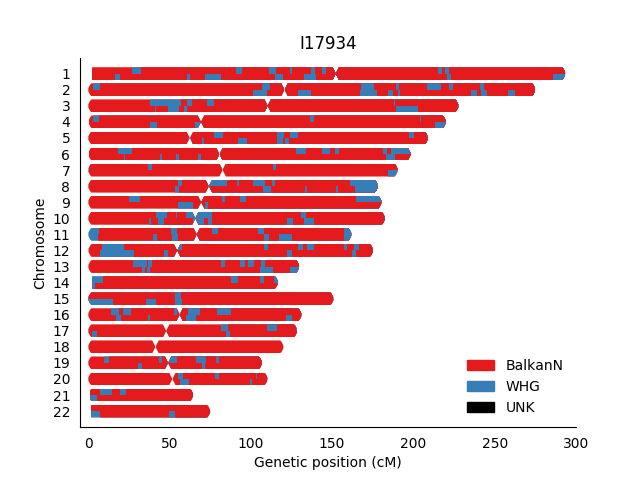

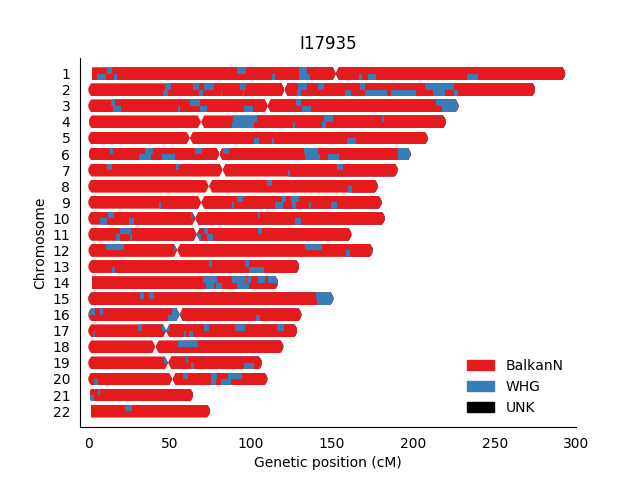

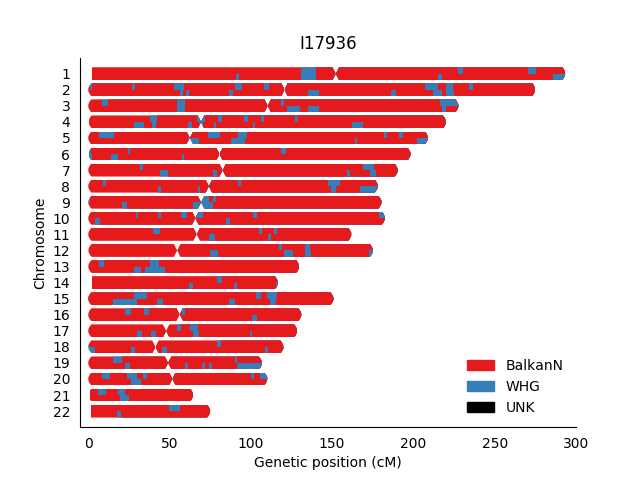

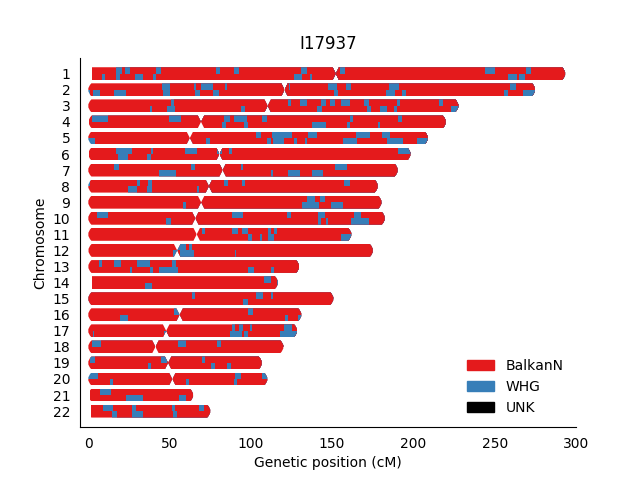

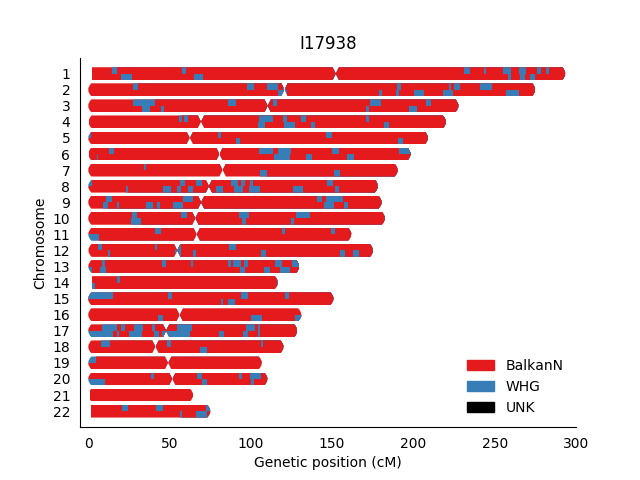

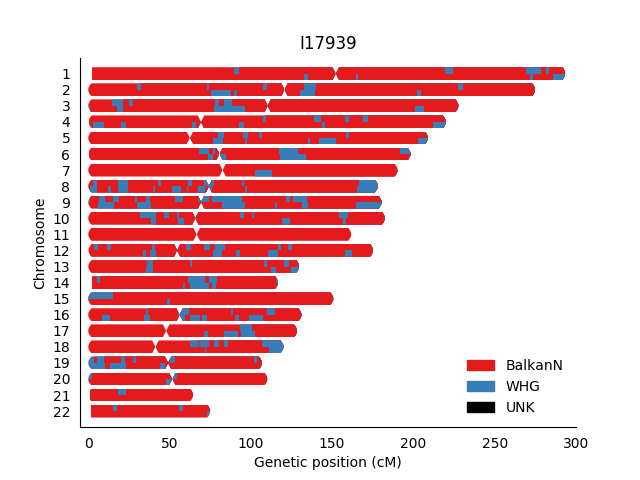

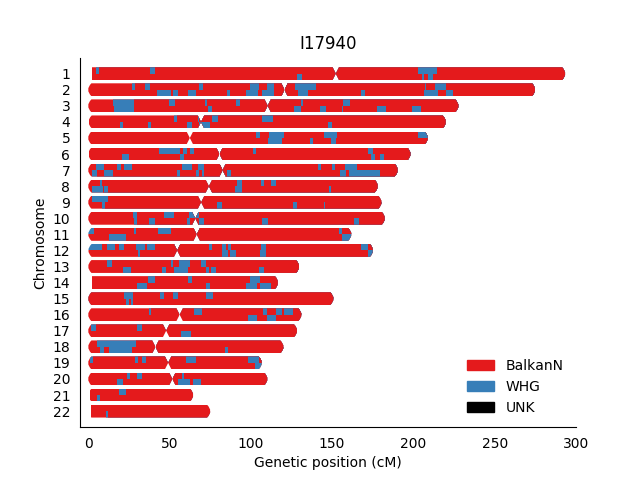

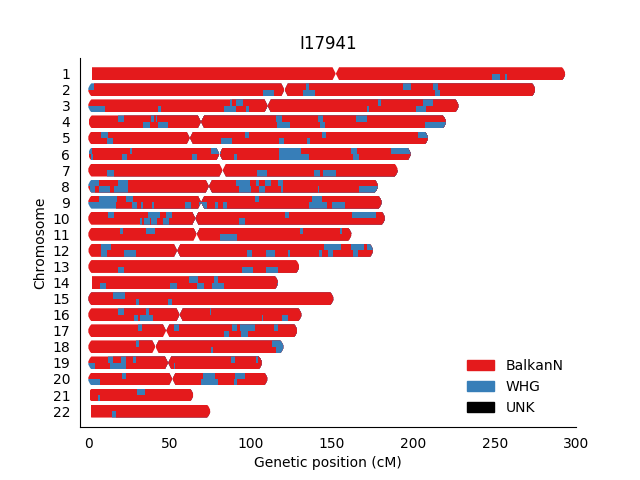

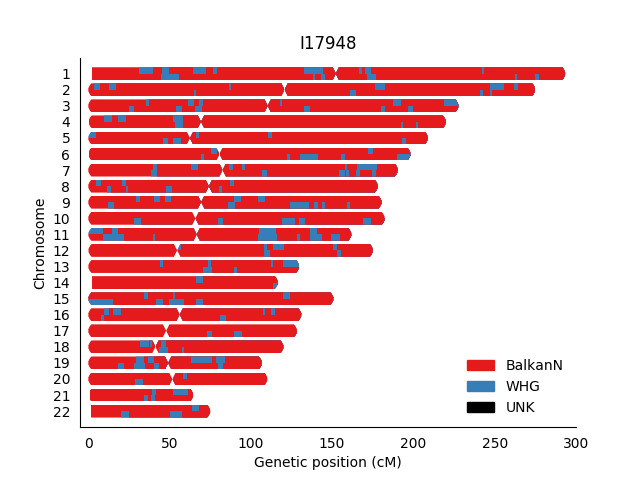

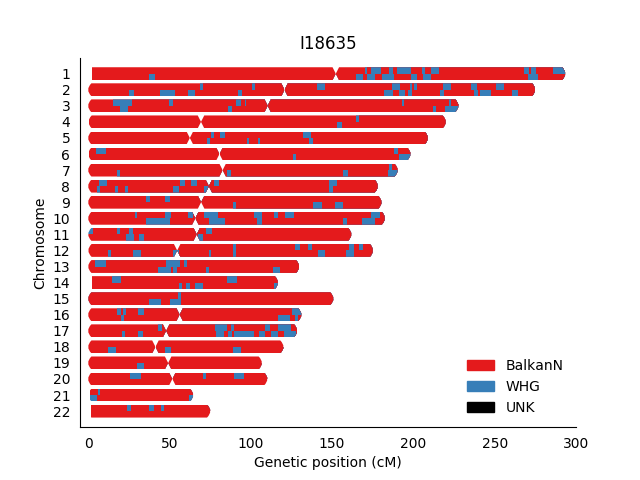

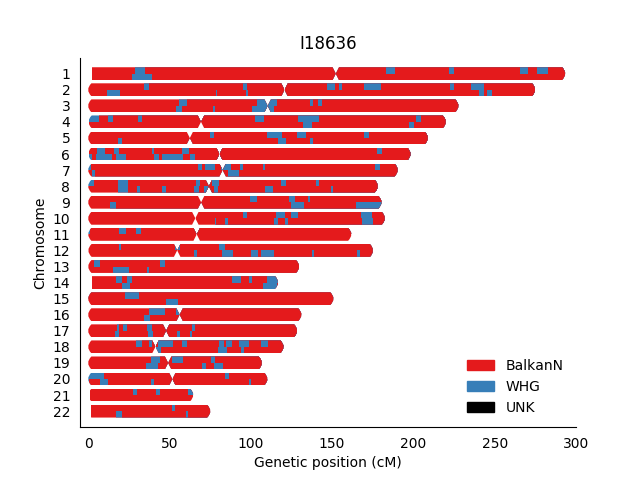

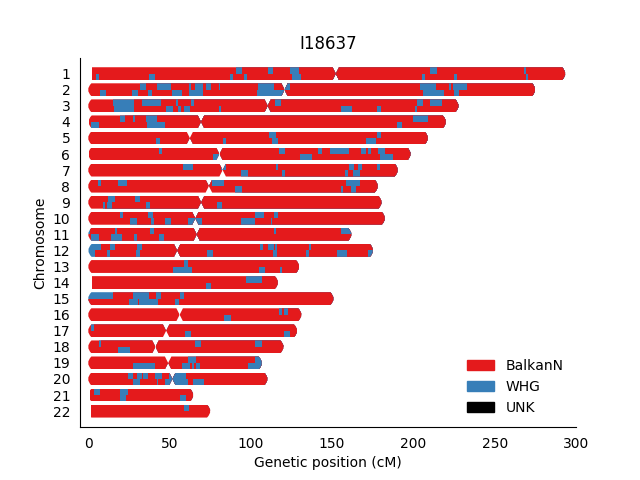

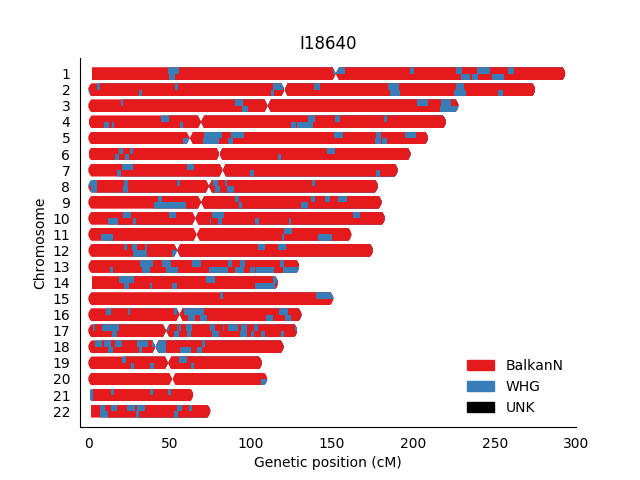

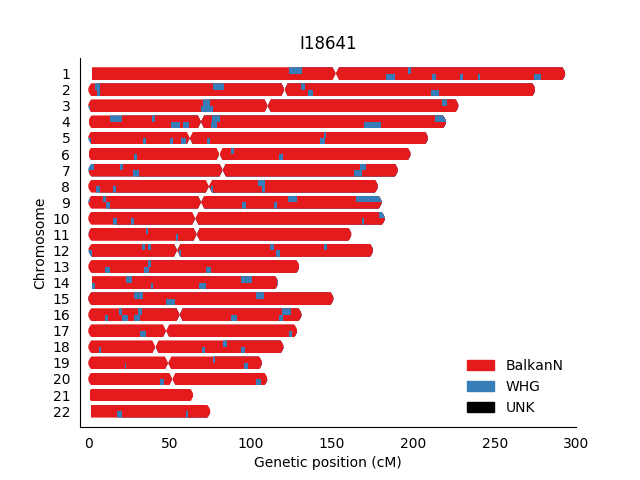

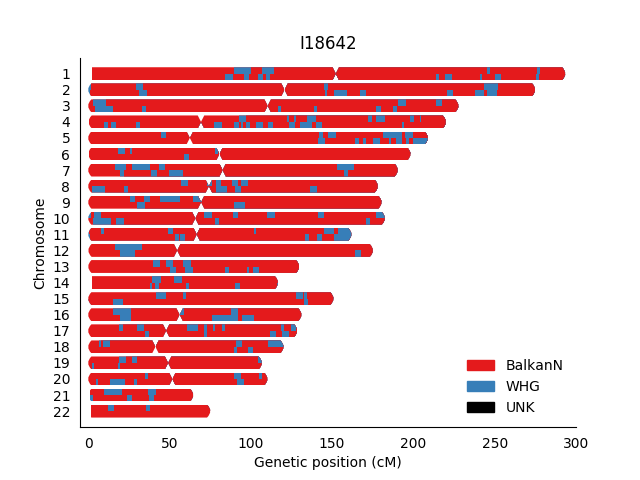

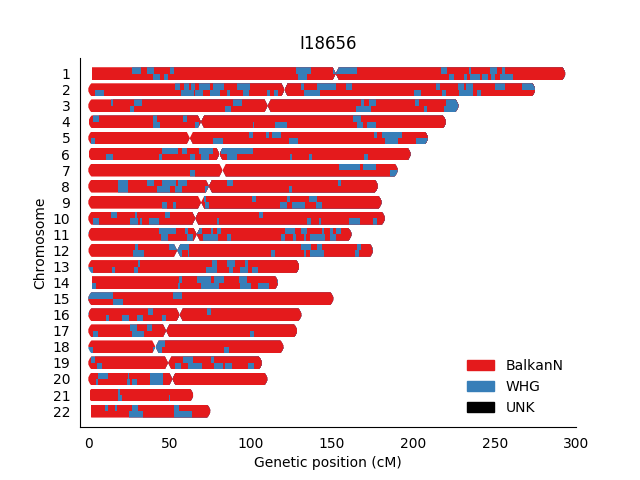

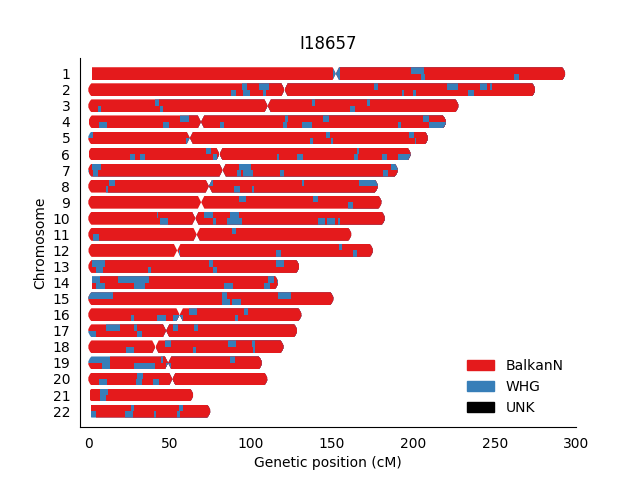

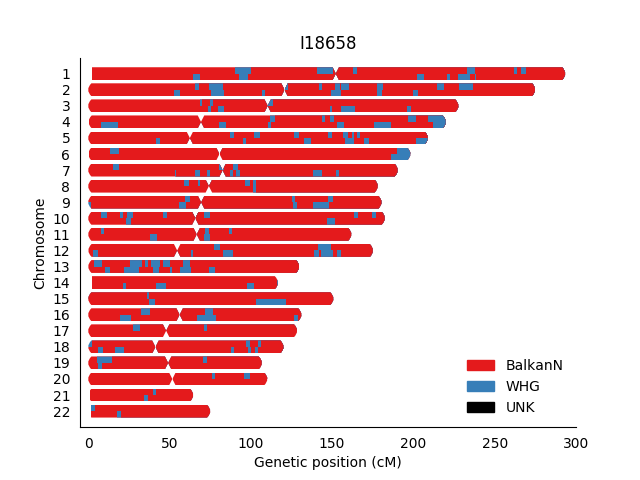

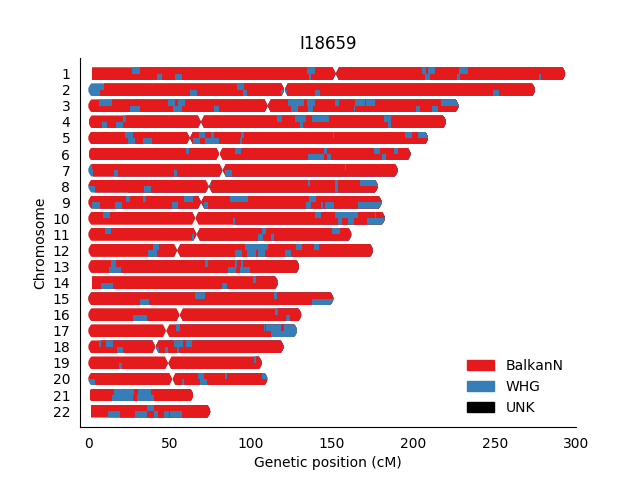

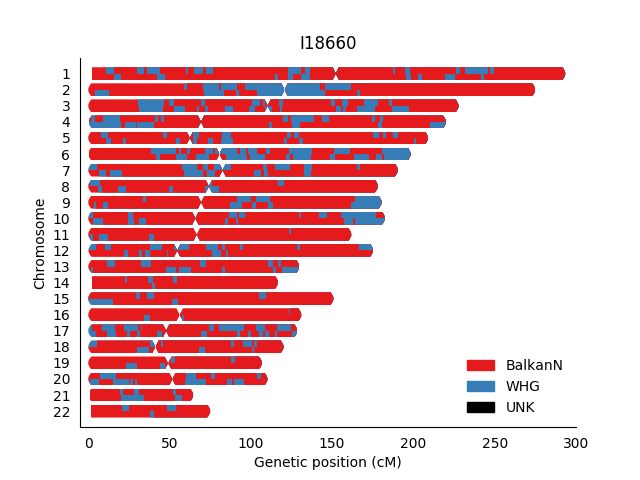

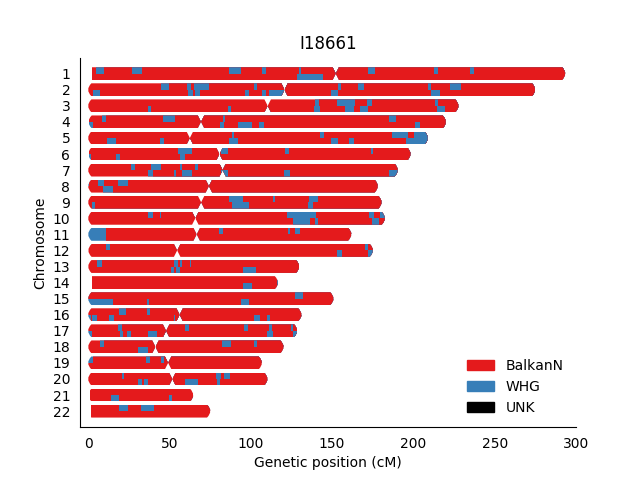

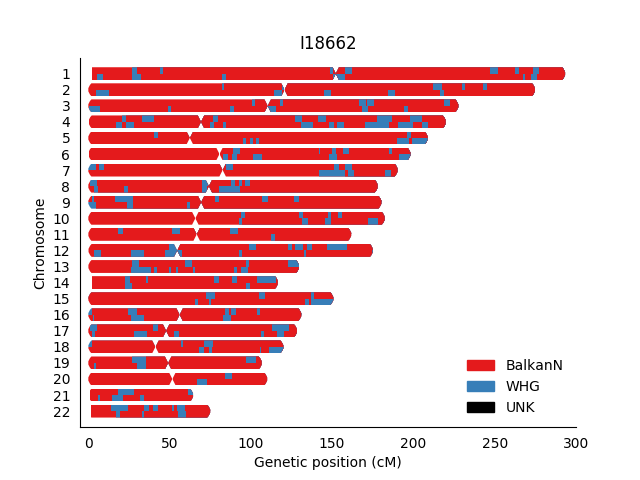

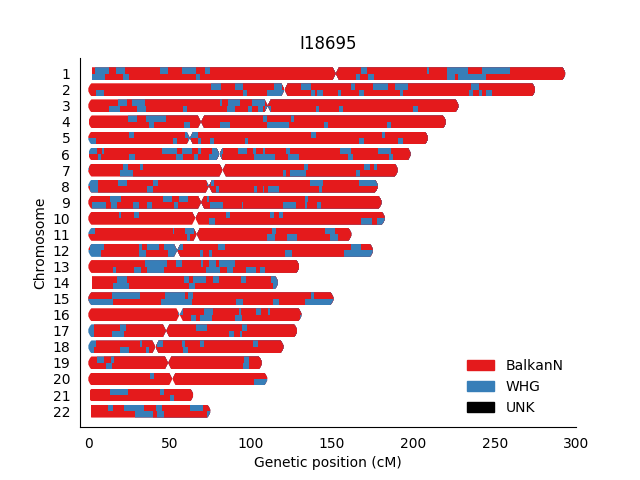

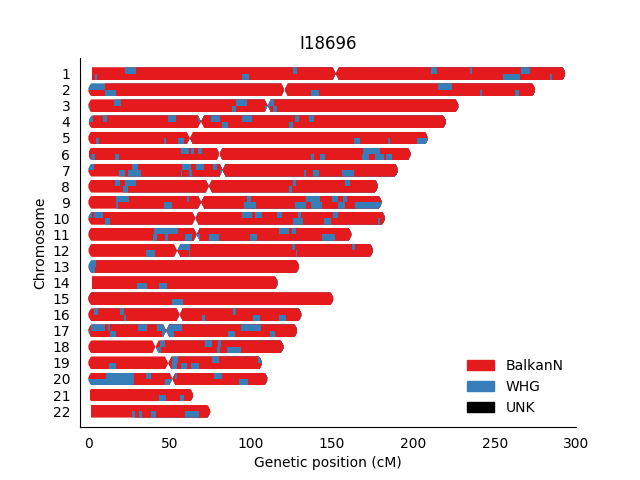

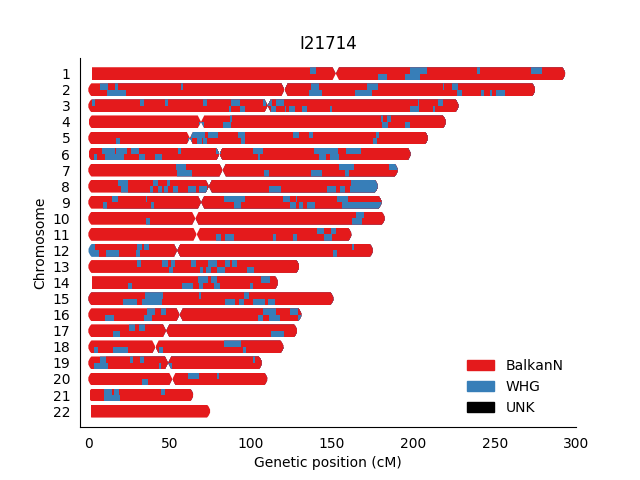

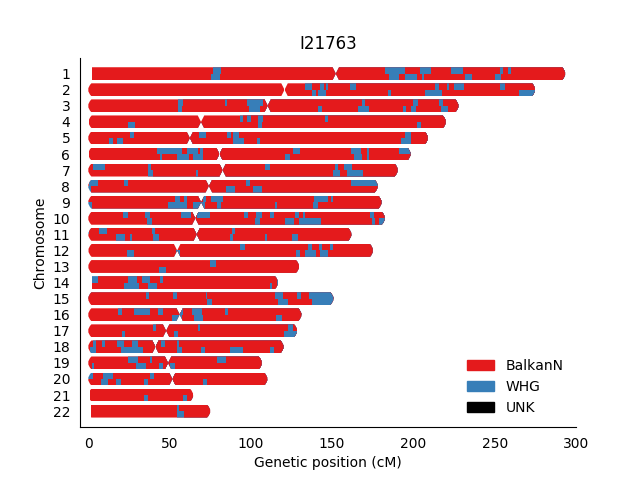

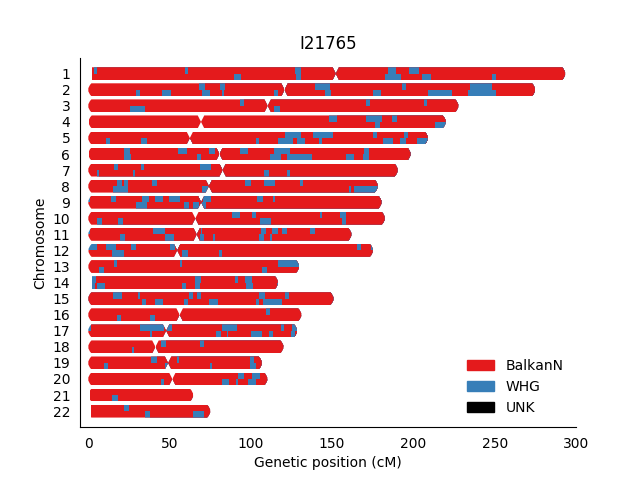

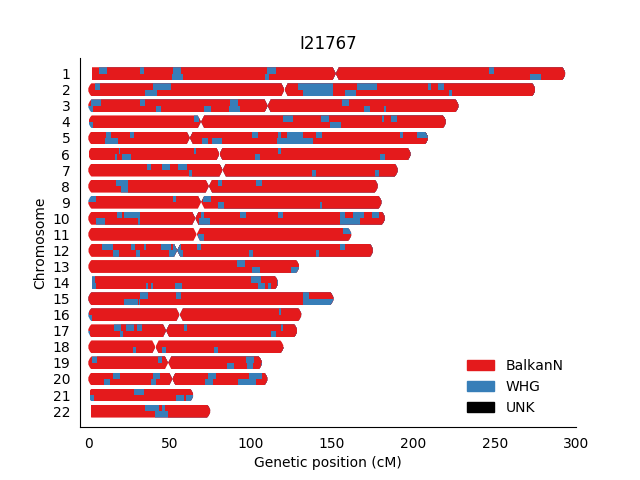

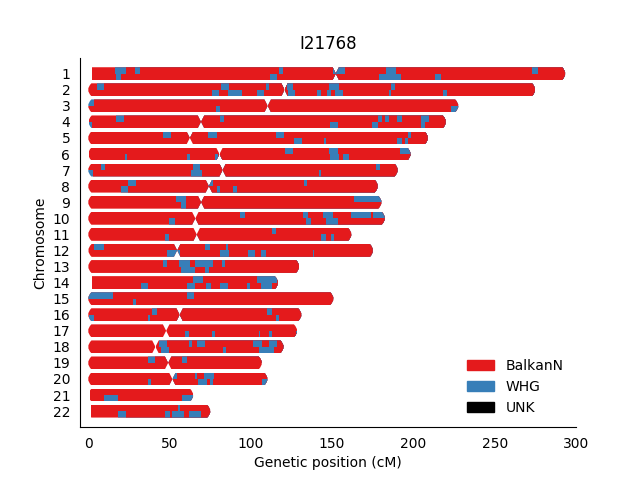

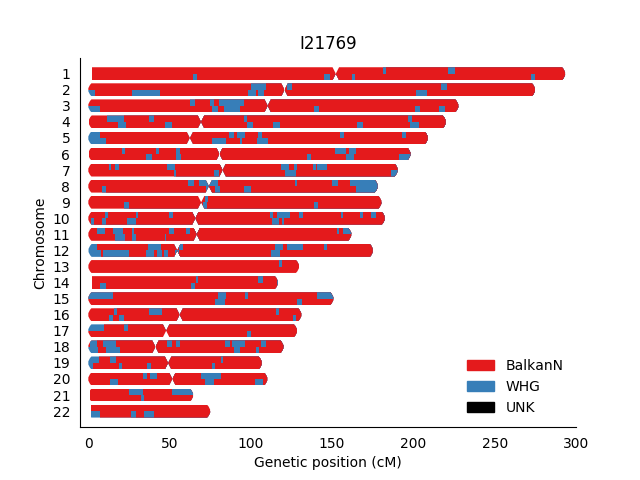

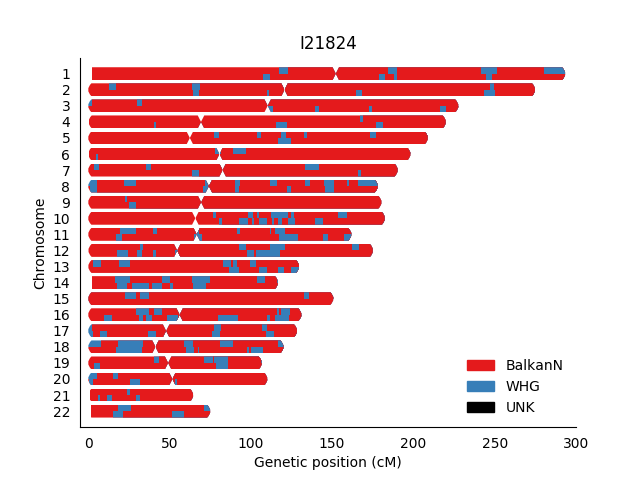

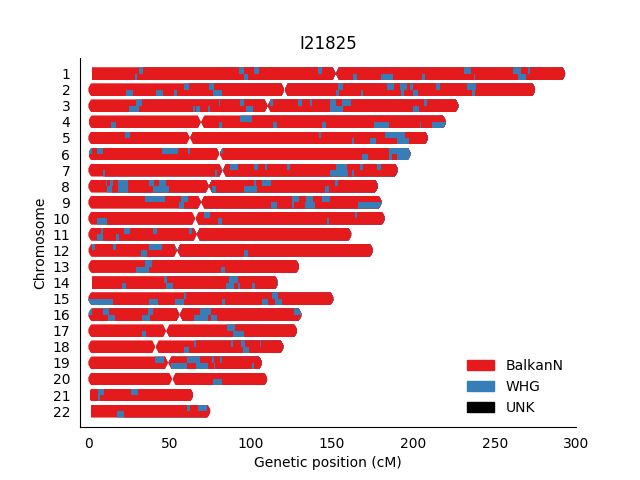

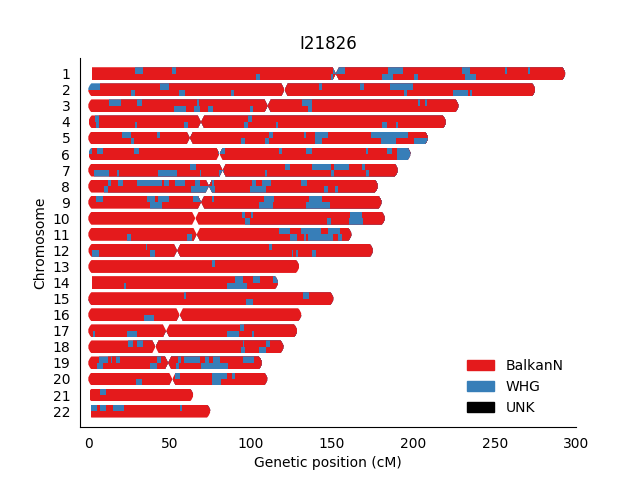

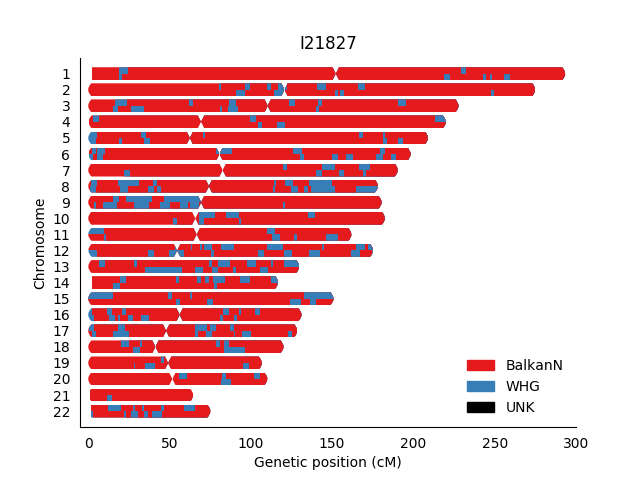

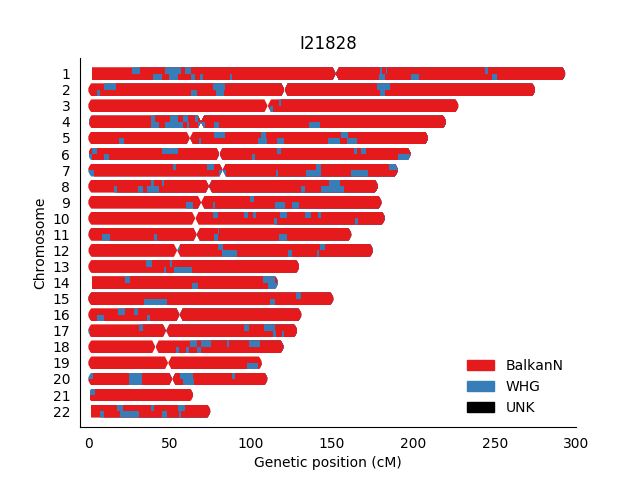

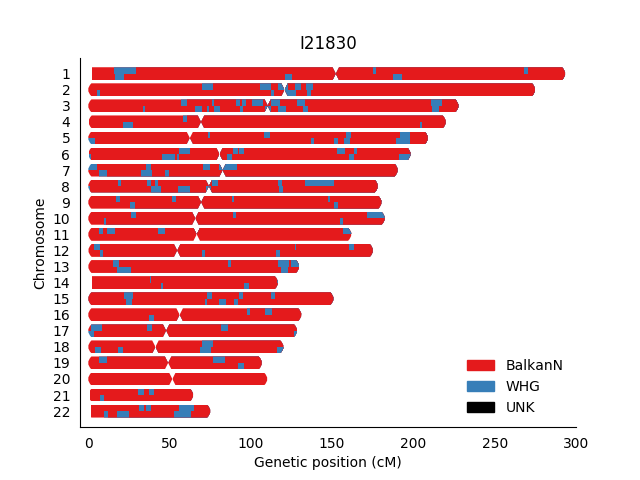

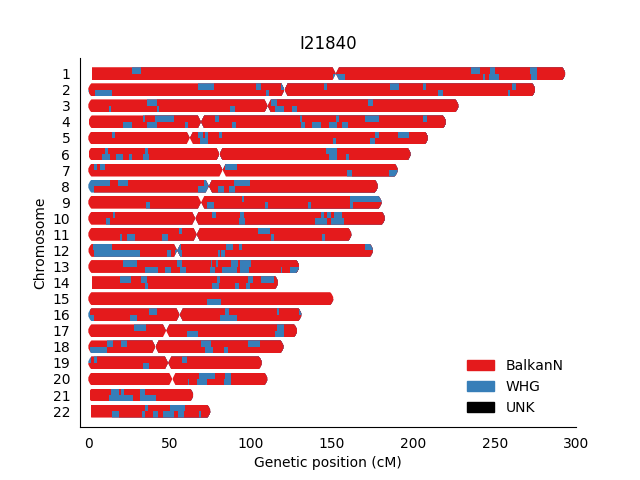

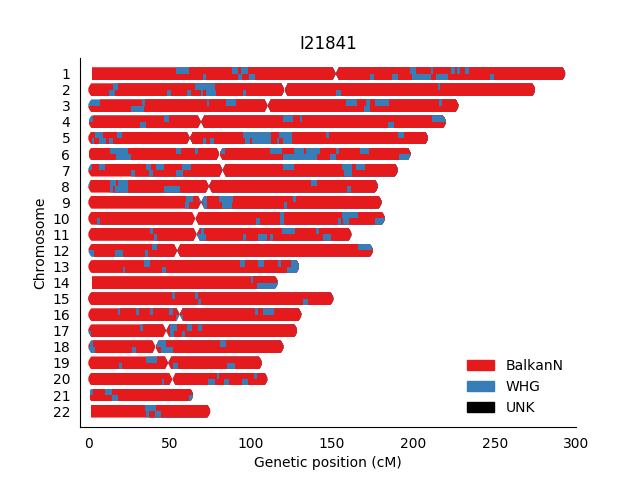

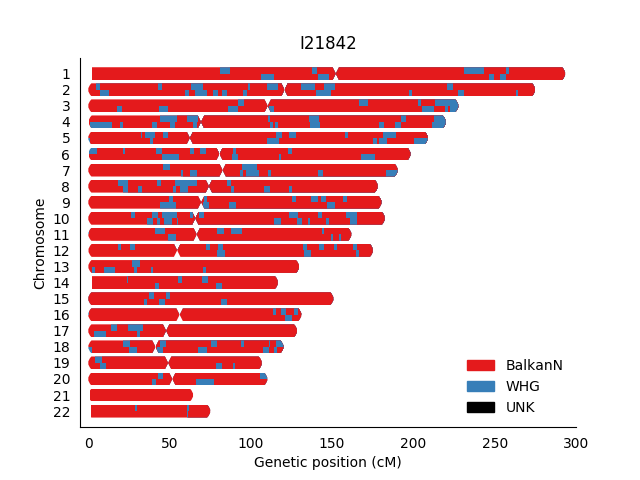

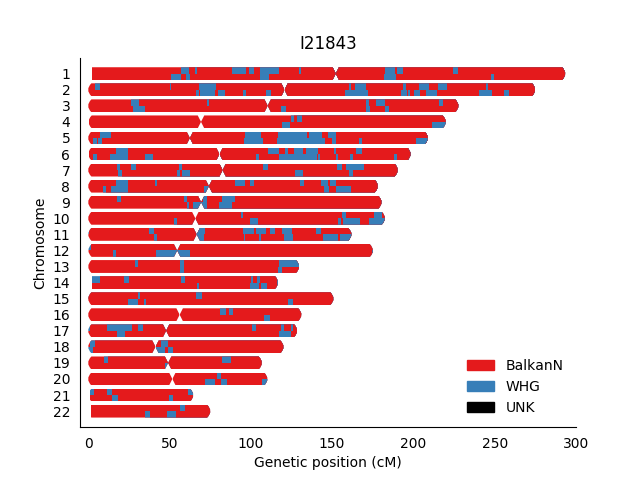

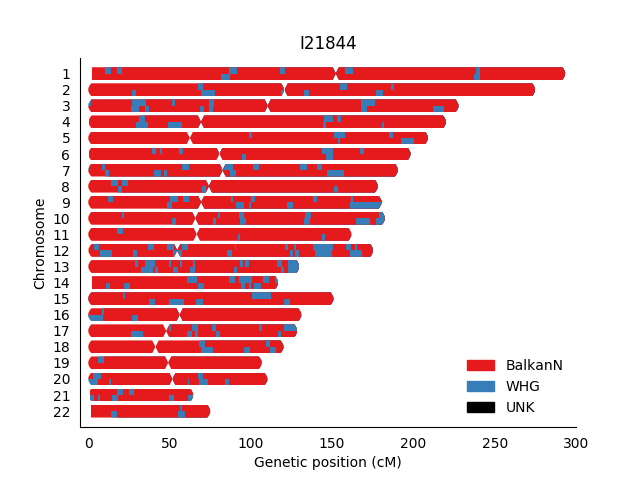

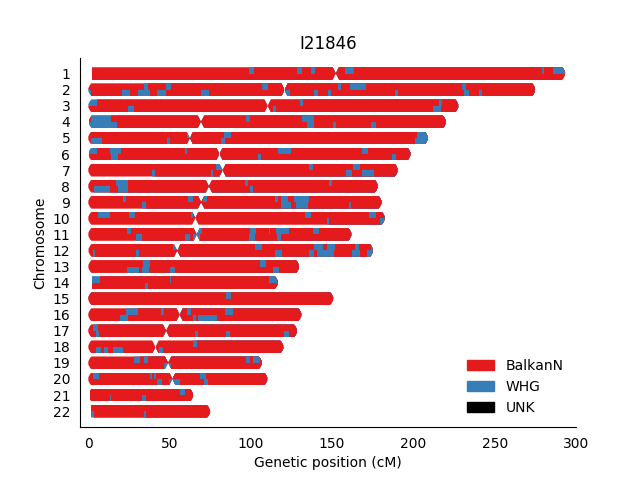

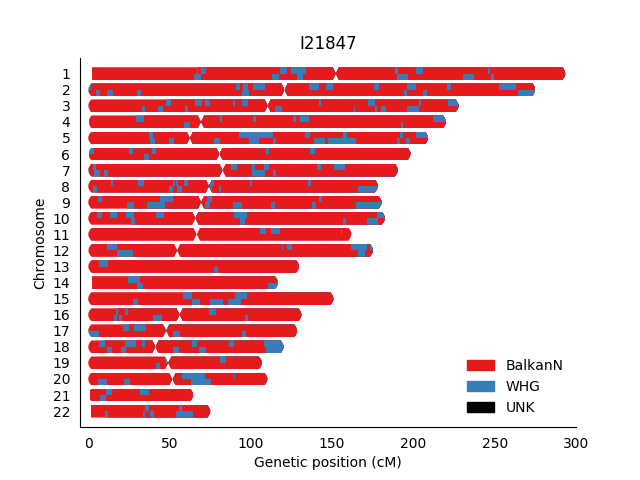

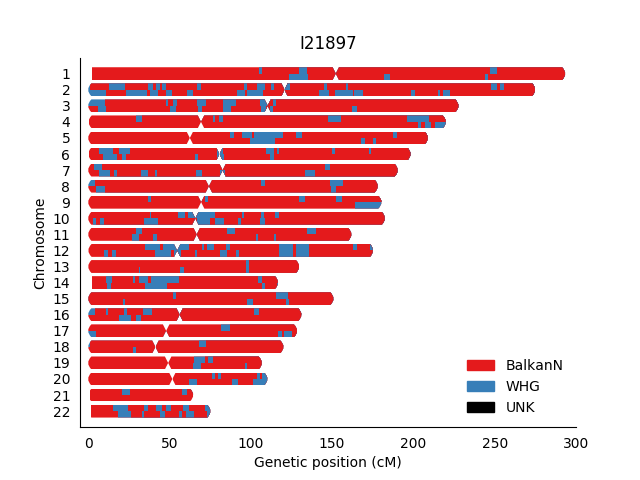

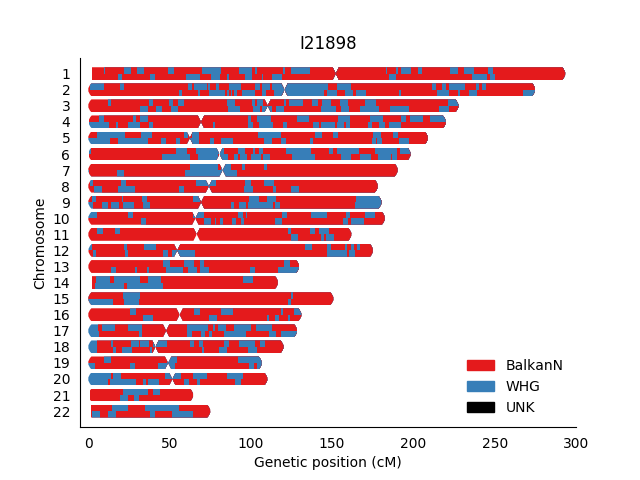

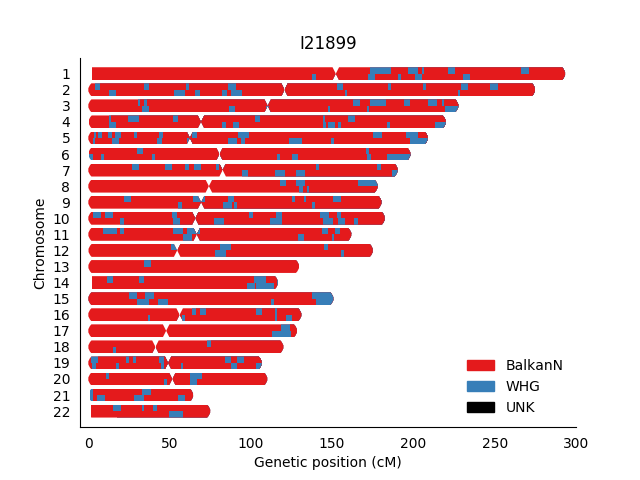

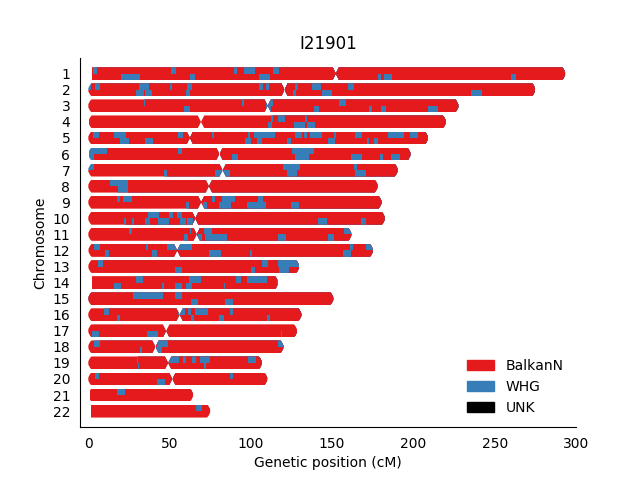

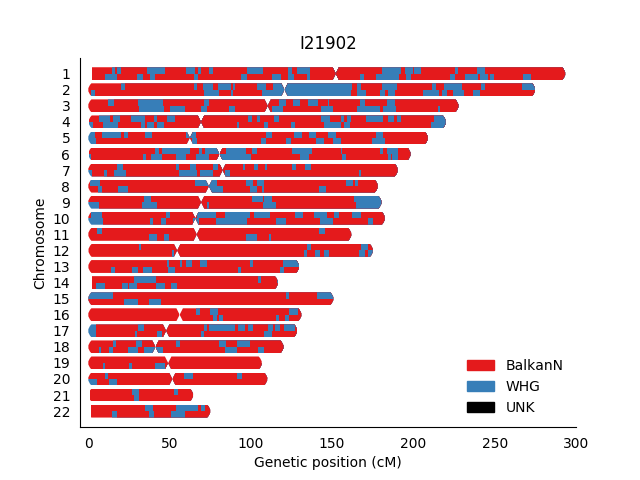

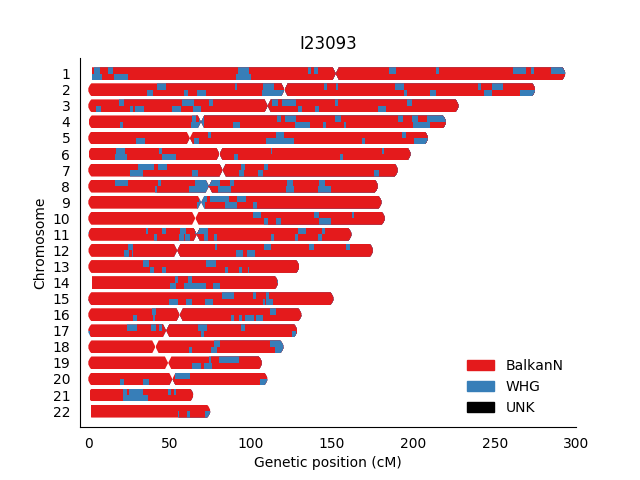

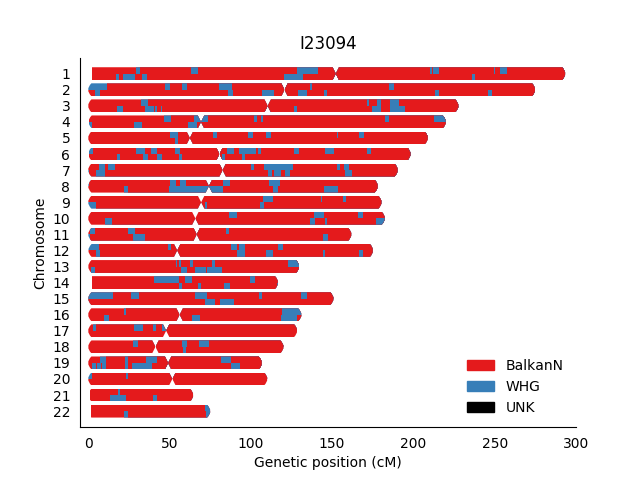

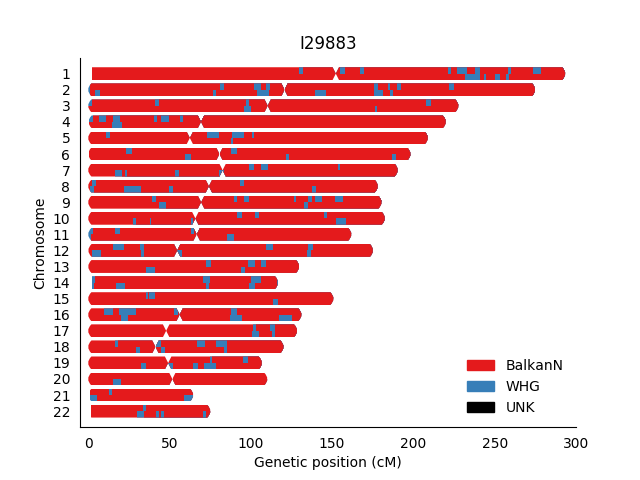

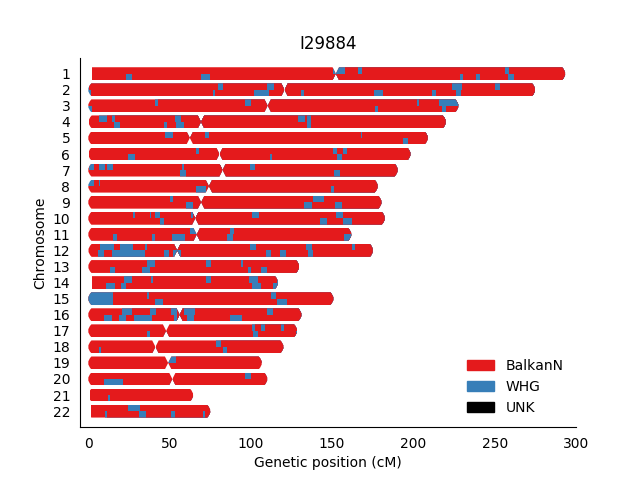

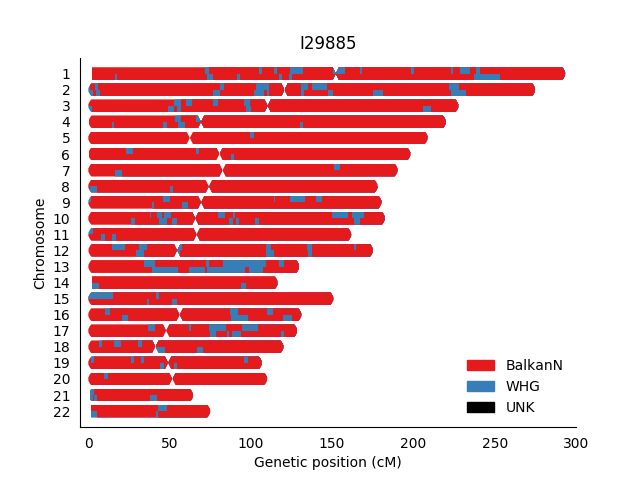


**Supplementary Figure 23:** Local ancestry maps obtained with RFMix on the 79 ALPC individuals with imputation scores > 0.8 imputed and phased with GLIMPSE and plotted with matplotlib 3.7.1 in Python 3.7.6. Some individuals show much longer inferred WHG segments than others, with I21902 having the longest segment and the largest summed WHG fractions in I10349 and I21898; these two individuals also are inferred to have the highest WHG ancestry with qpAdm (Supplementary Table 5).

**Supplementary Figure 24:** Regression of RFMix and qpAdm WHG ancestry estimates in ALPC individuals. We used a two-sided liniar regression test. The p-value of this test is 2.5e-30.

**Section 6: Kinship**

**Authors:** Iñigo Olalde, Pere Gelabert

The complete kinship results are displayed in Supplementary Table 7.

**Description of the Methodology**

We plotted the results in pedigrees. A circle denotes a female, and a square a male. Inside each circle, there are four lines: genetic ID, grave ID (in parenthesis), mtDNA haplogroup, and anthropological age. Inside each square, there are five lines: genetic ID, grave ID (in parenthesis), Y-chromosome haplogroup, mtDNA haplogroup, and anthropological age. A question mark inside a circle means that the individual is not sequenced. A line with dots denotes a relationship that cannot be drawn in the pedigree due to a lack of information.

**6.1 Nitra Horné Krškany**

**Nitra family A**

I16007 (female) and I14178 (male) are siblings according to the K1 value. I11873 is a 2nd- or 3rd-degree relative of them, showing a recombination pattern similar in both cases and very fragmented, which would be consistent with being a paternal aunt. I18143 and I11873 are 2nd- or 3rd-degree relatives, but the relationship's nature is unclear due to low coverage. I11873 is a young woman, and the two other individuals (I16007, I14178) are children who show indications of head trauma and are buried in the same grave. Both children show elevated ROH (33 and 28 cM in regions >4 cM) compared to the rest of the individuals from Nitra, compatible with inbreeding (Supplementary Figure 25).

**Supplementary Figure 25:** Pedigree of Nitra family A.

**Nitra family B**

I16246 is the mother of I16008 according to the K1 values.

**Nitra family C**

I25175 is a 3rd-degree relative of I18105. No further information can be added.

**Nitra family D**

I14599 is the mother of I16009 (female), according to K1 values. I17339 is a 3rd degree relative of I16009, probably on the paternal side. I14599 shows low strontium values, and I16009 and I17339 have average strontium values. I14599 is estimated to be a fourth-degree relative of I16010.

**Nitra family E**

I14180 is the father of I17538, consistent with the K1 value, and both are 4^th^-degree relatives of I14183 and I14600. I14600 and I18091 are 1^st^-degree relatives. Possibly I14600 is the son of I18091. I18144 and I18091 are sister and brother, and I16011 seems to be their nephew. Looking at the IBD plots, I14183 is differently related to I14600 and I14183, which is indicated by different recombination events. I18097 is a 3^rd^-degree relative of I18091 and a 4th-degree relative of I18144; this individual is not displayed in the pedigree as the relationship is unknown (Supplementary Figure 26).

**Supplementary Figure 26:** Pedigree of Nitra family E.

**Nitra family F**

I16013 and I16015 are both females and second-degree relatives. Probably they are half-sisters or aunt-niece.

**Nitra family G**

I16245 and I14177 are 2nd- or 3rd-degree relatives, both males. Both individuals have long ROH segments and different mtDNA haplogroups.

**Nitra family H**

I17345 and I16016 have a 3rd-degree relationship, and both are males. I17344 and I17345 are fourth-degree relatives.

**Nitra Family I**

I14179 and I25176 are 3rd-degree relatives.

**Nitra Family J**

I17545 and I16241 are daughter and mother

**6.2 Polgár-Ferenci-hát Family**

**Polgár-Ferenci-hát Family A**

Family A from Polgár-Ferenci-hát Family consists of 13 individuals. I17910 and I21897 are siblings based on the *r* value and low K1 value. I17910 and I17909 share long IBD and are inferred to be 2nd-degree relatives (I17909 was buried with 2 Spondylus beads). They may have been half-brothers, which is also consistent with the relationship between I17909 and I21897. I21824 and I11933 are 3rd-degree relatives of both I17910 and I21897, but not of I17909, which indicates that the relationship must be on the maternal side, which is also consistent with the mtDNA haplogroup. These patterns would be coincident with their being cousins. I11933 and I21824 are brother and sister according to the K1 and the r_X. I21769 is a 3rd-degree relative of I17910 and I21897 and a fourth of I11933. I11929 is a 3rd-degree relative of the father of I17909, I21897, and I17910, but it is unclear which relationship they share. I21843 is the father of I21765 according to the K1 value and the clear similar profile in the X chromosome. This individual is also the brother of I21841, according to the K1 values. Individual I21767 is the nephew of I21841 as he shares a 2nd-degree relationship with I21841 and I21843. I21901 is inferred to be a 3^rd^ or 4th-degree relative of I21843, I21841, and I21765. I21841 and I21843 are likely offspring of 1st-3 relatives, the same as I21897 and I21910 (Supplementary Figure 27).

**Supplementary Figure 27:** Reconstructed pedigree of Pólgar-Ferenci-hát family A.

The burials of this family are physically nearby at the site. Individuals I17909 and I17910 are buried together and in the same area as individual I21879. Individuals I21824 and I11933 are siblings and are also in the same area. The other nucleus of this family, composed of individuals I21841, I21843, I21901, I21765, and I21767, is located in a separate area. The first group (I17909, I17910, I21879, I21824, and I11933) are buried in the ALPC-I area, while I21841, I21843, I21901, I21765, I21767 are located in the ALPC II-IV areas, showing connections between individuals in different phases of Pólgar-Ferenci-hát.

**Pólgar-Ferenci-hát family B**

I21898 is the father of I21902 (male) and I18660 (female). I21898 is a 4th-degree relative of I18695, who is the daughter of I21827. I18695 and I21898 are also 4th-degree relatives of I17911, although the exact nature of that relationship is unclear (Supplementary Figure 28).

**Supplementary Figure 28:** Pedigree of Pólgar-Ferenci-hát family B.

Individuals I21898, I21902, and I18660 are buried together, and individuals I18695 and I21827 are buried together. They are not far from the other ones and also close to I17911. All these individuals are located in the ALPC I area. Individual I18660, which corresponds to PF718 burial (Supplementary Figure 29), is buried with a Spondylus clay bead and a cattle figurine, which is not common for the period of the burial. Individual I17911 has a lot of small Spondylus beads. In the burials which could imply membership of a privileged group [^47^](https://paperpile.com/c/2vFjDD/1ByVo).

**Supplementary Figure 29:** Individual I18660, which corresponds to PF718 burial.

**Polgár-Ferenci-hát family C**

I21840 is the mother of I18662 (male) and the grandmother of I21768 (male) and I21844 (male). I18662 is the grandfather of I18659 and the uncle of I21844. I18659 is compatible with being the grandson of I18662. Individuals I21847, I18662, I21768, and I18659 are all located very close to each other. Individual I21844 is located far from all. Far away from these, individuals I21825, I21826, and I18658 are all close together in the ALPC II-IV area. I18657 is compatible with being a 3rd-degree relative of I21847 (Supplementary Figure 30).

**Supplementary Figure 30:** Pedigree of Pólgar-Ferenci-hát family C.

**Polgár-Ferenci-hát family D**

I18656 and I21842 are 2nd-degree relatives and show no similarity at all on the X chromosome, implying that the relationship must be paternal-sided. They also share the same Y chromosome haplogroup and a different mtDNA one. These individuals are compatible with being: half-paternal brothers, uncles and nephews, or grand-father grand-son. I18696 (adult male) is a 3rd-degree relative of I18656 but with no evidence of relatedness to I21842, which could be evidence of the first two being half-brothers or grandfather and grandson. I18696 shows a possible 4th-degree relationship with I21826, who is a member of Family C. The graves of individuals I21842 and I18656 are closely located on the site.

**6.3 Asparn-Schletz**

**Asparn-Schletz Family A**

I30431 (FN 162, probably young adult) and I24892 (1993/4, FN 4464, FN 4518, probably 45-55 years are inferred to be son and father respectively, both from the ditch. They also have a 4th-degree relationship to I24017 (FN 9230, 2.5-3 years) from the settlement.

**Asparn-SchletzFamily B**

I24278 (1985/ind 57, FN 249, 1-2 years) and I27800 (FN 6950, 2-3 years) are both from the settlement area and genetically inferred to be brothers.

**Asparn-Schletz Family C**

I30411 (FN 10343, 2-4 years) and I24016 (FN 9366, 4-5 years) are both from the settlement (close to the ovens) and are genetically inferred to be brothers.

**Asparn-Schletz Family D**

I24906 (2001, FN 10806, 2-3 yrs) and I27787 (2001, FN 10640, 9 months), are from a double burial of males in the settlement and are siblings.

**Asparn-Schletz Family E**

I24272 (2005, FN 14143, 2-3 years) and I24884 (1996/2, FN 5076, 35-45 years) are 2nd-degree relatives and share part of the X chromosome; 1996/2 could be the maternal grandfather. Individual I24272 is from the settlement and I24884 is from the Massacre.

**Asparn-Schletz Family F**

I24021 (burial 16, 2000, 2-3 years) and I24904 (1999/3, FN 8328, 1.5-2.5 years) are both from the settlement and probably from the enclosing ditch system. They show the same Y Chromosome (C1a2) and different mtDNA and could be half-brothers.

**Asparn-Schletz Family G**

Individual I25347 (1991/1992, Fn. 3991 or 3491, age at death unclear) has a 3rd-degree relationship with I24269 (2001, S 33, FN11660, 3-4 years) (site), I24889 (1993/20, FN 4529, 2-3 years) (water well), and I24894 (1993/6, FN 4462, FN 4456, 3.5-4.5 years) (massacre).

**6.4 Rákóczifalva–Bagi-földek**

**Rákóczifalva Bivaly-Tó Family A**

Three individuals: I29884 (grave 53), I29883 (grave 53) and

I29885 (grave 53) are brothers. Individual I18640 (grave 53) is the paternal aunt of all these individuals, and I18641 (grave 561) is the maternal grandmother. I17934 (grave 53) is compatible with being a cousin of the three brothers (Supplementary Figure 31).

**Supplementary Figure 31:** Pedigree of Rákóczifalva Bivaly-Tó Family A.

**Rákóczifalva Bivaly-Tó Family B**

I17941 (Grave 272) and I17939 are second degree relatives.

I17933 (grave 340) is a 3rd-degree relative of I17941.

**Rákóczifalva Bivaly-Tó Family C**

I18637 (Grave 136) and I17940 (Grave 70) are brothers**.**

**Section 7: Population size trajectory inference**

**Authors**: Romain Fournier, Pier Francesco Palamara

**Methods**

HapNe is a method that can estimate demographic size changes in aDNA data over the few thousand years before an individual lived [^128^](https://paperpile.com/c/2vFjDD/6A2d). HapNe assumes that the individual is drawn independently from a single panmictic population. This hypothesis is likely to be inaccurate when individuals from an archaeological site are studied, as relatives are likely to be located in the same burial site. To mitigate this, we only retained one individual with the highest coverage within each identified family. However, the presence of undetected families or other structures in the remaining samples might introduce linkage disequilibrium (LD) that is not accounted for in the HapNe panmictic population model. To mitigate this potential issue, we performed a filtering step based on the genetic relationship matrix in each group. We ran HapNe's cross-chromosome LD test to compute an approximate p-value for the hypothesis that there is no underlying structure in the input samples after filtering using these two steps. This hypothesis was not rejected for the Schletz massacre samples (approximate p-value = 6.7%) and Nitra Horné Krškany (approximate p-value = 40.6%). However, the hypothesis was rejected for the individuals of Polgár-Ferenci-hát (approximate p-value < 0.0001), and thus we removed this site from our analyses of inference of population size changes over time.

We ran HapNe using default parameters and in "fixed prior" mode, which is recommended when comparing groups of different sample sizes or coverage. We ran HapNe without correcting for time heterogeneity for the Schletz samples, as they originate from the same time point. We assumed a uniform distribution between 5300-5000 BCE for the samples from Nitra Horné Krškany. Finally, generations were converted to years using a factor of 29.1 years per generation [^128^](https://paperpile.com/c/2vFjDD/6A2d). After selecting the individual with the highest coverage for each identified family, we used plink 2.0 [^129^](https://paperpile.com/c/2vFjDD/XPsGD) to compute the genetic relationship matrix (GRM). We then projected the individuals onto the first two principal components of the matrix, shown in Supplementary Figure 32. We removed all visual outliers (in red in Supplementary Figure 32) before testing for the presence of population structure.

**Supplementary Figure 32: Projection of unrelated individuals of different groups onto the first two principal components of their GRM**. (a) Schletz massacre. (b) Nitra Horné Krškany. (c) Polgár-Ferenci-hát. (d) Schletz (s=56) and Nitra Horné Krškany (s=18). Individuals filtered out in (a-c) are circled

**Section 8: Selection scans in the diploid data**

**Authors:** Xin Huang, Martin Kuhlwilm

**Filtering data**

After obtaining the imputed dataset, individuals with imputation quality score less than 0.8 were removed. We then extracted biallelic single nucleotide polymorphisms (SNPs) with moderate quality or high-quality imputation quality. Only SNPs with ancestral alleles in the 1000 Genomes Project [^130^](https://paperpile.com/c/2vFjDD/TWY38) were used for further analysis. The ancestral allele of each SNP was required to match either the reference allele or the alternative allele. The final SNP dataset was annotated with three databases—RefSeq, dbNFSP version 4.2c, and dbSNP version 150 with left-normalization—using the human reference genome hg19 coordinates by ANNOVAR version 2022Oct05 [^131–134^](https://paperpile.com/c/2vFjDD/ikEZE+2Mmik+Sqkhh+hYxTz). In total, 79 diploid imputed genomes from the ALPC population and 149 diploid imputed genomes from the LBK population were used for the studies of natural selection.

**Testing for evidence of selective sweeps in the thousands of years before the early farmers we analyzed lived**

To detect signatures of positive selection in the data, the iHS and nSL scores were estimated with selscan 2.0 [^135^](https://paperpile.com/c/2vFjDD/A4YQ). Only SNPs with a minor allele frequency (MAF) larger than 0.05 were used as cores when calculating these scores. Other parameters were set to the default values in selscan 2.0. Unnormalised iHS and nSL scores were normalised using the norm program from selscan 2.0 with the default settings. To detect signals of long-term balancing selection in the data, the B1 scores were estimated with unfolded allele frequencies by BetaScan [^136^](https://paperpile.com/c/2vFjDD/abFIC). Only SNPs with MAFs larger than 0.05 were used during the whole genome scan. SNPs were further removed if they were in the regions defined by the RepeatMasker table, simple repeats table, and segmental duplication table from the UCSC Table Browser with hg19 coordinates (last accessed December 2022). SNPs were also removed if they had p-values less than 10^-3^ from one-tailed exact tests for Hardy-Weinberg equilibrium in each population by PLINK 1.09 [^137,138^](https://paperpile.com/c/2vFjDD/WS09N+dGc7y). Following Siewert and Voight (2017), only SNPs with a minor allele frequency larger than 0.15 were used as cores when calculating the B1 scores. Other parameters were set to the default values in BetaScan.

**Results and Discussion**

We tested for signatures of positive selection with both the iHS and nSL scores (Extended Figure 6), because the iHS score may only perform well in detecting hard selective sweeps, while the nSL score is expected to be sensitive to soft as well as hard selective sweeps [^139^](https://paperpile.com/c/2vFjDD/BA1IC). We chose those SNPs with either absolute normalized iHS or nSL scores in the top 0.05% as candidates (Supplementary Table 12). Based on both the iHS and nSL scores (including phased and unphased versions), one gene harbours signatures of positive selection in both the ALPC and LBK populations (Supplementary Table 11). Several candidate genes are plausibly associated with human pigmentation. For example, the *PRKCH* gene encodes the PKCη protein in melanocytes and may participate in the protein kinase C-dependent pathway to regulate melanogenesis [^140^](https://paperpile.com/c/2vFjDD/PYYJT). The *PTPRN2* gene had a higher level of expression in lightly pigmented melanocytes than darkly pigmented melanocytes, a phenotype that is similar to that of human pigmentation gene *SLC45A2* [^141^](https://paperpile.com/c/2vFjDD/rTeGb). The expression levels of *CDH12* and *MACROD2* were significantly changed in hyperpigmented skin using meta-analysis, indicating they may affect human pigmentation [^142^](https://paperpile.com/c/2vFjDD/cXGv4).

We also tested for evidence of long-term balancing selection with the B1 score from BetaScan [^136^](https://paperpile.com/c/2vFjDD/abFIC). We chose those SNPs with B1 scores in the top 0.05% as candidates (Supplementary Table 12). Similar to previous studies using different approaches to detect long-term balancing selection with modern human populations [^143,144^](https://paperpile.com/c/2vFjDD/cFAMA+PQhPO) , one of the strongest signals is from the HLA region on human chromosome 6 (Figure 6, Extended Figure 6). Twenty-six genes harbor signatures of long-term balancing selection in both the ALPC and LBK populations (Supplementary Table 11). Many of them were reported as significant outliers in a previous study using genomes from modern European populations: GBR and TSI [^144^](https://paperpile.com/c/2vFjDD/PQhPO), indicating long-term balancing selection may persist at these genes.

We explored the possibility that some of the signals of selection would be associated with differential proportions of WHG ancestry and thus we computed the average WHG ancestry in the sections of the genome where the SNPs were located (Supplementary Table 12) and plotted the average selection scores from candidate SNPs within each region against the WHG ancestry (Extended Figure 7). The correlation coefficients provide suggestive but not compelling evidence that non-WHG ancestry contributed more to the top iHS/nSL scores (the best P-value of 0.031 is not significant after Bonferroni correction for eight hypotheses tested).

**Section 9: Supplementary References**

1. [Jovanović, J., Blagojević, T., Živanović, S., Putica, A. & Stefanović, S. *Контекстуална и антрополошка анализа људских скелетних остатака са локалитета Тополе-Бач*. (2017). doi:](http://paperpile.com/b/2vFjDD/ICR0G)[10.5281/zenodo.1218369](http://dx.doi.org/10.5281/zenodo.1218369)[.](http://paperpile.com/b/2vFjDD/ICR0G)

2. [Trajković, Č. Topole–Bač. in *The Neolithic of Serbia: Archaeological Research 1948–1988* 99–101 (University of Belgrade, Faculty of Philosophy, Centre for Archaeological Research, Belgrade, 1988).](http://paperpile.com/b/2vFjDD/c0fUs)

3. [Trajković, D. Šećerana, Topole, Bač – Praistorijsko naselje i grobovi. *Arheološki pregled 1* 23–24 (1977).](http://paperpile.com/b/2vFjDD/tmhsi)

4. [Whittle, A., Bartosiewicz, L., Boric, D., Pettitt, P. & Richards, M. In the beginning: New radiocarbon dates for the early Neolithic in northern Serbia and south-east Hungary. *Antaeus (Budapest)* 63–117 (2002).](http://paperpile.com/b/2vFjDD/2o0BP)

5. [Jovanović, J. *et al.* New radiocarbon dates, stable isotope, and anthropological analysis of prehistoric human bones from the Balkans and southwestern Carpathian Basin. *Doc. Praehist.* **48**, 224–251 (2021).](http://paperpile.com/b/2vFjDD/Qa8ym)

6. [Karmanski, S. *Donja Branjevina*. (Arheološka zbirka pri Narodnom univerzitetu, 1979).](http://paperpile.com/b/2vFjDD/FUx29)

7. [Karmanski, S. *Donja Branjevina: A Neolithic Settlement Near Deronje in the Vojvodina, Serbia*. (Trieste, 2005).](http://paperpile.com/b/2vFjDD/vIGz1)

8. [Vuković, J. B. Рециклирање предмета од керамике: неолитске фигурине као алатке, пример с локалитета Павловац- Чукар. *Zbornik Narodnog muzeja u Beogradu* **25**, 39–49 (2021).](http://paperpile.com/b/2vFjDD/xt5YP)

9. [Biagi, P. Karmanski S. 2005 - Donja Branjevina: A Neolithic Settlement near Deronje in the Vojvodina (Serbia). *Società per la Preistoria e Protostoria della Regione Friuli-Venezia Giulia, Quaderno 10* (2005).](http://paperpile.com/b/2vFjDD/Ux0zn)

10. Grbić, M. Bemalte Keramik aus Starčevo im Banater Danaugelände – Jugoslavien. In J. Kostrzewskiego Księga pamiątkowa ku uczczeniu siedemdziesiątej rocznicy urodzin Prof. Dr. Włodzimierza Demetrykiewicza. Poznan: Adam Mickiewicz University, 111–112 (1930).

11. Aranđelović-Garašanin, D. Starčevačka kultura. Ljubljana: Univerza v Ljubljani (1954).[.](http://paperpile.com/b/2vFjDD/iFdNO)

12. [Fewkes, V. J., Goldman, H. & Ehrich, R. W. Excavations at Starčevo, Yugoslavia, Seasons 1931 and 1932: а Preliminary Report. *Bulletin of American School of Prehistoric Research* **9**, 33–54 (1933).](http://paperpile.com/b/2vFjDD/RN571)

13. Reingruber, A. Timelines in the Neolithic of Southwestern Anatolia, the Circum-Aegean, the Balkans and the Middle Danube Area. In N.N. Tasić et al. Making Spaces into Places, BAR S3001. Oxford: BAR Publishing, 17–32 (2020)[.](http://paperpile.com/b/2vFjDD/eKOGO)

14. Reingruber, A. & Thissen, L. The 14SEA Project. A 14C database for Southeast Europe and Anatolia (10,000–3000 calBC)[.](http://paperpile.com/b/2vFjDD/uLoPl)

15. B[á](http://paperpile.com/b/2vFjDD/CAbFW)r[á](http://paperpile.com/b/2vFjDD/CAbFW)ny A[, B. Öskori Csonteszközök Vörs. *Communicationes Archaeologicae Hungariae.* (2002).](http://paperpile.com/b/2vFjDD/CAbFW)

16. [Miloglav, I. Ervenica - dio naselja Vučedolske kulture. *Opuscula archaeologica* **31**, 27–48 (2007).](http://paperpile.com/b/2vFjDD/seRAI)

17. [Durman, A. & Marić, R. *Vučedolski Orion I Najstariji Europski Kalendar*. (Nacionalna i sveučilišna knjižnica, 2000).](http://paperpile.com/b/2vFjDD/2hvH2)

18. [Dimitrijević, S. Arheološka iskopavanja na području vinkovačkog muzeja, rezultati 1957.–1965. *Acta Musei Cibalensis 1* (1966).](http://paperpile.com/b/2vFjDD/6d4M9)

19. [Minichreiter, K. *Starčevačka Kultura U Sjevernoj Hrvatskoj*. (Sveučilište u Zagrebu, Filozofski fakultet, Arheološki zavod, 1992).](http://paperpile.com/b/2vFjDD/1e7Ap)

20. [Brunšmid, J. Colonia Aurelia Cibalae. *Vjesnik Arheološkog muzeja u Zagrebu* 116–166 (1902).](http://paperpile.com/b/2vFjDD/faaYc)

21. [Iskra Janošić. Cibalae–Vinkovci, zaštitni radovi. *Arheološki pregled* (1977).](http://paperpile.com/b/2vFjDD/eSM5g)

22. [Iskra-Janošić, I. Arheološka istraživanja na području općine Vinkovci. *Arheološka istraživanja u istočnoj Slavoniji i Baranji* (1984).](http://paperpile.com/b/2vFjDD/S9Cfa)

23. [Iskra-Janošić, I. Povijesni pregled arheoloških istraživanja u Vinkovcima. *Godišnjak Ogranka Matice hrvatske Vinkovci* (1992).](http://paperpile.com/b/2vFjDD/usoJp)

24. [Macphail, R. I., Haită, C., Bailey, D. W., Andreescu, R. & Mirea, P. The soil micromorphology of enigmatic Early Neolithic pit-features at Măgura, southern Romania. *Studii de preistorie* **5**, 61–77 (2008).](http://paperpile.com/b/2vFjDD/8PM5L)

25. [Andreescu, R. & Mirea, P. Teleorman Valley: The beginning of the Neolithic in Southern Romania. *Acta Terrae Septemcastrensis* **7**, 57–75 (2008).](http://paperpile.com/b/2vFjDD/LRIST)

26. [Lazar, C. *Ituri Si Ritualuri Funerare in Neoliticul Si Eneoliticul Din Romania*. (Editura Cetatea de Scaun, 2020).](http://paperpile.com/b/2vFjDD/lVqIM)

27. [Vasic, R. Compte-rendu des fouilles du site préhistorique aVelesnica 1981--1982. *Djerdapske sveske. Cah Portes Fer* **3**, 271–285 (1986).](http://paperpile.com/b/2vFjDD/7UIXt)

28. [Mikić, G. Fauna mezolitskog sloja Velesnice. *Unpublished undergraduate thesis. The University of Belgrade* (1999).](http://paperpile.com/b/2vFjDD/4As6H)

29. [Vasić, R., Bonsall, C., Boroneanţ, V. & Radovanović, I. Velesnica and the Lepenski Vir culture. *in Prehistory. Oxford: Archaeopress …* (2008).](http://paperpile.com/b/2vFjDD/raVpa)

30. [Bonsall, C. *et al.* New AMS 14C Dates for Human Remains from Stone Age Sites in the Iron Gates Reach of the Danube, Southeast Europe. *Radiocarbon* **57**, 33–46 (2015).](http://paperpile.com/b/2vFjDD/qVbt3)

31. [Roksandic, M., Bonsall, C., Boroneanţ, V. & Radovanović, I. The human osteological material from Velesnica. *The Iron Gates in Prehistory. Oxford: Archaeopress. p* 243–249 (2008).](http://paperpile.com/b/2vFjDD/KQMPD)

32. [Boric, D. *Deathways at Lepenski Vir: Patterns in Mortuary Practice*. 565 (Serbian Archaeological Society, Belgrade, 2016).](http://paperpile.com/b/2vFjDD/WPs2D)

33. [Živanović, S. *Restes Des Ossements Humains à Velesnica*. (1991).](http://paperpile.com/b/2vFjDD/kq6eP)

34. [Ligner, J. Egerág határa (Egerág-Gyilkos-tó, HT-156 lh., KÖH74667). Régészeti kutatások. *Magyarországon 2010. Archaeological lnvestigations in Hungary 2010.* (2012).](http://paperpile.com/b/2vFjDD/nXbvH)

35. [Anders, A. & Siklósi, Z. *The First Neolithic Sites in Central/South-East European Transect. Volume III: The Körös Culture in Eastern Hungary*. (BAR Publishing, 2012).](http://paperpile.com/b/2vFjDD/NYKUP)

36. [Tóth, K. Arnót-Arnóti-oldal Dél in: Csengeri, P, A Herman Ottó Múzeum régészeti kutatásai 2014-ben. *A Herman Ottó Múzeum Évkönyve* **143**, 55 (2016).](http://paperpile.com/b/2vFjDD/tQuB2)

37. [Krisztán, T. Arnót-Nagy-bugyik in: Csengeri, P. – Kertész G. N., A Herman Ottó Múzeum régészeti kutatásai 2015-2016-ban. *A Herman Ottó Múzeum Évkönyve* **149**, 58–59 (2020).](http://paperpile.com/b/2vFjDD/d8R8d)

38. [Gamba, C. *et al.* Genome flux and stasis in a five millennium transect of European prehistory. *Nat. Commun.* **5**, 5257 (2014).](http://paperpile.com/b/2vFjDD/BE1Yv)

39. [Lipson, M. *et al.* Parallel palaeogenomic transects reveal complex genetic history of early European farmers. *Nature* **551**, 368–372 (2017).](http://paperpile.com/b/2vFjDD/Yzi14)

40. [Gyulai, F. Archaeobotanical research at the Körös Culture site of Ibrány--Nagyerdő and its relationship to plant remains from contemporaneous sites in Hungary. *Kozlowski, JK & Raczky, P. : Neolithization of the Carpathian Basin: Northernmost Distribution of the Starčevo/Körös Culture. Polish Academy of Arts & Sciences Krakow & Institute of Archaeological Sciences of The Eötvös Loránd University Budapest* 219–237 (2010).](http://paperpile.com/b/2vFjDD/XRWKE)

41. [Domboróczki, L., KaczanowsKa, M. & Kozłowski, J. The Neolithic settlement at Tiszaszőlős-Domaháza-puszta and the question of the northern spread of the Körös Culture. *Atti Soc. Preist. Protost. Friuli-VG* **17**, 101–155 (2010).](http://paperpile.com/b/2vFjDD/uIekL)

42. [Domboróczki, L., KaczanowsKa, M. & Kozłowski, J. The Neolithic settlement at Tiszaszőlős-Domaháza-puszta and the question of the northern spread of the Körös Culture. *Atti Soc. Preist. Protost. Friuli-VG* **17**, 101–155 (2010).](http://paperpile.com/b/2vFjDD/eiTJ1)

43. [Domboróczki, L., Kalli, A., Makoldi, M. & Tutkovics, E. K. The füzesabony-gubakút settlement development model of the alföld linear pottery culture in the light of the recent archaeological discoveries at hejőpapi-szeméttelep (2008-2011) and bükkábrány-bánya vii-vasúti dűlő (2009-2011). *Journal of Historical Archaeology & Anthropological Sciences* **2**, 31–43 (2017).](http://paperpile.com/b/2vFjDD/g2IKR)

44. [Bickle, P. & Whittle, A. *The First Farmers of Central Europe: Diversity in LBK Lifeways*. (Oxbow Books, 2013).](http://paperpile.com/b/2vFjDD/YuzSY)

45. [Domboróczki, L. I. Settlement structures of the Alföld Linear Pottery Culture (ALPC) in Heves County (North-Eastern Hungary): development models and historical reconstructions on micro, mezo and macro levels, 75-127 (2009).](http://paperpile.com/b/2vFjDD/kyKzi)

46. [Domboróczki, L. The Füzesabony-Gubakút Settlement Development Model. *Domestic Space in LBK Settlements, VML Verlag Marie GmbH,* 183-200 (2013).](http://paperpile.com/b/2vFjDD/d19B6)

47. [Raczky, P. & Anders, A. The woman, the pots, and the cattle figurine. New materiality of an early ALP burial from Polgár-Ferenci-hát. *Multas per Gentes et Multa per Saecula, Amici Magistro et College suo Ioanni Christopho Kozłowski dedicant. Kraków: Institute of Archaeology, Jagiellonian University and Alter Radosław Palonka* 317–328 (2018).](http://paperpile.com/b/2vFjDD/1ByVo)

48. [Domboróczki, L. Preliminary data on the house structures of the LBK settlement at Apc-Berekalja (NE-Hungary). in *Multas per gentes et multa per saecula: Amici magistro et collegae suo ioanni christopho kozłowski dedicant* 361–368 (Jagiellonian University, 2018).](http://paperpile.com/b/2vFjDD/3lZ73)

49. [Domboróczki, L. *et al.* Excavation along the easternmost frontier of the LBK in NE-Hungary at Apc-Berekalja I (2008–2009). *Archaeologiai Értesítő* **141**, 1–27 (2016).](http://paperpile.com/b/2vFjDD/MfY1v)

50. [Bayer, J. Das erste neolithische Gräberfeld in Österreich–Beginn eines neuen Abschnittes der österreichischen Neolithforschung. *Forschung und Fortschritt* (1931).](http://paperpile.com/b/2vFjDD/C68e8)

51. [Lenneis, E. & Neugebauer-Maresch, C. *Das linearbandkeramische Gräberfeld von Kleinhadersdorf*. vol. 82 297–300 (Austrian Academy of Sciences Press, 2015).](http://paperpile.com/b/2vFjDD/GpA1g)

52. [Lebzelter, V. & Zimmermann, G. *Neolithische Gräber Aus Klein-Hadersdorf Bei Poysdorf in Niederösterreich*. (na, 1936).](http://paperpile.com/b/2vFjDD/gkfax)

53. [Tiefenböck, B. Die krankhaften Veränderungen an den linearbandkera-mischen Skelettresten von Kleinhadersdorf, NÖ – ein anthropologischer Beitrag zur Rekonstruktion der Lebensbedingungen im Frühneolithikum. *Diplomarbeit, Universität Wien.* (2010).](http://paperpile.com/b/2vFjDD/xTdTJ)

54. [Stadler, P. Versuch einer Auswertung der 14C-Probenvon Kleinhadersdorf mittels Bayes´scher Statistik, in: Christine Neugebauer-Maresch und Eva Lenneis (Hrsg.), Das linearbandkeramische Gräberfeld von Kleinhadersdorf. *Mitteilungen der prähistorischen Kommission* **82**, 149–152 (2015).](http://paperpile.com/b/2vFjDD/PyMOU)

55. [Lenneis, E. Empty graves in LBK cemeteries – indications of special burial practises. *Doc. Praehist.* **37**, 161–166 (2010).](http://paperpile.com/b/2vFjDD/sqfxB)

56. [Neugebauer-Maresch, C. & Lenneis, E. Das Linearbandkeramische Gräberfeld von Kleinhadersdorf [The Kleinhadersdorf Linearbandkeramik cemetery]. *Vienna: Österreichischen Akademie der* (2015).](http://paperpile.com/b/2vFjDD/0vLI0)

57. [Alexander Bentley, R. Strontium isotopes from the earth to the archaeological skeleton: A review. *J. Archaeol. Method Theory* **13**, 135–187 (2006).](http://paperpile.com/b/2vFjDD/04KOS)

58. [Tiefenböck, B. & Teschler-Nicola, M. Teil II. Anthropologie, in: Christine Neugebauer-Maresch und Eva Lenneis (Hrsg.), Das linearbandkeramische Gräberfeld von Kleinhadersdorf. *Mitteilungen der prähistorischen Kommission* **82**, 298–397 (2015).](http://paperpile.com/b/2vFjDD/IX1cL)

59. [Neugebauer, J.-W. & Gattringer, A. Rettungsgrabungen im Unteren Traisental im Jahre 1988. Siebenter Vorbericht über die Aktivitäten der Abt. f. Bodendenkmale des Bundesdenkmalamtes im Raum St. Pölten-Traismauer. *Fundberichte aus Österreich* **27**, 65–97 (1988).](http://paperpile.com/b/2vFjDD/WaBEm)

60. [Neugebauer, J.-W. & Blesl, C. SG St. Pölten KG Ratzersdorf. *FÖ* **38**, 33 (1999).](http://paperpile.com/b/2vFjDD/VRD2i)

61. [Neugebauer, J. W. Rettungsgrabungen im Unteren Traisental in den Jahren 1998 und 1999. *FÖ* **38**, (1999).](http://paperpile.com/b/2vFjDD/Vp0I2)

62. [Pieler, F. Die bandkeramische Siedlung von Ratzersdorf bei St. Pölten (NÖ). *DUPA* **112**, 2–9 (2004).](http://paperpile.com/b/2vFjDD/9zUnv)

63. [Neugebauer, J.-W. & Blesl, C. SG St. Pölten, KG Ratzersdorf (Fundbericht). *FÖ* **39**, 20 (2000).](http://paperpile.com/b/2vFjDD/2My6k)

64. [Neugebauer, J. W. Rettungsgrabungen im Unteren Traisental in den Jahren 2000 und 2001. *Vorbericht über die Aktivitäten der Abteilung fur Bodendenkmale des Bundesdenkmalamtes im Raum St. Polten-Traismauer* **16**, 191–201 (2001).](http://paperpile.com/b/2vFjDD/jpnKv)

65. [Blesl, C. SG St. Pölten, KG Ratzersdorf an der Traisen. Industriegelände östlich der Bundesstraße. *FÖ* **40**, 199–200 (2001).](http://paperpile.com/b/2vFjDD/DOkgA)

66. [Preinfalk, F. SG St. Pölten, KG Ratzersdorf an der Traisen Industriegelände östlich der Bundesstraße B1. *FÖ*  **40**, 200 (2001).](http://paperpile.com/b/2vFjDD/WFVLG)

67. [Windl, H. J. Zehn Jahre Grabung Schletz, VB Mistelbach, NÖ. *Archäologie Österreichs* **5**, 11–18 (1994).](http://paperpile.com/b/2vFjDD/SeQ8o)

68. [Windl, H. J. Erdwerke der Linearbandkeramik in Asparn an der Zaya/Schletz, Niederösterreich. *Preist. alp.* 137–144 (2001).](http://paperpile.com/b/2vFjDD/0NNSk)

69. [Windl, H. J. Grabungen in einem Siedlungsareal der Linearbandkeramik in Asparn/Zaya, BH Mistelbach, Niederösterreich. *Zalai Múzeum* **2**, 19–21 (1990).](http://paperpile.com/b/2vFjDD/pSUy0)

70. [Windl, H. J. Weitere völkerwanderungszeitliche Gräber aus Schletz, MG Asparn an der Zaya, VB Mistelbach, Niederösterreich. *FÖ* **35**, 377–387 (1996).](http://paperpile.com/b/2vFjDD/e63pe)

71. [Windl, H. J. Zur Stratigraphie der bandkeramischen Grabenwerke von Asparn an der Zaya-Schletz. *Krisen-Kulturwandel-Kontinuitäten. Zum Ende der Bandkeramik in Mitteleuropa* 191–196 (2009).](http://paperpile.com/b/2vFjDD/4uwhk)

72. [Windl, H. J. *Die Siedlung Der Völkerwanderungszeit in Schletz, MG Asparn/Zaya, NÖ*. (na, 2002).](http://paperpile.com/b/2vFjDD/j2SU3)

73. [Kitzig, J. & Ramminger, B. Use and distribution of colourants in Western LBK sites. *Anthropologica et Praehistorica* **126**, 179–186 (2016).](http://paperpile.com/b/2vFjDD/Aafks)

74. [Wild, E. M. *et al.* Neolithic Massacres: Local Skirmishes or General Warfare in Europe? *Radiocarbon* **46**, 377–385 (2004).](http://paperpile.com/b/2vFjDD/VII5s)

75. [Stadler, P. & Kotova, N. Early Neolithic settlement from Brunn Wolfholz in Lower Austria and the problem of the origin of (Western) LBK. *Neolithization of the Carpathian basin: northernmost distribution of the Starčevo/Körös culture* 325–348 (2010).](http://paperpile.com/b/2vFjDD/Rur7r)

76. [Teschler-Nicola, M., Gerold, F., Kanz, F., Lindenbauer, K. & Spannagl, M. Anthropologische Spurensicherung: Die traumatischen und postmortalen Veränderungen an den linearbandkeramischen Skelettresten von Asparn/Schletz. *Archäologie Österreichs* **7**, 4–12 (1996).](http://paperpile.com/b/2vFjDD/CLoxh)

77. [Pavúk, J. Neolithisches Gräberfeld in Nitra. *Slovenská Archeológia* **20**, 5–106 (1972).](http://paperpile.com/b/2vFjDD/oHgo8)

78. [Tvrdý, Z. Anthropology of the neolithic population from Nitra-Horné Krškany (Slovakia). *Anthropologie* **54**, 231–284 (2016).](http://paperpile.com/b/2vFjDD/eWTKc)

79. [Whittle, A. *et al.* Moravia and western Slovakia. in *The first farmers of central Europe: Diversity in LBK lifeways* 101–158 (Oxbow Books, 2013).](http://paperpile.com/b/vSwLSt/tUxc)

80. [Griffiths, S. Radiocarbon dates from Nitra, Schwetzingen and Vedrovice. *The first farmers of Central Europe: diversity in LBK lifeways*](http://paperpile.com/b/2vFjDD/wvb3n). 443-457. [(Oxbow Books, 2013).](http://paperpile.com/b/vSwLSt/tUxc)

81. [van der Velde, P. On Bandkeramik social structure Leiden,. *Netherlands: nalecta Praehistorica Leidensia* **12**, (1979).](http://paperpile.com/b/2vFjDD/gqyPo)

82. [Dočkalová, M. & Čižmář, Z. Neolithic children burials at Moravian settlements in the Czech Republic. *Anthropologie* **45**, 31–60 (2007).](http://paperpile.com/b/2vFjDD/Kn7Un)

83. [Dočkalová, M. & Čižmář, Z. Neolithic settlement burials of adult and juvenile individuals in Moravia, Czech Republic. *Anthropologie* **46**, 37–76 (2008).](http://paperpile.com/b/2vFjDD/XJQSB)

84. [Jarošová, I. & Tvrdý, Z. Diet and diversity of early farmers in Neolithic period ( LBK): Buccal dental microwear and stable isotopic analysis at Vedrovice (Czech Republic) and Nitra - Horné Krškany (Slovakia). *Anthropologie* **55**, 353–384 (2017).](http://paperpile.com/b/2vFjDD/z4nfC)

85. [Masclans, A., Tvrdý, Z., Pavúk, J., Cheben, M. & Bickle, P. Exploring sexual division of labour at ‘Nitra Horné Krškany’ cemetery using stone tool use-wear analysis, physical activity markers, diet, and mobility as proxies. *Archaeol. Anthropol. Sci.* **13**, 109 (2021).](http://paperpile.com/b/2vFjDD/9DFCw)

86. [Rech, M. Zu den ältesten Bauopfern in Mittel-und Nordeuropa. *Festschrift für Hermann Müller-Karpe zum* (1995).](http://paperpile.com/b/2vFjDD/06sbW)

87. [Jakab, J. Kostry dvoch žien zo základového žľabu na neolitickom sídlisku v Jelšovciach. *Otázky neolitu a eneolitu našich krajín–1998, Nitra* (1999).](http://paperpile.com/b/2vFjDD/XsLeI)

88. [Bátora, J. Nález kostier v základovom žlabe domu želiezovskej skupiny v Jelšovciach. *Otázky neolitu a eneolitu našich krajín. Nitra* (1999).](http://paperpile.com/b/2vFjDD/CrNq9)

89. [Plesl, E. Pohřebiště lidu s volutovou keramikou v Mlynárcích na Slovensku. *Archeologické rozhledy IV* (1952).](http://paperpile.com/b/2vFjDD/wSykL)

90. [Steklá, M. Pohřby lidu s volutovou a vypíchanou keramikou. *Archeol. Rozhl.* (1956).](http://paperpile.com/b/2vFjDD/YPsC8)

91. [Farkaš, Z. Nálezy ľudských pozostatkov v prostredí kultúry ľudu s lineárnou keramikou na Slovensku. *Archeologické rozhledy* **54**, 23–43 (2002).](http://paperpile.com/b/2vFjDD/bqslK)

92. [Novotny, B. *Počiatky Vytvarného Prejavu Na Slovensku*. vol. 13 (Slovenské vydavatel̕stvo krásnej literatúry, 1958).](http://paperpile.com/b/2vFjDD/xNjPw)

93. [Kuzma, I., Jakab, J., Kopèeková, M. & Sav, A. Ú. Pohreby na sídlisku ludanickej skupiny v Nitre-Mlynárciach. *Živá archeologie, Supplementum* **3**, 65 – 76 (2010).](http://paperpile.com/b/2vFjDD/EZhPX)

94. [Gabulová, M. & Kuzma, I. Pochovávanie na sídlisku ludanickej skupiny v Nitre-Mlynárciach. *Slovenská archeológia (Slovak Archaeology)* **2**, 179 – 208 (2015).](http://paperpile.com/b/2vFjDD/qrSmv)

95. [Ramsey, C. B. Bayesian Analysis of Radiocarbon Dates. *Radiocarbon* **51**, 337–360 (2009).](http://paperpile.com/b/2vFjDD/FR5Yq)

96. [Reimer, P. J. *et al.* The IntCal20 Northern Hemisphere Radiocarbon Age Calibration Curve (0–55 cal kBP). *Radiocarbon* **62**, 725–757 (2020).](http://paperpile.com/b/2vFjDD/S8Vzz)

97. [Hamilton, J. *et al.* Seeking diversity: Methodology. in *The First Farmers of Central Europe: Diversity in LBK Lifeways* 29–48 (Ox Bow Press, 2013).](http://paperpile.com/b/2vFjDD/cErZY)

98. [Hedges, R. *et al.* The first farmers of central Europe. Diversity in LBK lifeways. (2013).](http://paperpile.com/b/2vFjDD/NPaGe)

99. [Bickle, P. Stable isotopes and dynamic diets: The Mesolithic-Neolithic dietary transition in terrestrial central Europe. *Journal of Archaeological Science: Reports* **22**, 444–451 (2018).](http://paperpile.com/b/2vFjDD/WiCnU)

100. [Bentley, A. Kinship and mobility during the prehistoric spread of farming: isotope evidence from the skeletons. *General Anthropology* **13**, 1–10 (2006).](http://paperpile.com/b/2vFjDD/eXSAF)

101. [Whittle, A. *et al.* The first farmers of Central Europe. *Diversity in LBK lifeways (Barnsley 2016)* **1**, (2013).](http://paperpile.com/b/2vFjDD/vnhYW)

102. [Pinhasi, R. *et al.* Optimal Ancient DNA Yields from the Inner Ear Part of the Human Petrous Bone. *PLoS One* **10**, e0129102 (2015).](http://paperpile.com/b/2vFjDD/KFmap)

103. [Sirak, K. *et al.* Human auditory ossicles as an alternative optimal source of ancient DNA. *Genome Res.* **30**, 427–436 (2020).](http://paperpile.com/b/2vFjDD/vVqKd)

104. [Rohland, N., Glocke, I., Aximu-Petri, A. & Meyer, M. Extraction of highly degraded DNA from ancient bones, teeth and sediments for high-throughput sequencing. *Nat. Protoc.* **13**, 2447–2461 (2018).](http://paperpile.com/b/2vFjDD/B0emc)

105. [Dabney, J. *et al.* Complete mitochondrial genome sequence of a Middle Pleistocene cave bear reconstructed from ultrashort DNA fragments. *Proc. Natl. Acad. Sci. U. S. A.* **110**, 15758–15763 (2013).](http://paperpile.com/b/2vFjDD/y1U6A)

106. [Rohland, N., Harney, E., Mallick, S., Nordenfelt, S. & Reich, D. Partial uracil–DNA–glycosylase treatment for screening of ancient DNA. *Philos. Trans. R. Soc. Lond. B Biol. Sci.* **370**, 20130624 (2015).](http://paperpile.com/b/2vFjDD/Rt7Fu)

107. [Gansauge, M.-T., Aximu-Petri, A., Nagel, S. & Meyer, M. Manual and automated preparation of single-stranded DNA libraries for the sequencing of DNA from ancient biological remains and other sources of highly degraded DNA. *Nat. Protoc.* **15**, 2279–2300 (2020).](http://paperpile.com/b/2vFjDD/8OeA8)

108. [Briggs, A. W. *et al.* Removal of deaminated cytosines and detection of in vivo methylation in ancient DNA. *Nucleic Acids Res.* **38**, e87 (2010).](http://paperpile.com/b/2vFjDD/Svsim)

109. [Fu, Q. *et al.* A revised timescale for human evolution based on ancient mitochondrial genomes. *Curr. Biol.* **23**, 553–559 (2013).](http://paperpile.com/b/2vFjDD/Bw5ma)

110. [Haak, W. *et al.* Massive migration from the steppe was a source for Indo-European languages in Europe. *Nature* **522**, 207–211 (2015).](http://paperpile.com/b/2vFjDD/FtgOj)

111. [Mathieson, I. *et al.* Genome-wide patterns of selection in 230 ancient Eurasians. *Nature* **528**, 499–503 (2015).](http://paperpile.com/b/2vFjDD/MaYt2)

112. [Fu, Q. *et al.* An early modern human from Romania with a recent Neanderthal ancestor. *Nature* **524**, 216–219 (2015).](http://paperpile.com/b/2vFjDD/rLX9g)

113. [Li, H. & Durbin, R. Fast and accurate short read alignment with Burrows-Wheeler transform. *Bioinformatics* **25**, 1754–1760 (2009).](http://paperpile.com/b/2vFjDD/Rxwsd)

114. [Behar, D. M. *et al.* A ‘Copernican’ reassessment of the human mitochondrial DNA tree from its root. *Am. J. Hum. Genet.* **90**, 675–684 (2012).](http://paperpile.com/b/2vFjDD/Ps4cf)

115. [Picard-tools.](http://paperpile.com/b/2vFjDD/TcdYb) <http://broadinstitute.github.io/picard.>

116. [Nikitin, A. G. *et al.* Interactions between earliest Linearbandkeramik farmers and central European hunter gatherers at the dawn of European Neolithization. *Sci. Rep.* **9**, 19544 (2019).](http://paperpile.com/b/2vFjDD/gXzgO)

117. [Lazaridis, I. *et al.* Genomic insights into the origin of farming in the ancient Near East. *Nature* **536**, 419–424 (2016).](http://paperpile.com/b/2vFjDD/Gqr9A)

118. [Harney, É. *et al.* A minimally destructive protocol for DNA extraction from ancient teeth. *Genome Res.* **31**, 472–483 (2021).](http://paperpile.com/b/2vFjDD/d3Y7Y)

119. [Rivollat, M. *et al.* Ancient genome-wide DNA from France highlights the complexity of interactions between Mesolithic hunter-gatherers and Neolithic farmers. *Sci Adv* **6**, eaaz5344 (2020).](http://paperpile.com/b/2vFjDD/EBkKz)

120. [Patterson, N. *et al.* Ancient admixture in human history. *Genetics* **192**, 1065–1093 (2012).](http://paperpile.com/b/2vFjDD/EvHav)

121. [Patterson, N. *et al.* Large-scale migration into Britain during the Middle to Late Bronze Age. *Nature* **601**, 588–594 (2022).](http://paperpile.com/b/2vFjDD/Y0ADE)

122. [Chintalapati, M., Patterson, N. & Moorjani, P. The spatiotemporal patterns of major human admixture events during the European Holocene. *Elife* **11**, (2022).](http://paperpile.com/b/2vFjDD/exOV)

123. [Rubinacci, S., Ribeiro, D. M., Hofmeister, R. J. & Delaneau, O. Efficient phasing and imputation of low-coverage sequencing data using large reference panels. *Nat. Genet.* **53**, 120–126 (2021).](http://paperpile.com/b/2vFjDD/qzZxq)

124. [Ringbauer, H. *et al.* Accurate detection of identity-by-descent segments in human ancient DNA. *Nat. Genet.* **56**, 143–151 (2024).](http://paperpile.com/b/2vFjDD/oe44t)

125. [Danecek, P., McCarthy, S. & Li, H. bcftools—utilities for variant calling and manipulating vcfs and bcfs. *The MIT/Expat License or GPL License, see the* (2015).](http://paperpile.com/b/2vFjDD/72Sef)

126. [Maples, B. K., Gravel, S., Kenny, E. E. & Bustamante, C. D. RFMix: a discriminative modeling approach for rapid and robust local-ancestry inference. *Am. J. Hum. Genet.* **93**, 278–288 (2013).](http://paperpile.com/b/2vFjDD/rmjda)

127. [Bickle, P. & Whittle, A. LBK lifeways: a search for difference. *The first farmers of central Europe. Diversity in LBK lifeways (Oxford 2013)* 1–27 (2013).](http://paperpile.com/b/2vFjDD/tcRR5)

128. [Fournier, R., Tsangalidou, Z., Reich, D. & Palamara, P. F. Haplotype-based inference of recent effective population size in modern and ancient DNA samples. *Nat. Commun.* **14**, 7945 (2023).](http://paperpile.com/b/2vFjDD/6A2d)

129. [Purcell, S. *et al.* PLINK: a tool set for whole-genome association and population-based linkage analyses. *Am. J. Hum. Genet.* **81**, 559–575 (2007).](http://paperpile.com/b/2vFjDD/XPsGD)

130. [1000 Genomes Project Consortium *et al.* An integrated map of genetic variation from 1,092 human genomes. *Nature* **491**, 56–65 (2012).](http://paperpile.com/b/2vFjDD/TWY38)

131. [Sherry, S. T. *et al.* dbSNP: the NCBI database of genetic variation. *Nucleic Acids Res.* **29**, 308–311 (2001).](http://paperpile.com/b/2vFjDD/ikEZE)

132. [Wang, K., Li, M. & Hakonarson, H. ANNOVAR: functional annotation of genetic variants from high-throughput sequencing data. *Nucleic Acids Res.* **38**, e164 (2010).](http://paperpile.com/b/2vFjDD/2Mmik)

133. [O’Leary, N. A. *et al.* Reference sequence (RefSeq) database at NCBI: current status, taxonomic expansion, and functional annotation. *Nucleic Acids Res.* **44**, D733–45 (2016).](http://paperpile.com/b/2vFjDD/Sqkhh)

134. [Liu, X., Li, C., Mou, C., Dong, Y. & Tu, Y. dbNSFP v4: a comprehensive database of transcript-specific functional predictions and annotations for human nonsynonymous and splice-site SNVs. *Genome Med.* **12**, 103 (2020).](http://paperpile.com/b/2vFjDD/hYxTz)

135. [Szpiech, Z. A. selscan 2.0: scanning for sweeps in unphased data. *Bioinformatics* **40**, (2024).](http://paperpile.com/b/2vFjDD/A4YQ)

136. [Siewert, K. M. & Voight, B. F. Detecting Long-Term Balancing Selection Using Allele Frequency Correlation. *Mol. Biol. Evol.* **34**, 2996–3005 (2017).](http://paperpile.com/b/2vFjDD/abFIC)

137. [Chang, C. C. *et al.* Second-generation PLINK: rising to the challenge of larger and richer datasets. *Gigascience* **4**, 7 (2015).](http://paperpile.com/b/2vFjDD/WS09N)

138. [Wigginton, J. E., Cutler, D. J. & Abecasis, G. R. A note on exact tests of Hardy-Weinberg equilibrium. *Am. J. Hum. Genet.* **76**, 887–893 (2005).](http://paperpile.com/b/2vFjDD/dGc7y)

139. [Ferrer-Admetlla, A., Liang, M., Korneliussen, T. & Nielsen, R. On detecting incomplete soft or hard selective sweeps using haplotype structure. *Mol. Biol. Evol.* **31**, 1275–1291 (2014).](http://paperpile.com/b/2vFjDD/BA1IC)

140. [D’Mello, S. A. N., Finlay, G. J., Baguley, B. C. & Askarian-Amiri, M. E. Signaling pathways in melanogenesis. *Int. J. Mol. Sci.* **17**, 1144 (2016).](http://paperpile.com/b/2vFjDD/PYYJT)

141. [Haltaufderhyde, K. D. & Oancea, E. Genome-wide transcriptome analysis of human epidermal melanocytes. *Genomics* **104**, 482–489 (2014).](http://paperpile.com/b/2vFjDD/rTeGb)

142. [Yin, L. *et al.* Identification of Genes Expressed in Hyperpigmented Skin Using Meta-Analysis of Microarray Data Sets. *J. Invest. Dermatol.* **135**, 2455–2463 (2015).](http://paperpile.com/b/2vFjDD/cXGv4)

143. [Cheng, X. & DeGiorgio, M. Flexible Mixture Model Approaches That Accommodate Footprint Size Variability for Robust Detection of Balancing Selection. *Mol. Biol. Evol.* **37**, 3267–3291 (2020).](http://paperpile.com/b/2vFjDD/cFAMA)

144. [Bitarello, B. D. *et al.* Signatures of Long-Term Balancing Selection in Human Genomes. *Genome Biol. Evol.* **10**, 939–955 (2018).](http://paperpile.com/b/2vFjDD/PQhPO)
